# c-Myc and viral cofactor Kaposin B co-operate to elicit angiogenesis through modulating miRNome traits of endothelial cells

**DOI:** 10.1186/s12918-015-0242-3

**Published:** 2016-01-11

**Authors:** Hsin-Chuan Chang, Tsung-Han Hsieh, Yi-Wei Lee, Cheng-Fong Tsai, Ya-Ni Tsai, Cheng-Chung Cheng, Hsei-Wei Wang

**Affiliations:** Institute of Microbiology and Immunology, National Yang-Ming University, Taipei, Taiwan; Institute of Biomedical Informatics, National Yang-Ming University, Taipei, Taiwan; Division of Cardiology, Department of Internal Medicine, Tri-Service General Hospital, National Defense Medical Center, Taipei, Taiwan; VGH-YM Genome Research Center, National Yang-Ming University, Taipei, Taiwan

**Keywords:** MicroRNAs, Angiogenesis, KS-associated herpesvirus, c-Myc, Kaposin B, Small RNA sequencing

## Abstract

**Background:**

MicroRNAs (miRNAs) have emerged as master regulators of angiogenesis and other cancer-related events. Discovering new angiogenesis-regulating microRNAs (angiomiRs) will eventually help in developing new therapeutic strategies for tumor angiogenesis and cardiovascular diseases. Kaposi’s sarcoma (KS), which is induced by the etiological infectious agent KS-associated herpesvirus (KSHV), is a peculiar neoplasm that expresses both blood and lymphatic endothelial markers and possesses extensive neovasculature. Using KSHV and its proteins as baits will be an efficient way to discover new angiomiRs in endothelial cells. Kaposin B is one of the latent viral genes and is expressed in all KSHV tumor cells. Since Kaposin B is a nuclear protein with no DNA-binding domain, it may regulate gene expression by incorporating itself into a transcription complex.

**Results:**

We demonstrated that c-Myc and Kaposin B form a transcription complex and bind to the miR-221/-222 promoter, thereby affecting their expression and anti-angiogenic ability. By small RNA sequencing (smRNA-Seq), we revealed that 72.1 % (173/240) of Kaposin B up-regulated and 46.5 % (113/243) of Kaposin B down-regulated known miRNAs were regulated by c-Myc. We also found that 77 novel miRNA were up-regulated and 28 novel miRNAs were down-regulated in cells expressing both c-Myc and Kaposin B compared with cells expressing Kaposin B only. The result was confirmed by RNA-IP-seq data.

**Conclusions:**

Our study identifies known and novel c-Myc-regulated microRNAs and reveals that a c-Myc-oriented program is coordinated by Kaposin B in KSHV-infected cells.

**Electronic supplementary material:**

The online version of this article (doi:10.1186/s12918-015-0242-3) contains supplementary material, which is available to authorized users.

## Background

Kaposi’s sarcoma-associated herpesvirus (KSHV), also known as human herpesvirus 8 (HHV8), is associated with Kaposi’s sarcoma (KS) [1]. KS is the most common cancer in individuals with AIDS and one of the most common cancers in patients who have immunosuppression [2]. KS typically appears as colored lesions or blotches on the skin, although it can spread to internal organs [3]. This disease has been recognized as a peculiar neoplasm that has a diverse cellular makeup and an extensive neovasculature. In host endothelial cells, KSHV infection alters the expression of mRNAs and microRNAs required for many aspects of tumorigenesis, including angiogenesis [4]. Therefore, KS and KSHV are recognized as an excellent model to study angiogenesis and to discover new angiogenic mechanisms, including coding and non-coding RNAs.

Recently, the effect of KSHV infection on cellular microRNA (miRNAs) expression has been reported [5]. It was found that pre-miRNA signatures delineate the stages of KSHV-associated HUVEC transformation [6, 7]. MiRNAs are small RNAs of 18–24 nucleotides in length that have emerged as master regulators of cancer and key modulators of angiogenesis [8–10]. An increasing number of studies have shown that some miRNAs, called angiomiRs (microRNAs that regulate angiogenesis), play a crucial role in regulating angiogenesis [11]. For example, miR-221 and miR-222, two closely related miRNAs encoded in cluster from a genomic region on chromosome X, can modulate the angiogenic properties of human umbilical vein endothelial cells (HUVECs) by targeting c-Kit [11] and endothelial nitric oxide synthase (eNOS) [12]. MiR-221 and miR-222 also regulate lymphatic endothelial cell (LEC) motility by targeting ETS2 and ETS1 transcription factors, respectively [4]. Loss of miR-221 expression demarks the transition from merely immortalized to fully tumorigenic HUVECs [6]. KSHV also induces endothelial cell motility by inducing miR-31 expression while suppressing that of miR-221 and miR-222 [4, 13].

Kaposin B, one of the three Kaposin proteins encoded from the KSHV genome K12 locus, is one of the viral genes expressed during latency and is therefore expressed in all KSHV tumor cells [4]. Kaposin B stabilizes various cytokine (such as IL6 and GM-CSF) mRNA containing AU-rich elements (AREs) *via* the p38/MK2 pathway. In response to LPS, Kaposin B and MK2 were shown to be exported to the cytoplasm, where mRNA stability is regulated [14]. Kaposin B also enhances the PROX1 mRNA stability during lymphatic reprogramming of vascular endothelial cells [15]. Kaposin B can influence cellular gene expression by regulating promoter activities of host genes: both Kaposin B and KSHV viral latency-associated nuclear antigen (LANA) proteins can down-regulate miR-221 and miR-222 levels by repressing the activity of miR-221/-222 cluster promoter [4]. Since there is no predictable DNA-binding domain on Kaposin B, how this nuclear protein can regulate mRNA and miRNA expression remains unclear.

c-Myc achieves its oncogenic effects by regulating transcription of protein-coding genes as well as microRNA genes such as miR-29b-1/miR-29a [16, 17]. c-Myc is also essential for vasculogenesis and angiogenesis during development and tumor progression [18] via inducing the expression of miR-17 ~ 92 angiogenic miRNA cluster [19]. Revealing the angiomiRs regulated by c-Myc and the underlying regulatory mechanisms will help to further understand c-Myc and endothelial cell biology.

Here, we showed that Kaposin B and c-Myc are in the same transcription complex that directly regulates the miR-221/-222 cluster promoter activity. A c-Myc-oriented circuit is therefore formed in the presence of Kaposin B in KSHV-infected endothelial cells. Furthermore, we also provide a global microRNA signature which is regulated by c-Myc and Kaposin B. We hope our roadmap will help the search and development of new therapeutic targets for virus- or cancer-induced angiogenesis, cancer formation and metastasis.

## Methods

### Cell culture and KSHV infection

Human primary umbilical vein endothelial cells (HUVECs) were purchased from Clonetics Inc. (Walkersville, Md.) and were cultured as described [4]. HMEC1, an immortalized human microvascular endothelial cell line, was cultured in endothelial cell growth medium MV (C-22020; PromoCell, Heidelberg, Germany). A recombinant virus, rKSHV.219, that expresses the red fluorescent protein (RFP) from the KSHV lytic PAN promoter, the green fluorescent protein (GFP) from the EF-1alpha promoter, and with a gene for puromycin resistance as a selectable marker, was constructed using JSC-1 cells as described previously [20].

### Plasmid construction

Plasmids expressing KSHV Kaposin B and miR-221, miR-222, or miR-221/-222 were constructed as described previously [4]. c-Myc expression constructs (pcDNA3-HA-c-Myc and pHR-c-Myc) and knock down construct (pLKO.1) was kindly provided by Prof. Kenneth CW Wu [21]. The full-length miR-221/-222 promoter reporter plasmid was constructed as described previously [4], and primers for cloning miR-221/-222 promoter mutants are listed in Additional file 1.

### Immunofluorescence assay (IFA)

Cells were fixed with 4 % paraformaldehyde (Sigma-Aldrich), permeabilized with 0.2 % Triton X-100 (Sigma-Aldrich), and then blocked with PBS containing 1 % bovine serum albumin (Sigma-Aldrich). For Kaposin B staining, cells were incubated with the monoclonal antibody anti-FLAG M2 at a 1:500 dilution for 60 to 120 min at 25 °C followed by incubation with FITC-conjugated goat anti-Mouse IgG (1:500, Jackson ImmunoResearch) for 60 to 120 min at 25 °C. Rhodamine-phalloidin (Molecular Probes, Invitrogen) was used to label actin cytoskeleton. Cell nuclei were counterstained with Hoechst 33342 (Sigma-Aldrich), and examined by fluorescence confocal microscopy (Olympus FV1000).

### Transwell cell migration and endothelial cell tube formation assays

Cell migration ability was evaluated using Costar Transwell® Polycarbonate Permeable Supports (Corning, NY, USA) as described previously [13]. In brief, 5 × 10^4^ cells in 500 μl of culture medium were applied to the upper chamber of the device, and 750 μl of medium containing 10 ng/ml human VEGF (R&D Systems, Minneapolis, MN, USA) was added to the lower chamber. A polycarbonate membrane with a pore size of 8 μm was placed in between the two chambers. After 6 h of incubation at 37 °C for HMEC1 and HUVEC cells, the membrane was fixed in 4 % paraformaldehyde (Sigma-Aldrich) for 20 min at room temperature and then stained with Hoechst 33342 solution (Sigma-Aldrich) for 30 min. On the upper side of membrane were identified un-migrated cells and removed. The cells under the membrane were counted under a microscope.

In vitro angiogenic activity of vascular endothelial cells was examined by tube formation assays: primary HUVECs were placed onto MatriGel® Basement Membrane Matrix gel (Becton-Dickinson, Franklin Lakes, NJ, USA) for 6 h at 37 °C. The formation of tubule structure was observed every hour under a microscope.

### Small RNA sequencing (smRNA-Seq) and data analysis

Total RNA was collected and small RNA fractions were sequenced using Illumina HiSeq2000 sequencer (Illumina, San Diego, CA USA) according to the manufacturer’s instructions. For data analysis, quality Fastq sequences, which were without poly-A, ambiguous nucleotides or a 5′ adapter, yet flanking 6–18 nt of 3′ adapter sequence, had the adapter sequences trimmed and the identical sequences were then collapsed to unique sequences. The resulting unique sequences that did not align to the mRNA database (UCSC genome browsers) but were aligned to known microRNA sequences (miRBase R20; http://www.mirbase.org/) were subjected to further quantification analysis. Sequencing reads were used to obtain a RPM (reads per million mapped reads) [22, 23] value calculated as C/(MN) × 10^9^, where C is “read numbers aligned to a given miRNA chromosomal region”, M is “multiple mapping numbers across all miRNA regions” and N is “total read numbers that map to human genome sequence”.

### RNA extraction, real-time quantitative polymerase chain reaction (qPCR) and microRNA expression

RNA extraction and reverse transcription were performed as previously described [13]. The expression of mature human miR-221 and miR-222 was determined by a stem-loop real-time PCR system using the primer pairs previously published [4]. MicroRNA expression data were normalized to that of U6 snRNA. Details of qPCR primers are in Additional file 1.

### Coimmunoprecipitation (co-IP), chromatin immunoprecipitation (ChIP) and Immunoblotting

Co-IP and ChIP assays were performed as previously described [24]. Briefly, Kaposin B or c-Myc expression plasmid was co-transfected into 293T cells using TurboFect™ (R0531; Fermentas, Glen Burnie, Maryland, USA). After 48 h, cells were lyzed in NET lysis buffer containing protease and phosphatase inhibitors and cell lysate (500 μl) was immunoprecipitated with anti-FLAG M2 (for detecting FLAG-tagged Kaposin B protein; Sigma-Aldrich), anti-HA or isotype IgG antibody. The immunoprecipitates were probed with anti-FLAG M2 (1:2000) and anti-c-Myc Tag (clone 9E10; MILLIPORE, 05–419, 1:2000) monoclonal antibodies, and visualized with horseradish peroxidase-conjugated secondary antibodies using the ECL reagent (Amersham Pharmacia Biosciences). For chromatin immunoprecipitation assay, chromosomal DNA fragments were prepared as described [25]. Briefly, lysates were incubated with isotype IgG or anti-FLAG antibody. The approximately 100-base pair products from miR-221/-222 proximal promoter (E1 region: −600 (45,607,128-45,607,214); E2/3 region: −1100 (45,607,635-45,607,764); NC region: −2600 (45,609,170-45,609,265)) were detected by real-time PCR.

### Luciferase reporter assays

For reporter assays, 293T cells (5 × 10^4^/well) in 24-well plates were transfected with either Kaposin B or c-Myc expression plasmid, along with the firefly luciferase reporter gene construct (500 ng) and 50 ng pRL-TK *Renilla* luciferase construct (for normalization) using TurboFect™. Cells were harvested 48 h after transfection, and the luciferase activity was measured using the Dual-Luciferase Reporter Assay System (E1910; Promega, Charbonnières, France).

## Results

### Kaposin B binds to c-Myc for regulating endothelial cell angiogenic activities

**Fig. 1:**
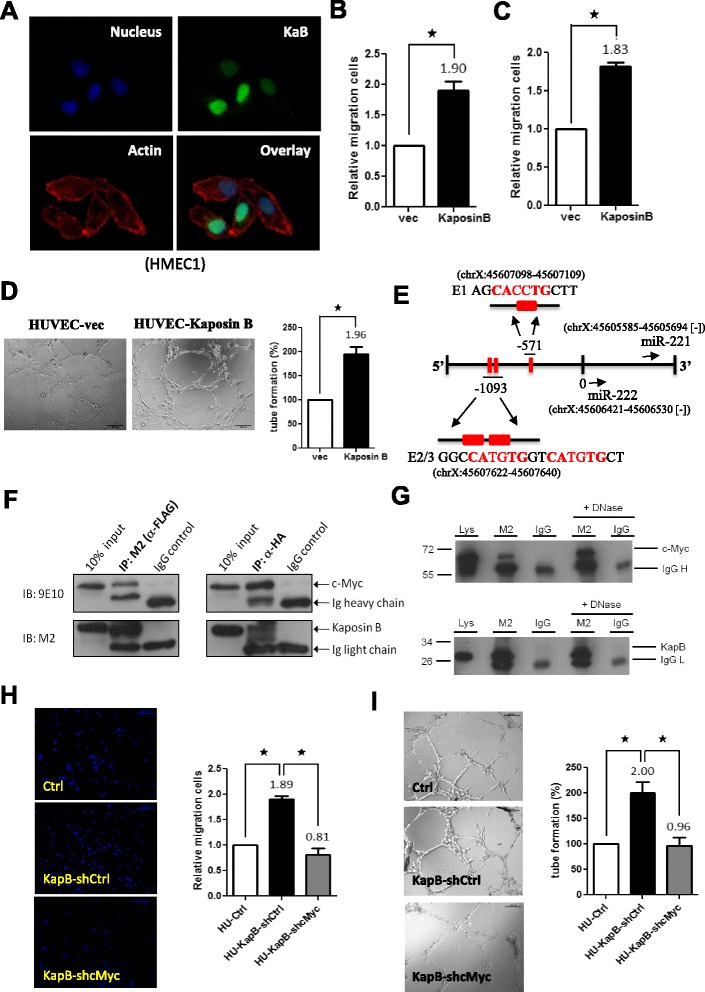
Kaposin B binds to c-Myc for regulating endothelial cell angiogenic activities. **a** Nuclear distribution pattern of KSHV Kaposin B protein in cells. Immunofluorescence staining for Kaposin B proteins. HMEC1 cells with stable Kaposin B (KapB) expression were fixed, and Kaposin B proteins were detected using anti-Flag mAb, followed by anti–mouse IgG secondary antibody conjugated with FITC (*green*). Cell nuclei were counterstained with Hoechst 33342, while actin filaments with Texas Red phalloidin (Alexa Fluor 568). **b** HMEC1 cells stably expressing Kaposin B were subjected to the Transwell cell-migration assay (*n* = 3). **c** Kaposin B increases cell motility in HUVEC. Primary HUVEC stably transduced with Kaposin B or the vector control by lentivirus were used for Transwell cell-migration assays (*n* = 3). **d** Kaposin B enhances microvascular formation of HUVEC in an in vitro MatriGel angiogenesis assay. Pictures were taken after 6 h of incubation (*left*), and tube length was then measured and compared (*right*). **e** Schematic representation of miR-221/-222 proximal promoter. Three E-boxes (E1 and E2/3, in *red*) were found. **f** Co-immunoprecipitation assays show Kaposin B and c-Myc form a protein complex. Cell lysates were prepared from HMEC1 cells stably expressing Kaposin B. Five micrograms of anti-FLAG (clone M2; *left panel*), anti-HA (*right panel*) or isotype IgG control were incubated with 500 μl of cell extracts and then analyzed by western blotting with indicated mAbs. **g** The interaction between c-Myc and Kaposin B was independent of promoter DNA. Co-immunoprecipitation assays were performed with or without DNase pre-treatment on cell lysates prepared from HMEC1 cells stably expressing Kaposin B. Anti-FLAG (clone M2) or isotype IgG control were incubated with cell extracts, and pull-down products were analyzed by western blotting with anti-HA mAb for c-Myc (upper panel) or anti-FLAG M2 mAb for Kaposin B (*lower panel*). **h**-**i** Knockdown of endogenous c-Myc levels in Kaposin B(+) HUVECs inhibits Kaposin B-induced cell migration (**h**, *left*) and microvasculature formation (**i**, *right*) (*n* = 3)

Kaposin B is a nuclear protein which can regulate miR-221 and miR-222 expressions without any predictable DNA-binding domain [4, 26]. Therefore, we hypothesized that Kaposin B may be a novel transcription cofactor which regulates promoter activities by binding to other transcription factor(s). To investigate, we overexpressed Kaposin B in HMEC1 and primary HUVEC cells and tested their migration and tube formation abilities. We verified expression of Kaposin B protein in lentivirus-transduced HUVEC using immunoblotting (Additional file 2-A), and immunofluorescence staining showed that localization of Kaposin B was in nucleus (Fig. 1a). Expression of Kaposin B induced an increase in cell migration rate in HMEC1 and primary HUVEC cells (Fig. 1b-c). Kaposin B also induced more microvascular structures formation by HUVEC in the in vitro tube formation assay (Fig. 1d).

To maintain KSHV latency and oncogenesis, KSHV-latency proteins, LANA and vIRF3, stabilized and activated c-Myc proteins [27–30] and c-Myc was shown to reduce miR-222 expression in mouse mammary tumors [31]. Therefore, we examined the proximal promoter region of miR-221/-222 and identified three putative c-Myc binding sites (E1, E2 and E3 E-boxes with a motif sequence CANNTG) (Fig. 1e). We hypothesized that Kaposin B may associate with c-Myc in regulating host gene expression. We therefore tested whether Kaposin B and c-Myc are in the same protein complex by co-transfecting FLAG-tagged Kaposin B and HA-tagged c-Myc into 293T cells. c-Myc was detected in immunoprecipitation (IP) assays using anti-FLAG monoclonal antibody (mAb) against Kaposin B. On the other hand, Kaposin B was detected in IP assay using anti-HA mAb against c-Myc (Fig. 1f). The interaction between c-Myc and Kaposin B was independent of promoter DNA since c-Myc was still detected in the anti-FLAG mAb pull-down products even when cell lysates were pre-treated with DNase digestion for removal of genomic DNA fragments (Fig. 1g, Additional file 2-B).

We further checked whether c-Myc is required for Kaposin B functions. Knockdown of endogenous c-Myc in Kaposin B-expressing HUVEC cells abolished Kaposin B-induced cell migration and microvasculature formation. (Fig. 1h-i, Additional file 2-C). We also knocked down c-Myc in Kaposin B-expressing HMEC1 cells and found that cell migration ability was down-regulated. (Additional file 2-D-E).

### Small RNA sequencing reveals known miRNAs co-regulated by c-Myc and Kaposin B in primary endothelial cells

Previous study showed that Kaposin B can down-regulate miR-221 and miR-222 levels by repressing the activity of miR-221/-222 cluster promoter [4]. However, there are few studies exploring Kaposin B-induced global miRNAs alteration. Therefore, we overexpressed Kaposin B in HUVEC to examine its regulated miRNA expression. We have demonstrated that c-Myc binds to Kaposin B (Fig. 1f-g) and is required for its functions (Fig. 1h-i), hence we overexpressed c-Myc in Kaposin B(+) HUVEC to examine its ability to augment Kaposin B-regulated miRNA expression.

**Fig. 2:**
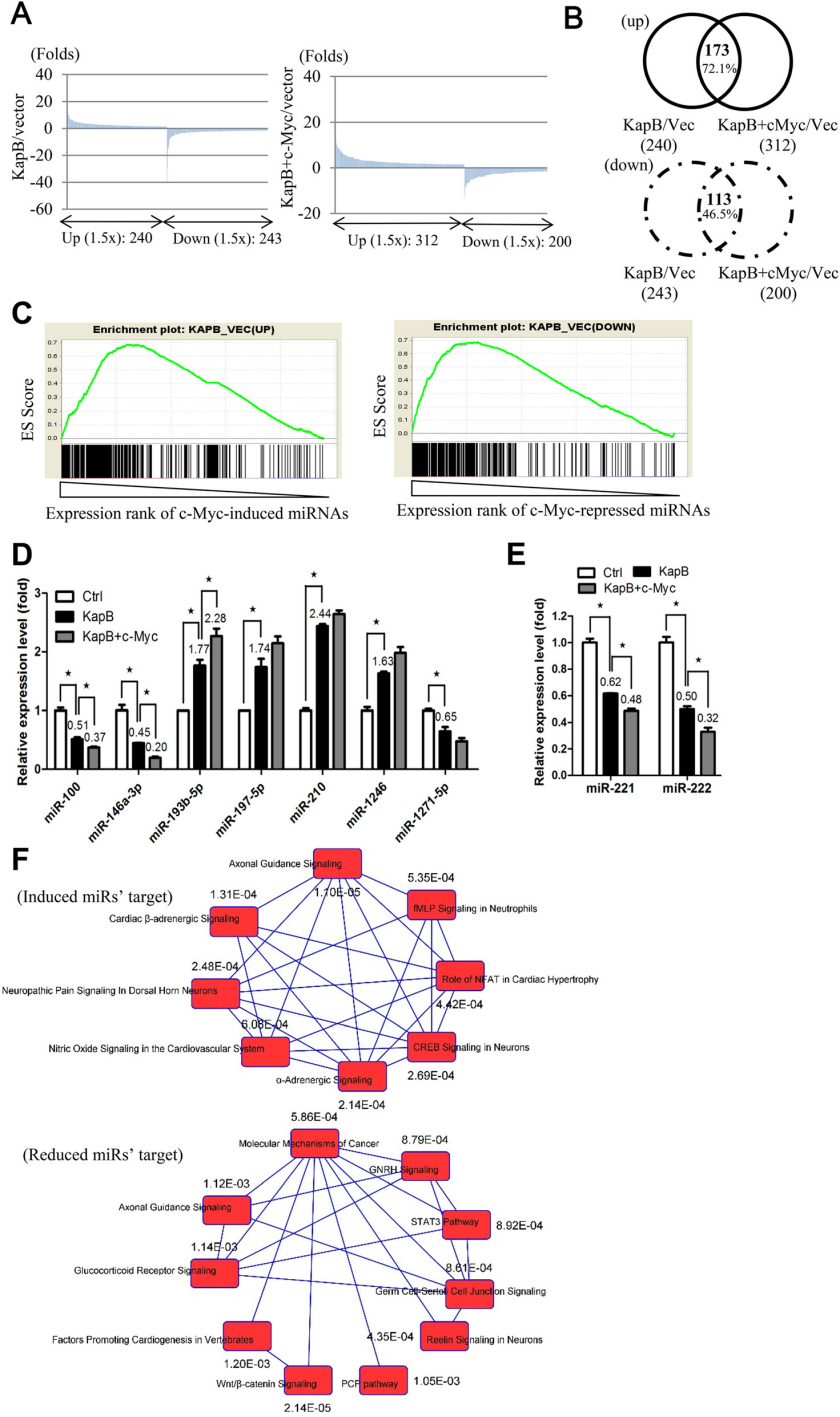
c-Myc cooperates with Kaposin B to regulate cellular known miRNAs. **a** smRNA-Seq revealed differentially expressed miRNAs between Kaposin B(+) and HUVECs (≥1.5 folds, *left*), and between Kaposin B(+)c-Myc(+) and HUVECs (≥1.5 folds, *right*). **b** Venn diagrams summarizing significant overlap between c-Myc and Kaposin B known miRNAs signature. (*upper panel*) up-regulated miRNAs; (*lower panel*) down-regulated miRNAs. **c** Gene Set Enrichment Analysis (GSEA) verified the significant overlap between c-Myc and Kaposin B miRNAs signature. **d** RT-qPCR detected various known miRNAs expression. Mean expression levels of the target miRNAs are compared with the U6 control (*n* = 3). *: *P* < .05. **e** RT-qPCR detected miR-221/miR-222 expression in Kaposin B(+) and Kaposin B(+)c-Myc(+) cells. **f** The biological pathway of genes which were targeted by the Kaposin B(+)c-Myc(+) induced/reduced miRNAs. The *P* value were marked on the figure

We analyzed small RNA sequencing (smRNA-Seq) data according to an in-house bioinformatics pipeline [32]. The Illumina next generation sequencing platform generated more than 29 million high-quality sequencing reads for HUVEC transduced with empty lentiviral vector alone, Kaposin B alone and Kaposin B with c-Myc. The initial operations included identifying sequence matches to the coding RNA database for eliminating reads of degraded mRNAs. Non-coding RNA reads that match previously annotated miRNAs deposited in the miRBase database (release 21) were subjected to normalization and quantitative profiling. Firstly, we explored known miRNAs expression. When we overexpressed Kaposin B alone (KapB/vec), we found 240 up-regulated and 243 down-regulated known miRNAs. In contrast, 312 up-regulated and 200 down-regulated miRNAs (≥1.5 fold change; Fig. 2a) were identified upon simultaneous overexpression of Kaposin B and c-Myc (KapB + c-Myc/vec). We further interrogated the contribution of c-Myc in Kaposin B-regulated miRNome changes. Comparison of the c-Myc(+)Kaposin B(+) miRNA signature with that triggered by Kaposin B alone in HUVEC indicated that c-Myc helped to further induced the expression of 72.1 % (173/240) of Kapsoin B up-regulated miRNAs (Fig. 2b, upper panel, fold change ≥1.5). Similarly, the miRNAs down-regulated by Kaposin B and c-Myc were 46.5 % (113/243); Fig. 2b, lower panel, fold change ≥1.5). c-Myc and Kaposin B-regulated miRNAs are enumerated in Tables 1 and 2 (only top 50 of c-Myc(+)Kaposin B(+) miRNA are listed). Gene Set Enrichment Analysis (GSEA) revealed that the expression of up- or down-regulated miRNAs by c-Myc correlated with those induced or repressed by Kaposin B in HUVEC (Fig. 2d). We also performed qPCR to detect miRNAs expression in Kaposin B(+) and Kaposin B(+)c-Myc(+) cells. We identified that miR-193b-5p, miR-197-5p, miR-210, and miR-1246 were up-regulated in Kaposin B(+) and Kaposin B(+)c-Myc(+) cells. In contrast, miR-100, miR-146a-3p and miR-1271-5p were down-regulated (Fig. 2d). RT-qPCR also demonstrated that miR-221 and miR-222 were down-regulated in KaposinB(+) and Kaposin B(+)c-Myc(+) cells (Fig. 2e). The miRNAs detected in RT-qPCR, except miR-1246 and miR-1271-5p, were similar to the ones detected in KSHV-infected HUVECs (Additional file 3). Moreover, we had used up to 3 different kind of tools (TargetScan (http://www.targetscan.org/vert_70/), mirDB (http://mirdb.org/miRDB/index.html) and TargetMiner (http://www.isical.ac.in/~bioinfo_miu/targetminer20.htm)) to analyze the targeted genes of c-Myc and Kaposin B induced/reduced top 5 miRNAs (Table 1). Based on our results, 806 genes were targeted by the up-regulated miRNAs (Additional file 4-A) and 818 genes were targeted by the down-regulated miRNAs (Additional file 4-B), respectively. The biological pathways were further analyzed, genes targeted by the up-regulated miRNAs contribute to axonal guidance, cardiac β-adrenergic signaling and α-adrenergic signaling; while the genes targeted by the down-regulated miRNAs contribute to Wnt/β signaling, reelin signaling and molecular mechanisms of cancer (Fig. 2f). This indicates Kaposin B + c-myc regulated miRNAs may trigger cells to more tumorigenic stage.

**Table 1:** Top 50 miRNAs induced by both c-Myc and Kaposin B

Name	HU_KapB_cMyc (RPM)	HU_KaposinB (RPM)	cMyc + KapB/vec (Fold)	KapB/vec (Fold)
hsa-miR-5010-5p	2.56436	0.728448	34.99103515	9.93977038
hsa-miR-616-3p	1.08273	0.463558	14.77399565	6.325310904
hsa-miR-3121-3p	0.854786	0.596003	11.66366929	8.132540642
hsa-miR-6851-5p	0.683829	0.52978	9.330938158	7.228918951
hsa-miR-193b-5p	2.56436	1.05956	8.747752819	3.614457009
hsa-miR-6894-5p	1.25369	0.596003	8.553407199	4.066281418
hsa-miR-3163	0.626843	1.25823	8.553356566	17.16871662
hsa-miR-3168	0.626843	0.331113	8.553356566	4.518081167
hsa-miR-30b-3p	1.76656	1.05956	8.034967866	4.819270532
hsa-miR-3607-3p	0.569857	0.26489	7.775774975	3.614459475
hsa-miR-4804-5p	0.569857	0.26489	7.775774975	3.614459475
hsa-miR-483-5p	1.13971	0.993338	7.775768905	6.777133422
hsa-miR-4647	1.02574	0.927115	6.998198837	6.325321344
hsa-miR-7854-3p	1.02574	0.331113	6.998198837	2.259046748
hsa-miR-6735-5p	2.7923	1.58934	6.35022071	3.614461119
hsa-miR-3188	0.455886	0.198668	6.220625438	2.710851429
hsa-let-7e-5p	2636.49	1690.55	6.098609794	3.910504036
hsa-miR-197-5p	3.53312	2.58268	6.026232752	4.405123744
hsa-miR-210-5p	7.1802	6.15869	5.763201618	4.943284612
hsa-miR-3651	1.25369	0.860893	5.70224553	3.915659582
hsa-miR-1909-3p	0.3989	0.397335	5.443043847	5.421689213
hsa-miR-483-3p	1.9945	1.4569	5.443043847	3.975919068
hsa-miR-3173-5p	4.3879	1.78801	5.443038446	2.217964669
hsa-miR-3197	1.1967	0.79467	5.443033944	3.614452899
hsa-miR-139-3p	3.87503	2.31779	5.287530258	3.162655452
hsa-miR-423-5p	5484.82	3592.57	5.221947179	3.420387684
hsa-let-7f-5p	29803.4	20292.6	4.887141234	3.327566727
hsa-miR-1284	1.42464	0.52978	4.859847516	1.807228505
hsa-miR-3613-5p	3.47613	1.72179	4.743225873	2.349405482
hsa-miR-3120-3p	0.341914	0.132445	4.665462256	1.807229738
hsa-miR-4436b-3p	1.70957	1.12578	4.665462256	3.072283731
hsa-miR-4501	0.341914	0.728448	4.665462256	9.93977038
hsa-miR-497-3p	0.341914	0.331113	4.665462256	4.518081167
hsa-miR-544a	0.341914	0.132445	4.665462256	1.807229738
hsa-miR-664b-5p	0.341914	0.331113	4.665462256	4.518081167
hsa-miR-6862-5p	0.341914	0.26489	4.665462256	3.614459475
hsa-miR-7107-3p	0.341914	0.26489	4.665462256	3.614459475
hsa-miR-6816-3p	1.02574	0.596003	4.665444671	2.710841949
hsa-let-7a-5p	29230.6	20145.4	4.632835878	3.19289826
hsa-miR-210-3p	66.5023	47.8789	4.606251818	3.316310416
hsa-miR-3145-3p	0.626843	0.397335	4.276689954	2.710852004
hsa-miR-3675-5p	0.626843	0.596003	4.276689954	4.066281418
hsa-miR-6515-5p	1.1967	0.662225	4.0822801	2.259035631
hsa-miR-6797-5p	0.569857	0.397335	3.887898098	2.710852004
hsa-miR-3183	0.284929	0.26489	3.88789431	3.614459475
hsa-miR-372-3p	0.284929	0.728448	3.88789431	9.93977038
hsa-miR-4488	0.284929	0.198668	3.88789431	2.710851429
hsa-miR-6797-3p	0.284929	0.198668	3.88789431	2.710851429
hsa-miR-548ar-3p	0.142464	0.132445	3.887880665	3.614459475
hsa-miR-6511b-3p	2.29842	1.2803	3.763465686	2.096381478

**Table 2:** Top 50 miRNAs reduced by both c-Myc and Kaposin B

Name	HU_KapB_cMyc (RPM)	HU_KaposinB (RPM)	cMyc + KapB/vec (Fold)	KapB/vec (Fold)
hsa-miR-3614-5p	25.7006	35.6277	0.117247798	0.162535869
hsa-miR-548f-5p	0.0121636	0.0245914	0.124207975	0.251113814
hsa-miR-4723-5p	0.0569857	0.0662225	0.129596309	0.150602547
hsa-miR-9-5p	2.10847	7.9467	0.139661522	0.526376101
hsa-miR-412-3p	0.170957	0.728448	0.145795596	0.621235225
hsa-miR-4484	0.0569857	0.0662225	0.1555155	0.180722974
hsa-miR-7844-5p	0.0569857	0.0662225	0.1555155	0.180722974
hsa-miR-3679-5p	0.0569857	0.198668	0.1555155	0.542170286
hsa-miR-4523	0.0569857	0.198668	0.1555155	0.542170286
hsa-miR-489-3p	0.170957	0.331113	0.155515833	0.301206233
hsa-miR-610	0.113971	0.198668	0.172794341	0.301205623
hsa-miR-548c-3p	0.07978	0.158934	0.209347972	0.417053279
hsa-miR-676-5p	0.170957	0.463558	0.212066256	0.575027693
hsa-miR-4485-3p	10.7703	26.3566	0.218044778	0.5335895
hsa-miR-570-3p	0.341914	0.596003	0.222164898	0.38726389
hsa-miR-891a-5p	0.455886	0.331113	0.222165584	0.161360325
hsa-miR-1271-5p	7.29417	17.2179	0.230927551	0.545104855
hsa-miR-3917	0.170957	0.397335	0.233273113	0.542168921
hsa-miR-5582-3p	0.455886	0.662225	0.239254975	0.347544399
hsa-miR-4284	2.84929	5.56269	0.241483672	0.471450365
hsa-miR-5091	0.113971	0.132445	0.259191707	0.301205093
hsa-miR-4720-5p	0.113971	0.198668	0.259191707	0.451808777
hsa-miR-144-3p	0.0569857	0.0662225	0.259192028	0.301204408
hsa-miR-3126-5p	0.0569857	0.132445	0.259192028	0.602408817
hsa-miR-3192-3p	0.0569857	0.132445	0.259192028	0.602408817
hsa-miR-4485-5p	0.0569857	0.132445	0.259192028	0.602408817
hsa-miR-1296-3p	0.227943	0.331113	0.25919255	0.376506507
hsa-miR-4521	3.47613	5.82758	0.269500868	0.451806426
hsa-miR-146a-3p	1.65259	2.78135	0.285440643	0.480403689
hsa-miR-7-5p	1.88053	2.45023	0.309157356	0.402815498
hsa-miR-4477b	0.0569857	0.0331113	0.31103015	0.180722753
hsa-miR-6733-5p	0.170957	0.132445	0.333246914	0.258175375
hsa-miR-548ab	0.372501	0.727659	0.335290464	0.654970387
hsa-miR-548 h-3p	0.487736	0.725844	0.337793045	0.502700344
hsa-miR-548z	0.487736	0.725844	0.337793045	0.502700344
hsa-miR-548b-5p	0.250673	0.433284	0.341223938	0.589799749
hsa-miR-200c-3p	0.227943	0.364224	0.345590197	0.552209298
hsa-miR-134-3p	0.7978	0.728448	0.351164459	0.320638065
hsa-miR-7705	10.0295	11.9863	0.372897929	0.445651972
hsa-miR-663a	0.113971	0.0662225	0.388787119	0.225903563
hsa-miR-301b-5p	0.170957	0.26489	0.388788698	0.602410187
hsa-miR-942-3p	0.284929	0.331113	0.388789431	0.451808117
hsa-miR-34b-3p	0.0569857	0.0662225	0.38878981	0.451808667
hsa-miR-6508-5p	0.0569857	0.0662225	0.38878981	0.451808667
hsa-miR-6510-3p	0.0569857	0.0662225	0.38878981	0.451808667
hsa-miR-548aj-5p	0.155689	0.149897	0.390962282	0.376417558
hsa-miR-548 g-5p	0.155689	0.149897	0.390962282	0.376417558
hsa-miR-548x-5p	0.155689	0.149897	0.390962282	0.376417558
hsa-miR-3944-3p	0.683829	0.728448	0.405693589	0.432164596
hsa-miR-153-3p	0.3989	0.0662225	0.418695505	0.069508807

### Small RNA sequencing identifies novel miRNAs co-regulated by c-Myc and Kaposin B in primary endothelial cells

**Fig. 3:**
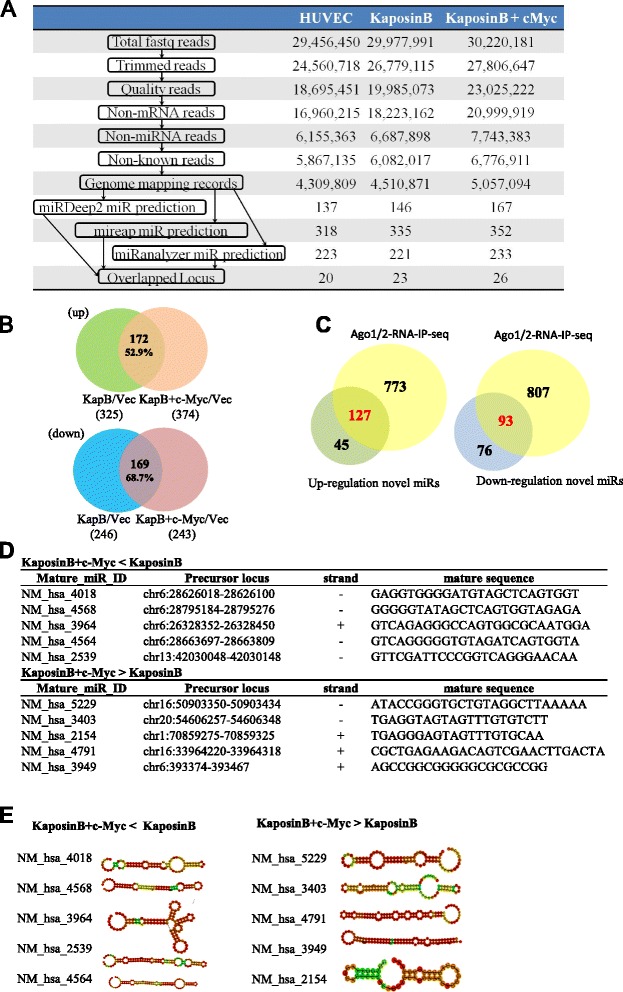
c-Myc cooperates with Kaposin B to regulate cellular novel miRNAs. **a** The analysis pipeline for identification of known and novel miRNAs from smRNA-seq data. Reads or sequences pass each filtration process are indicated. **b** Venn diagrams summarizing significant overlap between c-Myc and Kaposin B signature novel miRNAs. (*upper panel*) up-regulated miRNAs; (*lower panel*) down-regulated miRNAs. **c** Venn diagrams summarizing significant overlap between up-regulated or down-regulated novel miRNA candidates and Ago1/2(+) RNA-IP-seq data. **d** Detailed information of 5 c-Myc-promoting and -reducing novel miRNAs that expressed higher reads in different groups. **e** Deduced RNA secondary structures of a set of newly discovered miRNAs

To identify novel miRNAs, we first removed mRNA contamination and known miRNA loci. Using three independent bioinformatics algorithms (miRDeep2, mireap and miRanalyzer), 20–26 unique genomic loci yielded at the same time in Kaposin B(+)-, Kaposin B(+)c-Myc(+)-expressing cells and HUVEC control cells (Fig. 3a), and we listed all novel miRNA candidates in (Additional file 5). We found 325 and 374 novel miRNA candidates in Kaposin B-expressing cells and Kaposin B(+)c-Myc(+) cells (KapB + c-Myc/vec) that were up-regulated, respectively. In contrast, we identified 246 and 243 novel miRNA candidates in Kaposin B-expressing cells and down-regulated novel miRNA candidates in Kaposin B(+)c-Myc(+) cells (KapB + c-Myc/vec) that were down-regulated, respectively. To characterize c-Myc regulated novel miRNA candidates in Kaposin B cells, we compared the miRNA profiles between Kaposin B(+)c-Myc(+) cells and Kaposin B(+) cells. The miRNA profiles revealed that out of 325 miRNAs, 172 (52.9 %) were up-regulated and out of 246, 169 (68.7 %) were down-regulated novel mature miRNA candidates correlated with c-Myc expression in Kaposin B(+) cells (Fig. 3b). To investigate the RISC-binding properties of the novel miRNA candidates, we acquired nine public Ago1/2-mediated RNA-immunoprecipitation sequencing (Ago1/2-RNA-IP-seq) datasets from the GEO database and re-analyzed using our in-house pipelines [32]. The Ago1/2-RNA-IP-seq datasets verified 127 out of 172 up-regulated and 93 out of 169 down-regulated novel miRNA candidates (Fig. 3c).

To investigate c-Myc-promoting or -reducing novel miRNAs expression in Kaposin B(+) cells, we found that 77 out of 127 were up-regulated and 28 out of 93 were down-regulated novel mature miRNA candidates that were c-Myc-promoting or -reducing novel miRNAs expression in Kaposin B(+) cells (Additional files 6 and 7). We showed 5 up-regulated or down-regulated novel c-Myc-regulating miRNAs candidates (KaposinB + c-Myc > KapsoinB or KaposinB > KaposinB + c-Myc) that expressed higher read numbers in Kaposin B(+) and Kaposin B(+)c-Myc(+) cells (Fig. 3d) and their detailed information, including precursor locus, strand and mature sequence. Based on RNAfold analysis (http://rna.tbi.univie.ac.at/cgi-bin/RNAfold.cgi), we demonstrated that these novel miRNAs could fold into hairpin secondary structure, which is another miRNA property (Fig. 3e).

### c-Myc enhances Kaposin B association with and regulation of miRNA promoters

**Fig. 4:**
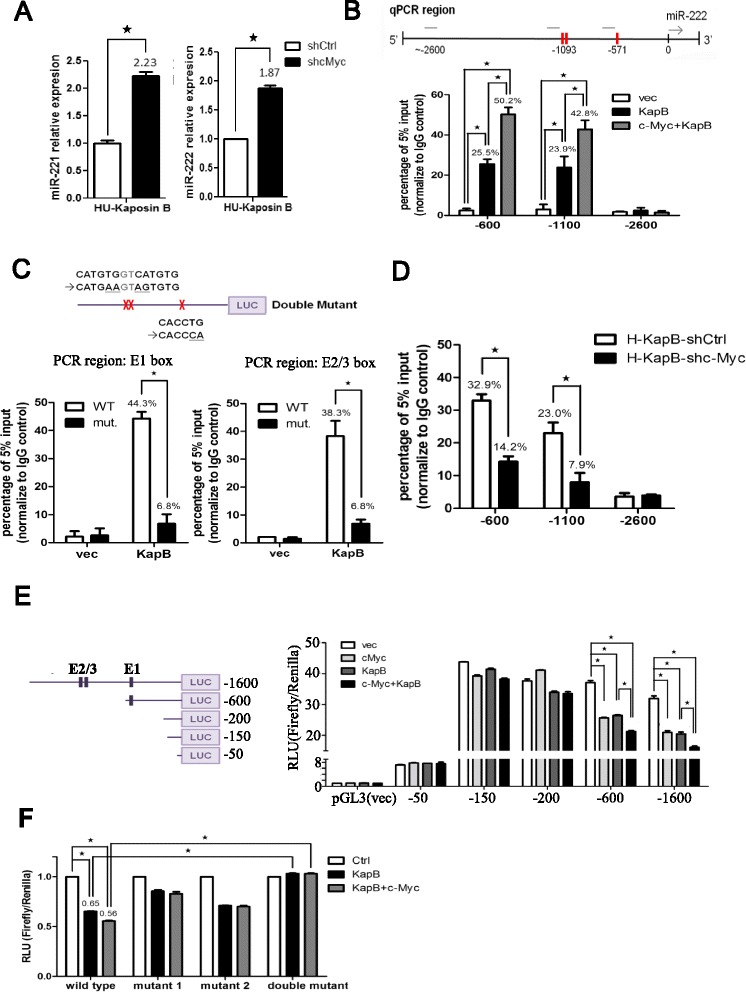
c-Myc enhances Kaposin B to regulate microRNA promoter activity. **a** Knockdown of endogenous c-Myc levels in Kaposin B(+) HUVECs rescues miR-221 (*left*) and miR-222 (*right*) expression. **b** ChIP analysis with immunoglobulin G control or anti-FLAG antibody (detecting Kaposin B). (*upper*) Schematic representation of miR-221/-222 promoter region and PCR fragments. (*lower*) HMEC1 cells stably expressing empty vector, Kaposin B, or Kaposin B + c-Myc were subjected into ChIP assays. The −2600 promoter region PCR product was used as a negative control. **c** Wild type (−1600) promoter construct or double mutant construct (*upper*) was transfected into a HMEC1 cell line stably expressing Kaposin B (HMEC1-Kaposin B), and ChIP-qPCR assays were performed on transfectants using anti-FLAG mAb. **d** Endogenous c-Myc was stably knocked down using shRNA in a HMEC1-Kaposin B stable cell line, and cells with or without c-Myc knockdown were subjected to ChIP assays and qPCR for indicated regions (*n* = 3). **e** Reporter activity of tested reporter plasmids after co-transfection with Kaposin B, c-Myc, or c-Myc + Kaposin B. Schematic representation of reporter constructs (*left*) and reporter assay results (*n* = 3) (*right*). *: *P* < .05. **f** Reporter assays on E1 or E2/3 single mutants, and on the E1 and E2/E3 E-boxes double mutant. Only the double mutant showed significant promoter activity restoration. (*n* = 3) *: *P* < .05

Previous studies showed that miR-221/-222 promoted angiogenesis and metastasis in various cancers, including gliomas, colon cancers and breast cancers [33–37] and that c-Myc reduces miR-222 expression in mouse mammary tumors [31]. Furthermore, miR-221/-222 were crucial in KSHV-mediated endothelial cell motility [4]. Therefore, to investigate whether c-Myc can enhance Kaposin B association with and regulation of miR-221/-222 promoters, we knocked down c-Myc expression and we found that miR-221/-222 level were rescued (Fig. 4a).

Next, we performed chromatin immunoprecipitation (ChIP) assays using an anti-FLAG mAb to pull down Kaposin B and its associated genomic DNA fragments. ChIP data identified the presence of c-Myc-interacting miR-221/-222 promoter fragments containing the E1 (−600) or E2/E3 (−1100) E-boxes in HMEC1 cells stably expressing Kaposin B (Fig. 4b). Overexpressing c-Myc further enhanced the association between Kaposin B and the miR-221/-222 promoter (Fig. 4b). A negative control PCR containing a region with no putative E-box motif (the “−2600” group) showed no off-target enrichment from the same ChIP set (Fig. 4b). Direct involvement of E1 and E2/3 motifs in Kaposin B-c-Myc complex binding was verified by ChIP assays using the site-directed mutagenesis of all the E1 and E2/E3 E-boxes plasmids. A wild type −1600 promoter construct, double mutant construct and Kaposin B (empty pLenti4/V5 vector) were co-transfected into 293T cells, and ChIP assays were performed using anti-FLAG mAb. qPCR on IP-purified DNA fragments showed that these E-box motifs were essential for Kaposin B to associate with miR-221/-222 promoter (Fig. 4c). We next examined the critical role of endogenous c-Myc in Kaposin B function. Knockdown of endogenous c-Myc in Kaposin B(+) HMEC1 cells significantly reduced Kaposin B and miR-221/-222 promoter association (Fig. 4d).

**Fig. 5:**
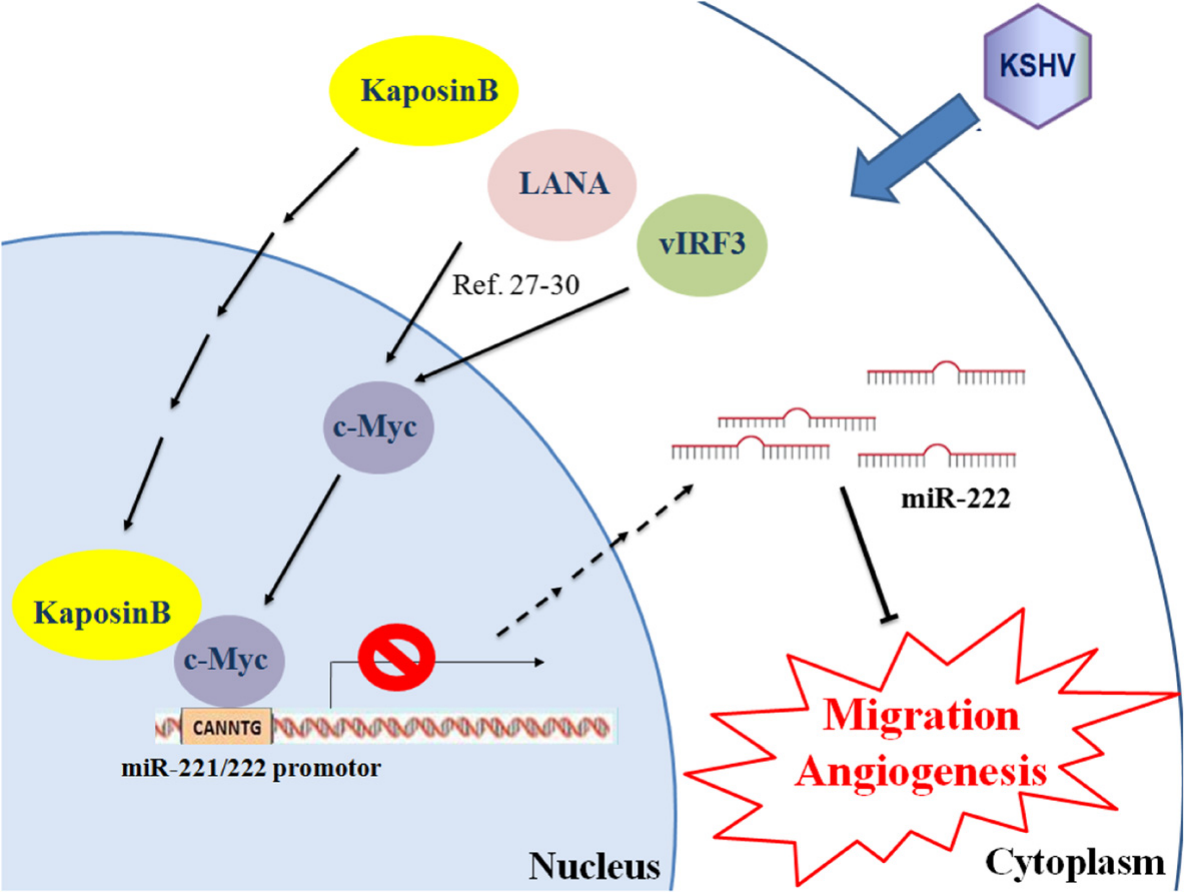
A proposed model of regulation of endothelial cell activities by the Kaposin B-c-Myc circuit

Repression of miR-221/-222 promoter activity was observed after transient co-transfection of Kaposin B- or c-Myc-expressing plasmids with the full-length reporter construct into 293T cells (Fig. 4f, the “−1600 bp” group). Ectopic expression of both Kaposin B and c-Myc repressed miR-221/-222 promoter activity significantly more than expression of Kaposin B or c-Myc alone (Fig. 4e), suggesting c-Myc enhances the capability of Kaposin B to regulate the miR-221/-222 promoter. Deleting the region containing E2/3 E-boxes did not dramatically influence Kaposin B or c-Myc function (Fig. 4e, the “−600” group), but additional deletion of the E1 E-box significantly abolished the repression of the miR-221/-222 promoter by Kaposin B, c-Myc or both Kaposin B and c-Myc together (Fig. 4e, the “−200”, “−150” and “−50” group). Site-directed mutagenesis of all the E1 and E2/E3 E-boxes, but not E1 or E2/3 alone, hampered Kaposin B- and/or c-Myc-mediated repression, indicating c-Myc could bind to all the 3 E-boxes to direct Kaposin B to the miR-221/-222 promoter (Fig. 4f). Figure 5 is an illustration of the mechanism whereby Kaposin B and c-Myc in the same transcription complex can directly regulate the miR-221/-222 cluster promoter activity.

## Discussion

Kaposin B is known to stabilize various AU-rich elements (ARE)-containing cytokine mRNAs, such as IL6, *via* the p38/MK2 pathway [14, 38]. AREs are usually 50 to 150 nucleotides in length and located in the 3′ untranslated region (UTR) of short half-life mRNAs. AREs serve as an mRNA-destabilizing determinant by promoting degradation of mRNAs. AREs are now grouped into three classes based on the number and distribution of the core AUUUA pentamers: cluster I ARE genes, such as cell cycle regulators c-Myc and cyclin D1, contain several dispersed copies of the AUUUA motif within the AU-rich region [39]. Cluster II ARE genes (such as cytokines IL1B, IL6, IL8 and granulocyte/macrophage colony-stimulating factor (GM-CSF)) have at least 2 overlapping UUAUUUA(U/A)(U/A) motifs, and cluster III ARE genes (including c-JUN and p53) are less characterized and do not contain the canonical AUUUA motif [39]. Database search revealed that there is no ARE on miR-221-222 pri-miRNA. Whether Kaposin B collaborates with c-Myc to regulate promoter activities of these ARE(−) genes is an interesting direction to follow and can be answered by global genomics approaches, such as ChIP-Seq.

In this study, we use miR-221/-222 as a model to elucidate how Kaposin B regulates promoter activity. We demonstrate that c-Myc together with Kaposin B regulate the activity of miR-221/-222 cluster promoter. c-Myc is known to maintain KSHV latency [40]. KSHV latency proteins, such as LANA and vIRF3, have been demonstrated to regulate c-Myc. LANA stabilizes and activates c-Myc through inhibition of GSK-3 phosphorylation and c-Myc ubiquitination [27, 28]. LANA protein that lacks a known DNA-binding domain, also represses the expression of miR-221/-222 cluster [4]. Nevertheless, pilot studies showed that the interaction of Kaposin B and c-Myc is independent of LANA (not shown). vIRF3 associated with c-Myc suppressor, MM-1α, to stimulate c-Myc function [29]. A c-Myc-oriented angiogenesis program is therefore activated and plays a crucial role in KSHV-infected cells and even in KS. Since KS is a neoplasm with an extensive neovasculature, the induction of endothelial cell migration and microvascular formation by c-Myc and Kaposin B may eventually benefit KS formation and progression.

MYC protein, including c-Myc, N-Myc and L-Myc, have been shown to be involved in pathogenesis of most cancers, such as burkitt’s lymphoma, breast cancer, prostate cancer, gastrointestinal cancer etc. [41]. c-Myc affects most important oncogenic programs through regulating several genes and miRNAs expression [16, 42, 43]. In c-Myc-induced mouse mammary tumors, 50 and 59 miRNAs show increased and decreased expression, respectively. The expression of miR-20a, −20b, and miR-9 were increased, while that of miR-222 was decreased [16, 31]. In angiogenesis, previous report showed that c-Myc activated miR-17 cluster expression to attenuate clusterin expression, thereby promoting angiogenesis [44]. In this report, we provide a comprehensive c-Myc-regulated miRome trait in primary human endothelial cells. Most of the c-Myc and/or Kaposin B-affected miRNAs have not been linked to angiogenesis, cell motility or tumorigenesis. Among miRNAs induced by both c-Myc and Kaposin B (Table 1), miR-210, the most prominent hypoxia-induced microRNA, modulates cellular response to hypoxia through MNT, a MAX-interacting transcriptional repressor that functions as a c-Myc antagonist [45]. miR-483-5p, a microRNA embedded in the intron of insulin-like growth factor 2 (Igf2), inhibits angiogenesis *via* targeting serum response factor [46]. miR-372 is involved in cell migration, proliferation, apoptosis, and globally affect the regulation circuits centered on MAPK/ERK signaling in colon cancer cells [47]. Knockdown of miR-139 in HUVEC significantly suppressed cell viability [48]. miR-423 promotes cell growth and regulates G(1)/S transition by targeting p21Cip1/Waf1 in hepatocellular carcinoma [49].

## Conclusions

In this study, we provide a global picture of KSHV- and/or Kaposin B-induced microRNAs, especially those co-regulated by c-Myc. We have demonstrated that c-Myc is essential for Kaposin B to stimulate endothelial cell activity and to activate miRNA promoters. In addition to providing a global picture, this study also benefits the development of new therapeutic approaches to treat KSHV-induced tumor progression in future.

## Additional files

Additional file 1:
**List of primers used in the study.** (PDF 72 kb) Additional file 2:
**(A) Primary HUVEC stably transduced with Kaposin B or the vector control by lentivirus.** Immunoblotting of Kaposin B proteins with anti-Flag mAb (bottom panel). β-actin was used as an internal control. (**B**) DNase treatment effects were verified by agarose gel electrophoresis. (**C**-**D**) knockdown of endogenous c-Myc levels with shRNA in Kaposin B(+) HUVEC (**C**) and Kaposin B(+) HMEC1 cells (**D**) were validated by immunoblotting and qRT-PCR. (**E**) knockdown of endogenous c-Myc levels with shRNA in Kaposin B(+) HMEC1 cells repressed Kaposin B-induced cellular migration (*n* = 3). (PDF 311 kb) Additional file 3:
**Validation of** **smRNA-Seq** **data by** **RT-qPCR** **in** **KSHV-infected** **cells.** Mean expression levels of the target miRNAs are compared with the U6 control (*n* = 3). *: *P* < .05. (PDF 76 kb) Additional file 4:
**Genes were targeted by the** **up-regulated** **miRNAs (A) and** **down-regulated** **miRNAs (B).** The genes interactions were analyzed by STRING (http://string-db.org/newstring_cgi/show_input_page.pl?UserId=PZAhu49PCqxs&sessionId=A8hUzxbU17ST). (PDF 8481 kb) Additional file 5:
**Detail information of all novel miRNAs.** (PDF 477 kb) Additional file 6:
**List of Kaposin B and** **c-Myc** **down-regulated** **novel miRNAs which exist in Ago1/2-IP-seq data.** (PDF 79 kb) Additional file 7:
**List of Kaposin B and** **c-Myc** **up-regulated** **novel miRNAs which exist in Ago1/2-IP-seq data.** (PDF 83 kb)
