# Supplementary material for: c-Myc and viral cofactor Kaposin B co-operate to elicit angiogenesis through modulating miRNome traits of endothelial cells
Source: BMC Syst Biol. 2016 Jan 11;10(Suppl 1):1. doi: 10.1186/s12918-015-0242-3 (PMC4895700; doi:10.1186/s12918-015-0242-3)
Supplement: Additional file 1: — List of primers used in the study. (PDF 72 kb) [file 12918_2015_242_MOESM1_ESM.pdf]

Additional file 1

qRT-PCR primer_Gene

GAPDH	forward	5’ GGAGTCCACTGGCGTCTTCA 3’
	reverse	5’ TGGTTCACACCCATGACGAA 3’
c-Myc	forward	5’ GCCGACCAGCTGGAGATGGT 3’
	reverse	5’ TGAAGGTCTCGTCGTCCGGG 3’
miR-221/222 promoter E1	forward	5’CAAGCTTTCCTCCCATGATAC 3’
	reverse	5’TTCTGGGGTGGATAAATGAATAG 3’
miR-221/222 promoter E2/E3	forward	5’CATGGCCAATTATTGTTTCCTAC 3’
	reverse	5’CTTTTCTAAGTGATAGCTGAAGC 3’
miR-221/222 promoter NC	forward	5’TTCGTGCGTCATTCTACTGA 3’
	reverse	5’TCAGAGATGCTTTTTGGGGG 3’

qRT-PCR primer_miRNA

miR-221	forward	5’ AGCCGAGCTACATTGTCTGCTG 3’
	reverse	5’GTGCAGGGTCCGAGGT 3’
miR-222	forward	5’ ATGCCGAGCTACATCTGGCTA 3’
	reverse	5’GTGCAGGGTCCGAGGT 3’
miR-100	forward	5’ GGCGAAACCCGTAGATCCGAA 3’
	reverse	5’GTGCAGGGTCCGAGGT 3’
miR-146a-3p	forward	5’ CGCGGCCTCTGAAATTCAGTT 3’
	reverse	5’GTGCAGGGTCCGAGGT 3’
miR-193b-5p	forward	5’ CCGGGGTTTTGAGGGCG 3’
	reverse	5’GTGCAGGGTCCGAGGT 3’
miR-197-5p	forward	5’ CACGGGTAGAGAGGGCAGT 3’
	reverse	5’GTGCAGGGTCCGAGGT 3’
miR-210	forward	5’ATCTGTGCGTGTGACAGC 3’
	reverse	5’GTGCAGGGTCCGAGGT 3’
miR-1246	forward	5’ CGCCGAATGGATTTTTGG 3’
	reverse	5’GTGCAGGGTCCGAGGT 3’
miR-1271-5p	forward	5’CGCGTGATTGGTACGTCTGT 3’
	reverse	5’GTGCAGGGTCCGAGGT 3’
U6	forward	5’-CTCGCTTCGGCAGCAC-3’
	reverse	5’-AACGCTTCACGAATTTGCG-3’

cloning

miR-222	forward	5’CAAGGAATCATGTATGCTG 3’
	reverse	5’GTGTGTGTAATTCAAGGTAAAG 3’
E-box E1 mutant	forward	5' GCATATTATAATAAATATTCAAAGTGAGTGCTCACTAGCATGTCAG 3’
	reverse	5'CTGACATGCTAGTGAGCACTCACTTTGAATATTTATTATAATATGC 3’
E-box E2/E3 mutant	forward	5’GGTGTTCTTTTATTAGCACACTACTTCATGGCCAATTATTGTTTCC 3’
	reverse	5’GGAAACAATAATTGGCCATGAAGTAGTGTGCTAATAAAAGAACACC 3’
