# Supplementary figures and images for: c-Myc and viral cofactor Kaposin B co-operate to elicit angiogenesis through modulating miRNome traits of endothelial cells
Source: BMC Syst Biol. 2016 Jan 11;10(Suppl 1):1. doi: 10.1186/s12918-015-0242-3 (PMC4895700; doi:10.1186/s12918-015-0242-3)

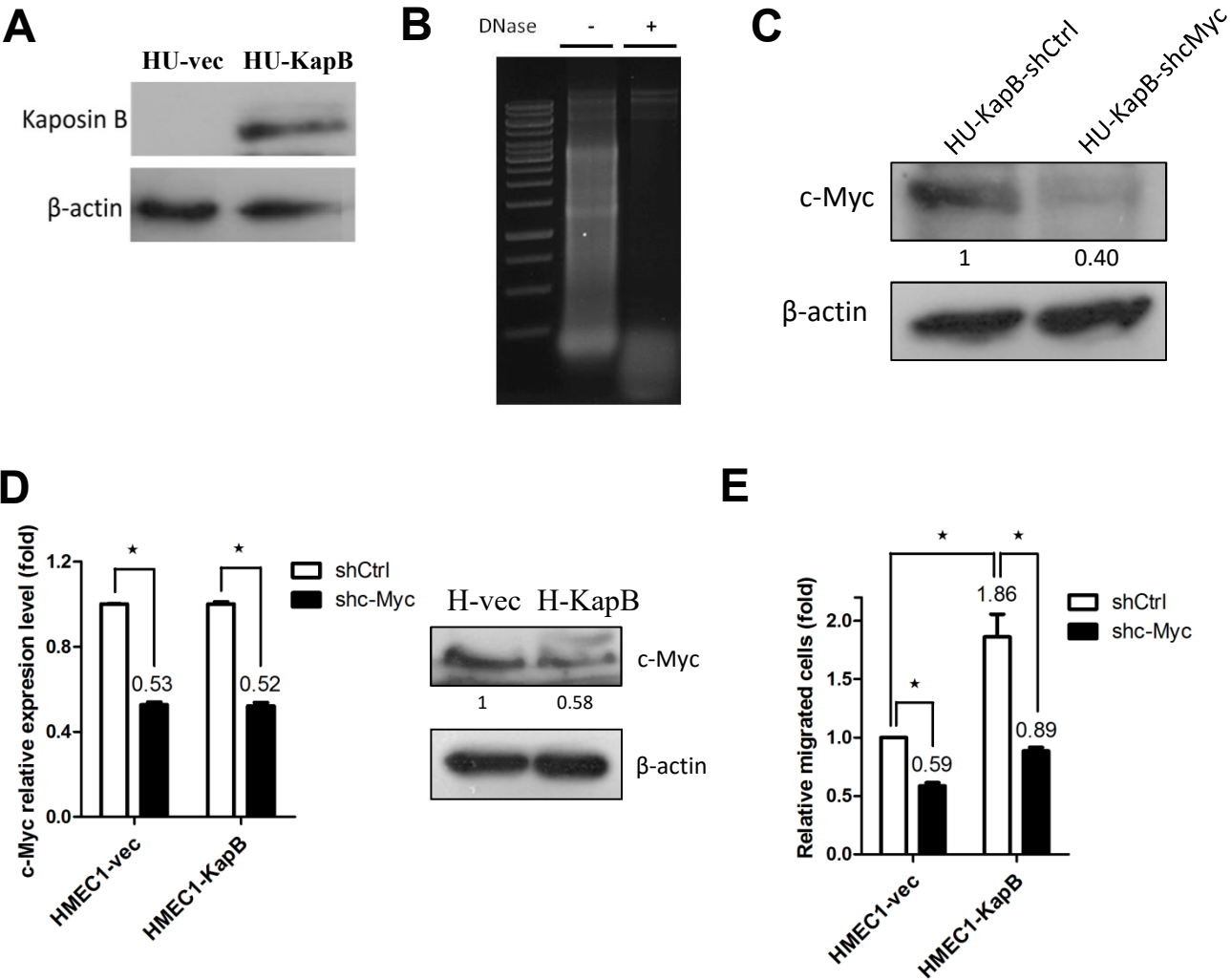

Supplement: Additional file 2: — (A) Primary HUVEC stably transduced with Kaposin B or the vector control by lentivirus. Immunoblotting of Kaposin B proteins with anti-Flag mAb (bottom panel). β-actin was used as an internal control. (B) DNase treatment effects were verified by agarose gel electrophoresis. (C-D) knockdown of endogenous c-Myc levels with shRNA in Kaposin B(+) HUVEC (C) and Kaposin B(+) HMEC1 cells (D) were validated by immunoblotting and qRT-PCR. (E) knockdown of endogenous c-Myc levels with shRNA in Kaposin B(+) HMEC1 cells repressed Kaposin B-induced cellular migration (n = 3). (PDF 311 kb) [file 12918_2015_242_MOESM2_ESM.pdf]

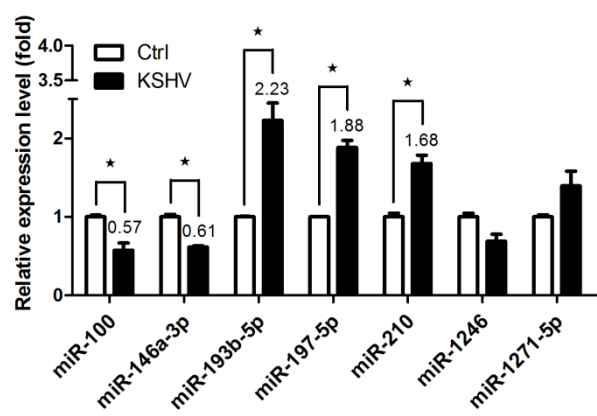

Supplement: Additional file 3: — Validation of smRNA-Seq data by RT-qPCR in KSHV-infected cells. Mean expression levels of the target miRNAs are compared with the U6 control (n = 3). *: P < .05. (PDF 76 kb) [file 12918_2015_242_MOESM3_ESM.pdf]

A

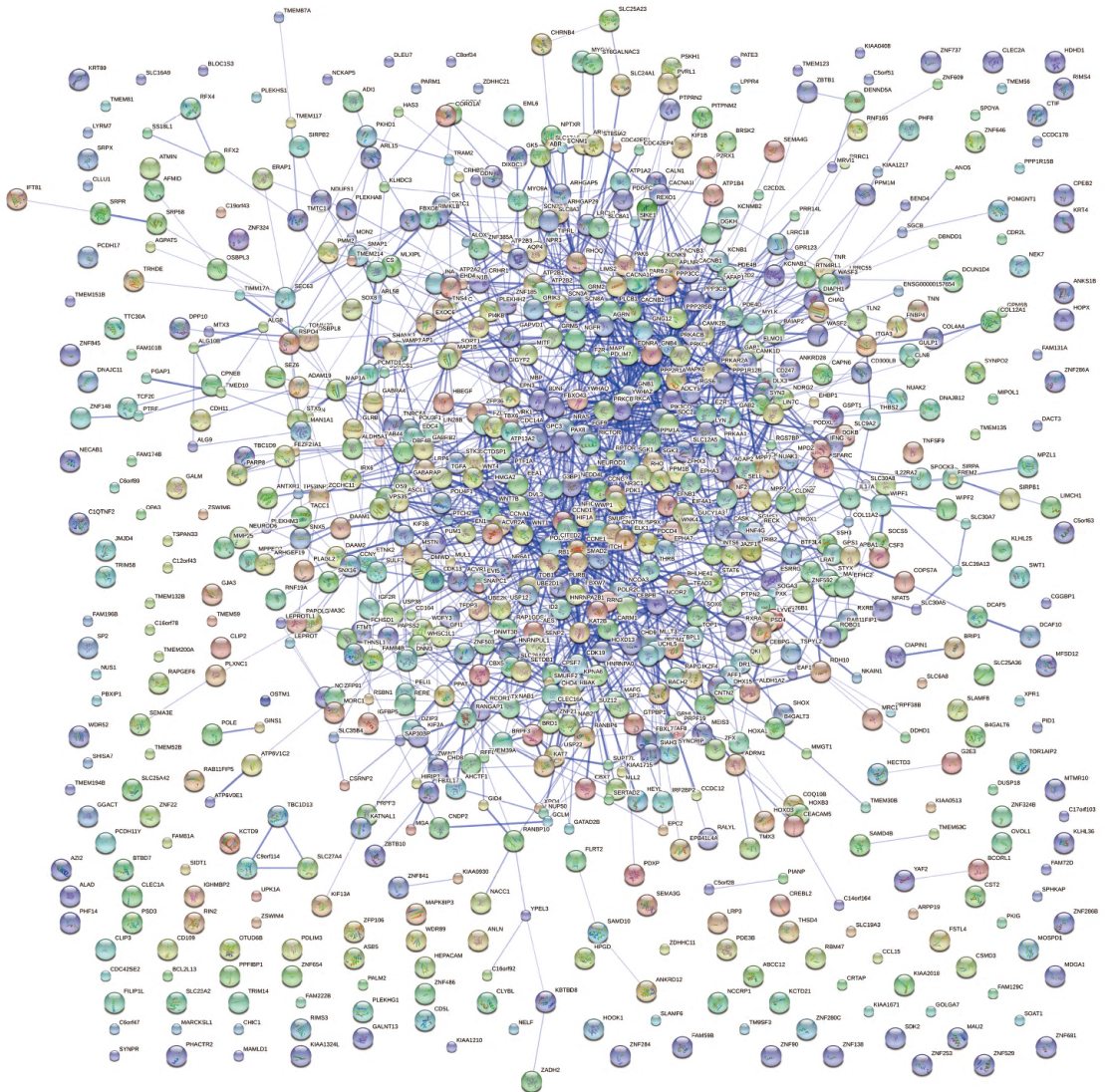

B

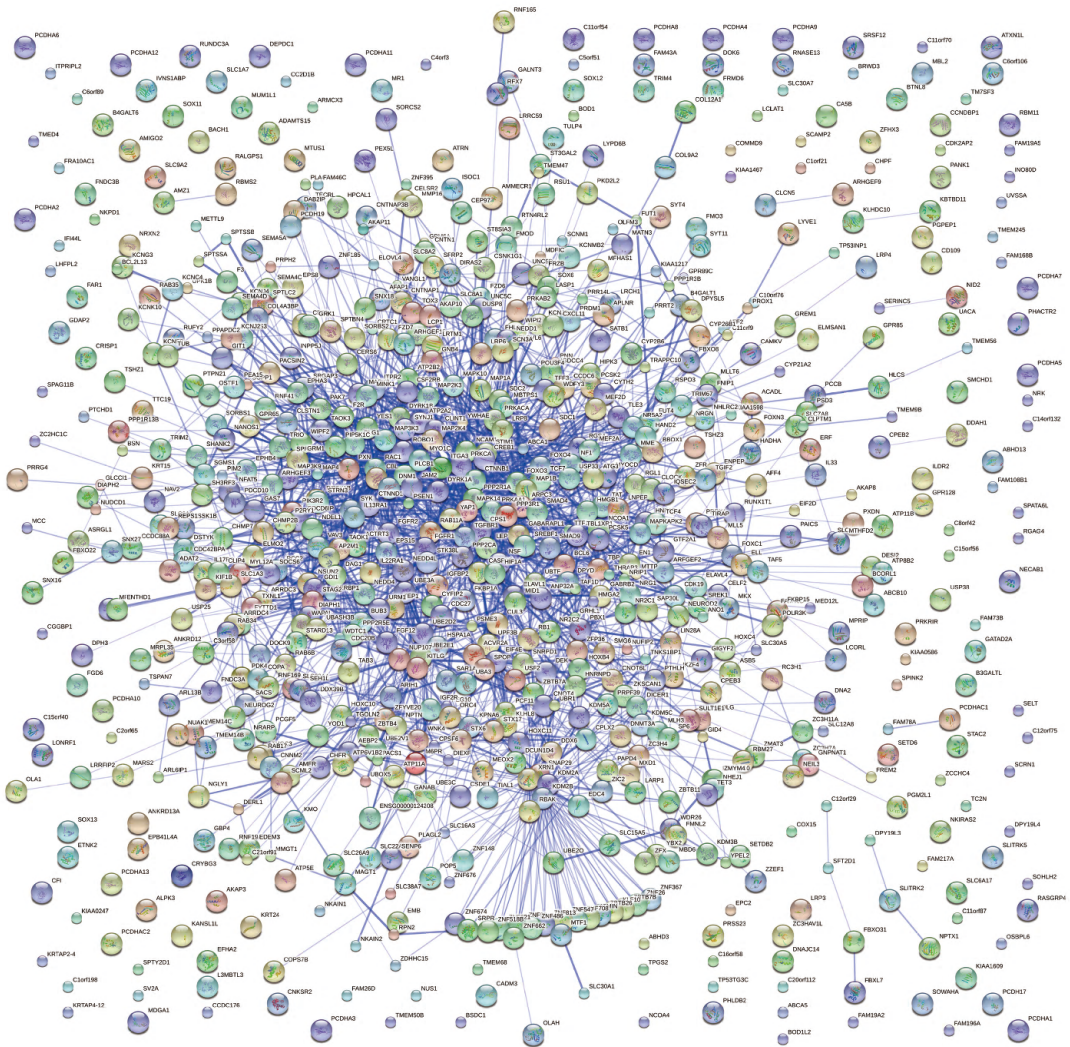

Supplement: Additional file 4: — Genes were targeted by the up-regulated miRNAs (A) and down-regulated miRNAs (B). The genes interactions were analyzed by STRING (http://string-db.org/newstring_cgi/show_input_page.pl?UserId=PZAhu49PCqxs&sessionId=A8hUzxbU17ST). (PDF 8481 kb) [file 12918_2015_242_MOESM4_ESM.pdf]
