# Supplementary material for: c-Myc and viral cofactor Kaposin B co-operate to elicit angiogenesis through modulating miRNome traits of endothelial cells
Source: BMC Syst Biol. 2016 Jan 11;10(Suppl 1):1. doi: 10.1186/s12918-015-0242-3 (PMC4895700; doi:10.1186/s12918-015-0242-3)
Supplement: Additional file 5: — Detail information of all novel miRNAs. (PDF 477 kb) [file 12918_2015_242_MOESM5_ESM.pdf]

Additional file 5

Name	locus	strand	sequence	mirdeep2	mireap	miranalyzer	RPM_HU_KapB_cMyc	RPM_HU_KaposinB	RPM_HUVEC	IP_confidence	annotation
NM_hsa_1	chrM:15955-16024	-	GTTAAAGACTTTTTCTCTGAT	Yes	No	No	0.113971	0.26489	1.24587	Yes	H>KapB>KapB+cMyc
NM_hsa_1000	chr17:58755240-58755300	+	GTGAGAGGGCTGGCAGGGCTTTGG	Yes	No	No	0.113971	0	0	No	
NM_hsa_1002	chr11:624936-625006	+	CCCAGCCAGACGCCGCCCAGT	Yes	Yes	No	0	0	0	No	
NM_hsa_1002	chr11:624925-625015	+	CCCAGCCAGACGCCGCCCAGT	Yes	Yes	No	0	0	0	No	
NM_hsa_1003	chr11:624936-625006	+	CGGGTCTGGCGTCTAGGACTGGCGC	Yes	No	No	0	0	0	No	
NM_hsa_1004	chr16:2618941-2618992	+	TGGGCTGTGCTCTGGGCATT	Yes	Yes	No	0	0	0	No	
NM_hsa_1004	chr16:2681202-2681253	-	TGGGCTGTGCTCTGGGCATT	Yes	Yes	No	0	0	0	No	
NM_hsa_1004	chr16:2681193-2681263	-	TGGGCTGTGCTCTGGGCATT	Yes	Yes	No	0	0	0	No	
NM_hsa_1005	chr16:2618941-2618992	+	TGCCCACAGCCAGCCTCAGG	Yes	No	No	0	0	0	No	
NM_hsa_1005	chr16:2681202-2681253	-	TGCCCACAGCCAGCCTCAGG	Yes	No	No	0	0	0	No	
NM_hsa_1008	chrX:129299909-129299961	+	CCGGTTTTCGCGAGTTTGTGG	Yes	Yes	Yes	0	0.0662225	0	No	
NM_hsa_1008	chrX:129299898-129299970	+	CCGGTTTTCGCGAGTTTGTGG	Yes	Yes	Yes	0	0.0662225	0	No	
NM_hsa_1008	chrX:129299879-129299985	+	CCGGTTTTCGCGAGTTTGTGG	Yes	Yes	Yes	0	0.0662225	0	No	
NM_hsa_1009	chrX:129299909-129299961	+	GCAAGCTCGCAAAGACGCTGGCT	Yes	No	No	0	0	0	No	
NM_hsa_101	chr10:135055629-135055715	+	TGGGGCGGGCTCCAAGTG	Yes	No	No	0	0	0	No	
NM_hsa_1010	chr4:9776962-9777035	-	CTCTGATGCTGCATGGGGGC	Yes	No	No	0	0	0	No	
NM_hsa_1011	chr4:9776962-9777035	-	CCCCTTTCTTGCTGCTTCTGGGCT	Yes	No	No	0	0	0	Yes	
NM_hsa_1014	chr1:147829303-147829365	+	TCAGGCACAGACAATGAGTGTGC	Yes	No	No	0	0	0	No	
NM_hsa_1015	chr20:60941181-60941248	+	CCTCACCCAAGGCTGACCCCAGGC	Yes	No	No	0	0	0	No	
NM_hsa_1016	chr2:74881211-74881264	-	AGTTCTGCCTCGGAAGTG	Yes	No	No	0	0	0	No	
NM_hsa_1017	chr2:74881211-74881264	-	AGGCAGAGGCGGAGCTAG	Yes	No	No	0	0	0	No	
NM_hsa_1018	chr11:117049437-117049480	+	GTTCGTGCGTCGGGACGG	Yes	No	No	0	0	0	No	
NM_hsa_1019	chr11:117049437-117049480	+	CCAGCGGCGCAGCTCAC	Yes	No	No	0	0	0	No	
NM_hsa_1020	chr3:43757997-43758072	+	AAAAAGATTGAGGCCATGGTAG	Yes	Yes	Yes	0	0	0	No	
NM_hsa_1020	chr3:43757967-43758105	+	AAAAAGATTGAGGCCATGGTAG	Yes	Yes	Yes	0	0	0	No	
NM_hsa_1020	chr3:43758007-43758064	+	AAAAAGATTGAGGCCATGGTAG	Yes	Yes	Yes	0	0	0	No	
NM_hsa_1021	chr3:43758007-43758064	+	GTAGCCTCAGTCTTTTTAA	Yes	No	No	0	0	0	No	
NM_hsa_1022	chr14:106354391-106354459	+	TGGGGTTGTGTCACTGTGGGAA	Yes	Yes	Yes	0	0	0	No	
NM_hsa_1022	chr14:106354381-106354479	+	TGGGGTTGTGTCACTGTGGGAA	Yes	Yes	Yes	0	0	0	No	
NM_hsa_1022	chr14:106354356-106354484	+	TGGGGTTGTGTCACTGTGGGAA	Yes	Yes	Yes	0	0	0	No	
NM_hsa_1023	chr14:106354391-106354459	+	CTCACAGTGACACGAACCCCCACA	Yes	No	No	0	0	0	No	
NM_hsa_1024	chr12:122905573-122905640	+	TATTCTGACTTCAGGTGCTTAG	Yes	No	No	0	0	0	No	
NM_hsa_1025	chr12:122905573-122905640	+	AAGCACCTGAAGTCAGAATACC	Yes	No	No	0	0	0	No	
NM_hsa_1026	chr9:33436453-33436553	+	CTGCCCTCTGGTTTCCTAGCTGT	Yes	Yes	Yes	0	0	0	No	
NM_hsa_1026	chr9:33436473-33436538	+	CTGCCCTCTGGTTTCCTAGCTGT	Yes	Yes	Yes	0	0	0	No	
NM_hsa_1026	chr9:33436463-33436548	+	CTGCCCTCTGGTTTCCTAGCTGT	Yes	Yes	Yes	0	0	0	No	
NM_hsa_1027	chr9:33436473-33436538	+	AGCTGGAAGCCTGAGGGCAAGA	Yes	No	No	0	0	0	No	
NM_hsa_1029	chr19:57019183-57019264	+	CTCGTCCGACCCCTGGA	Yes	No	No	0	0	0	No	
NM_hsa_1032	chr16:84004010-84004148	+	CCTCCCATAGCTGTGCTTCAGT	Yes	Yes	Yes	0	0	0	Yes	
NM_hsa_1032	chr16:84004035-84004118	+	CCTCCCATAGCTGTGCTTCAGT	Yes	Yes	Yes	0	0	0	Yes	
NM_hsa_1032	chr16:84004049-84004109	+	CCTCCCATAGCTGTGCTTCAGT	Yes	Yes	Yes	0	0	0	Yes	
NM_hsa_1033	chr16:84004049-84004109	+	TGAGGTTCAGCCAGGAGGAGGGG	Yes	No	No	0	0	0	Yes	
NM_hsa_1034	chr8:56821959-56822008	-	ATCTGTGGGATTATGACTGA	Yes	No	No	2.2082	1.22512	1.81383	Yes	
NM_hsa_1035	chr8:56821959-56822008	-	AGTCAGAATCCCACTCAGGT	Yes	No	No	0	0	0	No	
NM_hsa_1036	chr8:22033947-22034012	+	TAGGGGGTGGGACTGCCTGGA	Yes	Yes	No	0	0	0	Yes	
NM_hsa_1036	chr8:22033928-22034021	+	TAGGGGGTGGGACTGCCTGGA	Yes	Yes	No	0	0	0	Yes	
NM_hsa_1037	chr8:22033947-22034012	+	CAGGCAGTCTGCCCTCAGAA	Yes	No	No	0	0	0	No	
NM_hsa_1038	chr10:102798328-102798408	+	CATTGTCTTTCTGTCTCTCCACA	Yes	Yes	Yes	0.170957	0	0.219859	Yes	
NM_hsa_1038	chr10:102798318-102798418	+	CATTGTCTTTCTGTCTCTCCACA	Yes	Yes	Yes	0.170957	0	0.219859	Yes	
NM_hsa_1038	chr10:102798341-102798399	+	CATTGTCTTTCTGTCTCTCCACA	Yes	Yes	Yes	0.170957	0	0.219859	Yes	
NM_hsa_1039	chr10:102798341-102798399	+	TGGGGTGACAGTGAGGCAGGGG	Yes	No	No	0	0	0	No	
NM_hsa_104	chr22:31025635-31025717	+	AACCCAGGTGTCGGAGAGTGC	Yes	Yes	No	0	0	0	No	
NM_hsa_1040	chr1:120114220-120114275	-	GACTGGGTTGACTCTGGAGA	Yes	No	No	0	0	0	No	
NM_hsa_1041	chr1:120114220-120114275	-	GCCAGAATGTGGGCCCAGGCTA	Yes	No	No	0	0	0	No	
NM_hsa_1042	chr1:155197297-155197340	-	GCGGCACGGGTGGTGGGG	Yes	No	No	0	0	0	No	
NM_hsa_1043	chr1:155197297-155197340	-	CCGCCTCCCCAGGGCTGCTT	Yes	No	No	0	0	0	No	
NM_hsa_1046	chr19:4816222-4816293	+	TCCCCAGGTGGTCAGGCAAGGA	Yes	Yes	No	0	0	0	No	
NM_hsa_1046	chr19:4816212-4816300	+	TCCCCAGGTGGTCAGGCAAGGA	Yes	Yes	No	0	0	0	No	
NM_hsa_1047	chr19:4816222-4816293	+	CTTGTCCTCGGGCGCCTGGGACCC	Yes	No	No	0	0	0	No	
NM_hsa_1048	chr13:49888996-49889062	+	TTCAAATGCATGGAGTAGAAC	Yes	Yes	No	0	0	0	No	
NM_hsa_1048	chr13:49888983-49889071	+	TTCAAATGCATGGAGTAGAAC	Yes	Yes	No	0	0	0	No	
NM_hsa_1049	chr13:49888996-49889062	+	TCTACTCCATGCATTCAAATG	Yes	No	No	0	0	0	No	
NM_hsa_1050	chrX:2846908-2846953	+	ACCCAGCAGGGCGTGACCGG	Yes	No	No	0	0	0	No	
NM_hsa_1051	chrX:2846908-2846953	+	TTCCGCTCCCTCCGGGTCC	Yes	No	No	0	0	0	No	
NM_hsa_1052	chr8:15403349-15403408	+	TTATGCCTGTGACTGCTGTAG	Yes	Yes	No	0	0	0	No	
NM_hsa_1052	chr8:15403339-15403415	+	TTATGCCTGTGACTGCTGTAG	Yes	Yes	No	0	0	0	No	
NM_hsa_1053	chr8:15403349-15403408	+	TCAGCGTTTATTTGGCATGTCA	Yes	No	No	0	0	0	No	
NM_hsa_1054	chr16:57770361-57770435	+	TAGCTCTGTCCTTGGGAATCGC	Yes	Yes	No	0.740814	0.397335	0.146572	Yes	KapB+cMyc>KapB>H
NM_hsa_1054	chr16:57770371-57770428	+	TAGCTCTGTCCTTGGGAATCGC	Yes	Yes	No	0.740814	0.397335	0.146572	Yes	KapB+cMyc>KapB>H
NM_hsa_1055	chr16:57770371-57770428	+	GGTTCCCTTGCCAGGAGCTGGG	Yes	No	No	0	0	0	No	
NM_hsa_1056	chr17:27919433-27919569	+	CTTCCCTTCCTGAAGGGCCAG	Yes	Yes	Yes	0	0	0	Yes	
NM_hsa_1056	chr17:27919472-27919530	+	CTTCCCTTCCTGAAGGGCCAG	Yes	Yes	Yes	0	0	0	Yes	
NM_hsa_1056	chr17:27919460-27919539	+	CTTCCCTTCCTGAAGGGCCAG	Yes	Yes	Yes	0	0	0	Yes	
NM_hsa_1057	chr17:27919472-27919530	+	GGTCATTCAGGAAGGAGACAGTC	Yes	No	No	0	0	0	No	
NM_hsa_1058	chr8:72756808-72756880	+	CTCTGAAGCCCGACGGCCCTT	Yes	Yes	No	0	0	0	No	
NM_hsa_1058	chr8:72756820-72756871	+	CTCTGAAGCCCGACGGCCCTT	Yes	Yes	No	0	0	0	No	
NM_hsa_1059	chr8:72756820-72756871	+	GGGGACGTCTCTGCAGAGCC	Yes	No	No	0	0	0	No	
NM_hsa_106	chr17:16353818-16353875	+	TGTCACTCTTGTGTGTTGCAG	Yes	Yes	No	0	0	0	Yes	
NM_hsa_106	chr17:16353804-16353884	+	TGTCACTCTTGTGTGTTGCAG	Yes	Yes	No	0	0	0	Yes	
NM_hsa_1060	chr11:118661975-118662029	+	CGAAACCTCGGCCGCCGCGG	Yes	No	No	0	0	0	No	
NM_hsa_1061	chr11:118661975-118662029	+	GCGGCGTCACTGCCTTCCTGGCC	Yes	No	No	0	0	0	No	
NM_hsa_1062	chr19:13278696-13278743	+	CTGCCTGGTCTGTGCGGGCTG	Yes	No	No	0	0	0	No	
NM_hsa_1063	chr19:13278696-13278743	+	TCAGACTCAGGATGAGGTGG	Yes	No	No	0	0	0	No	
NM_hsa_1066	chr7:99691401-99691463	+	CTGCACGAACAGCACTTTGGA	Yes	Yes	No	0.113971	0	0	No	
NM_hsa_1066	chr7:99691388-99691472	+	CTGCACGAACAGCACTTTGGA	Yes	Yes	No	0.113971	0	0	No	
NM_hsa_1067	chr7:99691401-99691463	+	GGGAAGTGCTAGCTCAGCAGTA	Yes	No	No	0	0	0	No	
NM_hsa_107	chr17:16353818-16353875	+	GTATCACACCTGTGTGTTACA	Yes	No	No	0	0	0	No	
NM_hsa_1070	chr19:4357963-4358058	+	CCATGGGCTGCGGGCTGGTGA	Yes	Yes	No	0	0	0	No	
NM_hsa_1070	chr19:4358028-4358079	+	CCATGGGCTGCGGGCTGGTGA	Yes	Yes	No	0	0	0	No	
NM_hsa_1071	chr19:4358028-4358079	+	ACTGGTCTGTGTCCCTGCCA	Yes	No	No	0	0	0	No	
NM_hsa_1072	chr2:21728908-21728962	+	TTGAAAAGTTCACGATTGAG	Yes	Yes	No	0	0	0	No	
NM_hsa_1072	chr2:21728895-21728971	+	TTGAAAAGTTCACGATTGAG	Yes	Yes	No	0	0	0	No	
NM_hsa_1073	chr2:21728908-21728962	+	CAATCTTGAACTTTTCAGCT	Yes	No	No	0.0569857	0	0	No	
NM_hsa_1074	chr3:123811949-123812008	+	TCCCTCTCCCTCCTTGCTCCTA	Yes	Yes	No	0	0.26489	0.146572	No	
NM_hsa_1074	chr3:123811937-123812017	+	TCCCTCTCCCTCCTTGCTCCTA	Yes	Yes	No	0	0.26489	0.146572	No	
NM_hsa_1075	chr3:123811949-123812008	+	GGGGCAGGCGGTGGAGGTTGT	Yes	No	No	0	0	0	No	
NM_hsa_1076	chr1:37452826-37452886	-	CTGCCAGGGGAGCTCTGCTG	Yes	Yes	Yes	0	0	0	No	
NM_hsa_1076	chr1:37452778-37452856	-	CTGCCAGGGGAGCTCTGCTG	Yes	Yes	Yes	0	0	0	No	
NM_hsa_1076	chr1:37452787-37452921	-	CTGCCAGGGGAGCTCTGCTG	Yes	Yes	Yes	0	0	0	No	
NM_hsa_1077	chr1:37452826-37452886	-	GGGGAGCTCTGCTGAGCAGAC	Yes	No	No	0	0	0	No	
NM_hsa_1078	chr17:43394699-43394778	+	ACATGCGCCCTCGGCTTCTGG	Yes	Yes	No	0	0	0	Yes	
NM_hsa_1078	chr17:43394712-43394769	+	ACATGCGCCCTCGGCTTCTGG	Yes	Yes	No	0	0	0	Yes	
NM_hsa_1079	chr17:43394712-43394769	+	AGAAGGCGGAGGCGACTAGGGTGT	Yes	No	No	0	0	0	No	
NM_hsa_108	chr17:6920161-6920230	+	TAGCTTATGCCCCCTTCCCAGA	Yes	No	No	0	0	0	Yes	
NM_hsa_1082	chr14:69281839-69281949	+	TTGGTTGGACCTTGGGGCTGGAC	Yes	Yes	Yes	0	0	0.0732862	No	
NM_hsa_1082	chr14:69281892-69281984	+	TTGGTTGGACCTTGGGGCTGGAC	Yes	Yes	Yes	0	0	0.0732862	No	
NM_hsa_1082	chr14:69281902-69281977	+	TTGGTTGGACCTTGGGGCTGGAC	Yes	Yes	Yes	0	0	0.0732862	No	
NM_hsa_1083	chr14:69281902-69281977	+	CCTGACCCTGAGGCCACGCAGAGG	Yes	No	No	0	0	0	No	
NM_hsa_1084	chr7:140772396-140772459	+	CCGCGCCGGACGAGAGAGCG	Yes	No	No	0	0	0	No	
NM_hsa_1085	chr7:140772396-140772459	+	CTCGGTGTTCTCGTCTGTGGGT	Yes	No	No	0	0	0	No	
NM_hsa_1086	chr1:162531046-162531121	-	CCAGGACCCCTGACGT	Yes	No	No	0	0	0	No	
NM_hsa_1087	chr5:65250023-65250083	+	TCTCACCCCCAGATTGTTCTCG	Yes	Yes	No	0	0.0662225	0	Yes	
NM_hsa_1087	chr5:65250013-65250090	+	TCTCACCCCCAGATTGTTCTCG	Yes	Yes	No	0	0.0662225	0	Yes	
NM_hsa_1088	chr5:65250023-65250083	+	AAAATAATCTGTGGGGGAGAGG	Yes	No	No	0	0	0	Yes	
NM_hsa_1089	chr6:127588386-127588451	-	GCTGCTCCGCCCCTCGCACC	Yes	No	No	0	0	0	No	
NM_hsa_109	chr17:6920161-6920230	+	TGGGAAGCTTGGGGCTGGGGGAGAGG	Yes	No	No	0	0	0	No	
NM_hsa_1090	chr21:43741396-43741479	+	TCTCCCTTCCTGCCCTGGCCT	Yes	No	No	0.313421	0.0331113	0	Yes	KapB+cMyc>KapB>H
NM_hsa_1091	chr21:43741396-43741479	+	GTGAGGGAAGAGGGAAGGGAAGG	Yes	No	No	0	0	0	No	
NM_hsa_1092	chr16:3075631-3075710	+	TCATCCAGTCTTTCCCTGCAGA	Yes	No	No	0	0	0	Yes	
NM_hsa_1093	chr16:3075631-3075710	+	TTGGGGGAAGCAGGGAGG	Yes	No	No	0	0	0	No	
NM_hsa_1094	chr11:123480430-123480498	+	TGGAGGGGAAACTTGGATTGGG	Yes	Yes	No	0	0	0	No	
NM_hsa_1094	chr11:123480420-123480501	+	TGGAGGGGAAACTTGGATTGGG	Yes	Yes	No	0	0	0	No	
NM_hsa_1095	chr11:123480430-123480498	+	CATTCCAGGGCTCTCCTCTACC	Yes	No	No	0.170957	0	0.0732862	Yes	
NM_hsa_1096	chr1:94703309-94703351	+	CGGCCGGACGCTGTCCCAG	Yes	No	No	0	0	0.0732862	No	
NM_hsa_1096	chr1:94703264-94703328	+	CGGCCGGACGCTGTCCCAG	Yes	No	No	0	0	0.0732862	No	
NM_hsa_1097	chr1:94703264-94703328	+	GGGAGGCAACTAGCTCGCG	Yes	No	No	0	0	0	No	
NM_hsa_1098	chr11:61159942-61159993	-	CTGGACGAGCTCGGGCCTG	Yes	No	No	0	0	0.0732862	No	
NM_hsa_1099	chr11:61159942-61159993	-	CGCTCGGATGTCCCCTC	Yes	No	No	0	0	0	No	
NM_hsa_110	chr2:240079722-240079764	+	CGGACTGCAGGGCTGGGACAC	Yes	No	No	0	0	0	No	
NM_hsa_1102	chr20:34330581-34330636	+	CTGTCAAGGTCGCCATAAGG	Yes	No	No	0	0	0	No	
NM_hsa_1103	chr20:34330581-34330636	+	TTATGGCGATAGGATAAAACGGCC	Yes	No	No	0	0	0	No	
NM_hsa_1104	chr1:26827769-26827833	+	ATCTGGGGGCCAGATCTGGAG	Yes	No	No	0	0	0	No	
NM_hsa_1105	chr1:26827769-26827833	+	CCGGATCAGGTTCTTGGAACC	Yes	No	No	0	0	0	No	
NM_hsa_1106	chr4:129576517-129576588	+	TTCTTTATTCCAGATCCAGGAT	Yes	Yes	No	0	0	0	No	
NM_hsa_1106	chr4:129576499-129576597	+	TTCTTTATTCCAGATCCAGGAT	Yes	Yes	No	0	0	0	No	
NM_hsa_1107	chr4:129576517-129576588	+	CCTGGATCTGGAATGAAGAAGA	Yes	No	No	0	0	0	No	
NM_hsa_1108	chr3:43039463-43039535	+	TCTGCCATAGGTTGAGGGCTGC	Yes	No	No	0	0	0	Yes	
NM_hsa_1109	chr3:43039463-43039535	+	TGTCCTCCCCTACAGGTAAAGA	Yes	No	No	0	0	0	No	
NM_hsa_111	chr2:240079722-240079764	+	ATTCCACCATGCAGTCAGGC	Yes	No	No	0	0	0	No	
NM_hsa_1110	chr2:85892501-85892582	-	CCACCTGATTCTTTCCCCAG	Yes	No	No	0	0	0	No	
NM_hsa_1111	chr2:85892501-85892582	-	GGGAGGAAGAGAGAGGGGAG	Yes	No	No	0	0	0	No	
NM_hsa_1114	chr4:25661123-25661185	-	TCATGAACAATAAACAAGAGT	Yes	No	No	0	0	0	No	
NM_hsa_1115	chr4:25661123-25661185	-	TCTTGTTTATTGTTCATGACA	Yes	No	No	0	0	0	No	
NM_hsa_1116	chr6:28953463-28953508	+	CTCTGGCGGAGGCGGGAGGGT	Yes	No	No	0	0	0	No	
NM_hsa_1117	chr6:28953463-28953508	+	ATTCCTGTCCCGTTTACATT	Yes	No	No	0	0	0	No	
NM_hsa_1118	chr12:111882959-111883018	+	ACTAGGCCTGGAGCGGAACTGA	Yes	No	No	0.0569857	0	0.0732862	No	
NM_hsa_1119	chr12:111882959-111883018	+	CGTTCCCCTCCAGGCCTGGCCT	Yes	No	No	0.113971	0	0	No	
NM_hsa_112	chr10:104418532-104418617	+	CCTGCCCCCATCCACTTCGGC	Yes	Yes	No	0	0	0	No	
NM_hsa_112	chr10:104418545-104418608	+	CCTGCCCCCATCCACTTCGGC	Yes	Yes	No	0	0	0	No	
NM_hsa_1120	chr11:61770616-61770679	+	TTTCTTTCTTCAGCTTTCCTCT	Yes	Yes	Yes	0	0	0	No	
NM_hsa_1120	chr11:61770586-61770704	+	TTTCTTTCTTCAGCTTTCCTCT	Yes	Yes	Yes	0	0	0	No	
NM_hsa_1120	chr11:61770606-61770686	+	TTTCTTTCTTCAGCTTTCCTCT	Yes	Yes	Yes	0	0	0	No	
NM_hsa_1121	chr11:61770616-61770679	+	GGAGATCGCTGGAGAAAGAGGCA	Yes	No	No	0	0	0	No	
NM_hsa_1122	chr17:27074788-27074863	+	TTGGGTCTAGGGGTGGGAACTGG	Yes	No	Yes	0.284929	0	0	Yes	
NM_hsa_1122	chr17:27074758-27074878	+	TTGGGTCTAGGGGTGGGAACTGG	Yes	No	Yes	0.284929	0	0	Yes	
NM_hsa_1123	chr17:27074788-27074863	+	AGTTCCCCCATCCCTACCCCCAGAT	Yes	No	No	0	0.0662225	0	No	
NM_hsa_1124	chr14:106331715-106331801	-	CTGAGCTGAGAACCACTGTGCT	Yes	Yes	No	0	0	0	No	
NM_hsa_1124	chr14:106331738-106331791	-	CTGAGCTGAGAACCACTGTGCT	Yes	Yes	No	0	0	0	No	

Additional file 5

Name	locus	strand	sequence	mirdeep2	mireap	miranalyzer	RPM_HU_KapB_cMyc	RPM_HU_KaposinB	RPM_HUVEC	IP_confidence	annotation
NM_hsa_1125	chr14:106331738-106331791	-	CACAGTGATTGGCAGCTCTACA	Yes	No	No	0	0	0	No	
NM_hsa_1128	chr19:7593165-7593226	+	CAAGCCCCAGATCAGCGCTGCC	Yes	Yes	Yes	0	0	0	Yes	
NM_hsa_1128	chr19:7593140-7593235	+	CAAGCCCCAGATCAGCGCTGCC	Yes	Yes	Yes	0	0	0	Yes	
NM_hsa_1128	chr19:7593127-7593265	+	CAAGCCCCAGATCAGCGCTGCC	Yes	Yes	Yes	0	0	0	Yes	
NM_hsa_1129	chr19:7593165-7593226	+	CAGCACTGACCAGGGGCCCTGGC	Yes	No	No	0	0	0	No	
NM_hsa_113	chr10:104418545-104418608	+	CTTGGGAGGGGTGGAGGTGGGGT	Yes	No	No	0	0	0	No	
NM_hsa_1130	chr1:193126362-193126490	+	CCTGTTCGTAAATGTTAGTGGA	Yes	Yes	Yes	0	0	0.0732862	Yes	
NM_hsa_1130	chr1:193126397-193126457	+	CCTGTTCGTAAATGTTAGTGGA	Yes	Yes	Yes	0	0	0.0732862	Yes	
NM_hsa_1130	chr1:193126387-193126465	+	CCTGTTCGTAAATGTTAGTGGA	Yes	Yes	Yes	0	0	0.0732862	Yes	
NM_hsa_1131	chr1:193126397-193126457	+	CACTAACGTTTATGAACAGTCA	Yes	No	No	0	0	0	No	
NM_hsa_1132	chr17:1547608-1547684	+	TGGCTTTCTCCTGATCTCAGGC	Yes	Yes	No	0.256436	0.430446	0.109929	No	
NM_hsa_1132	chr17:1547618-1547677	+	TGGCTTTCTCCTGATCTCAGGC	Yes	Yes	No	0.256436	0.430446	0.109929	No	
NM_hsa_1133	chr17:1547618-1547677	+	CAGGGTGTCGGGAGAAGCCACC	Yes	No	No	0	0	0.0732862	No	
NM_hsa_1134	chr17:42284724-42284820	-	GTGACTCCACATCCTTTCTCA	Yes	Yes	Yes	0	0	0	No	
NM_hsa_1134	chr17:42284734-42284823	-	GTGACTCCACATCCTTTCTCA	Yes	Yes	Yes	0	0	0	No	
NM_hsa_1134	chr17:42284743-42284799	-	GTGACTCCACATCCTTTCTCA	Yes	Yes	Yes	0	0	0	No	
NM_hsa_1135	chr17:42284743-42284799	-	GGAGGGGAGGGGAGTCCTGCCA	Yes	No	No	0	0	0	No	
NM_hsa_1136	chr19:39869073-39869161	+	CTGGAACATGGCAGAGGGCAT	Yes	Yes	No	0	0	0	No	
NM_hsa_1136	chr19:39869083-39869142	+	CTGGAACATGGCAGAGGGCAT	Yes	Yes	No	0	0	0	No	
NM_hsa_1137	chr19:39869083-39869142	+	GCCCTTTCTCATGTCCCCCC	Yes	No	No	0	0	0	Yes	
NM_hsa_1138	chr3:9991236-9991322	+	GGAGGAACTGTGGCTACCCTGA	Yes	Yes	Yes	0.284929	0	0	No	
NM_hsa_1138	chr3:9991250-9991313	+	GGAGGAACTGTGGCTACCCTGA	Yes	Yes	Yes	0.284929	0	0	No	
NM_hsa_1138	chr3:9991214-9991352	+	GGAGGAACTGTGGCTACCCTGA	Yes	Yes	Yes	0.284929	0	0	No	
NM_hsa_1139	chr3:9991250-9991313	+	TAGGTGGCCACTGTCCCTCTTG	Yes	No	No	0	0	0	No	
NM_hsa_114	chr3:184298127-184298188	+	CCTGAGCCTGCTTGTTGCCTGC	Yes	No	No	0	0	0	No	
NM_hsa_1140	chr3:49752732-49752795	+	TGGCTGTGTGGCTGCTCTGGACC	Yes	Yes	Yes	0.341914	0.79467	0	No	
NM_hsa_1140	chr3:49752722-49752809	+	TGGCTGTGTGGCTGCTCTGGACC	Yes	Yes	Yes	0.341914	0.79467	0	No	
NM_hsa_1140	chr3:49752707-49752817	+	TGGCTGTGTGGCTGCTCTGGACC	Yes	Yes	Yes	0.341914	0.79467	0	No	
NM_hsa_1141	chr3:49752732-49752795	+	TGTCAGGGCTGGACACAGCTGCA	Yes	No	No	0.0569857	0	0	No	
NM_hsa_1142	chr18:48556415-48556457	-	CGGCTCTGAATCTGGACC	Yes	No	No	0	0	0	No	
NM_hsa_1142	chr18:48556362-48556433	-	CGGCTCTGAATCTGGACC	Yes	No	No	0	0	0	No	
NM_hsa_1143	chr18:48556362-48556433	-	TTTGAGGGAGTGGTC	Yes	No	No	0	0	0	No	
NM_hsa_1144	chr10:104139225-104139277	+	CCTGACCCTGCTCTTGCTCTCA	Yes	No	No	0	0.0662225	0	Yes	
NM_hsa_1145	chr10:104139225-104139277	+	GGAGCATCAGGATCAAGAT	Yes	No	No	0	0	0	No	
NM_hsa_1146	chr22:41732160-41732235	+	AGGTTGGAGTGTGGGAACTGTT	Yes	Yes	Yes	0	0	0	Yes	
NM_hsa_1146	chr22:41732170-41732225	+	AGGTTGGAGTGTGGGAACTGTT	Yes	Yes	Yes	0	0	0	Yes	
NM_hsa_1146	chr22:41732135-41732263	+	AGGTTGGAGTGTGGGAACTGTT	Yes	Yes	Yes	0	0	0	Yes	
NM_hsa_1147	chr22:41732170-41732225	+	CAGCTCTCCCAGCCCAACTTCC	Yes	No	No	0	0	0	No	
NM_hsa_1148	chr6:120216549-120216622	+	TTCTGAAGATACAGGCAATAGC	Yes	Yes	Yes	0	0	0	No	
NM_hsa_1148	chr6:120216562-120216613	+	TTCTGAAGATACAGGCAATAGC	Yes	Yes	Yes	0	0	0	No	
NM_hsa_1148	chr6:120216534-120216632	+	TTCTGAAGATACAGGCAATAGC	Yes	Yes	Yes	0	0	0	No	
NM_hsa_1149	chr6:120216562-120216613	+	TATTGCCTCTATCCTCAGAAGT	Yes	No	No	0	0	0	No	
NM_hsa_115	chr3:184298127-184298188	+	AGGTGGGCAGGCCATGGGCTGATGGGA	Yes	No	No	0	0	0	No	
NM_hsa_1152	chr2:29352294-29352352	-	AGACACAGAGATCTTATAGCTA	Yes	No	No	0	0	0	No	
NM_hsa_1153	chr11:35887555-35887683	+	TCAATGACAGCCCTTGGATTGT	Yes	Yes	Yes	0	0	0	No	
NM_hsa_1153	chr11:35887591-35887649	+	TCAATGACAGCCCTTGGATTGT	Yes	Yes	Yes	0	0	0	No	
NM_hsa_1153	chr11:35887578-35887658	+	TCAATGACAGCCCTTGGATTGT	Yes	Yes	Yes	0	0	0	No	
NM_hsa_1154	chr11:35887591-35887649	+	AGTTCCAGGGAGCCATCATTGATA	Yes	No	No	0	0	0	No	
NM_hsa_1155	chr20:2094889-2094973	+	AAAGAAGTACTTTTGAGGCCAG	Yes	No	No	0.113971	0	0	No	
NM_hsa_1156	chr20:2094889-2094973	+	GGCCTCAAAAGTACTTCTTTTG	Yes	No	No	0	0	0	No	
NM_hsa_1157	chr14:80794850-80794921	+	TCCTGTGTAGAATTCTCTCAGC	Yes	Yes	No	0	0	0	No	
NM_hsa_1157	chr14:80794840-80794929	+	TCCTGTGTAGAATTCTCTCAGC	Yes	Yes	No	0	0	0	No	
NM_hsa_1158	chr14:80794850-80794921	+	TCAGAGACTTCTACACGGATA	Yes	No	No	0	0	0	No	
NM_hsa_1159	chr19:49957932-49957988	+	TGGATCAGAACCCTGGCACAGC	Yes	Yes	No	0	0.0662225	0	Yes	
NM_hsa_1159	chr19:49957925-49957997	+	TGGATCAGAACCCTGGCACAGC	Yes	Yes	No	0	0.0662225	0	Yes	
NM_hsa_116	chr2:85109645-85109720	+	GTGGCCACAGAGAAGCCCGCC	Yes	Yes	No	0	0	0	No	
NM_hsa_116	chr2:85109655-85109713	+	GTGGCCACAGAGAAGCCCGCC	Yes	Yes	No	0	0	0	No	
NM_hsa_1160	chr19:49957932-49957988	+	AGGAGGCCAGAGCAATGATCCAAG	Yes	No	No	0	0	0	No	
NM_hsa_1161	chr1:11586568-11586642	-	TCAGGCTCCGTCCCCTCCCT	Yes	No	No	0.379905	0.0662225	0.171001	Yes	
NM_hsa_1161	chr1:11586622-11586669	-	TCAGGCTCCGTCCCCTCCCT	Yes	No	No	0.379905	0.0662225	0.171001	Yes	
NM_hsa_1162	chr1:11586568-11586642	-	GTTGGGGACAGAGCCCTGGG	Yes	No	No	0	0	0	No	
NM_hsa_1163	chr17:79637501-79637564	+	TTCCCATGACGGCCCTCTGCAG	Yes	No	No	0	0	0	No	
NM_hsa_1164	chr17:79637501-79637564	+	GGGGAGGGGTCTGCCCTGGGAGCC	Yes	No	No	0	0	0	No	
NM_hsa_1165	chr14:70075954-70076005	-	TACTTTTCTGTGGCACTTTGG	Yes	Yes	No	0	0.132445	0	No	
NM_hsa_1165	chr14:70075947-70076015	+	TACTTTTCTGTGGCACTTTGG	Yes	Yes	No	0	0.132445	0	No	
NM_hsa_1165	chr14:70075957-70076008	+	TACTTTTCTGTGGCACTTTGG	Yes	Yes	No	0	0.132445	0	No	
NM_hsa_1166	chr14:70075957-70076008	+	AAAGTGCCACAGAAAAGTAGA	Yes	No	No	0	0	0	No	
NM_hsa_1167	chr20:49184677-49184805	+	TCGCCTTTCGCTGCCTGGTAGA	Yes	Yes	Yes	0	0	0	No	
NM_hsa_1167	chr20:49184712-49184778	+	TCGCCTTTCGCTGCCTGGTAGA	Yes	Yes	Yes	0	0	0	No	
NM_hsa_1167	chr20:49184702-49184785	+	TCGCCTTTCGCTGCCTGGTAGA	Yes	Yes	Yes	0	0	0	No	
NM_hsa_1168	chr20:49184712-49184778	+	CACCAGGCAGAGAGAGGTGGGT	Yes	No	No	0	0	0	No	
NM_hsa_117	chr2:85109655-85109713	+	CTGGGCTTCTCTCTGGCCACAC	Yes	No	No	0	0	0	Yes	
NM_hsa_1171	chr17:19882712-19882775	+	TTGTTAGAGCAGGTTAAAAGAT	Yes	Yes	Yes	0.113971	0	0	Yes	
NM_hsa_1171	chr17:19882699-19882784	+	TTGTTAGAGCAGGTTAAAAGAT	Yes	Yes	Yes	0.113971	0	0	Yes	
NM_hsa_1171	chr17:19882686-19882804	+	TTGTTAGAGCAGGTTAAAAGAT	Yes	Yes	Yes	0.113971	0	0	Yes	
NM_hsa_1172	chr17:19882712-19882775	+	CCCTTAACCTGCTCTAATAATAATA	Yes	No	No	0	0	0	Yes	
NM_hsa_1173	chr4:129471308-129471370	+	CTACGTGACCAGAAGTTTCAG	Yes	Yes	No	0	0	0	No	
NM_hsa_1173	chr4:129471296-129471379	+	CTACGTGACCAGAAGTTTCAG	Yes	Yes	No	0	0	0	No	
NM_hsa_1174	chr4:129471308-129471370	+	GAAACTTCTGGCCCCATAGGA	Yes	No	No	0	0	0	No	
NM_hsa_1175	chrX:49021435-49021492	+	CCTGAAGCCCCTTCCACTGGTAG	Yes	Yes	No	0	0	0	No	
NM_hsa_1175	chrX:49021425-49021502	+	CCTGAAGCCCCTTCCACTGGTAG	Yes	Yes	No	0	0	0	No	
NM_hsa_1176	chrX:49021435-49021492	+	GCCTTTGTTGGGGTGCTCTCCTT	Yes	No	No	0	0	0	No	
NM_hsa_1177	chr9:132098562-132098698	+	CACAGCTCTTGGGGAGTGGAG	Yes	Yes	Yes	0	0	0	Yes	
NM_hsa_1177	chr9:132098592-132098670	+	CACAGCTCTTGGGGAGTGGAG	Yes	Yes	Yes	0	0	0	Yes	
NM_hsa_1177	chr9:132098602-132098665	+	CACAGCTCTTGGGGAGTGGAG	Yes	Yes	Yes	0	0	0	Yes	
NM_hsa_1178	chr9:132098602-132098665	+	CCGCCCCCAGAGCCTGTGTT	Yes	No	No	0	0	0	No	
NM_hsa_1179	chr2:98280770-98280842	+	TTCACCGGTCCAGGAAGA	Yes	No	No	0	0	0	No	
NM_hsa_118	chr3:42912136-42912210	+	TCAGGCTCTGAGGCAAGGTGGA	Yes	Yes	No	0	0	0	No	
NM_hsa_118	chr3:42912126-42912216	+	TCAGGCTCTGAGGCAAGGTGGA	Yes	Yes	No	0	0	0	No	
NM_hsa_1180	chr2:98280770-98280842	+	TTTCCCGCGTCCCAG	Yes	No	No	0	0	0	No	
NM_hsa_1183	chr16:4384744-4384804	+	ATGGGACGGGGGGCTCTGGGCCAGC	Yes	No	No	0.113971	0.0662225	0	No	KapB+cMyc>KapB>H
NM_hsa_1184	chr16:4384744-4384804	+	TAGCCCTCCCCTCGCACCCCAGGA	Yes	No	No	0.170957	0	0	No	
NM_hsa_1185	chr3:137844488-137844544	+	TAGAACTCTCTTCTCTTGCAG	Yes	Yes	No	0	0	0	No	
NM_hsa_1185	chr3:137844476-137844553	+	TAGAACTCTCTTCTCTTGCAG	Yes	Yes	No	0	0	0	No	
NM_hsa_1186	chr3:137844488-137844544	+	GCTGAGAAAAGAGAGTTCCAGG	Yes	No	No	0	0	0	No	
NM_hsa_1187	chr17:73752443-73752511	+	CGAGGAGGATCAGGCCAGGGGTGG	Yes	Yes	No	0	0	0	No	
NM_hsa_1187	chr17:73752433-73752517	+	CGAGGAGGATCAGGCCAGGGGTGG	Yes	Yes	No	0	0	0	No	
NM_hsa_1188	chr17:73752443-73752511	+	GCCCCCACCTGATCCCCCCAGG	Yes	No	No	0	0	0	No	
NM_hsa_1189	chr7:150759418-150759466	-	GCGTGCAGACTTGACCTG	Yes	No	No	0	0	0	No	
NM_hsa_119	chr3:42912136-42912210	+	GAAGTTGCTTTGCCTGTGG	Yes	No	No	0	0	0	No	
NM_hsa_1190	chr7:150759418-150759466	-	GCCGGGTCTGAGACGG	Yes	No	No	0	0	0	No	
NM_hsa_1193	chr16:12594560-12594633	+	AGCTTGTCGGATGTGCAGATT	Yes	Yes	No	0	0	0	No	
NM_hsa_1193	chr16:12594550-12594641	+	AGCTTGTCGGATGTGCAGATT	Yes	Yes	No	0	0	0	No	
NM_hsa_1194	chr16:12594560-12594633	+	TCTGCATGTTCACAGGCCCC	Yes	No	No	0	0	0	No	
NM_hsa_1195	chr12:28280283-28280330	-	AAGAAGATAAGGATGGTTGA	Yes	Yes	No	0	0	0	No	
NM_hsa_1195	chr12:28280255-28280340	-	AAGAAGATAAGGATGGTTGA	Yes	Yes	No	0	0	0	No	
NM_hsa_1196	chr12:28280283-28280330	-	AACCATCCTTACCTTCTTAG	Yes	No	No	0	0	0	No	
NM_hsa_1199	chr8:101170158-101170194	+	TGGCCGTCGGCAGATCTG	Yes	No	No	0	0	0	No	
NM_hsa_120	chr2:11636116-11636194	+	TGTGCCTGACTGTGAAGCTGG	Yes	Yes	No	0	0	0	No	
NM_hsa_120	chr2:11636130-11636185	+	TGTGCCTGACTGTGAAGCTGG	Yes	Yes	No	0	0	0	No	
NM_hsa_1200	chr8:101170158-101170194	+	GATCTGCCGGACTGCG	Yes	No	No	0	0	0	No	
NM_hsa_1201	chr2:175546628-175546706	+	TCTGATTTAGAGCTCTTTGCGA	Yes	Yes	Yes	0	0	0	No	
NM_hsa_1201	chr2:175546641-175546697	+	TCTGATTTAGAGCTCTTTGCGA	Yes	Yes	Yes	0	0	0	No	
NM_hsa_1201	chr2:175546603-175546731	+	TCTGATTTAGAGCTCTTTGCGA	Yes	Yes	Yes	0	0	0	No	
NM_hsa_1202	chr2:175546641-175546697	+	CCAAAGAGCTCAAAGTCTAACC	Yes	No	No	0	0	0	No	
NM_hsa_1203	chr3:50397169-50397234	+	TCCGTCGGTTCTCAGGAGGTGG	Yes	Yes	Yes	0	0	0	Yes	
NM_hsa_1203	chr3:50397129-50397267	+	TCCGTCGGTTCTCAGGAGGTGG	Yes	Yes	Yes	0	0	0	Yes	
NM_hsa_1203	chr3:50397159-50397241	+	TCCGTCGGTTCTCAGGAGGTGG	Yes	Yes	Yes	0	0	0	Yes	
NM_hsa_1204	chr3:50397169-50397234	+	ACCCCTGAAAGCGGCGGTGA	Yes	No	No	0	0	0	No	
NM_hsa_1205	chr14:36673763-36673821	+	TGTCCAGGGCCTTACCTCTTCAGA	Yes	Yes	No	0	0	0	Yes	
NM_hsa_1205	chr14:36673753-36673829	+	TGTCCAGGGCCTTACCTCTTCAGA	Yes	Yes	No	0	0	0	Yes	
NM_hsa_1206	chr14:36673763-36673821	+	TGGGGTGGAGCCCTGGAGTCT	Yes	No	No	0	0	0	No	
NM_hsa_121	chr2:11636130-11636185	+	AGCTCCAAAAAGCGGCACGGC	Yes	No	No	0	0	0	No	
NM_hsa_1211	chr16:6988653-6988791	+	TGAAACCAGTTTGGCTGTCAGG	Yes	Yes	Yes	0	0	0	No	
NM_hsa_1211	chr16:6988676-6988761	+	TGAAACCAGTTTGGCTGTCAGG	Yes	Yes	Yes	0	0	0	No	
NM_hsa_1213	chr19:55736839-55736926	+	TTCAGATCCTCCTCATGCCTGA	Yes	Yes	No	0	0	0	No	
NM_hsa_1213	chr19:55736851-55736917	+	TTCAGATCCTCCTCATGCCTGA	Yes	Yes	No	0	0	0	No	
NM_hsa_1214	chr19:55736851-55736917	+	AGGAGATGAGGAGGAAGTGGAGG	Yes	No	No	0	0	0	No	
NM_hsa_1215	chr1:6686279-6686362	+	GAACCCAGCTTCGAGGCTGCAGT	Yes	Yes	Yes	0	0.0662225	0	Yes	
NM_hsa_1215	chr1:6686264-6686374	+	GAACCCAGCTTCGAGGCTGCAGT	Yes	Yes	Yes	0	0.0662225	0	Yes	
NM_hsa_1217	chr5:1309432-1309488	+	TCACACGGTATTGACTGGAGT	Yes	Yes	No	0	0	0	Yes	
NM_hsa_1217	chr5:1309419-1309497	+	TCACACGGTATTGACTGGAGT	Yes	Yes	No	0	0	0	Yes	
NM_hsa_1218	chr5:1309432-1309488	+	GCCAGTCAATACCTTGTGAAT	Yes	No	No	0	0	0	No	
NM_hsa_1219	chr16:68387950-68388048	+	CACGTGGCTCCTGCTTGTCATC	Yes	Yes	Yes	0	0	0	Yes	
NM_hsa_1219	chr16:68387958-68388038	+	CACGTGGCTCCTGCTTGTCATC	Yes	Yes	Yes	0	0	0	Yes	
NM_hsa_1219	chr16:68387971-68388029	+	CACGTGGCTCCTGCTTGTCATC	Yes	Yes	Yes	0	0	0	Yes	
NM_hsa_122	chr1:223949800-223949889	+	TGTCTCTGCCTCACCTTCCAGT	Yes	Yes	No	0.3989	0.0662225	0.146572	Yes	
NM_hsa_122	chr1:223949803-223949898	+	TGTCTCTGCCTCACCTTCCAGT	Yes	Yes	No	0.3989	0.0662225	0.146572	Yes	
NM_hsa_1220	chr16:68387971-68388029	+	TGAGGAGCATGGGCCGTGCTGTGTA	Yes	No	No	0	0	0	Yes	
NM_hsa_1221	chr2:111892952-111893031	+	TTGTGTCCAGTTGTTGGGGGA	Yes	Yes	No	0	0	0	Yes	
NM_hsa_1221	chr2:111892962-111893024	+	TTGTGTCCAGTTGTTGGGGGA	Yes	Yes	No	0	0	0	Yes	
NM_hsa_1222	chr2:111892962-111893024	+	CCTCAGCCACTGCTGACACCAGG	Yes	No	No	0	0	0	No	
NM_hsa_1223	chr11:319456-319505	-	TGTGTCCTGCAGGTCTCAGCT	Yes	Yes	No	0	0	0	No	
NM_hsa_1223	chr11:319447-319517	-	TGTGTCCTGCAGGTCTCAGCT	Yes	Yes	No	0	0	0	No	
NM_hsa_1223	chr11:310275-310324	+	TGTGTCCTGCAGGTCTCAGCT	Yes	Yes	No	0	0	0	No	
NM_hsa_1224	chr11:319456-319505	-	CTGGATCCAGGACCCTGG	Yes	No	No	0	0	0	No	
NM_hsa_1224	chr11:310275-310324	+	CTGGATCCAGGACCCTGG	Yes	No	No	0	0	0	No	
NM_hsa_1225	chr2:52929666-52929750	+	ATGACTCTGACAACTAGAAAGT	Yes	No	No	0	0	0	No	
NM_hsa_1226	chr2:52929666-52929750	+	CTTCTAGTTTTCAGAGTCGCAA	Yes	No	No	0	0	0	No	
NM_hsa_1227	chr13:20981181-20981248	-	TTTGTAGAAATAAATGTCCCTTT	Yes	Yes	Yes	0	0	0	No	
NM_hsa_1227	chr13:20981184-20981251	+	TTTGTAGAAATAAATGTCCCTTT	Yes	Yes	Yes	0	0	0	No	
NM_hsa_1227	chr13:20981155-20981285	+	TTTGTAGAAATAAATGTCCCTTT	Yes	Yes	Yes	0	0	0	No	
NM_hsa_1227	chr13:20981171-20981260	+	TTTGTAGAAATAAATGTCCCTTT	Yes	Yes	Yes	0	0	0	No	
NM_hsa_1227	chr13:20981152-20981272	-	TTTGTAGAAATAAATGTCCCTTT	Yes	Yes	Yes	0	0	0	No	
NM_hsa_1228	chr13:20981181-20981248	-	AGGGACATTTATTTCTACAAAAC	Yes	No	No	0	0	0	No	
NM_hsa_1228	chr13:20981184-20981251	+	AGGGACATTTATTTCTACAAAAC	Yes	No	No	0	0	0	No	
NM_hsa_1229	chr15:43467107-43467171	+	TTTCTCAATCCTGACTGCTTAG	Yes	No	No	0	0	0	No	
NM_hsa_123	chr1:223949800-223949889	+	TGGGAGGTGGCTGCCATG	Yes	No	No	0	0	0	No	

Additional file 5

Name	locus	strand	sequence	mirdeep2	mireap	miranalyzer	RPM_HU_KapB_cMyc	RPM_HU_KaposinB	RPM_HUVEC	IP_confidence	annotation
NM_hsa_1230	chr15:43467107-43467171	+	GTGCAGTCAAGACTGAGAAGCA	Yes	No	No	0	0	0	No	
NM_hsa_1231	chr7:35602322-35602432	-	TCATGTCTCACTTGTCAGAACTG	Yes	Yes	Yes	0.0569857	0.132445	0	No	
NM_hsa_1231	chr7:35602346-35602407	-	TCATGTCTCACTTGTCAGAACTG	Yes	Yes	Yes	0.0569857	0.132445	0	No	
NM_hsa_1231	chr7:35602339-35602417	-	TCATGTCTCACTTGTCAGAACTG	Yes	Yes	Yes	0.0569857	0.132445	0	No	
NM_hsa_1232	chr7:35602346-35602407	-	GAGCTGCAAGTGAGGCATGGAA	Yes	No	No	0	0	0	No	
NM_hsa_1233	chr5:139044346-139044409	+	AAGTGTGGGCTCTAGAGTTGGG	Yes	Yes	Yes	0.170957	0	0	Yes	
NM_hsa_1233	chr5:139044306-139044444	+	AAGTGTGGGCTCTAGAGTTGGG	Yes	Yes	Yes	0.170957	0	0	Yes	
NM_hsa_1233	chr5:139044336-139044418	+	AAGTGTGGGCTCTAGAGTTGGG	Yes	Yes	Yes	0.170957	0	0	Yes	
NM_hsa_1234	chr5:139044346-139044409	+	CACCTCCACTGTCCACACTTGC	Yes	No	No	0	0	0	Yes	
NM_hsa_1235	chr18:18692018-18692089	+	CGGACGGAGGCGGAGCTCTGG	Yes	No	No	0	0	0	No	
NM_hsa_1236	chr18:18692018-18692089	+	CCAGCTCTGTTTCCTCTGGG	Yes	No	No	0	0	0	No	
NM_hsa_1237	chr4:142629145-142629206	+	CAGCAAGGCCATGCAGCTCTGC	Yes	No	No	0	0	0.0732862	Yes	
NM_hsa_1238	chr4:142629145-142629206	+	AGGACTGGATGTCGGGCTGCA	Yes	No	No	0.170957	0	0	Yes	
NM_hsa_1239	chr4:5209402-5209467	+	AGAAAGTGTCTGAGACTGCAG	Yes	No	No	0	0	0	No	
NM_hsa_124	chr6:39042090-39042158	+	TTTTTTGCTGGAACATTTCTGG	Yes	Yes	No	0	0	0	Yes	
NM_hsa_124	chr6:39042078-39042167	+	TTTTTTGCTGGAACATTTCTGG	Yes	Yes	No	0	0	0	Yes	
NM_hsa_1240	chr4:5209402-5209467	+	GCAGTCTCAGACACTTTCTTA	Yes	No	No	0	0	0	No	
NM_hsa_1241	chr22:31459487-31459566	-	TAGGGACTCGCTATGTTGCCCAGA	Yes	Yes	No	0	0	0	Yes	
NM_hsa_1243	chr4:73433950-73434019	+	CCCGACTCTGGCGGCAGCAGC	Yes	No	No	0	0	0	No	
NM_hsa_1244	chr4:73433950-73434019	+	TGCCCGAAAAAACCGTGAGTCAGTT	Yes	No	No	0	0	0	No	
NM_hsa_1245	chr1:176176797-176176878	+	AATCGCGGAATGTAGGTG	Yes	No	No	0	0	0	No	
NM_hsa_1246	chr1:176176797-176176878	+	CCACATTCCGGAACGG	Yes	No	No	0	0	0	No	
NM_hsa_1247	chr3:48471674-48471746	+	AGGGAACCTGGGCTGCAGAGG	Yes	Yes	No	0	0	0	No	
NM_hsa_1247	chr3:48471664-48471754	+	AGGGAACCTGGGCTGCAGAGG	Yes	Yes	No	0	0	0	No	
NM_hsa_1248	chr3:48471674-48471746	+	TTCCCAGCCCAGGACCCAGA	Yes	No	No	0	0	0	No	
NM_hsa_1249	chr14:33409145-33409209	+	GCCGAGCCCAGAGGAGCG	Yes	No	No	0	0	0	No	
NM_hsa_125	chr6:39042090-39042158	+	GGAAATGTTCTAGCCAAAAAAG	Yes	No	No	0	0	0	No	
NM_hsa_1250	chr14:33409145-33409209	+	CCCTCCGCTCGGCAA	Yes	No	No	0	0	0	No	
NM_hsa_1251	chr19:50373014-50373100	+	CCCGGATCGGCCTCAGATTAG	Yes	No	No	0.113971	0	0	No	
NM_hsa_1252	chr19:50373014-50373100	+	GGAAGGACGGCGGGGAT	Yes	No	No	0	0	0	No	
NM_hsa_1254	chr9:131379970-131380054	+	GGCTATTCAGGTAAGGAGGCCGCC	Yes	No	No	0	0	0	No	
NM_hsa_1255	chr16:47708273-47708330	+	CTGAAACCACAATTGCTTTGC	Yes	Yes	Yes	0	0	0	Yes	
NM_hsa_1255	chr16:47708238-47708364	+	CTGAAACCACAATTGCTTTGC	Yes	Yes	Yes	0	0	0	Yes	
NM_hsa_1255	chr16:47708261-47708339	+	CTGAAACCACAATTGCTTTGC	Yes	Yes	Yes	0	0	0	Yes	
NM_hsa_1256	chr16:47708273-47708330	+	AAAAGTAATCGTGGTTTTTGCC	Yes	No	No	1.58927	1.83573	1.85665	Yes	H>KapB>KapB+cMyc
NM_hsa_1257	chr13:32378944-32378997	-	ATTGCAATGGACATGAACTCT	Yes	Yes	Yes	0	0	0	No	
NM_hsa_1257	chr13:32378937-32379007	-	ATTGCAATGGACATGAACTCT	Yes	Yes	Yes	0	0	0	No	
NM_hsa_1257	chr13:32378906-32379032	-	ATTGCAATGGACATGAACTCT	Yes	Yes	Yes	0	0	0	No	
NM_hsa_1258	chr13:32378944-32378997	-	AGTTTATGTCGATTGCAATTT	Yes	No	No	0	0	0	No	
NM_hsa_1259	chr3:38367136-38367198	+	CTGCATCTCAGGTCTGCACCTCTG	Yes	No	No	0	0	0	No	
NM_hsa_126	chr5:139166225-139166307	+	TGATTGGTTCAGGGCTGGGAGC	Yes	Yes	No	0	0	0	Yes	
NM_hsa_126	chr5:139166235-139166300	+	TGATTGGTTCAGGGCTGGGAGC	Yes	Yes	No	0	0	0	Yes	
NM_hsa_1260	chr3:38367136-38367198	+	CAGGGCAACTCTGGGATCACAT	Yes	No	No	0	0	0	No	
NM_hsa_1265	chr18:48556415-48556457	-	TACAGGGCAGCGCCAGCC	Yes	No	No	0	0	0	No	
NM_hsa_1266	chr6:81128992-81129065	+	ATGCTGTAGTTCTCTGCTGGT	Yes	Yes	Yes	0	0	0	No	
NM_hsa_1266	chr6:81128972-81129088	+	ATGCTGTAGTTCTCTGCTGGT	Yes	Yes	Yes	0	0	0	No	
NM_hsa_1266	chr6:81129002-81129059	+	ATGCTGTAGTTCTCTGCTGGT	Yes	Yes	Yes	0	0	0	No	
NM_hsa_1267	chr6:81129002-81129059	+	CAGCACAGAAGCTATGGTGTGG	Yes	No	No	0	0	0	No	
NM_hsa_127	chr5:139166235-139166300	+	TGCCCAGGCTGGGCCGATGAGACT	Yes	No	No	0	0	0	No	
NM_hsa_1270	chr1:113424672-113424751	+	TTCAGGTCTGGGGCTGAAACCTG	Yes	Yes	No	0.227943	0.26489	0.219859	No	
NM_hsa_1270	chr1:113424682-113424743	+	TTCAGGTCTGGGGCTGAAACCTG	Yes	Yes	No	0.227943	0.26489	0.219859	No	
NM_hsa_1271	chr1:113424682-113424743	+	GAGGCAGTCCCAGGCCCGAAAG	Yes	No	No	0	0	0	No	
NM_hsa_1274	chr16:51272972-51273049	-	CACTAGATTGTGAGCTGCTTGAG	Yes	No	No	33.6282	29.6699	11.975	Yes	KapB+cMyc>KapB>H
NM_hsa_1275	chr16:51272972-51273049	-	CGAGCAGCTTGCACATGGGCTG	Yes	No	No	0	0	0	No	
NM_hsa_1276	chr16:27181508-27181589	+	AGCCTAGGAGAGCAAGGCTGTGG	Yes	Yes	No	0	0	0	No	
NM_hsa_1278	chr22:49144502-49144580	+	CTGGCCCTGACGGAACCTGG	Yes	Yes	No	0	0	0	No	
NM_hsa_1278	chr22:49144515-49144571	+	CTGGCCCTGACGGAACCTGG	Yes	Yes	No	0	0	0	No	
NM_hsa_1279	chr22:49144515-49144571	+	AGGAACCGCAGCTGGCCAGGG	Yes	No	No	0	0	0	No	
NM_hsa_128	chr6:158914875-158914957	-	TGAGGTAGTAGGTGGTGTGC	Yes	No	No	10.3954	7.70178	1.78214	Yes	KapB+cMyc>KapB>H
NM_hsa_1280	chr8:133962238-133962325	+	CTGTGTTCTAACAAGTGGTCCAG	Yes	No	No	0	0	0	No	
NM_hsa_1281	chr8:133962238-133962325	+	GGAAGCTGGTTAGAAATGCAGAG	Yes	No	No	0	0.132445	0	No	
NM_hsa_1282	chr2:225235712-225235792	+	GAAGCCAGGCTGTTAGGCTAG	Yes	Yes	No	0	0	0	No	
NM_hsa_1282	chr2:225235722-225235785	+	GAAGCCAGGCTGTTAGGCTAG	Yes	Yes	No	0	0	0	No	
NM_hsa_1283	chr2:225235722-225235785	+	AGCCTGACAGCCTCGCTTCCAA	Yes	No	No	0	0	0	No	
NM_hsa_1284	chr6:33169450-33169518	+	TACTTGACCTTGACTCTCCCTCA	Yes	Yes	No	2.50737	1.39067	1.02601	Yes	KapB+cMyc>KapB>H
NM_hsa_1284	chr6:33169434-33169527	+	TACTTGACCTTGACTCTCCCTCA	Yes	Yes	No	2.50737	1.39067	1.02601	Yes	KapB+cMyc>KapB>H
NM_hsa_1285	chr6:33169450-33169518	+	TGGGAGAGAGAAGGGCTGGTTCT	Yes	No	No	0.284929	0.0662225	0.58629	Yes	
NM_hsa_1286	chr6:35055799-35055866	+	ACAGAGCTGCTGGAGGACAGGGA	Yes	Yes	No	0	0	0	No	
NM_hsa_1286	chr6:35055789-35055882	+	ACAGAGCTGCTGGAGGACAGGGA	Yes	Yes	No	0	0	0	No	
NM_hsa_1287	chr6:35055799-35055866	+	CATTGTCCTCTGGGAGCTTTCTGACA	Yes	No	No	0	0	0	No	
NM_hsa_1288	chr11:128556313-128556351	+	CCCGGGGACGGGACTGAG	Yes	No	No	0	0	0	No	
NM_hsa_1289	chr11:128556313-128556351	+	CACCGTCCCCTGGCC	Yes	No	No	0	0	0	No	
NM_hsa_129	chr6:158914875-158914957	-	AAGATCAGAACTGGCTGAGG	Yes	No	No	0	0	0	No	
NM_hsa_1290	chr21:45622824-45622899	+	CTCGGCCTGTGGGCACTGAGC	Yes	Yes	No	0	0	0	No	
NM_hsa_1290	chr21:45622834-45622893	+	CTCGGCCTGTGGGCACTGAGC	Yes	Yes	No	0	0	0	No	
NM_hsa_1291	chr21:45622834-45622893	+	TCAGTAAATGCTTGCAGGCCGATGC	Yes	No	No	0	0	0	No	
NM_hsa_1294	chr1:26515033-26515088	+	ACAACAGGGCGTGGAGCTCAGA	Yes	No	No	0	0	0	Yes	
NM_hsa_1295	chr1:26515033-26515088	+	TGTGCTTTCCACCCTGCCTGCA	Yes	No	No	0	0	0	No	
NM_hsa_1296	chr12:14743647-14743785	+	ACTTCTTTGCAGCTAGGACTGT	Yes	Yes	Yes	0	0	0	No	
NM_hsa_1296	chr12:14743677-14743762	+	ACTTCTTTGCAGCTAGGACTGT	Yes	Yes	Yes	0	0	0	No	
NM_hsa_1296	chr12:14743687-14743758	+	ACTTCTTTGCAGCTAGGACTGT	Yes	Yes	Yes	0	0	0	No	
NM_hsa_1297	chr12:14743687-14743758	+	AGTCCTAGCTGCAAAGAAGTGG	Yes	No	No	0	0	0	No	
NM_hsa_1298	chr7:75508112-75508179	-	CTACTCGGGCTGGTACTGA	Yes	No	No	0	0	0	No	
NM_hsa_1299	chr7:75508112-75508179	-	GGGGCCACTTCCGGGTAGGA	Yes	No	No	0	0	0	No	
NM_hsa_13	chr1:149900286-149900341	+	TGGATGGGATCGGAGCTCTAGA	Yes	Yes	No	0.0569857	0	0	Yes	
NM_hsa_13	chr1:149900276-149900350	+	TGGATGGGATCGGAGCTCTAGA	Yes	Yes	No	0.0569857	0	0	Yes	
NM_hsa_1300	chr6:30710743-30710805	+	ATTGCCACATCTCGGATTCGC	Yes	Yes	No	0	0	0	No	
NM_hsa_1300	chr6:30710733-30710820	+	ATTGCCACATCTCGGATTCGC	Yes	Yes	No	0	0	0	No	
NM_hsa_1301	chr6:30710743-30710805	+	AAACCGCAGACTGGGCAATGA	Yes	No	No	0	0	0	No	
NM_hsa_1304	chr1:12601932-12602013	+	TTCCCCCAAACTCTGATTCACC	Yes	Yes	Yes	0	0.0662225	0	No	
NM_hsa_1304	chr1:12601943-12602004	+	TTCCCCCAAACTCTGATTCACC	Yes	Yes	Yes	0	0.0662225	0	No	
NM_hsa_1304	chr1:12601910-12602038	+	TTCCCCCAAACTCTGATTCACC	Yes	Yes	Yes	0	0.0662225	0	No	
NM_hsa_1305	chr1:12601943-12602004	+	TGGATCAGGGCTCTGAGGGTGGGC	Yes	No	No	0	0	0	No	
NM_hsa_1306	chr8:46951885-46951943	-	ACTATCTGTGGGATTATGAC	Yes	Yes	Yes	2.48363	1.09267	1.16647	Yes	
NM_hsa_1306	chr8:46951864-46951968	-	ACTATCTGTGGGATTATGAC	Yes	Yes	Yes	2.48363	1.09267	1.16647	Yes	
NM_hsa_1306	chr8:46951869-46951953	-	ACTATCTGTGGGATTATGAC	Yes	Yes	Yes	2.48363	1.09267	1.16647	Yes	
NM_hsa_1307	chr8:46951885-46951943	-	CAGAATCCCACGGAAGGATATGG	Yes	No	No	0	0	0	No	
NM_hsa_1308	chr1:178712679-178712756	+	TTAGTTCTGTGGCAACATCGA	Yes	Yes	Yes	0.0569857	0.0662225	0	Yes	
NM_hsa_1308	chr1:178712650-178712786	+	TTAGTTCTGTGGCAACATCGA	Yes	Yes	Yes	0.0569857	0.0662225	0	Yes	
NM_hsa_1308	chr1:178712692-178712747	+	TTAGTTCTGTGGCAACATCGA	Yes	Yes	Yes	0.0569857	0.0662225	0	Yes	
NM_hsa_1309	chr1:178712692-178712747	+	GATGTTGCCATGGAACTAATT	Yes	No	No	0	0	0	No	
NM_hsa_1310	chr10:80376290-80376371	+	AGGCTGCTGTATTCCTCTGTGC	Yes	Yes	Yes	0	0	0	No	
NM_hsa_1310	chr10:80376300-80376363	+	AGGCTGCTGTATTCCTCTGTGC	Yes	Yes	Yes	0	0	0	No	
NM_hsa_1310	chr10:80376285-80376373	+	AGGCTGCTGTATTCCTCTGTGC	Yes	Yes	Yes	0	0	0	No	
NM_hsa_1311	chr10:80376300-80376363	+	ACAGAGGGACTACTCAGCCAAT	Yes	No	No	0	0	0	No	
NM_hsa_1312	chr15:24920989-24921066	-	GCAGGGGCCGGCGGCGGCA	Yes	No	No	0.626843	0.26489	0.0732862	Yes	KapB+cMyc>KapB>H
NM_hsa_1313	chr15:24920989-24921066	-	GAGCTAGCCGTGCCTCCTACCG	Yes	No	No	0	0	0	No	
NM_hsa_1316	chr22:42835753-42835805	+	TCCGCCTTTGGATCTCTGGACC	Yes	Yes	No	0	0	0	No	
NM_hsa_1316	chr22:42835743-42835832	+	TCCGCCTTTGGATCTCTGGACC	Yes	Yes	No	0	0	0	No	
NM_hsa_1317	chr22:42835753-42835805	+	TCCAGCAGGTCACAGGTGACCA	Yes	No	No	0	0	0	No	
NM_hsa_1318	chr22:43011340-43011397	+	GATGCCTGGGAGTTGCGATCTGC	Yes	No	No	4.58735	4.41483	4.56818	Yes	
NM_hsa_1319	chr22:43011340-43011397	+	AGGTCGCCCTCAAGGTGACCC	Yes	No	No	0	0	0	No	
NM_hsa_1320	chr10:24646848-24646890	+	AGGCTGTAGGCACCACTGC	Yes	No	No	0	0	0	No	
NM_hsa_1321	chr10:24646848-24646890	+	TGTGAAGTGTGCAGGCTTG	Yes	No	No	0	0	0	No	
NM_hsa_1322	chr10:6018943-6019000	-	AGGCGCCTGGACTGCAGGG	Yes	No	No	0	0	0.0732862	No	
NM_hsa_1322	chr10:6018981-6019043	-	AGGCGCCTGGACTGCAGGG	Yes	No	No	0	0	0.0732862	No	
NM_hsa_1323	chr10:6018943-6019000	-	CTCGGAGGGGTCCGG	Yes	No	No	0	0	0	No	
NM_hsa_1324	chr19:13063444-13063504	+	CTTCCCCACCCTCTCCTGCAGC	Yes	No	No	0.170957	0.198668	0	Yes	
NM_hsa_1325	chr19:13063444-13063504	+	TGGGGAGAGGAGGGGACCAGGG	Yes	No	No	0	0	0	Yes	
NM_hsa_1326	chr19:38642729-38642823	+	TCGTGAGCTGGGCGTGGTAGA	Yes	Yes	No	0	0	0	Yes	
NM_hsa_1326	chr19:38642756-38642814	+	TCGTGAGCTGGGCGTGGTAGA	Yes	Yes	No	0	0	0	Yes	
NM_hsa_1327	chr19:38642756-38642814	+	ATCCACTCCCCAACTCTATGAGG	Yes	No	No	0	0	0	No	
NM_hsa_1328	chrM:9511-9565	-	CTGTTGGGGGCCAGTGCCCTCC	Yes	Yes	No	0	0.132445	0	No	
NM_hsa_1328	chrM:9502-9575	-	CTGTTGGGGGCCAGTGCCCTCC	Yes	Yes	No	0	0.132445	0	No	
NM_hsa_1329	chrM:9511-9565	-	GGGGTAGGGGCTAGGCTGGAGTGGT	Yes	No	No	0	0	0	No	
NM_hsa_1330	chr2:11546162-11546229	-	TCTGATTATAGTTGATACAG	Yes	Yes	No	0	0	0	No	
NM_hsa_1330	chr2:11546169-11546219	-	TCTGATTATAGTTGATACAG	Yes	Yes	No	0	0	0	No	
NM_hsa_1331	chr2:11546169-11546219	-	GTATCAACTATAATCAAACG	Yes	No	No	0	0	0	No	
NM_hsa_1333	chr1:27022478-27022551	+	CCCCCTCGCCTCCCTCCCTCCCTC	Yes	No	No	0	0	0	No	
NM_hsa_1334	chr15:87422341-87422402	+	AAGAGGATGTCTGGCAGGTGG	Yes	No	No	0	0	0	No	
NM_hsa_1334	chr15:85827076-85827137	-	AAGAGGATGTCTGGCAGGTGG	Yes	No	No	0	0	0	No	
NM_hsa_1335	chr15:87422341-87422402	+	TCTTGCCCCATTCATCCTCTCTT	Yes	No	No	0	0	0	No	
NM_hsa_1335	chr15:85827076-85827137	-	TCTTGCCCCATTCATCCTCTCTT	Yes	No	No	0	0	0	No	
NM_hsa_1336	chr17:46114826-46114881	+	TCTCCGGTCGGCGCTGGGACGA	Yes	Yes	No	0	0	0.0732862	Yes	
NM_hsa_1336	chr17:46114810-46114890	+	TCTCCGGTCGGCGCTGGGACGA	Yes	Yes	No	0	0	0.0732862	Yes	
NM_hsa_1337	chr17:46114826-46114881	+	AGCCCCGCGCCGACGGACTCAGG	Yes	No	No	0	0	0	No	
NM_hsa_1338	chr5:58999459-58999515	+	TAACTGGTTGAACAACTGTAA	Yes	No	No	0.455886	0.198668	0.58629	Yes	
NM_hsa_1339	chr5:58999459-58999515	+	TCAGTTGTTCAACCAGTTACA	Yes	No	No	0	0	0	Yes	
NM_hsa_134	chr14:21460202-21460252	+	TCTGAGCCCTGTTCTCCCTAGG	Yes	No	No	0.0569857	0.132445	0	Yes	
NM_hsa_1340	chr4:57845324-57845396	+	CGTTCACTGCTGCTCTCTGCAC	Yes	No	No	0.227943	0	0	Yes	
NM_hsa_1341	chr4:57845324-57845396	+	GTAGGGAGAGGCGGAGGGTGCA	Yes	No	No	0	0	0	No	
NM_hsa_1344	chr14:76640404-76640490	+	TTCTGCCTTTGCCAGGGATGGT	Yes	Yes	Yes	0	0	0	Yes	
NM_hsa_1344	chr14:76640417-76640481	+	TTCTGCCTTTGCCAGGGATGGT	Yes	Yes	Yes	0	0	0	Yes	
NM_hsa_1344	chr14:76640397-76640505	+	TTCTGCCTTTGCCAGGGATGGT	Yes	Yes	Yes	0	0	0	Yes	
NM_hsa_1344	chr14:76640459-76640531	+	TTCTGCCTTTGCCAGGGATGGT	Yes	Yes	Yes	0	0	0	Yes	
NM_hsa_1345	chr14:76640417-76640481	+	CCCTCCTTGGCCATGAGGGAGAACT	Yes	No	No	0	0	0	No	
NM_hsa_1346	chr1:85040768-85040829	+	CTTCATGTCTCTGGGCAGCTGC	Yes	No	No	0	0	0	No	
NM_hsa_1347	chr1:85040768-85040829	+	GGCTGCTCAAGGACTCTGATCGCT	Yes	No	No	0	0	0	No	
NM_hsa_1348	chr3:123380028-123380076	+	GTGATCGGAGGAGGGGAGTG	Yes	No	No	0	0	0	No	
NM_hsa_1349	chr3:123380028-123380076	+	CTGCGCAGGAGCTGGGCATCG	Yes	No	No	0	0	0	No	
NM_hsa_135	chr14:21460202-21460252	+	TGGGTGGGCAGTATATTCAGATA	Yes	No	No	0	0	0	No	
NM_hsa_1350	chr19:50128299-50128363	+	AGGCATCTCCACTCCTACCCAGG	Yes	No	No	0	0	0	Yes	
NM_hsa_1351	chr19:50128299-50128363	+	AGGGAGGAGAGGGGCTGCCAAG	Yes	No	No	0	0	0	No	
NM_hsa_1352	chr8:121511219-121511337	+	TTATGTCACTCCTTTGCCCAGA	Yes	Yes	Yes	0.170957	0.0662225	0	Yes	KapB+cMyc>KapB>H
NM_hsa_1352	chr8:121511254-121511308	+	TTATGTCACTCCTTTGCCCAGA	Yes	Yes	Yes	0.170957	0.0662225	0	Yes	KapB+cMyc>KapB>H
NM_hsa_1352	chr8:121511241-121511317	+	TTATGTCACTCCTTTGCCCAGA	Yes	Yes	Yes	0.170957	0.0662225	0	Yes	KapB+cMyc>KapB>H
NM_hsa_1353	chr8:121511254-121511308	+	TGAGCAAAGGATGAACACAATC	Yes	No	No	0	0	0	No	
NM_hsa_1354	chr8:72897007-72897093	+	ACCAATATCCAGATATCAAGA	Yes	Yes	Yes	0	0	0	No	

Additional file 5

Name	locus	strand	sequence	mirdeep2	mireap	miranalyzer	RPM_HU_KapB_cMyc	RPM_HU_KaposinB	RPM_HUVEC	IP_confidence	annotation
NM_hsa_1354	chr8:72896997-72897113	+	ACCAATATCCAGATATCAAGA	Yes	Yes	Yes	0	0	0	No	
NM_hsa_1354	chr8:72897021-72897084	+	ACCAATATCCAGATATCAAGA	Yes	Yes	Yes	0	0	0	No	
NM_hsa_1355	chr8:72897021-72897084	+	TGGTGTCTGGGTTTGTGGTCC	Yes	No	No	0	0	0	No	
NM_hsa_1356	chr19:58024384-58024431	-	CACGTCCCTGTTCGGGCGCCA	Yes	No	No	716.254	518.787	850.78	Yes	
NM_hsa_1357	chr19:58024384-58024431	-	GCTGTCGGTTCGGGACTGAA	Yes	No	No	0	0	0	No	
NM_hsa_1358	chr1:232423543-232423603	+	TCCAGAGACTGGAGCTGACTTG	Yes	Yes	No	0	0	0	No	
NM_hsa_1358	chr1:232423533-232423610	+	TCCAGAGACTGGAGCTGACTTG	Yes	Yes	No	0	0	0	No	
NM_hsa_1359	chr1:232423543-232423603	+	AGCCAACTGCAGTCATTGGACC	Yes	No	No	0	0	0	No	
NM_hsa_136	chr12:49061316-49061376	-	TTGGCCCTGAATCAAGGC	Yes	No	No	0	0	0	No	
NM_hsa_1360	chr20:37078079-37078150	+	GCTGAAAGCCGTTTCCCGTGTT	Yes	No	No	0	0	0	No	
NM_hsa_1361	chr20:37078079-37078150	+	CTGGCGCTTTCACACACTC	Yes	No	No	0	0.0662225	0.0732862	No	H>KapB>KapB+cMyc
NM_hsa_1366	chr11:116708369-116708428	-	AGCCCAGACCCTGGCTGCAGA	Yes	Yes	No	0	0	0	No	
NM_hsa_1366	chr11:116708360-116708440	-	AGCCCAGACCCTGGCTGCAGA	Yes	Yes	No	0	0	0	No	
NM_hsa_1367	chr11:116708369-116708428	-	AGCAGCAACAGGGCCGGGGCTGG	Yes	No	No	0	0	0	No	
NM_hsa_1368	chr11:126279097-126279155	+	TGGGTTGGATTTGAGAAACAGG	Yes	Yes	No	0	0	0	No	
NM_hsa_1368	chr11:126279087-126279159	+	TGGGTTGGATTTGAGAAACAGG	Yes	Yes	No	0	0	0	No	
NM_hsa_1369	chr11:126279097-126279155	+	TGTCTCTCTCTTCTGACCCCAT	Yes	No	No	0	0	0	No	
NM_hsa_137	chr12:49061316-49061376	-	TTCAAGCAGGAGCCTAAA	Yes	No	No	0	0	0	No	
NM_hsa_1370	chr1:10575844-10575926	+	GATCCAGGTTGTGACATGCTGA	Yes	Yes	No	0	0	0	No	
NM_hsa_1370	chr1:10575855-10575917	+	GATCCAGGTTGTGACATGCTGA	Yes	Yes	No	0	0	0	No	
NM_hsa_1371	chr1:10575855-10575917	+	ATGTCTCAGCCTGGAACTC	Yes	No	No	0	0	0	No	
NM_hsa_1372	chr17:7754259-7754340	+	CTGCTCGGCCCTCCCTTCCAGA	Yes	No	No	0	0	0.0732862	No	
NM_hsa_1373	chr17:7754259-7754340	+	AGGAAGGGAAAGGGTCGATTGGG	Yes	No	No	0	0	0	No	
NM_hsa_1374	chr19:35500733-35500793	+	TCACCTCTCAAACTTCCTCAGA	Yes	No	No	0.170957	0.0662225	0	Yes	KapB+cMyc>KapB>H
NM_hsa_1375	chr19:35500733-35500793	+	TGGGGCAGCATTGGGAGTGTGA	Yes	No	No	0	0	0	No	
NM_hsa_1376	chr19:4058540-4058595	+	CCTGGCGGCCCCGAGTCAC	Yes	No	No	0	0	0	No	
NM_hsa_1383	chr13:53029459-53029516	-	CGGTTGGGACTGAGTCTG	Yes	No	No	0	0	0	No	
NM_hsa_1384	chr13:53029459-53029516	-	GACTGCAGTTGCCGGGGCCGGT	Yes	No	No	0	0	0	No	
NM_hsa_1385	chr12:66260644-66260782	+	TTCCCATCTTGACACTGGTAGA	Yes	Yes	Yes	0	0	0	No	
NM_hsa_1385	chr12:66260693-66260743	+	TTCCCATCTTGACACTGGTAGA	Yes	Yes	Yes	0	0	0	No	
NM_hsa_1385	chr12:66260681-66260752	+	TTCCCATCTTGACACTGGTAGA	Yes	Yes	Yes	0	0	0	No	
NM_hsa_1386	chr12:66260693-66260743	+	TGCTGCTGTCAAGATAGGAACA	Yes	No	No	0	0	0	No	
NM_hsa_1387	chr1:154223232-154223316	+	TAACCCAGGAGTCGGATGTTGA	Yes	Yes	No	0	0	0	Yes	
NM_hsa_139	chr4:175636932-175637007	+	TAAAGTAATCGTGGGTTTTGC	Yes	Yes	Yes	0	0	0	No	
NM_hsa_139	chr4:175636912-175637028	+	TAAAGTAATCGTGGGTTTTGC	Yes	Yes	Yes	0	0	0	No	
NM_hsa_1391	chr6:30854247-30854307	-	ACCCCAGAGCTCTGAAGCAG	Yes	No	No	0	0	0	No	
NM_hsa_1392	chr6:30854247-30854307	-	CCCACAATGCACTAGGGCAG	Yes	No	No	0	0	0	No	
NM_hsa_1397	chr1:27856551-27856679	+	TTGGGGTAGGACTTGGGCATCG	Yes	Yes	Yes	0	0	0	No	
NM_hsa_1397	chr1:27856583-27856645	+	TTGGGGTAGGACTTGGGCATCG	Yes	Yes	Yes	0	0	0	No	
NM_hsa_1397	chr1:27856569-27856654	+	TTGGGGTAGGACTTGGGCATCG	Yes	Yes	Yes	0	0	0	No	
NM_hsa_1398	chr1:27856583-27856645	+	ATGCCCAGGCCCCACCCCAGAC	Yes	No	No	0	0	0	No	
NM_hsa_1399	chr9:128003903-128003982	+	GCCCTAGGGGGTCGGAGTAGGTC	Yes	No	No	0	0	0	No	
NM_hsa_14	chr1:149900286-149900341	+	TAGAACACTCTGGCCCCATCTG	Yes	Yes	No	0.0569857	0	0	Yes	
NM_hsa_14	chr1:149900276-149900350	+	TAGAACACTCTGGCCCCATCTG	Yes	Yes	No	0.0569857	0	0	Yes	
NM_hsa_1400	chr9:128003903-128003982	+	CTTCTGACCCCGAGGCATTTCCG	Yes	No	No	0	0	0	No	
NM_hsa_1401	chr22:25509154-25509240	+	CTCTGACCTCCCCATTCACGGCT	Yes	Yes	No	0	0	0	No	
NM_hsa_1401	chr22:25509168-25509231	+	CTCTGACCTCCCCATTCACGGCT	Yes	Yes	No	0	0	0	No	
NM_hsa_1402	chr22:25509168-25509231	+	CACAGTGAGTGAGTGGCAGAGCC	Yes	No	No	0.0569857	0	0	No	
NM_hsa_1403	chr2:114030814-114030897	+	CTGCCTGAGCCGGAGAGGATG	Yes	No	No	0	0	0	No	
NM_hsa_1404	chr2:114030814-114030897	+	TTAGCTCTGGCTTAGCTAGC	Yes	No	No	0	0	0	No	
NM_hsa_1405	chr1:160040786-160040879	+	GTCCCCAGGACTCTGTTGCAT	Yes	Yes	No	0	0	0	No	
NM_hsa_1405	chr1:160040796-160040857	+	GTCCCCAGGACTCTGTTGCAT	Yes	Yes	No	0	0	0	No	
NM_hsa_1406	chr1:160040796-160040857	+	GTAGCAGAGTCCTGGGGAGAC	Yes	No	No	0	0	0	No	
NM_hsa_141	chr14:70449634-70449701	+	CCGCCTCCACCAAGCCTGGAAT	Yes	Yes	No	0	0	0	Yes	
NM_hsa_141	chr14:70449620-70449710	+	CCGCCTCCACCAAGCCTGGAAT	Yes	Yes	No	0	0	0	Yes	
NM_hsa_1411	chr19:41316668-41316765	-	CGGCCGTGCTGGTGGACACTG	Yes	Yes	No	0	0	0	No	
NM_hsa_1411	chr19:41316677-41316730	-	CGGCCGTGCTGGTGGACACTG	Yes	Yes	No	0	0	0	No	
NM_hsa_1412	chr19:41316677-41316730	-	CCGTCCCCACCACAGGAATCTTGT	Yes	No	No	0	0	0	No	
NM_hsa_1413	chr17:61904846-61904939	+	GCTTGACGGACCAGAGCAGG	Yes	Yes	No	0	0	0	No	
NM_hsa_1413	chr17:61904856-61904932	+	GCTTGACGGACCAGAGCAGG	Yes	Yes	No	0	0	0	No	
NM_hsa_1414	chr17:61904856-61904932	+	GGCTGGGTGCGGAGCGAGCGT	Yes	No	No	0	0	0	No	
NM_hsa_1417	chr20:2520536-2520601	+	AGGAGTGGGCTAAGGAAACCTGC	Yes	No	No	0	0	0	No	
NM_hsa_1418	chr20:2520536-2520601	+	AGGTTCTCTTAGCTCACTCCCGC	Yes	No	No	0	0	0	No	
NM_hsa_1419	chr13:99218938-99219023	-	TAGGTGGAAGGACTGAGCCTG	Yes	Yes	No	0.227943	0	0.366431	No	
NM_hsa_1419	chr13:99218947-99219010	-	TAGGTGGAAGGACTGAGCCTG	Yes	Yes	No	0.227943	0	0.366431	No	
NM_hsa_142	chr14:70449634-70449701	+	TCCAAGCCTGAGAGAGGCAGCT	Yes	No	No	0	0	0	Yes	
NM_hsa_1420	chr13:99218947-99219010	-	GAGCTTTCTTCTCCCACCTGCT	Yes	No	No	0	0	0	No	
NM_hsa_1421	chr20:61809865-61809921	-	TCAAGGTCCGCTGTGAACACG	Yes	Yes	No	0	0	0	No	
NM_hsa_1421	chr8:65291729-65291784	-	TCAAGGTCCGCTGTGAACACG	Yes	Yes	No	0	0	0	No	
NM_hsa_1421	chr8:65291720-65291797	-	TCAAGGTCCGCTGTGAACACG	Yes	Yes	No	0	0	0	No	
NM_hsa_1422	chr20:61809865-61809921	-	CATTCACCGCGTGCCTTAATT	Yes	No	No	0	0	0	No	
NM_hsa_1422	chr8:65291729-65291784	-	CATTCACCGCGTGCCTTAATT	Yes	No	No	0	0	0	No	
NM_hsa_1423	chr14:102828025-102828123	-	ATCTGTTACTTTTGCACCAAGC	Yes	Yes	Yes	0	0	0	Yes	
NM_hsa_1423	chr14:102828044-102828113	-	ATCTGTTACTTTTGCACCAAGC	Yes	Yes	Yes	0	0	0	Yes	
NM_hsa_1423	chr14:102828035-102828125	-	ATCTGTTACTTTTGCACCAAGC	Yes	Yes	Yes	0	0	0	Yes	
NM_hsa_1424	chr14:102828044-102828113	-	TTGGTGCAAAAGTAATGGCAATTT	Yes	No	No	0	0	0	No	
NM_hsa_1425	chr12:6980771-6980813	-	GGGCTGTAGAGTGGAGGGC	Yes	No	No	0	0	0	No	
NM_hsa_1426	chr12:6980771-6980813	-	CCTCCTCACCTCAGCCCCA	Yes	No	No	0	0	0	No	
NM_hsa_1427	chr5:40798552-40798623	+	AACGATTAGGCGGACCGT	Yes	No	No	0	0	0.0732862	No	
NM_hsa_1428	chr5:40798552-40798623	+	TGCCCGCTGCGATCTTCC	Yes	No	No	0	0	0	No	
NM_hsa_1429	chr13:111214171-111214266	+	CCAGCCGAGGCTCGGGACTGCTG	Yes	Yes	No	0	0	0	No	
NM_hsa_1429	chr13:111214185-111214257	+	CCAGCCGAGGCTCGGGACTGCTG	Yes	Yes	No	0	0	0	No	
NM_hsa_1430	chr13:111214185-111214257	+	CCCGTCCCGCCCCTGCTGGAC	Yes	No	No	0	0	0	No	
NM_hsa_1431	chr5:175962811-175962880	+	CACGGAGCTCTGTGTGAACGGGT	Yes	Yes	No	0	0	0	No	
NM_hsa_1431	chr5:175962822-175962871	+	CACGGAGCTCTGTGTGAACGGGT	Yes	Yes	No	0	0	0	No	
NM_hsa_1432	chr5:175962822-175962871	+	CCGTCCACATCTCCATGCT	Yes	No	No	0	0	0	No	
NM_hsa_1433	chr7:98617335-98617399	+	ACATCAGAGCGCAGGACTGTGT	Yes	No	No	0	0	0	No	
NM_hsa_1434	chr7:98617335-98617399	+	ACACACTGCATTCTAGTGACT	Yes	No	No	0	0	0	No	
NM_hsa_1435	chr17:41409255-41409387	+	TACTGCCTGAACTACTGGAACATC	Yes	Yes	Yes	0	0	0	Yes	
NM_hsa_1435	chr17:41409280-41409356	+	TACTGCCTGAACTACTGGAACATC	Yes	Yes	Yes	0	0	0	Yes	
NM_hsa_1435	chr17:41409290-41409348	+	TACTGCCTGAACTACTGGAACATC	Yes	Yes	Yes	0	0	0	Yes	
NM_hsa_1436	chr17:41409290-41409348	+	TGTCTCAGCGGCAGGCAGTTGT	Yes	No	No	0	0	0	No	
NM_hsa_1437	chr11:109983414-109983497	+	CCAAGAAGGACTGCAGAGTTGA	Yes	Yes	No	0.113971	0	0	Yes	
NM_hsa_1437	chr11:109983427-109983488	+	CCAAGAAGGACTGCAGAGTTGA	Yes	Yes	No	0.113971	0	0	Yes	
NM_hsa_1438	chr11:109983427-109983488	+	CACTCCCAGTCAGTCCTGGCT	Yes	No	No	0.170957	0	0.0732862	No	
NM_hsa_1441	chr17:75675448-75675519	+	AGCACCTGCCGAGCACTGAGA	Yes	Yes	No	0	0	0	Yes	
NM_hsa_1441	chr17:75675458-75675512	+	AGCACCTGCCGAGCACTGAGA	Yes	Yes	No	0	0	0	Yes	
NM_hsa_1442	chr17:75675458-75675512	+	TCAGTGCACGTTAGCTGTCAT	Yes	No	No	0	0	0	No	
NM_hsa_1443	chr3:160118981-160119039	+	TGTGAATTTTTGTTTGTGACCTG	Yes	No	No	0	0.0496669	0.109929	Yes	H>KapB>KapB+cMyc
NM_hsa_1444	chr3:160118981-160119039	+	GGTCACAAACAAAAATTCAATAT	Yes	No	No	0	0	0	No	
NM_hsa_1445	chr8:58508963-58509033	+	ATGCATTTGTAGCTGTAGAAT	Yes	Yes	No	0	0	0	No	
NM_hsa_1445	chr8:58508975-58509024	+	ATGCATTTGTAGCTGTAGAAT	Yes	Yes	No	0	0	0	No	
NM_hsa_1446	chr8:58508975-58509024	+	ACTACAGCTGCAAATGCATGA	Yes	No	No	0	0	0	No	
NM_hsa_1449	chr6:29601075-29601130	+	CACGCACTCGTCGGGGGG	Yes	No	No	0	0	0	No	
NM_hsa_1450	chr6:29601075-29601130	+	GGCTGAGAGGTGGGGG	Yes	No	No	0	0	0	No	
NM_hsa_1453	chr1:209957811-209957893	-	GGTGCCTGCGGGACTGGAG	Yes	No	No	0	0	0	No	
NM_hsa_1455	chr15:37491014-37491081	+	ATCATATGGCATAGAGCTCTGC	Yes	Yes	No	0	0	0	No	
NM_hsa_1455	chr15:37491001-37491090	+	ATCATATGGCATAGAGCTCTGC	Yes	Yes	No	0	0	0	No	
NM_hsa_1456	chr15:37491014-37491081	+	AGAGCTGTATGCCATATGATTG	Yes	No	No	0	0	0	No	
NM_hsa_1457	chr5:115257216-115257276	+	AGAACTGCCACCAGCTGTAG	Yes	No	No	0	0	0	No	
NM_hsa_1458	chr5:115257216-115257276	+	TCAGCTGCTGGCAGTGACCACTCA	Yes	No	No	0	0	0	No	
NM_hsa_1459	chr13:98085739-98085786	+	CTCTGACTCGCCCCTCTGGCG	Yes	No	No	0.0569857	0	0	No	
NM_hsa_146	chr1:171560222-171560285	+	TAGGAGCTATCAGAACTTAGTGA	Yes	Yes	No	0.0569857	0.397335	0	Yes	
NM_hsa_146	chr1:171560212-171560301	+	TAGGAGCTATCAGAACTTAGTGA	Yes	Yes	No	0.0569857	0.397335	0	Yes	
NM_hsa_1460	chr13:98085739-98085786	+	CTAGAGGGGGGCGTCACCCTC	Yes	No	No	0	0	0	No	
NM_hsa_1461	chr5:129448036-129448176	+	TCTCAGACCGGATTTCCACAGTT	Yes	Yes	Yes	0	0	0	Yes	
NM_hsa_1461	chr5:129448076-129448134	+	TCTCAGACCGGATTTCCACAGTT	Yes	Yes	Yes	0	0	0	Yes	
NM_hsa_1461	chr5:129448066-129448142	+	TCTCAGACCGGATTTCCACAGTT	Yes	Yes	Yes	0	0	0	Yes	
NM_hsa_1462	chr5:129448076-129448134	+	CCTGGAGTCTGGCCCGAGAAT	Yes	No	No	0	0.0662225	0.0732862	Yes	H>KapB>KapB+cMyc
NM_hsa_1463	chr4:144105766-144105831	-	TGGCTGCGACTGTAGGAT	Yes	No	No	0	0	0	No	
NM_hsa_1464	chr4:144105766-144105831	-	CACAGGCGGTCTTC	Yes	No	No	0	0	0	No	
NM_hsa_1465	chr1:21933564-21933614	-	ACAGGTCACACAGCTGGAG	Yes	No	No	0	0	0	No	
NM_hsa_1466	chr1:21933564-21933614	-	CCGGCTGGTCACACCTCACC	Yes	No	No	0	0	0	No	
NM_hsa_1467	chr16:11024208-11024287	+	CTGGGCACTGAGCATTTAGCAG	Yes	Yes	Yes	0	0	0	No	
NM_hsa_1467	chr16:11024178-11024316	+	CTGGGCACTGAGCATTTAGCAG	Yes	Yes	Yes	0	0	0	No	
NM_hsa_1467	chr16:11024218-11024281	+	CTGGGCACTGAGCATTTAGCAG	Yes	Yes	Yes	0	0	0	No	
NM_hsa_1468	chr16:11024218-11024281	+	ACTAAATGCCAGTAGCACCCCTG	Yes	No	No	0	0	0	No	
NM_hsa_1469	chr3:9885800-9885871	+	TTTTAGGGAGAGCGCTGTAG	Yes	No	No	0	0	0.0732862	No	
NM_hsa_147	chr1:171560222-171560285	+	ACTAATTTCTGATGCTCTTCCC	Yes	No	No	0	0	0	No	
NM_hsa_1470	chr3:9885800-9885871	+	GCGGGGCTTTTCCTCCGCCC	Yes	No	No	0	0	0	No	
NM_hsa_1473	chr3:28282843-28282906	-	CGGACCTGCAGGACTTTGG	Yes	No	No	0	0	0	No	
NM_hsa_1473	chr3:28282887-28282932	-	CGGACCTGCAGGACTTTGG	Yes	No	No	0	0	0	No	
NM_hsa_1474	chr3:28282887-28282932	-	AGACCCGGGTCGGCC	Yes	No	No	0	0	0	No	
NM_hsa_1477	chr1:108962691-108962747	-	AGTTAGGTCTGAAGACTGTAA	Yes	Yes	No	0	0	0	No	
NM_hsa_1477	chr1:108962682-108962759	-	AGTTAGGTCTGAAGACTGTAA	Yes	Yes	No	0	0	0	No	
NM_hsa_1477	chr1:108816877-108816933	+	AGTTAGGTCTGAAGACTGTAA	Yes	Yes	No	0	0	0	No	
NM_hsa_1478	chr1:108962691-108962747	-	GCAGTCTTCAGATATAACTCC	Yes	No	No	0	0	0	No	
NM_hsa_1478	chr1:108816877-108816933	+	GCAGTCTTCAGATATAACTCC	Yes	No	No	0	0	0	No	
NM_hsa_1479	chr17:20841711-20841788	+	ATAATAGTCTCCAACTCTGATT	Yes	Yes	No	0	0.0331113	0	Yes	
NM_hsa_1479	chr17:20841721-20841781	+	ATAATAGTCTCCAACTCTGATT	Yes	Yes	No	0	0.0331113	0	Yes	
NM_hsa_1480	chr17:20841721-20841781	+	TCAGAGATGGAAATTATTATCC	Yes	No	No	0	0	0	No	
NM_hsa_1481	chr7:45144573-45144642	-	CTGCAGTCAAGTCAAATTCA	Yes	No	No	0	0	0	Yes	
NM_hsa_1483	chr11:68890975-68891030	+	AACCCAGAACTTTGTGACCTTGC	Yes	Yes	Yes	0	0	0	No	
NM_hsa_1483	chr11:68890949-68891049	+	AACCCAGAACTTTGTGACCTTGC	Yes	Yes	Yes	0	0	0	No	
NM_hsa_1483	chr11:68890948-68891039	+	AACCCAGAACTTTGTGACCTTGC	Yes	Yes	Yes	0	0	0	No	
NM_hsa_1484	chr11:68890975-68891030	+	AGGGTCACATAGTGGGGCCCA	Yes	No	No	0	0	0	No	
NM_hsa_1485	chr5:180688373-180688449	+	CGGCTCTGATCAGGTCAGCGG	Yes	No	No	0	0	0	No	
NM_hsa_1486	chr5:180688373-180688449	+	GGTGGTTTGGTGGGTTGGCTGGTT	Yes	No	No	0	0	0	No	
NM_hsa_1489	chr9:90234688-90234753	+	GCTGATTGTCGTTGTCATCTGG	Yes	Yes	Yes	0	0	0	Yes	
NM_hsa_1489	chr9:90234666-90234762	+	GCTGATTGTCGTTGTCATCTGG	Yes	Yes	Yes	0	0	0	Yes	
NM_hsa_1489	chr9:90234664-90234782	+	GCTGATTGTCGTTGTCATCTGG	Yes	Yes	Yes	0	0	0	Yes	
NM_hsa_1490	chr9:90234688-90234753	+	AGAGGCCAATGACAGTCAGAGG	Yes	No	No	0	0.0662225	0	No	
NM_hsa_1492	chr8:144218018-144218098	-	CATGGTAAGGACTGAGGCTAC	Yes	No	No	0	0	0.146572	No	
NM_hsa_1493	chr8:144218018-144218098	-	TGCCGTGTGACTTTATGGACGAA	Yes	No	No	0	0	0	No	
NM_hsa_1494	chr14:24845414-24845486	+	TCACCAGCATGTCCTCCCACT	Yes	No	Yes	0	0	0	Yes	
NM_hsa_1494	chr14:24845404-24845510	+	TCACCAGCATGTCCTCCCACT	Yes	No	Yes	0	0	0	Yes	
NM_hsa_1495	chr14:24845414-24845486	+	TGGGAGGAGCAGGCAGCTGGAAGGT	Yes	No	No	0	0	0	No	
NM_hsa_1496	chr3:133467592-133467652	+	TTTTGCTTTCCAGACACAGTAG	Yes	Yes	No	0	0	0	No	

Additional file 5

Name	locus	strand	sequence	mirdeep2	mireap	miranalyzer	RPM_HU_KapB_cMyc	RPM_HU_KaposinB	RPM_HUVEC	IP_confidence	annotation
NM_hsa_1496	chr3:133467577-133467661	+	TTTTGCTTTCCAGACACAGTAG	Yes	Yes	No	0	0	0	No	
NM_hsa_1497	chr3:133467592-133467652	+	AATGTGTCTGGAAAGCAAAAGT	Yes	No	No	0	0	0	No	
NM_hsa_15	chr14:101506177-101506255	+	AGTCGGTGGACCTCAGAACATGT	Yes	Yes	No	0.170957	0	0	No	
NM_hsa_15	chr14:101506187-101506246	+	AGTCGGTGGACCTCAGAACATGT	Yes	Yes	No	0.170957	0	0	No	
NM_hsa_1500	chr7:1731872-1731930	+	ATTGCCAGAGGGTTCTGTGAGT	Yes	Yes	No	0	0	0	No	
NM_hsa_1500	chr7:1731898-1731984	+	ATTGCCAGAGGGTTCTGTGAGT	Yes	Yes	No	0	0	0	No	
NM_hsa_1501	chr7:1731872-1731930	+	TGCACAGATCTGTCTGGCCTGCC	Yes	No	No	0	0	0	No	
NM_hsa_1502	chr17:33760236-33760295	+	CACACTTCCCCAGCGTGGCCTGC	Yes	No	No	0.0569857	0.463558	0	Yes	
NM_hsa_1503	chr17:33760236-33760295	+	AGCTGGCGCTCCGGAAGTGTCCG	Yes	No	No	0	0	0	No	
NM_hsa_1504	chr16:78068162-78068233	+	ACTGACATCTTCAGGAAGGTAG	Yes	Yes	No	0	0	0	No	
NM_hsa_1504	chr16:78068172-78068225	+	ACTGACATCTTCAGGAAGGTAG	Yes	Yes	No	0	0	0	No	
NM_hsa_1505	chr16:78068172-78068225	+	GTCTTCCCAAAGATGTCAGTTA	Yes	No	No	0	0	0	No	
NM_hsa_1507	chr17:41363861-41363932	+	GAGTCAAAGCTTGAGG	Yes	No	No	0	0	0	No	
NM_hsa_1508	chr20:37053929-37053969	-	GAAAGTGATCGTGGGCTGC	Yes	No	No	0	0	0	No	
NM_hsa_1509	chr20:37053929-37053969	-	TGCCCTGGTCATTGATAG	Yes	No	No	0	0	0	No	
NM_hsa_1510	chr14:36278428-36278491	-	CTCTGCAGGGTGTAAGCTGC	Yes	Yes	No	0	0	0.0732862	No	
NM_hsa_1510	chr14:36278435-36278481	-	CTCTGCAGGGTGTAAGCTGC	Yes	Yes	No	0	0	0.0732862	No	
NM_hsa_1511	chr14:36278435-36278481	-	AGCTTCAACCTTAAGGGGGC	Yes	No	No	0	0	0	No	
NM_hsa_1512	chr2:112109376-112109459	+	ATCCAGGTAACAGGTGTGTTAGA	Yes	Yes	Yes	0	0	0	No	
NM_hsa_1512	chr2:112109390-112109450	+	ATCCAGGTAACAGGTGTGTTAGA	Yes	Yes	Yes	0	0	0	No	
NM_hsa_1512	chr2:112109369-112109469	+	ATCCAGGTAACAGGTGTGTTAGA	Yes	Yes	Yes	0	0	0	No	
NM_hsa_1513	chr2:112109390-112109450	+	TGGCAGATCTGGTACCTGGATAA	Yes	No	No	0	0	0	No	
NM_hsa_1516	chr5:167517512-167517566	+	TGTCTCATGCATGGTACTCTCT	Yes	Yes	No	0	0	0	Yes	
NM_hsa_1516	chr5:167517499-167517575	+	TGTCTCATGCATGGTACTCTCT	Yes	Yes	No	0	0	0	Yes	
NM_hsa_1517	chr5:167517512-167517566	+	AAGGTACCAGGTATGGGACCAC	Yes	No	No	0	0	0	No	
NM_hsa_1518	chr1:144984098-144984167	-	ATTCTGTAGGCATGGACTG	Yes	No	No	0	0	0	No	
NM_hsa_1519	chr1:144984098-144984167	-	TACCAGGTAGCCGGAGTTA	Yes	No	No	0	0	0	No	
NM_hsa_152	chr9:116310598-116310649	+	CACTGTGTTGGGCTGTAATGGA	Yes	No	No	0	0.0662225	0	No	
NM_hsa_1520	chr1:18073682-18073757	+	TTAGGGTTTGCCTGGGACTGA	Yes	No	No	102.717	70.6594	40.3441	Yes	KapB+cMyc>KapB>H
NM_hsa_1521	chr1:18073682-18073757	+	AAAACCAGGCAAACCAAGATGAAT	Yes	No	No	0	0	0	No	
NM_hsa_1526	chr3:184454966-184455062	+	TTCCAGGTCAAAGGCTGCAGGAG	Yes	Yes	No	0	0	0	No	
NM_hsa_1526	chr3:184455030-184455093	+	TTCCAGGTCAAAGGCTGCAGGAG	Yes	Yes	No	0	0	0	No	
NM_hsa_1527	chr3:184455030-184455093	+	CCTCCTCAGCAAGGGATCTGCAAC	Yes	No	No	0	0	0	No	
NM_hsa_1528	chr9:84706196-84706257	+	CAGTGAGCCAGGTCAGGGTTAGG	Yes	Yes	No	0	0	0	No	
NM_hsa_1528	chr9:84706186-84706264	+	CAGTGAGCCAGGTCAGGGTTAGG	Yes	Yes	No	0	0	0	No	
NM_hsa_1529	chr9:84706196-84706257	+	CAGCCCTCAATGAGCTCACAGTC	Yes	No	No	0	0	0	No	
NM_hsa_153	chr9:116310598-116310649	+	GAAAGCAGCTCAGCTAGTGGC	Yes	No	No	0	0	0	No	
NM_hsa_1530	chr12:123193895-123193957	-	CCCAGAAATCTGGTATGCCAGC	Yes	No	Yes	0	0	0	No	
NM_hsa_1530	chr12:123193874-123193982	-	CCCAGAAATCTGGTATGCCAGC	Yes	No	Yes	0	0	0	No	
NM_hsa_1530	chr12:123180470-123180532	-	CCCAGAAATCTGGTATGCCAGC	Yes	No	Yes	0	0	0	No	
NM_hsa_1530	chr12:123180449-123180557	-	CCCAGAAATCTGGTATGCCAGC	Yes	No	Yes	0	0	0	No	
NM_hsa_1531	chr12:123193895-123193957	-	TGACAAGCCAGGCTTCTGGTCT	Yes	No	No	0	0	0	No	
NM_hsa_1531	chr12:123180470-123180532	-	TGACAAGCCAGGCTTCTGGTCT	Yes	No	No	0	0	0	No	
NM_hsa_1532	chr2:69402666-69402750	+	AGCCCAGAAGCTGGAAACTGCAG	Yes	Yes	No	0	0	0	Yes	
NM_hsa_1534	chrX:36124372-36124449	+	AGATTGCTCTTTGACTAATTAG	Yes	Yes	No	0	0	0	No	
NM_hsa_1534	chrX:36124382-36124441	+	AGATTGCTCTTTGACTAATTAG	Yes	Yes	No	0	0	0	No	
NM_hsa_1535	chrX:36124382-36124441	+	AATTAGCCAAGGAACAATCTTG	Yes	No	No	0	0	0	No	
NM_hsa_1536	chr5:156924088-156924168	-	TCCTCTCATTCCTTGGCTCAG	Yes	Yes	No	0	0	0	No	
NM_hsa_1536	chr5:156924097-156924151	-	TCCTCTCATTCCTTGGCTCAG	Yes	Yes	No	0	0	0	No	
NM_hsa_1537	chr5:156924097-156924151	-	GGGTTGAGGGTGGGGAGGAGA	Yes	No	No	0	0	0	No	
NM_hsa_1538	chr3:105289190-105289244	+	AAATTGACATTTGGGAACTGA	Yes	No	No	0	0	0	Yes	
NM_hsa_1539	chr3:105289190-105289244	+	AGTTCCAAAATGTCAATTTGA	Yes	No	No	0	0	0	Yes	
NM_hsa_154	chr14:19318990-19319070	-	TTCTGGAATACTGTGTGAGGG	Yes	No	No	0.0854786	0	0	Yes	
NM_hsa_1540	chr13:110218012-110218076	-	TGAGACTCCTCTTCCCCTCAGA	Yes	No	No	0	0	0	No	
NM_hsa_1541	chr13:110218012-110218076	-	TGAGGAGAGGGCAGCAGG	Yes	No	No	0	0	0	No	
NM_hsa_1542	chr6:46030719-46030810	+	TAAAGACTTTGAAAAAGACAGC	Yes	Yes	No	0	0	0	No	
NM_hsa_1542	chr6:46030731-46030801	+	TAAAGACTTTGAAAAAGACAGC	Yes	Yes	No	0	0	0	No	
NM_hsa_1543	chr6:46030731-46030801	+	TGCCTTTTGCAAAGTCTTTATT	Yes	No	No	0	0	0	No	
NM_hsa_1544	chr5:114753411-114753498	+	TGGGACTGGAACACATATATAT	Yes	Yes	No	0	0	0	No	
NM_hsa_1544	chr5:114753421-114753491	+	TGGGACTGGAACACATATATAT	Yes	Yes	No	0	0	0	No	
NM_hsa_1545	chr5:114753421-114753491	+	ATACATGTGCTCCAGTCCCCAT	Yes	No	No	0	0	0	No	
NM_hsa_1548	chr7:50570219-50570283	+	CTGCAGTCCTGAGTCAGCCAGT	Yes	Yes	No	0	0	0	No	
NM_hsa_1548	chr7:50570207-50570292	+	CTGCAGTCCTGAGTCAGCCAGT	Yes	Yes	No	0	0	0	No	
NM_hsa_1549	chr7:50570219-50570283	+	TGAGCTGATGAAGGTTTGCAGCT	Yes	No	No	0	0	0	No	
NM_hsa_155	chr14:19318990-19319070	-	CAAAAGCAGTGTTCTAGAATC	Yes	No	No	0	0	0	No	
NM_hsa_1552	chr15:91416192-91416261	-	ACTCTGTTCCAGGCTGTGGC	Yes	No	No	0	0	0	No	
NM_hsa_1553	chr15:91416192-91416261	-	TGTGTTCTTGGGCAAAGCCC	Yes	No	No	0	0	0	No	
NM_hsa_1554	chr1:229695009-229695070	+	CAGGGCTGAGGTGCGGAGGGA	Yes	No	No	0.0569857	0	0	No	
NM_hsa_1555	chr1:229695009-229695070	+	GCTGCGTGTCAGCTCAGCTCTGGA	Yes	No	No	0	0	0	No	
NM_hsa_1556	chr5:135314906-135314960	-	TACACCAGATAAAAAGCATCA	Yes	Yes	No	0	0	0	No	
NM_hsa_1556	chr5:135314897-135314972	-	TACACCAGATAAAAAGCATCA	Yes	Yes	No	0	0	0	No	
NM_hsa_1557	chr5:135314906-135314960	-	ATGCCTTTTATCTGGTGTATT	Yes	No	No	0	0	0	No	
NM_hsa_1558	chr14:70075954-70076005	-	AAAGTGCCACAGAAAAGTATT	Yes	No	No	0	0	0	No	
NM_hsa_1559	chr4:160229033-160229118	+	AGCCCCAGAAGTTGTCAGAGCAG	Yes	Yes	No	0	0.198668	0	Yes	
NM_hsa_1559	chr4:160229043-160229108	+	AGCCCCAGAAGTTGTCAGAGCAG	Yes	Yes	No	0	0.198668	0	Yes	
NM_hsa_1560	chr4:160229043-160229108	+	GCACTCCAGCCTATGGGGCAGA	Yes	No	No	0	0	0	No	
NM_hsa_1561	chr12:101694587-101694655	+	ACTGTACTGGATTGTGGCGACAT	Yes	Yes	No	0	0	0	No	
NM_hsa_1561	chr12:101694574-101694664	+	ACTGTACTGGATTGTGGCGACAT	Yes	Yes	No	0	0	0	No	
NM_hsa_1562	chr12:101694587-101694655	+	CTCGCCGCATGTCAAGTGCTCTGTAG	Yes	No	No	0	0	0	No	
NM_hsa_1563	chr14:69952747-69952808	+	TGGTATGGCCTGGAGAATGCAGA	Yes	Yes	Yes	0	0	0	No	
NM_hsa_1563	chr14:69952736-69952817	+	TGGTATGGCCTGGAGAATGCAGA	Yes	Yes	Yes	0	0	0	No	
NM_hsa_1563	chr14:69952712-69952842	+	TGGTATGGCCTGGAGAATGCAGA	Yes	Yes	Yes	0	0	0	No	
NM_hsa_1564	chr14:69952747-69952808	+	TCCACTCTCCAGTCAATTTCAGT	Yes	No	No	0	0	0	Yes	
NM_hsa_1565	chr9:112857459-112857512	+	TAGGAACAAGGGACTGCCAGA	Yes	No	No	0	0	0	No	
NM_hsa_1566	chr9:112857459-112857512	+	CAGCAGTTCCCTTGTGCCCTGGG	Yes	No	No	0	0	0	No	
NM_hsa_1567	chr5:140048155-140048197	+	GTTCTACATCTGTGTCTCTAG	Yes	No	No	0.113971	0.198668	0	No	
NM_hsa_1568	chr5:140048155-140048197	+	GGGACAGAGATGCTCCAAGA	Yes	No	No	0	0	0	No	
NM_hsa_157	chr14:94582742-94582805	+	GCATGTCCCACTCTGTCCACCCT	Yes	No	No	0	0	0	No	
NM_hsa_1571	chr3:167452996-167453075	+	GGGCTGTCGGGCTGAGTATG	Yes	No	No	0	0	0	No	
NM_hsa_1572	chr3:167452996-167453075	+	GGGGCGGGCTGACAGAGCAGA	Yes	No	No	0	0	0	No	
NM_hsa_1573	chr17:38478846-38478929	-	GAAGGAGGGCTGAGGACCTG	Yes	No	No	0	0	0	No	
NM_hsa_1574	chr17:38478846-38478929	-	GCTTCCCCTTCTCAAG	Yes	No	No	0	0	0	No	
NM_hsa_1575	chr16:15781178-15781233	+	CTCTGCTTTTCTCTTCGCCAGC	Yes	No	No	0	0	0	No	
NM_hsa_1576	chr16:15781178-15781233	+	TGGAGGGATGCTGCGGTTTG	Yes	No	No	0	0	0	No	
NM_hsa_1579	chr16:18189384-18189522	-	GCCCCTGAGAGTGAGTGCCACC	Yes	Yes	Yes	0	0	0	No	
NM_hsa_1579	chr16:18189414-18189492	-	GCCCCTGAGAGTGAGTGCCACC	Yes	Yes	Yes	0	0	0	No	
NM_hsa_1579	chr16:18189423-18189480	-	GCCCCTGAGAGTGAGTGCCACC	Yes	Yes	Yes	0	0	0	No	
NM_hsa_1579	chr16:15277821-15277878	-	GCCCCTGAGAGTGAGTGCCACC	Yes	Yes	Yes	0	0	0	No	
NM_hsa_1579	chr16:15277782-15277920	-	GCCCCTGAGAGTGAGTGCCACC	Yes	Yes	Yes	0	0	0	No	
NM_hsa_1580	chr16:18189423-18189480	-	AAGTACCCATTCTTAGGGTCAT	Yes	No	No	0	0	0	No	
NM_hsa_1580	chr16:15277821-15277878	-	AAGTACCCATTCTTAGGGTCAT	Yes	No	No	0	0	0	No	
NM_hsa_1581	chr19:23441560-23441638	-	CTGCCTGGGCCTGGACCACAGA	Yes	Yes	No	0	0	0	No	
NM_hsa_1581	chr19:23441569-23441626	-	CTGCCTGGGCCTGGACCACAGA	Yes	Yes	No	0	0	0	No	
NM_hsa_1582	chr19:23441569-23441626	-	TGTGGCTGGCCCAGGTACCA	Yes	No	No	0	0	0	No	
NM_hsa_1585	chr12:186290-186420	-	TCAAGACTGTCGGAGATGCCAGA	Yes	No	Yes	0	0	0	No	
NM_hsa_1585	chr12:186324-186387	-	TCAAGACTGTCGGAGATGCCAGA	Yes	No	Yes	0	0	0	No	
NM_hsa_1586	chr12:186324-186387	-	TGGCCTTCTCTGATGGGTTCTTACCATG	Yes	No	No	0	0	0	No	
NM_hsa_1587	chr1:156717330-156717421	+	TCCTAGGCCTTTCTTCTGTGG	Yes	Yes	No	0	0	0	Yes	
NM_hsa_1587	chr1:156717353-156717412	+	TCCTAGGCCTTTCTTCTGTGG	Yes	Yes	No	0	0	0	Yes	
NM_hsa_1588	chr1:156717353-156717412	+	CCAGGAGACAGGCATAGGACA	Yes	No	No	0	0	0	No	
NM_hsa_1589	chr17:18935738-18935802	+	TGAGCAAACTCACAAGGCTGG	Yes	Yes	No	0	0	0	Yes	
NM_hsa_1589	chr17:19045370-19045434	-	TGAGCAAACTCACAAGGCTGG	Yes	Yes	No	0	0	0	Yes	
NM_hsa_1589	chr17:19045361-19045448	-	TGAGCAAACTCACAAGGCTGG	Yes	Yes	No	0	0	0	Yes	
NM_hsa_1590	chr17:18935738-18935802	+	TGCCTTGTGGTTTTCACACAGC	Yes	No	No	0	0	0	Yes	
NM_hsa_1590	chr17:19045370-19045434	-	TGCCTTGTGGTTTTCACACAGC	Yes	No	No	0	0	0	Yes	
NM_hsa_1591	chr3:1361024-1361160	+	ACAGGTACTGTGAGTCTGCAT	Yes	Yes	Yes	0	0	0	No	
NM_hsa_1591	chr3:1361055-1361130	+	ACAGGTACTGTGAGTCTGCAT	Yes	Yes	Yes	0	0	0	No	
NM_hsa_1591	chr3:1361068-1361121	+	ACAGGTACTGTGAGTCTGCAT	Yes	Yes	Yes	0	0	0	No	
NM_hsa_1592	chr3:1361068-1361121	+	ACAGATACATAGTACTTGGTAC	Yes	No	No	0	0	0	No	
NM_hsa_1593	chr9:126083417-126083490	+	TGAGATTCTTGGACCCGTGCT	Yes	Yes	Yes	0	0	0	No	
NM_hsa_1593	chr9:126083402-126083508	+	TGAGATTCTTGGACCCGTGCT	Yes	Yes	Yes	0	0	0	No	
NM_hsa_1593	chr9:126083427-126083483	+	TGAGATTCTTGGACCCGTGCT	Yes	Yes	Yes	0	0	0	No	
NM_hsa_1594	chr9:126083427-126083483	+	CATGGGTTCAAGAATCTGCAGC	Yes	No	No	0	0	0	No	
NM_hsa_1597	chr10:119407434-119407496	+	CAGGAATAGGACTCAGCCCAGG	Yes	No	No	0	0	0	No	
NM_hsa_1598	chr10:119407434-119407496	+	TGGGTTTATTTCTATTCCAACA	Yes	No	No	0	0	0	No	
NM_hsa_1599	chr4:106816228-106816269	+	CTGTTCGGGCGGAACCTG	Yes	No	No	0	0	0	No	
NM_hsa_16	chr14:101506177-101506255	+	TATGTCTGCGGACCACCCACC	Yes	Yes	Yes	0.512871	0.52978	0.366431	Yes	
NM_hsa_16	chr14:101506169-101506265	+	TATGTCTGCGGACCACCCACC	Yes	Yes	Yes	0.512871	0.52978	0.366431	Yes	
NM_hsa_16	chr14:101506187-101506246	+	TATGTCTGCGGACCACCCACC	Yes	Yes	Yes	0.512871	0.52978	0.366431	Yes	
NM_hsa_160	chr17:44215945-44215993	-	GTGAAATGTTTGGGACCAGA	Yes	No	No	0.0284929	0	0	Yes	
NM_hsa_1600	chr4:106816228-106816269	+	CTGCTCGCCCCTGCAGTC	Yes	No	No	0	0	0	No	
NM_hsa_1601	chr20:17946237-17946301	+	TCCCCAGGTCGAGGCTGCAGTGA	Yes	No	No	0.0854786	0	0.0732862	No	
NM_hsa_1602	chr20:17946237-17946301	+	ACTGCACTCCCGCCTGGGTGACAGAGC	Yes	No	No	0.0284929	0.165556	0.0732862	Yes	
NM_hsa_1605	chr3:164641627-164641689	-	AATCCAGGTGGCGGAGCTTGC	Yes	No	No	0.0569857	0	0	No	
NM_hsa_1607	chr12:87138845-87138923	-	TTTCCTCTCTGCCCCATAGG	Yes	Yes	No	0	0	0	Yes	
NM_hsa_1607	chr12:87138854-87138913	-	TTTCCTCTCTGCCCCATAGG	Yes	Yes	No	0	0	0	Yes	
NM_hsa_1608	chr12:87138854-87138913	-	TAGGGAAGAGAAGGTTGGG	Yes	No	No	0	0	0	No	
NM_hsa_1609	chr8:144337360-144337401	+	CAGGGGCTGTGGACTTGACC	Yes	No	No	0	0	0	No	
NM_hsa_161	chr17:44215945-44215993	-	TGGATTTTGGAATCTTTGCAT	Yes	No	No	0	0	0	No	
NM_hsa_1610	chr8:144337360-144337401	+	TCAAGAGCATAGCTCTCAAG	Yes	No	No	0	0	0	No	
NM_hsa_1614	chr13:86929489-86929567	+	TACAGACGGCTCGTTTGGATCTG	Yes	No	No	0	0	0	No	
NM_hsa_1617	chr7:63811988-63812051	+	AATTAGGCCCAACAGTTCCCGT	Yes	Yes	No	0	0	0.0732862	Yes	
NM_hsa_1617	chr7:63811978-63812060	+	AATTAGGCCCAACAGTTCCCGT	Yes	Yes	No	0	0	0.0732862	Yes	
NM_hsa_1618	chr7:63811988-63812051	+	GGGAGACAGTTGAGCCTAGAGAA	Yes	No	No	0	0	0	Yes	
NM_hsa_1619	chr19:56617806-56617848	-	CGCGGGCGGGCTGGGGCCTG	Yes	No	No	0	0	0	No	
NM_hsa_1620	chr19:56617806-56617848	-	GGCTTCTCCTTCCCAGAG	Yes	No	No	0	0	0	No	
NM_hsa_1621	chr12:97983686-97983744	+	CCCAAACTGCAATTACTTGTGT	Yes	No	No	0	0	0	Yes	
NM_hsa_1622	chr12:97983686-97983744	+	AAATGTAATTGTGGTTTTTGCC	Yes	No	No	0.0262739	0.026016	0.133137	Yes	
NM_hsa_1623	chr2:147743493-147743553	+	CATCCTTGCTTCTTCCTTCCAGG	Yes	No	No	0	0	0	Yes	
NM_hsa_1624	chr2:147743493-147743553	+	TAGGAGGGGGAAGCTCCAAGGACAAC	Yes	No	No	0	0	0	No	
NM_hsa_1625	chr22:37545981-37546033	-	GAAGTGGGCTGGCTCTGGGCT	Yes	No	No	0	0	0	No	
NM_hsa_1626	chr22:37545981-37546033	-	CCCAGGCCTTGCCCCTCTATCCT	Yes	No	No	0	0	0	No	
NM_hsa_1627	chr7:157040840-157040904	+	TGTTCCAGAGTTCTCTGCATGC	Yes	Yes	No	0	0	0	No	
NM_hsa_1627	chr7:157040830-157040911	+	TGTTCCAGAGTTCTCTGCATGC	Yes	Yes	No	0	0	0	No	
NM_hsa_1628	chr7:157040840-157040904	+	CTGCACGGGATCCTGGAGCACA	Yes	No	No	0	0	0	No	
NM_hsa_1635	chr20:48767788-48767867	+	AGGCTGTCTGCAGACTCTGA	Yes	Yes	No	0	0	0	No	
NM_hsa_1635	chr20:48767798-48767858	+	AGGCTGTCTGCAGACTCTGA	Yes	Yes	No	0	0	0	No	
NM_hsa_1636	chr20:48767798-48767858	+	AGAGAGTGAAGGGCACTTGG	Yes	No	No	0	0	0	No	

Additional file 5

Name	locus	strand	sequence	mirdeep2	mireap	miranalyzer	RPM_HU_KapB_cMyc	RPM_HU_KaposinB	RPM_HUVEC	IP_confidence	annotation
NM_hsa_1637	chr10:112064848-112064899	+	TCGCCAGGCGCGGAATGCAGG	Yes	No	No	0	0	0	No	
NM_hsa_1638	chr10:112064848-112064899	+	TGGAGTTCCGGCTTGGAATTT	Yes	No	No	0	0	0	No	
NM_hsa_1639	chr1:231400555-231400615	+	TCATGTTGCTCTGCTGTTGC	Yes	Yes	No	0	0	0	Yes	
NM_hsa_1639	chr1:231400543-231400624	+	TCATGTTGCTCTGCTGTTGC	Yes	Yes	No	0	0	0	Yes	
NM_hsa_1640	chr1:231400555-231400615	+	AGCAGCATGGCTGACTAAAGACA	Yes	No	No	0	0	0	No	
NM_hsa_1647	chr8:92262508-92262591	+	AAGAGAATCCTGTGTGTCCTGA	Yes	Yes	No	0	0	0	No	
NM_hsa_1647	chr8:92262520-92262582	+	AAGAGAATCCTGTGTGTCCTGA	Yes	Yes	No	0	0	0	No	
NM_hsa_1648	chr8:92262520-92262582	+	TGTACATAAAGGAGGCTCTTTG	Yes	No	No	0	0	0	No	
NM_hsa_1649	chr3:160118979-160119035	-	TTGAATTTTTGTTTGTGACCTG	Yes	Yes	No	0	0.0496669	0.0732862	No	H>KapB>KapB+cMyc
NM_hsa_1649	chr3:160118972-160119045	-	TTGAATTTTTGTTTGTGACCTG	Yes	Yes	No	0	0.0496669	0.0732862	No	H>KapB>KapB+cMyc
NM_hsa_1650	chr3:160118979-160119035	-	GGTCACAAACAAAAATTCACAA	Yes	No	No	0	0	0	No	
NM_hsa_1651	chrX:28036667-28036793	+	TATCCTTTTCTAGGGCTGAAC	Yes	Yes	Yes	0	0	0	No	
NM_hsa_1651	chrX:28036692-28036769	+	TATCCTTTTCTAGGGCTGAAC	Yes	Yes	Yes	0	0	0	No	
NM_hsa_1651	chrX:28036702-28036761	+	TATCCTTTTCTAGGGCTGAAC	Yes	Yes	Yes	0	0	0	No	
NM_hsa_1652	chrX:28036702-28036761	+	TCAGACTTAAAAAAGGATACT	Yes	No	No	0	0	0	No	
NM_hsa_1659	chr16:29923370-29923425	-	CACCTTCCGCTTCTTCCCCAG	Yes	No	No	0	0	0	Yes	
NM_hsa_166	chr6:53141798-53141859	-	CGTGATAACTGCTGGGCTGTG	Yes	Yes	No	0	0	0	No	
NM_hsa_166	chr6:53141789-53141871	-	CGTGATAACTGCTGGGCTGTG	Yes	Yes	No	0	0	0	No	
NM_hsa_1660	chr16:29923370-29923425	-	GGGGGAGTACGTGGCCTTTGGT	Yes	No	No	0	0	0	No	
NM_hsa_1661	chr14:23447386-23447459	+	GGGATCCAACAGCTGCTCTGT	Yes	Yes	No	0	0	0	No	
NM_hsa_1661	chr14:23447398-23447450	+	GGGATCCAACAGCTGCTCTGT	Yes	Yes	No	0	0	0	No	
NM_hsa_1662	chr14:23447398-23447450	+	AGAGCAGCTGCGGACCTCCA	Yes	No	No	0	0	0.0732862	No	
NM_hsa_1663	chr19:35652401-35652451	+	TCTCATTGGTTCAGCTGGAGT	Yes	No	No	0	0	0	No	
NM_hsa_1664	chrX:87333961-87334013	-	TCTGGACTCTGAATCTAGTA	Yes	No	No	0.170957	0.176593	0.146572	Yes	
NM_hsa_1665	chrX:87333961-87334013	-	CTAGAGTTAGAGATGGAGC	Yes	No	No	0	0	0	No	
NM_hsa_1666	chr9:119179666-119179733	-	CTTGGGACTCTGTGTTTTTGC	Yes	No	No	0	0	0	No	
NM_hsa_1667	chr9:119179666-119179733	-	AGAAATACAGATTCCTGGGCC	Yes	No	No	0	0	0	No	
NM_hsa_1668	chrX:24498103-24498180	+	CCCGCCTGATCGTCTTACCACCT	Yes	Yes	No	0	0	0	No	
NM_hsa_1668	chrX:24498113-24498173	+	CCCGCCTGATCGTCTTACCACCT	Yes	Yes	No	0	0	0	No	
NM_hsa_1669	chrX:24498113-24498173	+	GTAGGTGAGGGATTGGGCGCA	Yes	No	No	0	0	0	No	
NM_hsa_167	chr6:53141798-53141859	-	CAGCCCTGCAGTTATCACGGG	Yes	No	No	0	0	0	No	
NM_hsa_1670	chr16:31711876-31711952	+	GCGCTTCCCTGCTTGATCTG	Yes	No	No	0	0	0	No	
NM_hsa_1671	chr16:31711876-31711952	+	GCCGAAAAAGGGGGCGCAC	Yes	No	No	0	0	0	No	
NM_hsa_1672	chr20:48925131-48925196	-	CTGTCCTGAAAACAAGCAG	Yes	No	No	0	0	0	No	
NM_hsa_1673	chr20:48925131-48925196	-	GCAGGGTGGTCAGGAAGAGGGA	Yes	No	No	0	0	0	No	
NM_hsa_1674	chrX:14485447-14485505	+	AATGCAGCCTGAGTAGTACT	Yes	Yes	No	0	0	0	No	
NM_hsa_1674	chrX:14485437-14485512	+	AATGCAGCCTGAGTAGTACT	Yes	Yes	No	0	0	0	No	
NM_hsa_1675	chrX:14485447-14485505	+	TACTATGAGGTTCTGCATTTC	Yes	No	No	0	0	0	No	
NM_hsa_1676	chr10:124169916-124169961	+	TGATTTTCATTGGACAGTTGG	Yes	No	No	0	0	0	No	
NM_hsa_1677	chr10:124169916-124169961	+	AACTGCCCAGTGAGATCACT	Yes	No	No	0	0	0	No	
NM_hsa_1678	chr3:50293915-50293974	+	TCCTGCCTGATGTGGCTGCAG	Yes	No	No	0	0	0	No	
NM_hsa_1679	chr3:50293915-50293974	+	CCACGCCACCTCTGCCAGCTGC	Yes	No	No	0	0	0	No	
NM_hsa_168	chr1:28202665-28202719	-	TTTGTAGCTGTACACTAAGGGA	Yes	No	Yes	0	0	0	No	
NM_hsa_168	chr1:28202668-28202722	+	TTTGTAGCTGTACACTAAGGGA	Yes	No	Yes	0	0	0	No	
NM_hsa_168	chr1:28202636-28202744	-	TTTGTAGCTGTACACTAAGGGA	Yes	No	Yes	0	0	0	No	
NM_hsa_1680	chr10:6018981-6019043	-	CGGCGTCCAGCAGTTTGG	Yes	No	No	0	0	0	No	
NM_hsa_1681	chr15:75644306-75644383	+	TTTCCTTCCCCTTGCACCCAGA	Yes	Yes	Yes	0	0	0	Yes	
NM_hsa_1681	chr15:75644318-75644374	+	TTTCCTTCCCCTTGCACCCAGA	Yes	Yes	Yes	0	0	0	Yes	
NM_hsa_1681	chr15:75644300-75644388	+	TTTCCTTCCCCTTGCACCCAGA	Yes	Yes	Yes	0	0	0	Yes	
NM_hsa_1682	chr15:75644318-75644374	+	CGGGGGCAGGGAGAAGAGGAACT	Yes	No	No	0	0	0.0732862	No	
NM_hsa_1685	chr11:61915507-61915569	+	TCATGAGCCAGTGTGTTGTGGA	Yes	Yes	No	0	0	0	Yes	
NM_hsa_1685	chr11:61915497-61915575	+	TCATGAGCCAGTGTGTTGTGGA	Yes	Yes	No	0	0	0	Yes	
NM_hsa_1686	chr11:61915507-61915569	+	CACAGCCTCTTGTTCATGCTT	Yes	No	No	0	0	0	No	
NM_hsa_1687	chr1:245449551-245449615	+	TTGGGAGGCCAGATCCACTTTT	Yes	Yes	No	0	0	0	No	
NM_hsa_1687	chr1:245449539-245449624	+	TTGGGAGGCCAGATCCACTTTT	Yes	Yes	No	0	0	0	No	
NM_hsa_1688	chr1:245449551-245449615	+	ACTCAGGTGGGTGTGGTGGCTCATGC	Yes	No	No	0	0	0	Yes	
NM_hsa_169	chr1:28202665-28202719	-	CCTTAGTGTACAGCTACAAAAT	Yes	No	No	0	0	0	No	
NM_hsa_169	chr1:28202668-28202722	+	CCTTAGTGTACAGCTACAAAAT	Yes	No	No	0	0	0	No	
NM_hsa_1691	chr22:31556050-31556106	+	CGGAACCTTAGAGCTTCAGC	Yes	Yes	No	0	0	0	No	
NM_hsa_1691	chr22:31556023-31556115	+	CGGAACCTTAGAGCTTCAGC	Yes	Yes	No	0	0	0	No	
NM_hsa_1692	chr22:31556050-31556106	+	CGAAGCTCCAAGGTTCCTCC	Yes	No	No	0	0	0	No	
NM_hsa_1693	chr12:58388745-58388825	-	GATGCCTGGGAGTTGCCATCTGCT	Yes	No	No	2.67833	2.09705	2.36959	Yes	
NM_hsa_1694	chr12:58388745-58388825	-	AAGGTGGCAGTCTTTTTTTTCT	Yes	No	No	0	0	0	No	
NM_hsa_1695	chr5:96271113-96271165	+	CTCTGCCGAAAACACCTA	Yes	No	No	0	0	0	No	
NM_hsa_1696	chr5:96271113-96271165	+	GGTCCTTCGAGCAGTGAT	Yes	No	No	0	0	0	No	
NM_hsa_1699	chr3:28282843-28282906	-	AAAGTCCGAGCCCCGTC	Yes	No	No	0	0	0	No	
NM_hsa_170	chr8:127876737-127876873	-	TTTCTGAGTTCAGATCCCAGG	Yes	Yes	Yes	0	0	0	No	
NM_hsa_170	chr8:127876762-127876844	+	TTTCTGAGTTCAGATCCCAGG	Yes	Yes	Yes	0	0	0	No	
NM_hsa_170	chr8:127876769-127876833	-	TTTCTGAGTTCAGATCCCAGG	Yes	Yes	Yes	0	0	0	No	
NM_hsa_170	chr8:127876732-127876868	+	TTTCTGAGTTCAGATCCCAGG	Yes	Yes	Yes	0	0	0	No	
NM_hsa_170	chr8:127876772-127876836	+	TTTCTGAGTTCAGATCCCAGG	Yes	Yes	Yes	0	0	0	No	
NM_hsa_1700	chr2:76414851-76414910	+	TGAATTCTGATTTTCCACTGTT	Yes	Yes	No	0	0	0	Yes	
NM_hsa_1700	chr2:76414838-76414919	+	TGAATTCTGATTTTCCACTGTT	Yes	Yes	No	0	0	0	Yes	
NM_hsa_1701	chr2:76414851-76414910	+	AGGATGGAAAAGATGAATTCATC	Yes	No	No	0	0	0	No	
NM_hsa_1702	chr1:91852978-91853032	+	GGCCCCGCTTTCACGGTCTGT	Yes	No	No	0	0	0	No	
NM_hsa_1703	chr1:91852978-91853032	+	AGATCAAGCGAGCTTT	Yes	No	No	0	0	0	No	
NM_hsa_1704	chr5:138614607-138614669	+	TTTGCACCTCTGAGAGTGGAATG	Yes	Yes	No	0.569857	0.26489	0.293145	Yes	
NM_hsa_1704	chr5:138614597-138614678	+	TTTGCACCTCTGAGAGTGGAATG	Yes	Yes	No	0.569857	0.26489	0.293145	Yes	
NM_hsa_1705	chr5:138614607-138614669	+	TCCTAGTCTGGGTGCAAACAATT	Yes	Yes	No	0	0	0	Yes	
NM_hsa_1705	chr5:138614597-138614678	+	TCCTAGTCTGGGTGCAAACAATT	Yes	Yes	No	0	0	0	Yes	
NM_hsa_1706	chr1:94703309-94703351	+	GGGCTGCGTCGGCTGCC	Yes	No	No	0	0	0	No	
NM_hsa_1707	chr22:30178248-30178315	+	CACGGCAGACCGGGACTGTA	Yes	Yes	No	0	0	0	No	
NM_hsa_1707	chr22:30178236-30178324	+	CACGGCAGACCGGGACTGTA	Yes	Yes	No	0	0	0	No	
NM_hsa_1708	chr22:30178248-30178315	+	CAGCCCGGTCCCTGTGGG	Yes	No	No	0	0	0	No	
NM_hsa_1709	chr6:27445250-27445302	-	AACTCAGATCCCAGGAGAGA	Yes	No	No	0	0	0	No	
NM_hsa_171	chr8:127876769-127876833	-	TGGGATCTGAACTCAGAAACT	Yes	No	No	0	0	0	No	
NM_hsa_171	chr8:127876772-127876836	+	TGGGATCTGAACTCAGAAACT	Yes	No	No	0	0	0	No	
NM_hsa_1710	chr6:27445250-27445302	-	TCTCCTGGAATCTGTATTAA	Yes	No	No	0	0	0	No	
NM_hsa_1711	chr4:8364901-8364964	-	AACTGGGCATGTTGGAACTAAGC	Yes	Yes	No	0.151962	0	0.097715	Yes	
NM_hsa_1711	chr4:8364894-8364974	-	AACTGGGCATGTTGGAACTAAGC	Yes	Yes	No	0.151962	0	0.097715	Yes	
NM_hsa_1711	chr4:7087114-7087177	+	AACTGGGCATGTTGGAACTAAGC	Yes	Yes	No	0.151962	0	0.097715	Yes	
NM_hsa_1712	chr4:8364901-8364964	-	TGATTCTAATGTGCCCAGAATT	Yes	No	No	0	0	0	No	
NM_hsa_1713	chr9:131662662-131662722	+	TGACTGCGCCCCACTCTGCACA	Yes	No	No	0.3989	0.0662225	0	No	KapB+cMyc>KapB>H
NM_hsa_1714	chr9:131662662-131662722	+	AGCATTTGGGGGCTGGACGCAGTGGC	Yes	No	No	0	0	0	No	
NM_hsa_1716	chr1:159895212-159895284	-	CAGGTCGGGGGTACGGGGGTCG	Yes	No	No	0	0	0	No	
NM_hsa_1717	chr17:42344324-42344398	+	TTTGGGGATTCTAAGAGGAAG	Yes	Yes	Yes	0	0	0	No	
NM_hsa_1717	chr17:42344333-42344389	+	TTTGGGGATTCTAAGAGGAAG	Yes	Yes	Yes	0	0	0	No	
NM_hsa_1717	chr17:42344317-42344403	+	TTTGGGGATTCTAAGAGGAAG	Yes	Yes	Yes	0	0	0	No	
NM_hsa_1718	chr17:42344333-42344389	+	AACTCTTAGAATCCCCAAAGC	Yes	No	No	0	0	0	No	
NM_hsa_172	chr9:132175588-132175666	-	CTCCTCGCCCCTTCCCCCGCCA	Yes	Yes	No	0	0.132445	0	Yes	
NM_hsa_172	chr9:132175579-132175672	-	CTCCTCGCCCCTTCCCCCGCCA	Yes	Yes	No	0	0.132445	0	Yes	
NM_hsa_1723	chr15:85590642-85590708	+	TCCGGCTGTTAGGAGACTCTG	Yes	Yes	No	0	0	0	No	
NM_hsa_1723	chr15:85590652-85590700	+	TCCGGCTGTTAGGAGACTCTG	Yes	Yes	No	0	0	0	No	
NM_hsa_1724	chr15:85590652-85590700	+	GAGGTCCTGAGAGCCGGATA	Yes	No	No	0	0	0	No	
NM_hsa_1725	chr16:89938437-89938496	-	AAAATACAGGCCAGAGAGTCC	Yes	No	No	0	0	0	No	
NM_hsa_1726	chr16:89938437-89938496	-	CCTCTCTGTGCCTGTTTTCTTA	Yes	No	No	0	0	0	No	
NM_hsa_1727	chr10:94624510-94624568	+	TCAGGTCTTCAGACTGTGGGG	Yes	Yes	No	0.3989	0.331113	0.146572	Yes	KapB+cMyc>KapB>H
NM_hsa_1727	chr10:94624500-94624576	+	TCAGGTCTTCAGACTGTGGGG	Yes	Yes	No	0.3989	0.331113	0.146572	Yes	KapB+cMyc>KapB>H
NM_hsa_1728	chr10:94624510-94624568	+	CCCCTGGTTTGCAGGCCTTTTG	Yes	No	No	0	0	0	No	
NM_hsa_1729	chr3:50223008-50223066	+	TCCCCCCAGTCCCAGCCTCTGA	Yes	Yes	No	0	0	0	Yes	
NM_hsa_1729	chr3:50222998-50223069	+	TCCCCCCAGTCCCAGCCTCTGA	Yes	Yes	No	0	0	0	Yes	
NM_hsa_173	chr9:132175588-132175666	-	GCGGGGGGCAGGGCGAGAGCA	Yes	No	No	0	0	0	No	
NM_hsa_1730	chr3:50223008-50223066	+	AGGGCAGGGAGGGGGTCCC	Yes	No	No	0	0	0	No	
NM_hsa_1731	chr10:114468976-114469036	+	AACACCTCAGAAAAGGGACAGA	Yes	Yes	Yes	0	0	0.146572	Yes	
NM_hsa_1731	chr10:114468952-114469060	+	AACACCTCAGAAAAGGGACAGA	Yes	Yes	Yes	0	0	0.146572	Yes	
NM_hsa_1731	chr10:114468962-114469045	+	AACACCTCAGAAAAGGGACAGA	Yes	Yes	Yes	0	0	0.146572	Yes	
NM_hsa_1732	chr10:114468976-114469036	+	TGTCCCTTCTTTAAGAGTGTCTG	Yes	No	No	0	0	0	No	
NM_hsa_1733	chr19:1422706-1422787	+	TCATCCAGTCCTCCCAGTGTGG	Yes	Yes	No	0	0	0	Yes	
NM_hsa_1733	chr19:1422716-1422780	+	TCATCCAGTCCTCCCAGTGTGG	Yes	Yes	No	0	0	0	Yes	
NM_hsa_1734	chr19:1422716-1422780	+	GTCAGCTGGGTCAGCTGGCTCGGT	Yes	No	No	0	0	0	No	
NM_hsa_1735	chr2:3583842-3583932	+	GAAGAACGGTCGGCCTAGAG	Yes	Yes	No	0	0	0	No	
NM_hsa_1735	chr2:3583852-3583905	+	GAAGAACGGTCGGCCTAGAG	Yes	Yes	No	0	0	0	No	
NM_hsa_1736	chr2:3583852-3583905	+	CGGCTGCAGTTTCCTCCC	Yes	No	No	0	0	0	No	
NM_hsa_1737	chr2:189355167-189355248	+	GGGGTTTGGCAGAGATGTG	Yes	No	No	0	0	0	No	
NM_hsa_1738	chr2:189355167-189355248	+	CATCTGTGGAACCCTGCCCCTG	Yes	No	No	0	0	0	No	
NM_hsa_1739	chr2:234197384-234197445	+	CCAGCTGCCTCTCCTCCATCG	Yes	Yes	No	0	0	0	Yes	
NM_hsa_1739	chr2:234197374-234197452	+	CCAGCTGCCTCTCCTCCATCG	Yes	Yes	No	0	0	0	Yes	
NM_hsa_174	chr1:183645825-183645891	+	GAGCCAGAGGCCCCTGGGCTGT	Yes	Yes	Yes	0.227943	0.198668	0.219859	No	
NM_hsa_174	chr1:183645795-183645913	+	GAGCCAGAGGCCCCTGGGCTGT	Yes	Yes	Yes	0.227943	0.198668	0.219859	No	
NM_hsa_174	chr1:183645815-183645899	+	GAGCCAGAGGCCCCTGGGCTGT	Yes	Yes	Yes	0.227943	0.198668	0.219859	No	
NM_hsa_1740	chr2:234197384-234197445	+	ATGGGCAGAAGGGCAGCTGACA	Yes	No	No	0.113971	0	0.439717	No	
NM_hsa_1743	chr2:158733341-158733428	+	TGCATGTCAGACTTGGGCTGGA	Yes	Yes	No	0	0	0	No	
NM_hsa_1743	chr2:158733351-158733425	+	TGCATGTCAGACTTGGGCTGGA	Yes	Yes	No	0	0	0	No	
NM_hsa_1744	chr2:158733351-158733425	+	CAGGCCATCTCTGACCTGCCTT	Yes	No	No	0	0	0	No	
NM_hsa_1745	chr17:7344638-7344714	+	GGGAGAGAGCGTGCTTGGA	Yes	No	No	0	0	0	Yes	
NM_hsa_1746	chr17:7344638-7344714	+	CTTTCATTCTCCTTTCTTCC	Yes	No	No	0	0	0	No	
NM_hsa_1747	chr19:45260213-45260275	+	CTCACAGTCACTGTTCCCCAGA	Yes	No	No	0.142464	0.463558	0	No	
NM_hsa_1748	chr19:45260213-45260275	+	GGGGGAGGCAGGACTAGAGAGCA	Yes	No	No	0	0	0	No	
NM_hsa_1749	chr16:56512503-56512555	+	TTTGAGACTCATGGCTGTAGG	Yes	Yes	No	0	0	0	No	
NM_hsa_1749	chr16:56512491-56512564	+	TTTGAGACTCATGGCTGTAGG	Yes	Yes	No	0	0	0	No	
NM_hsa_175	chr1:183645825-183645891	+	AGCCCAGGGGCCTCTGGCTTTG	Yes	No	No	0	0	0	No	
NM_hsa_1750	chr16:56512503-56512555	+	AACTGTCATGTGTGCCAAGCT	Yes	No	No	0	0	0	No	
NM_hsa_1751	chr1:150587911-150587998	-	AGCCCAGGACGAGACTGCAGTG	Yes	Yes	No	0	0	0	No	
NM_hsa_1753	chr3:63833515-63833568	-	TTCAATTGGCTGGAGGGCTTAGA	Yes	Yes	No	0	0	0	No	
NM_hsa_1753	chr3:63833506-63833580	-	TTCAATTGGCTGGAGGGCTTAGA	Yes	Yes	No	0	0	0	No	
NM_hsa_1754	chr3:63833515-63833568	-	AAAGCCCTTGGCCTATTGAATG	Yes	No	No	0	0	0	No	
NM_hsa_1756	chr12:7088434-7088516	+	AAGGTTGTTCAGTGCTGGCTGAT	Yes	Yes	No	0.113971	0	0	No	
NM_hsa_1756	chr12:7088444-7088504	+	AAGGTTGTTCAGTGCTGGCTGAT	Yes	Yes	No	0.113971	0	0	No	
NM_hsa_1757	chr12:7088444-7088504	+	CGGCTGGCATTGACAACTCACA	Yes	No	No	0	0	0	No	
NM_hsa_1758	chr19:35605931-35605997	+	CACATAGAGAAGGGGTGTAG	Yes	No	No	0	0	0	No	
NM_hsa_1759	chr19:35605931-35605997	+	CTTACCCTTGTCTAGGA	Yes	No	No	0	0	0	No	
NM_hsa_176	chr8:94908931-94909022	-	TTGGACTGTCTTGCAACTCTCT	Yes	No	No	0	0	0	No	
NM_hsa_176	chr8:94908934-94909025	+	TTGGACTGTCTTGCAACTCTCT	Yes	No	No	0	0	0	No	
NM_hsa_1760	chr4:123484757-123484819	+	TAAATGATCGTAGTGATTCAGA	Yes	Yes	Yes	0	0	0	Yes	
NM_hsa_1760	chr4:123484717-123484855	+	TAAATGATCGTAGTGATTCAGA	Yes	Yes	Yes	0	0	0	Yes	
NM_hsa_1760	chr4:123484747-123484826	+	TAAATGATCGTAGTGATTCAGA	Yes	Yes	Yes	0	0	0	Yes	
NM_hsa_1761	chr4:123484757-123484819	+	TGAATCATTGCGATCGTTTCTA	Yes	No	No	0	0	0	No	
NM_hsa_1766	chr22:42068981-42069027	+	TCCCCCAGGTGTGCTTCTT	Yes	No	No	0.0284929	0.0331113	0.0366431	No	H>KapB>KapB+cMyc

Additional file 5

Name	locus	strand	sequence	mirdeep2	mireap	miranalyzer	RPM_HU_KapB_cMyc	RPM_HU_KaposinB	RPM_HUVEC	IP_confidence	annotation
NM_hsa_1767	chr22:42068981-42069027	+	GGGTATATTGGCCCAAG	Yes	No	No	0	0	0	No	
NM_hsa_1768	chr16:11024667-11024712	+	CCTCAGGCTGTAAGTGCAGGCAGA	Yes	No	No	0	0	0	No	
NM_hsa_1769	chr16:11024667-11024712	+	TGCCTCCAGAGCCTGCTTGG	Yes	No	No	0	0	0	No	
NM_hsa_177	chr8:94908931-94909022	-	AGAGTTGCAAGACAGTCCAAGA	Yes	No	No	0	0	0	No	
NM_hsa_177	chr8:94908934-94909025	+	AGAGTTGCAAGACAGTCCAAGA	Yes	No	No	0	0	0	No	
NM_hsa_1770	chr19:35761295-35761351	+	TCTGCCCTCCTACTCCCCAGG	Yes	No	No	0	0	0	Yes	
NM_hsa_1771	chr19:35761295-35761351	+	ACGGGATGGAGGAACAGAGA	Yes	No	No	0	0	0	No	
NM_hsa_1772	chr5:56162429-56162489	+	AGTGCCCAGTTTGAGAAGCATT	Yes	Yes	Yes	0	0	0	No	
NM_hsa_1772	chr5:56162415-56162498	+	AGTGCCCAGTTTGAGAAGCATT	Yes	Yes	Yes	0	0	0	No	
NM_hsa_1772	chr5:56162405-56162513	+	AGTGCCCAGTTTGAGAAGCATT	Yes	Yes	Yes	0	0	0	No	
NM_hsa_1773	chr5:56162429-56162489	+	TGCTTCTCTTACCAGGGCACCTTC	Yes	No	No	0	0	0	Yes	
NM_hsa_1774	chr1:17294640-17294699	+	TGAGCCCCATGCCACCTGCAGG	Yes	No	No	0	0.0662225	0	Yes	
NM_hsa_1775	chr1:17294640-17294699	+	TGCGGGAGGGCTGCGGGGTGCACT	Yes	No	No	0	0	0	No	
NM_hsa_1776	chr7:74140308-74140373	+	CTTCCCTTCCCCTGGGCCTCAGC	Yes	No	No	0	0	0.293145	Yes	
NM_hsa_1777	chr7:74140308-74140373	+	TGAGATCCATGGGATGGAGCTC	Yes	No	No	0	0	0	Yes	
NM_hsa_1778	chr7:129414768-129414832	+	TTATGGCCCTTCGGTAATTCACT	Yes	Yes	No	0	0	0	Yes	
NM_hsa_1778	chr7:129414758-129414840	+	TTATGGCCCTTCGGTAATTCACT	Yes	Yes	No	0	0	0	Yes	
NM_hsa_1779	chr7:129414768-129414832	+	TGAATTCTACCAGTGCCATACAC	Yes	No	No	0	0	0	Yes	
NM_hsa_178	chr4:86594807-86594881	+	ATGCTTAGATCTCCCCACTGGT	Yes	Yes	No	0	0	0	No	
NM_hsa_178	chr4:86594817-86594873	+	ATGCTTAGATCTCCCCACTGGT	Yes	Yes	No	0	0	0	No	
NM_hsa_1784	chr5:77863355-77863419	+	TTAGAAACATTGGTCTGTTACT	Yes	Yes	No	0	0	0.219859	No	
NM_hsa_1784	chr5:77863345-77863426	+	TTAGAAACATTGGTCTGTTACT	Yes	Yes	No	0	0	0.219859	No	
NM_hsa_1785	chr5:77863355-77863419	+	TAAGAGACTAATGTTTCTGAAA	Yes	No	No	0	0	0	No	
NM_hsa_1786	chr6:28616037-28616087	-	GGCTCTGTGGCTTAGTTGGC	Yes	No	No	0.569857	1.12578	0.513004	Yes	
NM_hsa_1787	chr6:28616037-28616087	-	CATAAAAGAAGCCACAGAATCAG	Yes	No	No	0	0	0	No	
NM_hsa_1789	chr1:204791131-204791188	+	AGGCCCACTCTGAACAGTTGTGC	Yes	No	No	0	0	0	No	
NM_hsa_179	chr4:86594817-86594873	+	CAGTGGGGGGATCTAAGCATTG	Yes	No	No	0	0	0	No	
NM_hsa_1790	chr1:204791131-204791188	+	ATAACTTCAGCTGGGCCCAT	Yes	No	No	0	0	0	No	
NM_hsa_1792	chr12:6624066-6624136	+	TTTTTCTGGGGATTTTAAGTC	Yes	No	No	0	0	0	No	
NM_hsa_1795	chr3:160232773-160232838	-	TGCATCTGAGGCTGTTGTA	Yes	No	No	0	0	0	No	
NM_hsa_1796	chr3:160232773-160232838	-	CAACATTGCCAATGATTATAACC	Yes	No	No	0	0	0	No	
NM_hsa_1797	chr18:6094670-6094725	-	TCATGATAAAATTGTGGACCAG	Yes	No	No	0	0	0	No	
NM_hsa_1798	chr18:6094670-6094725	-	AGTCCACAATTTTATCATGACC	Yes	No	No	0	0	0	No	
NM_hsa_1799	chr3:126800546-126800644	+	TCAGGCCCTGTCCCTCTCCAGC	Yes	Yes	No	0	0	0	Yes	
NM_hsa_1799	chr3:126800613-126800691	+	TCAGGCCCTGTCCCTCTCCAGC	Yes	Yes	No	0	0	0	Yes	
NM_hsa_180	chr1:203144249-203144326	-	CACCCACCTGCCCTTTCTTGCT	Yes	Yes	Yes	0	0	0	Yes	
NM_hsa_180	chr1:203144228-203144346	-	CACCCACCTGCCCTTTCTTGCT	Yes	Yes	Yes	0	0	0	Yes	
NM_hsa_180	chr1:203144257-203144316	-	CACCCACCTGCCCTTTCTTGCT	Yes	Yes	Yes	0	0	0	Yes	
NM_hsa_1800	chr3:126800613-126800691	+	TGGTCTTGGGCAGGCTCTCTCTGTGG	Yes	No	No	0	0	0	No	
NM_hsa_1801	chr15:23916614-23916690	+	ACTGTGAACTGCAAATCGTGGT	Yes	Yes	No	0	0	0	No	
NM_hsa_1801	chr15:23916627-23916681	+	ACTGTGAACTGCAAATCGTGGT	Yes	Yes	No	0	0	0	No	
NM_hsa_1802	chr15:23916627-23916681	+	CACGATTTGCAGCTCACAGTAA	Yes	No	No	0	0	0	No	
NM_hsa_1803	chr7:141466671-141466741	+	TTCTTGGCTATGTTCTGTCTGG	Yes	No	No	0	0	0	No	
NM_hsa_1803	chr7:141466605-141466693	+	TTCTTGGCTATGTTCTGTCTGG	Yes	No	No	0	0	0	No	
NM_hsa_1804	chr7:141466671-141466741	+	AGACAGTGTATTCATAGCCAGAGAGGA	Yes	No	No	0	0	0	No	
NM_hsa_1805	chr2:3307804-3307866	+	ACTGTGTGATTTGCTGACTCTG	Yes	Yes	No	0	0	0	No	
NM_hsa_1805	chr2:3307794-3307873	+	ACTGTGTGATTTGCTGACTCTG	Yes	Yes	No	0	0	0	No	
NM_hsa_1806	chr2:3307804-3307866	+	CGGTCACCAAGTTCACACAGAAA	Yes	No	No	0	0	0	No	
NM_hsa_1807	chr11:76155617-76155658	-	AGGTTAGGGACGATGGGA	Yes	No	No	0	0	0	No	
NM_hsa_1808	chr11:76155617-76155658	-	CTTTGTTCCTGAAGTGC	Yes	No	No	0	0	0	No	
NM_hsa_181	chr1:203144257-203144316	-	CAAGAAAGGGTAGGTGGGTGGG	Yes	No	No	0	0	0	No	
NM_hsa_1818	chr18:20814547-20814597	+	TTTTTCCTCTGACTTGTC	Yes	No	No	0	0	0	No	
NM_hsa_1819	chr13:112276339-112276401	+	TCCACAGGGTCCAGATCCGAGT	Yes	Yes	No	0	0	0	No	
NM_hsa_1819	chr13:112276369-112276457	+	TCCACAGGGTCCAGATCCGAGT	Yes	Yes	No	0	0	0	No	
NM_hsa_182	chr21:9826449-9826499	+	GGGCCCCGGGCCCTCGACCGGG	Yes	No	No	0	0	0	No	
NM_hsa_1820	chr13:112276339-112276401	+	CGGGGTCCGGATCCAAGTGCACT	Yes	No	No	0	0	0	No	
NM_hsa_1825	chr12:110757493-110757547	+	TACTACCTGTTGGTCCTGTAGG	Yes	No	No	0	0	0	Yes	
NM_hsa_1826	chr12:110757493-110757547	+	TATAGGACCAATAGGTAAGACC	Yes	No	No	0	0	0	No	
NM_hsa_1829	chr19:6393256-6393333	+	ACCTTCTTAGCGACTGTGGC	Yes	No	No	0	0	0	No	
NM_hsa_183	chr21:9826449-9826499	+	CGGCGGGGCTCGGAGCCGGG	Yes	No	No	0	0.0662225	0	No	
NM_hsa_1830	chr19:6393256-6393333	+	TATGGTCACCTCAAAAGGTCC	Yes	No	No	0	0	0	No	
NM_hsa_1831	chrX:118921315-118921385	-	ATGACAGATTGACATGGACAATT	Yes	No	No	1.25369	1.72179	2.05201	Yes	H>KapB>KapB+cMyc
NM_hsa_1832	chrX:118921315-118921385	-	AAAGCCGTGTTAATCATTTTTGA	Yes	No	No	0	0	0	Yes	
NM_hsa_1834	chr2:234357988-234358053	+	TGTGATGAGGAGGGGCTTT	Yes	No	No	0	0	0	No	
NM_hsa_1835	chr19:4635773-4635849	+	CCTGGACTTTGAGAGGTGAGC	Yes	Yes	No	0.113971	0	0.0732862	No	
NM_hsa_1835	chr19:4635783-4635842	+	CCTGGACTTTGAGAGGTGAGC	Yes	Yes	No	0.113971	0	0.0732862	No	
NM_hsa_1836	chr19:4635783-4635842	+	TCCCCTCCTTCTGTCCCTGCT	Yes	No	No	0	0	0	No	
NM_hsa_1837	chr11:65192931-65193006	+	CTTGCATACGCAGCAGATCAG	Yes	No	No	0	0	0	No	
NM_hsa_1838	chr11:65192931-65193006	+	GATTTGAAACTGTGAAGGTG	Yes	No	No	0	0	0	No	
NM_hsa_184	chr20:23078420-23078489	-	TTTGCCAGATCCTTGCACGGGT	Yes	No	No	0	0	0	No	
NM_hsa_184	chr20:23078428-23078497	+	TTTGCCAGATCCTTGCACGGGT	Yes	No	No	0	0	0	No	
NM_hsa_1841	chr11:90010216-90010281	+	TGACCTGTAGGCTCTTGTTAGC	Yes	No	Yes	0.512871	0.331113	0.0732862	No	KapB+cMyc>KapB>H
NM_hsa_1841	chr11:90010196-90010294	+	TGACCTGTAGGCTCTTGTTAGC	Yes	No	Yes	0.512871	0.331113	0.0732862	No	KapB+cMyc>KapB>H
NM_hsa_1842	chr11:90010216-90010281	+	TAACAAGATCTTACCAAGTCAGA	Yes	No	No	0	0	0	No	
NM_hsa_1849	chr2:9146603-9146671	+	TTGCTTCTCTGGAGAACTCTAGA	Yes	Yes	No	0	0	0	No	
NM_hsa_1849	chr2:9146590-9146680	+	TTGCTTCTCTGGAGAACTCTAGA	Yes	Yes	No	0	0	0	No	
NM_hsa_185	chr20:23078420-23078489	-	CAAGGATCTGGCAAAGCTGAGG	Yes	No	No	0	0	0	No	
NM_hsa_185	chr20:23078428-23078497	+	CAAGGATCTGGCAAAGCTGAGG	Yes	No	No	0	0	0	No	
NM_hsa_1850	chr2:9146603-9146671	+	TAGAGTTCTCCAGAGAAACACAA	Yes	No	No	0	0	0	No	
NM_hsa_1851	chr1:45277680-45277777	+	CACATTCATTTCTGTTCCCAG	Yes	Yes	No	0.0569857	0	0	No	
NM_hsa_1853	chr22:30234395-30234469	+	GAGGGCGGGCTGTGTGAT	Yes	No	No	0	0	0	No	
NM_hsa_1854	chr22:30234395-30234469	+	CCACGCCCCCTTCCC	Yes	No	No	0	0	0	No	
NM_hsa_1857	chr16:2683183-2683246	+	AACATGGTCCGAGGCTAGA	Yes	No	No	0.0569857	0	0	No	
NM_hsa_1858	chr16:2683183-2683246	+	TGGCCCGAGTGGACTTTCAGCA	Yes	No	No	0	0	0	No	
NM_hsa_1859	chr19:51340286-51340341	+	CTTCTGGAACCAGACAAGCTGC	Yes	Yes	No	0	0	0	Yes	
NM_hsa_1859	chr19:51340276-51340349	+	CTTCTGGAACCAGACAAGCTGC	Yes	Yes	No	0	0	0	Yes	
NM_hsa_186	chr6:109638885-109638972	-	GTGCACACGCCTGACCTTGACC	Yes	No	No	0	0	0	No	
NM_hsa_1860	chr19:51340286-51340341	+	CGCTGGGTCTGGATCCAGACAGC	Yes	No	No	0	0	0	No	
NM_hsa_1861	chr3:112455952-112456008	+	ACAGGTCCTAAGAGACTGCAT	Yes	No	No	0	0	0	Yes	
NM_hsa_1862	chr3:112455952-112456008	+	GTGGCCTCTAAGAACCCGCTC	Yes	No	No	0	0	0	No	
NM_hsa_1864	chr5:149784624-149784697	-	TCAGCAGGTTTCCCTGCATGGG	Yes	No	No	0	0	0	No	
NM_hsa_1869	chr1:153750878-153750940	+	TACCCAGGTCTCCCTTTCCCCAGC	Yes	No	No	0	0	0	Yes	
NM_hsa_187	chr6:109638885-109638972	-	TCAAGGTCAGACGTGTGCACCC	Yes	No	No	0	0	0	No	
NM_hsa_1870	chr1:153750878-153750940	+	TGGCAGGAGAGGGGGCTCATGTGACT	Yes	No	No	0	0	0	No	
NM_hsa_1871	chr3:195459964-195460021	+	GCTGGCTCTTTTGCTCTCCAG	Yes	Yes	Yes	0	0	0	No	
NM_hsa_1871	chr3:195459954-195460030	+	GCTGGCTCTTTTGCTCTCCAG	Yes	Yes	Yes	0	0	0	No	
NM_hsa_1871	chr3:195459934-195460050	+	GCTGGCTCTTTTGCTCTCCAG	Yes	Yes	Yes	0	0	0	No	
NM_hsa_1872	chr3:195459964-195460021	+	CTGGCGAGCTCATGGGCCAGAT	Yes	Yes	No	0	0	0	No	
NM_hsa_1872	chr3:195459954-195460030	+	CTGGCGAGCTCATGGGCCAGAT	Yes	Yes	No	0	0	0	No	
NM_hsa_1873	chr2:231274386-231274432	-	AGGCTGAGCTGTGATGACAA	Yes	No	No	0	0	0	No	
NM_hsa_1874	chr2:231274386-231274432	-	GTCTTGAGCTGTGCTTGT	Yes	No	No	0	0	0	No	
NM_hsa_1877	chr20:62890459-62890506	+	TTGAGGAGGGGCTGTAGGAA	Yes	No	No	0	0	0	No	
NM_hsa_1878	chr20:62890459-62890506	+	CTGGTAGCAGCCCCTCCTACACTT	Yes	No	No	0	0	0	No	
NM_hsa_1879	chr1:159462100-159462163	+	GATCTGAGTCAGGAGCTCTCTGT	Yes	Yes	Yes	0	0	0	No	
NM_hsa_1879	chr1:159462072-159462192	+	GATCTGAGTCAGGAGCTCTCTGT	Yes	Yes	Yes	0	0	0	No	
NM_hsa_1879	chr1:159462088-159462172	+	GATCTGAGTCAGGAGCTCTCTGT	Yes	Yes	Yes	0	0	0	No	
NM_hsa_188	chr11:118501675-118501735	+	ACAACAGCCTCGTTCTTCCTGC	Yes	Yes	No	0.7978	0.728448	0.58629	Yes	KapB+cMyc>KapB>H
NM_hsa_188	chr11:118501662-118501744	+	ACAACAGCCTCGTTCTTCCTGC	Yes	Yes	No	0.7978	0.728448	0.58629	Yes	KapB+cMyc>KapB>H
NM_hsa_1880	chr1:159462100-159462163	+	TGCAGGCTCCTGCACATCAGATATCTG	Yes	No	No	0	0	0	No	
NM_hsa_1881	chr11:43497642-43497707	+	TCTAGATTGTGGACTTTAGC	Yes	Yes	No	0	0	0	No	
NM_hsa_1881	chr11:43497632-43497726	+	TCTAGATTGTGGACTTTAGC	Yes	Yes	No	0	0	0	No	
NM_hsa_1882	chr11:43497642-43497707	+	TAAAGGTCACAATCTAGGTG	Yes	No	No	0	0	0	No	
NM_hsa_1883	chr16:643570-643637	-	ATGCCAGACAAGACTTCCAG	Yes	No	No	0	0	0	No	
NM_hsa_1884	chr16:643570-643637	-	GTGAAGCTGGAGAGGGCAATGT	Yes	No	No	0	0	0	No	
NM_hsa_1887	chr12:111779521-111779586	+	CTGGCCAGGAACTTTTGGGATGG	Yes	No	No	0	0	0	No	
NM_hsa_1888	chr12:111779521-111779586	+	CTCCCTCTCTTCCTGGCCCCAGCC	Yes	No	No	0	0	0	No	
NM_hsa_189	chr11:118501675-118501735	+	AGGAAGAACCAGGCTGTTATGC	Yes	No	No	0	0	0	No	
NM_hsa_1891	chr6:31782486-31782549	-	ATTGATTGGTCGGAGCAG	Yes	No	No	0	0	0	No	
NM_hsa_1892	chr6:31782486-31782549	-	GATCCGGGTAGGAGGAGC	Yes	No	No	0	0	0	No	
NM_hsa_1893	chr7:156756579-156756645	+	TCCATTCATCGTGCTTCCAGG	Yes	No	No	0.227943	0	0	Yes	
NM_hsa_1895	chr6:36954328-36954390	+	GCGGCCTGCGTGGAACTCTGC	Yes	No	No	0	0	0	No	
NM_hsa_1896	chr6:36954328-36954390	+	AGTTCTCTGCCCGCCCCTTTGCGG	Yes	No	No	0	0	0.0732862	No	
NM_hsa_1897	chr1:11586622-11586669	-	GGGGGGCTGCCTCGGCCTGGGC	Yes	No	No	0	0	0	No	
NM_hsa_19	chr12:6619458-6619524	+	AAGGTCTGTAATCTTGGTGGGC	Yes	No	No	0.0569857	0	0.0732862	No	
NM_hsa_190	chr17:79919290-79919367	+	GCGCCGCCGCCCCCCCCCGCG	Yes	No	No	0.0284929	0.0331113	0	Yes	
NM_hsa_1902	chr9:36154925-36154989	+	ACGGCCTGTAGAAGTAGGGA	Yes	No	No	0	0	0	No	
NM_hsa_1903	chr9:36154925-36154989	+	CTGTGAGAGCAGGCTAGTGT	Yes	No	No	0	0	0	No	
NM_hsa_1906	chr4:7087114-7087177	+	TGATTGTAATGTGCCCAGAATT	Yes	No	No	0	0	0	No	
NM_hsa_1907	chr13:21186431-21186475	+	CTCCAGGAACTGTGCACCC	Yes	No	No	501.075	784.935	616.044	Yes	
NM_hsa_1908	chr13:21186431-21186475	+	ATGCATTGACCTGGTAGTG	Yes	No	No	0	0	0	No	
NM_hsa_1909	chr4:1362041-1362095	+	CTGTTATGATGGCTTCTGAAC	Yes	No	No	0	0	0	No	
NM_hsa_191	chr17:79919290-79919367	+	TGGGGGGGCAGCGCGGCCG	Yes	No	No	0	0	0	No	
NM_hsa_1910	chr4:1362041-1362095	+	GCAGAAGTTACACATCTTAGACGGGG	Yes	No	No	0	0	0	No	
NM_hsa_1911	chr11:65270755-65270793	+	AGGGGATTTCAGGATTGAGA	Yes	No	No	0.0569857	0	0	No	
NM_hsa_1912	chr11:65270755-65270793	+	TCTCCTGACCCCTTC	Yes	No	No	0	0	0	No	
NM_hsa_1913	chr16:71792308-71792384	-	ACTCAAACTGCTGACAGCA	Yes	No	No	0.170957	0	0	No	
NM_hsa_1914	chr16:71792308-71792384	-	TTGGAGGATGAAAGTACGGAGTGA	Yes	No	No	1.25369	0.463558	0.879435	No	
NM_hsa_1915	chr10:121484438-121484530	+	GAAGGCAAGGAGTTAGGAGTAG	Yes	Yes	No	0	0	0	No	
NM_hsa_1915	chr10:121484452-121484521	+	GAAGGCAAGGAGTTAGGAGTAG	Yes	Yes	No	0	0	0	No	
NM_hsa_1916	chr10:121484452-121484521	+	TCTCCTACTTCTTGTCTTTTT	Yes	No	No	0	0	0	No	
NM_hsa_1919	chr16:57466266-57466348	-	AAGCCCAGATTTGTGACTATG	Yes	Yes	No	0.113971	0	0	Yes	
NM_hsa_1919	chr16:57466275-57466334	-	AAGCCCAGATTTGTGACTATG	Yes	Yes	No	0.113971	0	0	Yes	
NM_hsa_192	chr4:86594806-86594880	-	ATGCTTAGATCCCCCCACTGGT	Yes	Yes	No	0	0	0	No	
NM_hsa_192	chr4:86594814-86594870	-	ATGCTTAGATCCCCCCACTGGT	Yes	Yes	No	0	0	0	No	
NM_hsa_1920	chr16:57466275-57466334	-	TAGTGACCTTTCTGGACTTGT	Yes	No	No	0	0	0	No	
NM_hsa_1921	chr17:37043135-37043194	+	AAAACAGGCTCTCGCTCTGTAG	Yes	Yes	No	0	0	0.146572	Yes	
NM_hsa_1921	chr17:37043122-37043203	+	AAAACAGGCTCTCGCTCTGTAG	Yes	Yes	No	0	0	0.146572	Yes	
NM_hsa_1922	chr17:37043135-37043194	+	ACTGGAGCTGGCCTCTGTTTTA	Yes	No	No	0	0	0	Yes	
NM_hsa_1923	chrX:51933793-51933845	+	CAGACAAATGCCCTACATGT	Yes	No	No	0	0	0	No	
NM_hsa_1923	chrX:51806442-51806494	-	CAGACAAATGCCCTACATGT	Yes	No	No	0	0	0	No	
NM_hsa_1924	chrX:51933793-51933845	+	GTGATGAGGGGCACAAACTTGTG	Yes	No	No	0	0	0	No	
NM_hsa_1924	chrX:51806442-51806494	-	GTGATGAGGGGCACAAACTTGTG	Yes	No	No	0	0	0	No	
NM_hsa_1925	chr17:37900260-37900324	+	CTCTGCTCTCCTCTGGCTCAGA	Yes	No	No	0.0569857	0	0	No	
NM_hsa_1926	chr17:37900260-37900324	+	TGGTGGTAGAGGGGAAGGGAGGA	Yes	No	No	0	0	0	No	
NM_hsa_1927	chr2:160986904-160986980	+	TTATGTCGGGATAAACTCATTG	Yes	Yes	No	0	0	0	Yes	
NM_hsa_1927	chr2:160986914-160986972	+	TTATGTCGGGATAAACTCATTG	Yes	Yes	No	0	0	0	Yes	
NM_hsa_1928	chr2:160986914-160986972	+	ATGGATTTATCCAGACATAACA	Yes	No	No	0	0	0	No	
NM_hsa_1929	chr19:11202090-11202150	+	TGAGGTCTCGCTTGGTTGCT	Yes	No	No	0	0	0	No	

Additional file 5

Name	locus	strand	sequence	mirdeep2	mireap	miranalyzer	RPM_HU_KapB_cMyc	RPM_HU_KaposinB	RPM_HUVEC	IP_confidence	annotation
NM_hsa_193	chr4:86594814-86594870	-	CAGTGGGGAGATCTAAGCATTG	Yes	No	No	0	0	0	No	
NM_hsa_1930	chr19:11202090-11202150	+	CCTCAAGCGATCCTCCTA	Yes	No	No	0.113971	0	0	Yes	
NM_hsa_1933	chr2:114035492-114035547	-	TGGACTGTGAGCTCCCCGC	Yes	No	No	0	0	0	No	
NM_hsa_1934	chr2:114035492-114035547	-	CGGGTGCGCGCAGTCCTTC	Yes	No	No	0	0	0	No	
NM_hsa_1935	chr13:42733748-42733817	+	ACCTCTGATGAACTCTGAGA	Yes	Yes	No	0.113971	0.0662225	0	No	KapB+cMyc>KapB>H
NM_hsa_1935	chr13:42733738-42733826	+	ACCTCTGATGAACTCTGAGA	Yes	Yes	No	0.113971	0.0662225	0	No	KapB+cMyc>KapB>H
NM_hsa_1936	chr13:42733748-42733817	+	TCAGACATTGGGGTTG	Yes	No	No	0	0	0	No	
NM_hsa_1937	chr20:45112781-45112832	-	CATCTGTGGGATTATGAC	Yes	No	No	1.88528	0.695336	0.54354	Yes	KapB+cMyc>KapB>H
NM_hsa_1938	chr20:45112781-45112832	-	CAGAATCCCACCCAAGCA	Yes	No	No	0	0	0	No	
NM_hsa_194	chr6:51425076-51425132	-	TGCCTAGATTTTGTCCTGTACT	Yes	Yes	No	0	0	0	No	
NM_hsa_194	chr6:51425066-51425144	+	TGCCTAGATTTTGTCCTGTACT	Yes	Yes	No	0	0	0	No	
NM_hsa_194	chr6:51425079-51425135	+	TGCCTAGATTTTGTCCTGTACT	Yes	Yes	No	0	0	0	No	
NM_hsa_1943	chr10:80831879-80831955	-	CTGTGAGAGGAACAAGAAC	Yes	No	No	0	0	0	No	
NM_hsa_1944	chr10:80831879-80831955	-	TTTTGTCCCCACAAAGAAGCC	Yes	No	No	0	0	0	No	
NM_hsa_1945	chr5:167718384-167718447	-	AGGCTCTCGGCACAGGTGG	Yes	No	No	0	0	0	No	
NM_hsa_1946	chr5:167718384-167718447	-	GCCTGTACTTAAAACGCTCCTGT	Yes	No	No	0	0	0	No	
NM_hsa_1949	chr1:28573685-28573735	-	CGAGAAGAGGCTGAACTG	Yes	No	No	0	0	0	No	
NM_hsa_195	chr6:51425076-51425132	-	TACAGGACAAAATCTAGGCATG	Yes	No	No	0	0	0	No	
NM_hsa_195	chr6:51425079-51425135	+	TACAGGACAAAATCTAGGCATG	Yes	No	No	0	0	0	No	
NM_hsa_1950	chr1:28573685-28573735	-	GTCCGCCCCTGACTCCGTC	Yes	No	No	0	0	0	No	
NM_hsa_1951	chr7:25989545-25989603	+	AAGTTCTGTAGTGCACTGACT	Yes	Yes	No	0	0	0	No	
NM_hsa_1951	chr7:25989535-25989610	+	AAGTTCTGTAGTGCACTGACT	Yes	Yes	No	0	0	0	No	
NM_hsa_1952	chr7:25989545-25989603	+	TCGGAGTGTCTCAGAACTTTG	Yes	No	No	0	0	0	No	
NM_hsa_1953	chrY:19692289-19692341	-	GTCTGAGGAGCCAATGGGGT	Yes	No	No	0.854786	0.79467	0	Yes	KapB+cMyc>KapB>H
NM_hsa_1954	chrY:19692289-19692341	-	ATCACAGGTTCAGACAT	Yes	No	No	0	0	0	No	
NM_hsa_1955	chr17:41368125-41368174	+	GTCAGAATCCGGGCCAGA	Yes	No	No	0	0	0	Yes	
NM_hsa_1956	chr17:41368125-41368174	+	TGGTAAGGATTGGGACTC	Yes	No	No	0	0	0	No	
NM_hsa_1957	chr2:205575435-205575485	+	TATCCTCTGTGTTGGGGTCCTGGA	Yes	No	No	0	0	0	No	
NM_hsa_1958	chr2:205575435-205575485	+	TAGGAAACTTACATAGATTCTTGT	Yes	No	No	0	0	0	No	
NM_hsa_196	chr4:94280882-94280942	-	CTACTGTTTCATTTGCTAAGC	Yes	Yes	Yes	0	0	0	No	
NM_hsa_196	chr4:94280873-94280955	-	CTACTGTTTCATTTGCTAAGC	Yes	Yes	Yes	0	0	0	No	
NM_hsa_196	chr4:94280848-94280984	+	CTACTGTTTCATTTGCTAAGC	Yes	Yes	Yes	0	0	0	No	
NM_hsa_196	chr4:94280885-94280945	+	CTACTGTTTCATTTGCTAAGC	Yes	Yes	Yes	0	0	0	No	
NM_hsa_196	chr4:94280843-94280979	-	CTACTGTTTCATTTGCTAAGC	Yes	Yes	Yes	0	0	0	No	
NM_hsa_1961	chr12:76049395-76049444	+	GATAAAAGACAAAAGGCTAGGG	Yes	No	No	0	0	0	No	
NM_hsa_1962	chr17:39739706-39739765	-	TTCCCTTTCCCTCCCTCACAGA	Yes	No	No	0	0	0	Yes	
NM_hsa_1962	chr17:16722475-16722534	-	TTCCCTTTCCCTCCCTCACAGA	Yes	No	No	0	0	0	Yes	
NM_hsa_1963	chr17:39739706-39739765	-	TGGCAGGGAGAGAACGGCCACA	Yes	No	No	0	0	0	No	
NM_hsa_1966	chr17:57233146-57233231	+	CTTGAGTAGCTGGGCCTGTTG	Yes	No	No	0	0	0	No	
NM_hsa_1968	chr4:129523249-129523330	+	TCCCCAGGAGCCTGACTGGAGC	Yes	Yes	No	0	0	0	No	
NM_hsa_1968	chr4:129523262-129523321	+	TCCCCAGGAGCCTGACTGGAGC	Yes	Yes	No	0	0	0	No	
NM_hsa_1969	chr4:129523262-129523321	+	TCCTGTCTTGCTCCATGGTAATGAGG	Yes	No	No	0	0	0	No	
NM_hsa_197	chr4:94280885-94280945	+	TTAGCAAATGAAACAGTAGCT	Yes	No	No	0	0	0	No	
NM_hsa_1972	chr19:35224647-35224731	-	GGGCAGACCTTGTACCAG	Yes	No	No	0	0	0	No	
NM_hsa_1973	chr19:35224647-35224731	-	GCTACTACGCTGTCCAC	Yes	No	No	0	0	0	No	
NM_hsa_1974	chr11:111412280-111412351	+	ACTGCAGCCTCGGATTTGGA	Yes	No	No	0	0	0	No	
NM_hsa_1975	chr11:111412280-111412351	+	TGGCTTCTTTCAGTTGTTCTGC	Yes	No	No	0	0	0	No	
NM_hsa_1977	chr2:85555026-85555068	-	GGCGGGTAAGACCCTCCA	Yes	No	No	0	0	0	No	
NM_hsa_1978	chr19:6729174-6729234	+	TGGGCTTGAGTGATACTCCTGC	Yes	No	No	0	0	0	No	
NM_hsa_1979	chr19:6729174-6729234	+	TGGGGTCTCGCTTGTTGCCCTGG	Yes	No	No	0	0	0	No	
NM_hsa_198	chr17:65822071-65822137	-	GGGGGGCCGGCGGCGGCGGCGG	Yes	No	No	0.168644	0.0927115	0.0355985	Yes	KapB+cMyc>KapB>H
NM_hsa_1980	chr22:35732413-35732471	+	TGTTTCTGCTCCAGCCTTGACC	Yes	Yes	No	0.113971	0	0	No	
NM_hsa_1980	chr22:35732401-35732480	+	TGTTTCTGCTCCAGCCTTGACC	Yes	Yes	No	0.113971	0	0	No	
NM_hsa_1981	chr22:35732413-35732471	+	CCTGGCTGGCAGGGAAACCAGC	Yes	No	No	0	0	0	No	
NM_hsa_1982	chr19:51894688-51894766	+	TAGACACAGAATCTGGACCAC	Yes	Yes	No	0	0	0	No	
NM_hsa_1982	chr19:51894702-51894757	+	TAGACACAGAATCTGGACCAC	Yes	Yes	No	0	0	0	No	
NM_hsa_1983	chr19:51894702-51894757	+	TGTCCAGGTTCTTGTGATATTACT	Yes	No	No	0	0	0	No	
NM_hsa_1984	chr3:9933550-9933626	+	CTCTGCCAATACCTTCTCCCGC	Yes	Yes	No	0	0	0	Yes	
NM_hsa_1984	chr3:9933562-9933617	+	CTCTGCCAATACCTTCTCCCGC	Yes	Yes	No	0	0	0	Yes	
NM_hsa_1985	chr3:9933562-9933617	+	TGGAGAAGTATGGGCGGCCTGAG	Yes	No	No	0	0	0	No	
NM_hsa_199	chr17:65822071-65822137	-	GCCCCCGCCTCCTCGCCCCCCCC	Yes	No	No	0	0	0	Yes	
NM_hsa_1996	chr17:46134631-46134716	+	AGCCTCTGTGTTTTGCCCTAGT	Yes	Yes	No	0	0	0	No	
NM_hsa_1996	chr17:46134646-46134707	+	AGCCTCTGTGTTTTGCCCTAGT	Yes	Yes	No	0	0	0	No	
NM_hsa_1997	chr17:46134646-46134707	+	TGGGAGGAAAGCACAAGGTTTT	Yes	No	No	0	0	0	Yes	
NM_hsa_2	chrM:15955-16024	-	CAGAGAATAGTTTAAATTAGA	Yes	No	No	0.512871	0.927115	1.46572	Yes	H>KapB>KapB+cMyc
NM_hsa_20	chr12:6619458-6619524	+	CACTGTAAGGGCAGACCAACAAGA	Yes	No	No	0	0	0	No	
NM_hsa_200	chr10:50035510-50035600	+	TCTCTCTCCGTGTCAGTGTGC	Yes	Yes	No	0	0	0	No	
NM_hsa_200	chr10:50035523-50035591	+	TCTCTCTCCGTGTCAGTGTGC	Yes	Yes	No	0	0	0	No	
NM_hsa_2000	chr3:9439030-9439105	-	AGGGGAGCGGAACTCTGG	Yes	No	No	0	0.0662225	0	No	
NM_hsa_2001	chr3:9439030-9439105	-	AGGGGGGCCGTCTGGTGG	Yes	No	No	0	0	0	No	
NM_hsa_2002	chr12:96903118-96903256	+	TACATTTGACCTTTTCAGTTAG	Yes	Yes	Yes	0	0	0	No	
NM_hsa_2002	chr12:96903156-96903217	+	TACATTTGACCTTTTCAGTTAG	Yes	Yes	Yes	0	0	0	No	
NM_hsa_2002	chr12:96903144-96903226	+	TACATTTGACCTTTTCAGTTAG	Yes	Yes	Yes	0	0	0	No	
NM_hsa_2003	chr12:96903156-96903217	+	GACTGAAAATGTCAAATGTGCC	Yes	No	No	0	0	0	No	
NM_hsa_2006	chr12:46590639-46590712	-	ATTCTTGTTTCCAGGTCTGG	Yes	Yes	No	0	0	0	No	
NM_hsa_2006	chr12:46590648-46590703	-	ATTCTTGTTTCCAGGTCTGG	Yes	Yes	No	0	0	0	No	
NM_hsa_2007	chr12:46590648-46590703	-	AGACCTGGATTCAAAAACTA	Yes	No	No	0	0	0	No	
NM_hsa_2008	chr9:75773366-75773447	+	AGCATCTAACTGTTTTCTTTCCAG	Yes	Yes	No	0.170957	0	0	No	
NM_hsa_2008	chr9:75773376-75773438	+	AGCATCTAACTGTTTTCTTTCCAG	Yes	Yes	No	0.170957	0	0	No	
NM_hsa_2009	chr9:75773376-75773438	+	GGAAGGAGAGGTTGTGTGTGGT	Yes	No	No	0	0	0	No	
NM_hsa_201	chr10:50035523-50035591	+	ACACTGACACAGAGAGAGA	Yes	No	No	0	0	0	No	
NM_hsa_2010	chr5:140700473-140700524	+	GACAGTGTTTCGGAAGAG	Yes	No	No	0	0	0	No	
NM_hsa_2011	chr5:140700473-140700524	+	TTTCGCGAGAACTTGTTTT	Yes	No	No	0	0	0	No	
NM_hsa_2012	chr3:8788889-8788950	+	TTGGGAACTAGGACGCAGGCAG	Yes	No	No	0	0	0	No	
NM_hsa_2013	chr3:8788889-8788950	+	GCTTCCGCTCCTAGTTGCCTTTT	Yes	No	No	0	0	0	No	
NM_hsa_2018	chr19:52185392-52185457	+	GCTTCAGAGTCAGTTTCCTGAAGAATC	Yes	No	No	0	0	0	No	
NM_hsa_2019	chr2:118588136-118588189	+	TTTCCTTAATGTTTGCCTCCAG	Yes	No	No	0	0	0	No	
NM_hsa_202	chr1:171217975-171218060	+	TAAGTATACTGTTGGGTAGATT	Yes	Yes	Yes	0	0	0	No	
NM_hsa_202	chr1:171217988-171218051	+	TAAGTATACTGTTGGGTAGATT	Yes	Yes	Yes	0	0	0	No	
NM_hsa_202	chr1:171217962-171218080	+	TAAGTATACTGTTGGGTAGATT	Yes	Yes	Yes	0	0	0	No	
NM_hsa_2021	chr14:102276058-102276095	-	GTTCTGTTTGGCTGTCAGTG	Yes	No	No	0	0	0	No	
NM_hsa_2022	chr14:102276058-102276095	-	CTGCAAACGGAAAGA	Yes	No	No	0	0	0	No	
NM_hsa_2023	chr1:156711688-156711759	+	TATTCTACGACTCTGCCCACA	Yes	Yes	No	0	0	0.0732862	No	
NM_hsa_2023	chr1:156711700-156711750	+	TATTCTACGACTCTGCCCACA	Yes	Yes	No	0	0	0.0732862	No	
NM_hsa_2024	chr1:156711700-156711750	+	TGGGCAGGAGTAGAATGAA	Yes	No	No	0	0	0	No	
NM_hsa_2025	chr7:141466605-141466693	+	AACGGTCTGAGCAAGATTT	Yes	No	No	0	0	0	No	
NM_hsa_2026	chr1:222139255-222139341	+	CTTGTGCCCAGATGGCCTGAAG	Yes	Yes	No	0	0	0	No	
NM_hsa_2028	chr1:236070011-236070151	+	TCTGAGACTAGAGCAAAGCCCTT	Yes	Yes	Yes	0.0569857	0	0	Yes	
NM_hsa_2028	chr1:236070051-236070112	+	TCTGAGACTAGAGCAAAGCCCTT	Yes	Yes	Yes	0.0569857	0	0	Yes	
NM_hsa_2028	chr1:236070035-236070121	+	TCTGAGACTAGAGCAAAGCCCTT	Yes	Yes	Yes	0.0569857	0	0	Yes	
NM_hsa_2029	chr1:236070051-236070112	+	GAGACTTAGTTCTAGTCCTAGTAC	Yes	No	No	0	0	0	No	
NM_hsa_203	chr1:171217988-171218051	+	TCTACCCAGCAGTATACTTACC	Yes	No	No	0	0	0	No	
NM_hsa_2030	chr16:50677251-50677315	+	CCTGCTGGCATTGATACAGGTTG	Yes	No	No	0	0	0	No	
NM_hsa_2031	chr16:50677251-50677315	+	GCCTGACATATGCTAGCCACAT	Yes	No	No	0	0	0	No	
NM_hsa_2034	chr11:118491772-118491846	+	ACAGGGGGCTGGGAATGAGAC	Yes	Yes	No	0	0	0	No	
NM_hsa_2034	chr11:118491782-118491839	+	ACAGGGGGCTGGGAATGAGAC	Yes	Yes	No	0	0	0	No	
NM_hsa_2035	chr11:118491782-118491839	+	CTCACTCCTGCCTCCTGGCT	Yes	No	No	0	0	0	No	
NM_hsa_2038	chr20:45984655-45984702	+	TGGCTGTAGGCTGGCTTGC	Yes	No	No	0	0	0	No	
NM_hsa_2039	chr20:45984655-45984702	+	TAGCAACCTAAGTCAGG	Yes	No	No	0	0	0	No	
NM_hsa_204	chr6:6826321-6826413	+	TGCCCCAGCTCTTAACAGTGGA	Yes	Yes	No	0	0	0	No	
NM_hsa_204	chr6:6826333-6826404	+	TGCCCCAGCTCTTAACAGTGGA	Yes	Yes	No	0	0	0	No	
NM_hsa_2040	chr14:76640459-76640531	+	CAGCAACCTACAAGGGCAAGCA	Yes	No	No	0	0	0	No	
NM_hsa_2047	chr3:148601332-148601409	+	CATCCACCTATGTCTTCTGCAG	Yes	Yes	No	0	0	0	No	
NM_hsa_2047	chr3:148601342-148601400	+	CATCCACCTATGTCTTCTGCAG	Yes	Yes	No	0	0	0	No	
NM_hsa_2048	chr3:148601342-148601400	+	GCAGGAGCTATATGGATGGG	Yes	No	No	0	0	0	No	
NM_hsa_2049	chr6:109025459-109025538	+	CTCTGAGGATTCAAGCACTGGC	Yes	No	No	0	0	0	No	
NM_hsa_205	chr6:6826333-6826404	+	TGCTGTTGAGAGCTGGGGCATA	Yes	No	No	0	0	0	Yes	
NM_hsa_2051	chr22:21300936-21301000	+	AAGTTTCTCTGAAGGTGTAGAGC	Yes	No	No	69.8835	54.7826	40.2403	Yes	KapB+cMyc>KapB>H
NM_hsa_2053	chrM:3442-3488	-	GTCAGCGAAGGGTTGTAG	Yes	No	No	0	0	0	No	
NM_hsa_2054	chrM:3442-3488	-	GGGGCTCTTTGGTGAAGA	Yes	No	No	0	0	0	No	
NM_hsa_2057	chr4:154561034-154561118	+	AGAATTGTGGCTGGACATGTT	Yes	No	No	0	0	0	No	
NM_hsa_2058	chr4:154561034-154561118	+	CAGTCCACCCACAAACCTGA	Yes	No	No	0	0	0	No	
NM_hsa_2059	chr9:124031492-124031541	+	TTCTGACCAGGGCACCAGGA	Yes	No	No	0	0	0	No	
NM_hsa_206	chr9:131102776-131102853	+	GCTGCGCCGCCGGCTCTGTGG	Yes	Yes	No	0	0	0	No	
NM_hsa_206	chr9:131102788-131102844	+	GCTGCGCCGCCGGCTCTGTGG	Yes	Yes	No	0	0	0	No	
NM_hsa_2060	chr9:124031492-124031541	+	CTGTGTGTCCTGTTCTCCCAGGACC	Yes	No	No	0	0	0	No	
NM_hsa_2063	chr20:11189241-11189314	-	TTCTCTGATGAAATCACTT	Yes	No	No	2.25094	2.25157	2.49173	Yes	H>KapB>KapB+cMyc
NM_hsa_2064	chr20:11189241-11189314	-	GTAGGTTCTGGAGAACT	Yes	No	No	0	0	0	No	
NM_hsa_2068	chr7:105753665-105753746	+	AAAAACCAGCTTCCAAGGACT	Yes	Yes	No	0	0	0	No	
NM_hsa_2068	chr7:105753677-105753737	+	AAAAACCAGCTTCCAAGGACT	Yes	Yes	No	0	0	0	No	
NM_hsa_2069	chr7:105753677-105753737	+	TTCCTGGACAGCTGGAATGTGGTCA	Yes	No	No	0	0	0	No	
NM_hsa_207	chr9:131102788-131102844	+	CTGGGGCCGAGCGGCGTCAGGCGC	Yes	No	No	0	0	0	No	
NM_hsa_2070	chr1:151485256-151485312	-	AGAGTCGGGCAAATCTCTGG	Yes	No	No	0	0	0	No	
NM_hsa_2071	chr1:151485256-151485312	-	AGAGCCAGCCCCCAGACTCCCA	Yes	No	No	0	0	0	No	
NM_hsa_2072	chr2:172850158-172850210	+	AACTGGTAGCCTCTGGACACA	Yes	Yes	No	0	0.0662225	0	No	
NM_hsa_2072	chr2:172850148-172850217	+	AACTGGTAGCCTCTGGACACA	Yes	Yes	No	0	0.0662225	0	No	
NM_hsa_2073	chr2:172850158-172850210	+	TGGCCAGCAGCATACCAACAGC	Yes	No	No	0	0	0	No	
NM_hsa_2074	chr10:43904832-43904896	+	CTCTGACTGCCCCTGAGGCC	Yes	No	No	0	0	0	No	
NM_hsa_2075	chr10:43904832-43904896	+	GGGAGGGGGAGGAGGAGGAGGG	Yes	No	No	0	0	0	Yes	
NM_hsa_2078	chr3:98312729-98312789	+	TAGGGCTGGGAGGCTGAGGGCG	Yes	No	No	0.113971	0.0662225	0	No	KapB+cMyc>KapB>H
NM_hsa_2078	chr3:98312697-98312751	+	TAGGGCTGGGAGGCTGAGGGCG	Yes	No	No	0.113971	0.0662225	0	No	KapB+cMyc>KapB>H
NM_hsa_2079	chr3:98312697-98312751	+	GCTGTTTACTCAGCCAAGGA	Yes	No	No	0	0	0	No	
NM_hsa_208	chr16:75110498-75110579	+	ATGCCATGTGCTACGGACTGA	Yes	No	No	0	0	0	No	
NM_hsa_2081	chr2:201507289-201507362	+	CAGGTTCAGAAGATCTTAATCA	Yes	No	No	0	0	0	No	
NM_hsa_2082	chr8:86304016-86304082	+	TATTTCTTCTGACCATCACT	Yes	No	No	10.172	9.17182	10.3334	No	
NM_hsa_2083	chr8:86304016-86304082	+	TGATGGGAGAACTCGAACCTAGG	Yes	No	No	0	0	0	No	
NM_hsa_2084	chr19:12433798-12433866	+	TTCTGTAGTAGGTTGTAG	Yes	No	No	0	0	0	No	
NM_hsa_2085	chr19:12433798-12433866	+	ACTACCTATTGTGCAGAAGA	Yes	No	No	0	0	0	No	
NM_hsa_209	chr16:75110498-75110579	+	AGTCCATAGCACATGGCATCC	Yes	No	No	0	0	0	No	
NM_hsa_2096	chr6:33792348-33792401	-	GCACGGAGAGCCGGAGAG	Yes	No	No	0	0	0	No	
NM_hsa_2097	chr6:33792348-33792401	-	CTCCAGCACTTCCGCAC	Yes	No	No	0	0	0	No	
NM_hsa_2098	chrX:47084310-47084385	-	TGGAATGTGAGGAAGTGTGA	Yes	No	No	0	0	0	No	
NM_hsa_2099	chrX:47084310-47084385	-	ACCTTCATCCCCACCATG	Yes	No	No	0	0	0	No	
NM_hsa_21	chr10:5003136-5003219	+	TACAGATCTGGGTCTCTTAGA	Yes	Yes	Yes	0	0	0	No	
NM_hsa_21	chr10:5003146-5003210	+	TACAGATCTGGGTCTCTTAGA	Yes	Yes	Yes	0	0	0	No	
NM_hsa_21	chr10:5003106-5003242	+	TACAGATCTGGGTCTCTTAGA	Yes	Yes	Yes	0	0	0	No	
NM_hsa_210	chr5:108949190-108949247	+	TGACCTTCCAGAGTCCTTGCCAG	Yes	Yes	Yes	0	0	0	No	
NM_hsa_210	chr5:108949177-108949256	+	TGACCTTCCAGAGTCCTTGCCAG	Yes	Yes	Yes	0	0	0	No	

Additional file 5

Name	locus	strand	sequence	mirdeep2	mireap	miranalyzer	RPM_HU_KapB_cMyc	RPM_HU_KaposinB	RPM_HUVEC	IP_confidence	annotation
NM_hsa_210	chr5:108949146-108949286	+	TGACCTTCCAGAGTCCTTGCCAG	Yes	Yes	Yes	0	0	0	No	
NM_hsa_2100	chr1:173834848-173834912	-	TTGGCTGATGAATACTGA	Yes	No	No	0	0	0	No	
NM_hsa_2101	chr1:173834848-173834912	-	AGTGTTTATGTGTTTTCC	Yes	No	No	0	0	0	No	
NM_hsa_2102	chr10:4989335-4989396	-	ATACAGGTGATTGACTAG	Yes	No	No	0	0	0	No	
NM_hsa_2103	chr10:4989335-4989396	-	ACAACTGCCTGCTTGTCA	Yes	No	No	0	0	0	No	
NM_hsa_2104	chr13:92002869-92002929	-	ACCTGCACTGTAAGCACTTTGAC	Yes	Yes	No	0	0	0	No	
NM_hsa_2104	chr13:92002860-92002942	-	ACCTGCACTGTAAGCACTTTGAC	Yes	Yes	No	0	0	0	No	
NM_hsa_2105	chr13:92002869-92002929	-	TACAAGTGCCTTCACTGCAGTAG	Yes	No	No	0	0	0	No	
NM_hsa_2106	chr3:98312729-98312789	+	TGGCAGAGATCCCAGTCCCGCT	Yes	No	No	0	0	0	No	
NM_hsa_211	chr5:108949190-108949247	+	AGCAAGGGCTCCGGAAGGTCATC	Yes	No	No	0	0	0	No	
NM_hsa_2110	chr2:201683073-201683143	+	GTAAGTTTTTGTTTAAACCGTCTTTT	Yes	No	No	0	0	0	No	
NM_hsa_2111	chr1:110601597-110601657	+	CCTCAGAGCCAGCGTCAAGA	Yes	No	No	0	0	0	No	
NM_hsa_2112	chr1:110601597-110601657	+	TGCTGGGTCCCCTCCCGGAT	Yes	No	No	0	0	0	No	
NM_hsa_2113	chr6:32986887-32986955	+	GAGGTTGCGTCGGTTCTG	Yes	No	No	0	0	0	No	
NM_hsa_2114	chr6:32986887-32986955	+	GAAAACAGCAGCCCCTC	Yes	No	No	0	0	0	No	
NM_hsa_212	chr2:103048755-103048817	-	TGCAATTTTGCCTGATCAGAGG	Yes	Yes	Yes	0	0	0	No	
NM_hsa_212	chr2:103048721-103048849	-	TGCAATTTTGCCTGATCAGAGG	Yes	Yes	Yes	0	0	0	No	
NM_hsa_212	chr2:103048746-103048828	-	TGCAATTTTGCCTGATCAGAGG	Yes	Yes	Yes	0	0	0	No	
NM_hsa_2122	chr18:56826040-56826086	+	TGGTTTTCTTTTGTGGACT	Yes	No	No	0	0	0	No	
NM_hsa_2123	chr12:71892246-71892292	+	GGTTCCATAGTGTAGTGGTT	Yes	No	No	0	0.132445	0	Yes	
NM_hsa_2124	chr12:71892246-71892292	+	CCAACTTCACATTGTATCCTT	Yes	No	No	0	0	0	No	
NM_hsa_2127	chr20:8769037-8769096	+	GCAAACTGACATTTTCTTTTTCCAG	Yes	No	No	0	0	0	No	
NM_hsa_2128	chr20:8769037-8769096	+	GGGAAGGAAGCTCTGTTTGCAA	Yes	No	No	0	0	0	No	
NM_hsa_2129	chr12:4947007-4947046	-	CTGTAGCCTCTTCTGCTTG	Yes	No	No	0	0	0	No	
NM_hsa_213	chr2:103048755-103048817	-	TCTGATCAGGCAAAGTTGCAGG	Yes	No	No	0	0	0	No	
NM_hsa_2130	chr12:4947007-4947046	-	AGCAGATGCTCAGTA	Yes	No	No	0	0	0	No	
NM_hsa_2131	chr3:172241341-172241397	+	AGTAGTCGTTGGAAAGGAG	Yes	No	No	0	0	0	No	
NM_hsa_2132	chr3:172241341-172241397	+	CCCCTTTCCTACTGCCCA	Yes	No	No	0	0	0	No	
NM_hsa_2135	chr16:50678624-50678679	+	CACCTGATTCTGTTTCCTCTGA	Yes	No	No	0	0	0	No	
NM_hsa_2136	chr16:50678624-50678679	+	TCAAAAGGCAGCATCTGATTGTGAT	Yes	No	No	0	0	0	No	
NM_hsa_2137	chr17:8125793-8125852	+	ACAGAGTGCCTTCAGGACTGCAG	Yes	No	No	0	0	0	No	
NM_hsa_2138	chr17:8125793-8125852	+	GACAGTTCCTGGGCTCTCCCGAA	Yes	No	No	0	0	0	No	
NM_hsa_214	chr2:144337500-144337628	+	TTTTATTAAGAAAGAACACTGA	Yes	Yes	Yes	0	0	0	No	
NM_hsa_214	chr2:144337517-144337604	-	TTTTATTAAGAAAGAACACTGA	Yes	Yes	Yes	0	0	0	No	
NM_hsa_214	chr2:144337529-144337594	+	TTTTATTAAGAAAGAACACTGA	Yes	Yes	Yes	0	0	0	No	
NM_hsa_214	chr2:144337497-144337615	-	TTTTATTAAGAAAGAACACTGA	Yes	Yes	Yes	0	0	0	No	
NM_hsa_214	chr2:144337526-144337591	-	TTTTATTAAGAAAGAACACTGA	Yes	Yes	Yes	0	0	0	No	
NM_hsa_2142	chr12:104336300-104336342	+	TTTAGGTGATTAGGAAGA	Yes	No	No	0	0	0	No	
NM_hsa_2143	chr1:170649590-170649650	+	TATGACATCACTGGAACCCCGT	Yes	Yes	No	0	0	0	No	
NM_hsa_2143	chr1:170649581-170649659	+	TATGACATCACTGGAACCCCGT	Yes	Yes	No	0	0	0	No	
NM_hsa_2144	chr1:170649590-170649650	+	TGGGATCCTTTGGTGACATGAA	Yes	No	No	0	0	0	No	
NM_hsa_2145	chr5:50698183-50698233	+	ATTGGACTAGATTCTCTAACATT	Yes	No	No	0	0	0	No	
NM_hsa_2146	chr5:50698183-50698233	+	GAACTGAGAGTGATAGTCCAAGAT	Yes	No	No	0	0	0	No	
NM_hsa_2147	chr5:168406096-168406140	-	GTCCAGTTTTCCCAGGATG	Yes	No	No	0	0	0	Yes	
NM_hsa_2148	chr5:168406096-168406140	-	GCCTGGAGTGAGGTTGAAGGATAG	Yes	No	No	0	0	0	No	
NM_hsa_2149	chr11:57532844-57532908	+	TTACTGTAGTCTGTAGTTTTA	Yes	No	No	0	0	0	No	
NM_hsa_215	chr2:144337529-144337594	+	AGTGTTCTTTCTTAATAAAACA	Yes	No	No	0	0	0	No	
NM_hsa_215	chr2:144337526-144337591	-	AGTGTTCTTTCTTAATAAAACA	Yes	No	No	0	0	0	No	
NM_hsa_2150	chr11:57532844-57532908	+	AAACAACATAGATTAATCAAA	Yes	No	No	0	0	0	No	
NM_hsa_2151	chr17:16722475-16722534	-	TGGCAGGGAGAGAATGGCCACA	Yes	No	No	0	0	0	No	
NM_hsa_2152	chr13:113006387-113006454	+	AAGCTGCCAGTTGAACCACTGT	Yes	No	No	2.70682	2.88068	1.42908	Yes	
NM_hsa_2153	chr13:113006387-113006454	+	AGTAAGCAGTCCGGCAGGAATTTG	Yes	No	No	0	0	0	No	
NM_hsa_2154	chr1:70859275-70859325	+	TGAGGGAGTAGTTTGTGCAA	Yes	No	No	313.671	284.254	145.109	Yes	KapB+cMyc>KapB>H
NM_hsa_2155	chr1:70859275-70859325	+	GGGGGAAAGGTATTCCCACAGT	Yes	No	No	0	0	0	No	
NM_hsa_2157	chr20:47614704-47614761	+	TATTTTGTCTGGAATTATCTTCCT	Yes	No	No	0	0	0	No	
NM_hsa_2158	chr12:25409790-25409843	-	AGATTGTAGAGGAACAAGAG	Yes	No	No	0	0	0	No	
NM_hsa_2159	chr12:25409790-25409843	-	CTGTTAGCTGCTATCTGC	Yes	No	No	0	0	0	No	
NM_hsa_216	chr3:138553852-138553972	+	TCTACCGTTGCCAGCCCGGCTGC	Yes	No	Yes	0.455886	0.52978	0.219859	Yes	
NM_hsa_216	chr3:138553876-138553943	+	TCTACCGTTGCCAGCCCGGCTGC	Yes	No	Yes	0.455886	0.52978	0.219859	Yes	
NM_hsa_2160	chr12:97856551-97856604	+	ATTCTGCACGGACTGCTGGG	Yes	No	No	0	0	0	No	
NM_hsa_2161	chr12:97856551-97856604	+	TACATATCTGTCTCTGGAACAA	Yes	No	No	0	0	0	No	
NM_hsa_2164	chr3:186505088-186505150	+	AAAGCAGGATTCAGACTACAAT	Yes	No	No	1.76656	1.25823	0.439717	Yes	KapB+cMyc>KapB>H
NM_hsa_2165	chr3:186505088-186505150	+	TGTCGTTCCCCCTGCTTAAA	Yes	No	No	0	0	0	No	
NM_hsa_2166	chr6:50790656-50790749	+	TTTGTTGTGATTTGTACCTCTGG	Yes	Yes	No	0	0	0	No	
NM_hsa_2166	chr6:50790631-50790689	+	TTTGTTGTGATTTGTACCTCTGG	Yes	Yes	No	0	0	0	No	
NM_hsa_2167	chr6:50790631-50790689	+	AGGGGGAGCAGCACAGCAAATA	Yes	No	No	0	0	0	No	
NM_hsa_2169	chr20:36065455-36065501	-	AGAGTTTGATCCTGGCTCAG	Yes	No	No	0.455886	0.463558	0.146572	Yes	
NM_hsa_217	chr3:138553876-138553943	+	AGTTGGGCTGGCAGGGTGTGGG	Yes	No	No	0	0	0	No	
NM_hsa_2170	chr20:36065455-36065501	-	GAGTGGGGGTGGGGGTAGGA	Yes	No	No	0	0	0	No	
NM_hsa_2171	chr1:12232652-12232721	-	ACAGGAACTCTGGGCAACTG	Yes	No	No	0	0	0	No	
NM_hsa_2172	chr1:12232652-12232721	-	GGGAGGAAAGAAGTTGCTTTCT	Yes	No	No	0	0	0	No	
NM_hsa_218	chr11:62609168-62609210	-	AATGGATTTTTGGAAATAGG	Yes	No	No	0.170957	0.52978	0.146572	Yes	
NM_hsa_22	chr10:5003136-5003219	+	TTAAGTGGCCTGAATTTGTAGA	Yes	Yes	No	0	0	0	No	
NM_hsa_22	chr10:5003146-5003210	+	TTAAGTGGCCTGAATTTGTAGA	Yes	Yes	No	0	0	0	No	
NM_hsa_220	chr14:81686528-81686614	+	CGGGGCTGCGCGCCCGACCGG	Yes	Yes	No	0	0	0	Yes	
NM_hsa_220	chr14:81686538-81686605	+	CGGGGCTGCGCGCCCGACCGG	Yes	Yes	No	0	0	0	Yes	
NM_hsa_221	chr14:81686538-81686605	+	GGCCCGCGGCGGCCCCAGGC	Yes	No	No	0	0	0	No	
NM_hsa_222	chr7:99647002-99647091	-	TGTCCATAAACCTGGATCGAGG	Yes	Yes	No	0	0	0	No	
NM_hsa_222	chr7:99647010-99647081	-	TGTCCATAAACCTGGATCGAGG	Yes	Yes	No	0	0	0	No	
NM_hsa_223	chr7:99647010-99647081	-	TCGGTCCAGGTTTATGGACATG	Yes	No	No	0	0	0	No	
NM_hsa_224	chr4:94280882-94280942	-	TTAGCAAATGAAACAGTAGCG	Yes	No	No	0	0	0	No	
NM_hsa_225	chr16:31226769-31226860	+	GCCGTTCGGGCTGGCTCCCTG	Yes	Yes	No	0	0	0	No	
NM_hsa_225	chr16:31226781-31226851	+	GCCGTTCGGGCTGGCTCCCTG	Yes	Yes	No	0	0	0	No	
NM_hsa_226	chr16:31226781-31226851	+	GGGACTGACCAGCCCCTGCGGCCC	Yes	No	No	0	0	0	No	
NM_hsa_227	chr11:112563017-112563105	+	GAAGCTGGGCTCAGACTGCTGA	Yes	Yes	Yes	0	0	0	No	
NM_hsa_227	chr11:112563027-112563097	+	GAAGCTGGGCTCAGACTGCTGA	Yes	Yes	Yes	0	0	0	No	
NM_hsa_227	chr11:112562987-112563125	+	GAAGCTGGGCTCAGACTGCTGA	Yes	Yes	Yes	0	0	0	No	
NM_hsa_228	chr11:112563027-112563097	+	AGCAGTCTCAGCCCAGCTTCCT	Yes	No	No	0	0	0	No	
NM_hsa_229	chr10:123790175-123790251	+	CTGGGTCATGTCTGGCTGAGGT	Yes	Yes	Yes	0	0	0	No	
NM_hsa_229	chr10:123790188-123790242	+	CTGGGTCATGTCTGGCTGAGGT	Yes	Yes	Yes	0	0	0	No	
NM_hsa_229	chr10:123790185-123790239	-	CTGGGTCATGTCTGGCTGAGGT	Yes	Yes	Yes	0	0	0	No	
NM_hsa_229	chr10:123790156-123790274	-	CTGGGTCATGTCTGGCTGAGGT	Yes	Yes	Yes	0	0	0	No	
NM_hsa_230	chr10:123790188-123790242	+	CTCAGCCAGACATGACCCAGTC	Yes	No	No	0	0	0	No	
NM_hsa_231	chr6:120144241-120144322	+	CCACATACTTCACTTGCTCAGT	Yes	Yes	Yes	0	0	0	No	
NM_hsa_231	chr6:120144214-120144352	+	CCACATACTTCACTTGCTCAGT	Yes	Yes	Yes	0	0	0	No	
NM_hsa_231	chr6:120144254-120144313	+	CCACATACTTCACTTGCTCAGT	Yes	Yes	Yes	0	0	0	No	
NM_hsa_232	chr6:120144254-120144313	+	TGAGCAAGTGAAGTATGTGGTA	Yes	No	No	0	0	0	No	
NM_hsa_235	chr17:59438884-59438972	+	TAATATATCTGTGGACACATCTG	Yes	Yes	No	0	0	0	No	
NM_hsa_235	chr17:59438897-59438963	+	TAATATATCTGTGGACACATCTG	Yes	Yes	No	0	0	0	No	
NM_hsa_236	chr17:59438897-59438963	+	GATGTGTCCACAGGTATAATATA	Yes	No	No	0	0	0	No	
NM_hsa_237	chr8:74668055-74668193	-	TGTAGTTTCTCTGTATCACTGC	Yes	Yes	Yes	0	0	0	No	
NM_hsa_237	chr8:74668085-74668171	-	TGTAGTTTCTCTGTATCACTGC	Yes	Yes	Yes	0	0	0	No	
NM_hsa_237	chr8:74668094-74668157	-	TGTAGTTTCTCTGTATCACTGC	Yes	Yes	Yes	0	0	0	No	
NM_hsa_238	chr8:74668094-74668157	-	AGTGATACAGAGAGACTACAGA	Yes	No	No	0	0	0	No	
NM_hsa_239	chr2:103141896-103141958	+	TATGGTGACCAGGTACTGACC	Yes	Yes	No	0	0	0	No	
NM_hsa_239	chr2:103141886-103141965	+	TATGGTGACCAGGTACTGACC	Yes	Yes	No	0	0	0	No	
NM_hsa_240	chr2:103141896-103141958	+	TCAGTACCTGGTCACCATGGG	Yes	No	No	0	0	0	No	
NM_hsa_241	chr10:95976706-95976842	-	TTACTGTTGAGTCTAATCTTC	Yes	Yes	Yes	0	0	0	No	
NM_hsa_241	chr10:95976745-95976803	-	TTACTGTTGAGTCTAATCTTC	Yes	Yes	Yes	0	0	0	No	
NM_hsa_241	chr10:95976736-95976816	-	TTACTGTTGAGTCTAATCTTC	Yes	Yes	Yes	0	0	0	No	
NM_hsa_241	chr10:95976709-95976845	+	TTACTGTTGAGTCTAATCTTC	Yes	Yes	Yes	0	0	0	No	
NM_hsa_241	chr10:95976748-95976806	+	TTACTGTTGAGTCTAATCTTC	Yes	Yes	Yes	0	0	0	No	
NM_hsa_242	chr10:95976745-95976803	-	AGATTAGACTCAACAGTAAAT	Yes	No	No	0	0	0	No	
NM_hsa_242	chr10:95976748-95976806	+	AGATTAGACTCAACAGTAAAT	Yes	No	No	0	0	0	No	
NM_hsa_243	chr14:104095215-104095288	-	GCAGCCGCGATGACCGAG	Yes	No	No	0	0	0	No	
NM_hsa_244	chr14:104095215-104095288	-	CGGGGCAGGGCGCGGCTCGCGG	Yes	No	No	0	0	0	No	
NM_hsa_245	chr11:100841605-100841665	+	TTCGCTGGGAATTCAGCCTCTGC	Yes	No	No	0.0569857	0.0662225	0.0732862	Yes	H>KapB>KapB+cMyc
NM_hsa_246	chr11:100841605-100841665	+	AGAGGCTGAATTCCCAGTGAGTG	Yes	No	No	0	0	0	Yes	
NM_hsa_247	chr7:41697430-41697484	+	TCAGCCGTGTCTGTTCTCTGGC	Yes	Yes	No	0.0569857	0	0	No	
NM_hsa_247	chr7:41697417-41697493	+	TCAGCCGTGTCTGTTCTCTGGC	Yes	Yes	No	0.0569857	0	0	No	
NM_hsa_248	chr7:41697430-41697484	+	CAGAGAACAGGCACATCTGTCC	Yes	No	No	0	0	0	No	
NM_hsa_249	chr3:156445518-156445602	-	ACTGACTGTCTCTGAAGAAATC	Yes	Yes	No	0	0	0	No	
NM_hsa_249	chr3:156445528-156445595	+	ACTGACTGTCTCTGAAGAAATC	Yes	Yes	No	0	0	0	No	
NM_hsa_249	chr3:156445525-156445592	-	ACTGACTGTCTCTGAAGAAATC	Yes	Yes	No	0	0	0	No	
NM_hsa_25	chrX:15510162-15510240	-	CTACCAGTGAGGCTGACAGATG	Yes	Yes	No	0.0854786	0.0331113	0.0732862	Yes	
NM_hsa_25	chrX:15510171-15510230	-	CTACCAGTGAGGCTGACAGATG	Yes	Yes	No	0.0854786	0.0331113	0.0732862	Yes	
NM_hsa_250	chr3:156445528-156445595	+	TTTCTTCAGAGACAGTCAGTCA	Yes	No	No	0	0	0	No	
NM_hsa_250	chr3:156445525-156445592	-	TTTCTTCAGAGACAGTCAGTCA	Yes	No	No	0	0	0	No	
NM_hsa_251	chr5:176812429-176812506	+	CATGTGAGTCTCGGAGGGCTGT	Yes	Yes	No	0	0	0	No	
NM_hsa_251	chr5:176812417-176812515	+	CATGTGAGTCTCGGAGGGCTGT	Yes	Yes	No	0	0	0	No	
NM_hsa_252	chr5:176812429-176812506	+	ACCCCTCCGAGACTCACATGCA	Yes	No	No	0	0	0	No	
NM_hsa_253	chr3:195373768-195373832	+	AACATCAAACTGGAACATCTG	Yes	Yes	Yes	0	0	0	No	
NM_hsa_253	chr3:195668521-195668585	-	AACATCAAACTGGAACATCTG	Yes	Yes	Yes	0	0	0	No	
NM_hsa_253	chr3:197365885-197366001	-	AACATCAAACTGGAACATCTG	Yes	Yes	Yes	0	0	0	No	
NM_hsa_253	chr3:197365914-197365978	-	AACATCAAACTGGAACATCTG	Yes	Yes	Yes	0	0	0	No	
NM_hsa_253	chr3:195373745-195373861	+	AACATCAAACTGGAACATCTG	Yes	Yes	Yes	0	0	0	No	
NM_hsa_253	chr3:195373811-195373862	+	AACATCAAACTGGAACATCTG	Yes	Yes	Yes	0	0	0	No	
NM_hsa_253	chr3:195668512-195668598	-	AACATCAAACTGGAACATCTG	Yes	Yes	Yes	0	0	0	No	
NM_hsa_253	chr3:195668491-195668542	-	AACATCAAACTGGAACATCTG	Yes	Yes	Yes	0	0	0	No	
NM_hsa_253	chr5:1620426-1620490	-	AACATCAAACTGGAACATCTG	Yes	Yes	Yes	0	0	0	No	
NM_hsa_253	chr3:197365884-197365935	-	AACATCAAACTGGAACATCTG	Yes	Yes	Yes	0	0	0	No	
NM_hsa_254	chr5:1620426-1620490	-	GATGTTCCAGTTTGAGGGCC	Yes	No	No	0	0	0	No	
NM_hsa_255	chr2:16383058-16383156	-	GCAGATGCCTTGCTTGGCCAAT	Yes	Yes	Yes	0	0	0	No	
NM_hsa_255	chr2:16383058-16383141	+	GCAGATGCCTTGCTTGGCCAAT	Yes	Yes	Yes	0	0	0	No	
NM_hsa_255	chr2:16383033-16383171	+	GCAGATGCCTTGCTTGGCCAAT	Yes	Yes	Yes	0	0	0	No	
NM_hsa_255	chr2:16383077-16383129	-	GCAGATGCCTTGCTTGGCCAAT	Yes	Yes	Yes	0	0	0	No	
NM_hsa_255	chr2:16383080-16383132	+	GCAGATGCCTTGCTTGGCCAAT	Yes	Yes	Yes	0	0	0	No	
NM_hsa_256	chr2:16383077-16383129	-	TGGCCAAGCAAGGCATCTGCCC	Yes	No	No	0	0	0	No	
NM_hsa_256	chr2:16383080-16383132	+	TGGCCAAGCAAGGCATCTGCCC	Yes	No	No	0	0	0	No	
NM_hsa_257	chr2:85060872-85060922	-	AAGTGCCCAGGGGCTGTGAGC	Yes	Yes	No	0	0	0	Yes	
NM_hsa_257	chr2:85060863-85060935	-	AAGTGCCCAGGGGCTGTGAGC	Yes	Yes	No	0	0	0	Yes	
NM_hsa_258	chr2:85060872-85060922	-	TGACAGCCCCTGGGCACTCCT	Yes	No	No	0	0	0	No	
NM_hsa_259	chr2:88932783-88932877	-	TCTGTTCCTGAACCTAGCATGA	Yes	Yes	No	0	0	0	No	
NM_hsa_259	chr2:88932791-88932867	-	TCTGTTCCTGAACCTAGCATGA	Yes	Yes	No	0	0	0	No	
NM_hsa_259	chr2:88932794-88932870	+	TCTGTTCCTGAACCTAGCATGA	Yes	Yes	No	0	0	0	No	
NM_hsa_26	chrX:15510162-15510240	-	TCTGTCAGCCTCATTGGTAG	Yes	Yes	No	0	0	0	Yes	
NM_hsa_26	chrX:15510171-15510230	-	TCTGTCAGCCTCATTGGTAG	Yes	Yes	No	0	0	0	Yes	
NM_hsa_260	chr2:88932791-88932867	-	ATGCTAGGTTCAGGAACAGATA	Yes	No	No	0	0	0	No	

Additional file 5

Name	locus	strand	sequence	mirdeep2	mireap	miranalyzer	RPM_HU_KapB_cMyc	RPM_HU_KaposinB	RPM_HUVEC	IP_confidence	annotation
NM_hsa_260	chr2:88932794-88932870	+	ATGCTAGGTTCAGGAACAGATA	Yes	No	No	0	0	0	No	
NM_hsa_261	chr6:3504754-3504833	-	TGTCATGTTTGGGCTGATGAGA	Yes	Yes	Yes	0	0	0	No	
NM_hsa_261	chr6:3504762-3504823	-	TGTCATGTTTGGGCTGATGAGA	Yes	Yes	Yes	0	0	0	No	
NM_hsa_261	chr6:3504725-3504863	-	TGTCATGTTTGGGCTGATGAGA	Yes	Yes	Yes	0	0	0	No	
NM_hsa_262	chr6:3504762-3504823	-	TCATCAGCCAAAACATGACATA	Yes	No	No	0	0	0	No	
NM_hsa_263	chrX:134459119-134459179	-	TGCATGCCAGCCACTGTACTGA	Yes	Yes	Yes	0	0	0.0732862	Yes	
NM_hsa_263	chrX:134459095-134459203	-	TGCATGCCAGCCACTGTACTGA	Yes	Yes	Yes	0	0	0.0732862	Yes	
NM_hsa_263	chrX:134459110-134459192	-	TGCATGCCAGCCACTGTACTGA	Yes	Yes	Yes	0	0	0.0732862	Yes	
NM_hsa_264	chrX:134459119-134459179	-	AGAACAGTGGCTGGCATGCACT	Yes	No	No	0	0	0	No	
NM_hsa_265	chr6:12011896-12011991	+	CGGGTTCGTGCCGGGCCGGG	Yes	Yes	No	0	0	0	No	
NM_hsa_265	chr6:12011908-12011982	+	CGGGTTCGTGCCGGGCCGGG	Yes	Yes	No	0	0	0	No	
NM_hsa_266	chr6:12011908-12011982	+	GGGGCCGGCGCGGTCTGGG	Yes	No	No	0	0	0	No	
NM_hsa_267	chr5:102123207-102123263	+	CAGATTTCCTGGGCTTAGTGA	Yes	Yes	No	0	0	0	No	
NM_hsa_267	chr5:102123204-102123260	-	CAGATTTCCTGGGCTTAGTGA	Yes	Yes	No	0	0	0	No	
NM_hsa_267	chr5:102123194-102123272	+	CAGATTTCCTGGGCTTAGTGA	Yes	Yes	No	0	0	0	No	
NM_hsa_268	chr5:102123207-102123263	+	ACTAAGCCCAGGAAATCTGTG	Yes	No	No	0	0	0	No	
NM_hsa_269	chr10:72082639-72082769	-	CCCATCTGATGGGGAATGGCCTG	Yes	Yes	Yes	0.0569857	0.198668	0	Yes	
NM_hsa_269	chr10:72082673-72082732	-	CCCATCTGATGGGGAATGGCCTG	Yes	Yes	Yes	0.0569857	0.198668	0	Yes	
NM_hsa_269	chr10:72082664-72082744	-	CCCATCTGATGGGGAATGGCCTG	Yes	Yes	Yes	0.0569857	0.198668	0	Yes	
NM_hsa_27	chr10:107803675-107803737	+	TAGAGAACCATGGTCATAGACT	Yes	Yes	No	0	0	0	No	
NM_hsa_27	chr10:107803665-107803746	+	TAGAGAACCATGGTCATAGACT	Yes	Yes	No	0	0	0	No	
NM_hsa_270	chr10:72082673-72082732	-	GGCCATTCCCCATCAGATGAGTC	Yes	No	No	0	0	0	No	
NM_hsa_273	chr18:15197815-15197866	+	GGACCAGGGGCCCTTGCCAGG	Yes	No	No	0	0	0	No	
NM_hsa_274	chr18:15197815-15197866	+	TAGGCAAGGAACCCCTGAGCCAG	Yes	No	No	0	0	0	No	
NM_hsa_275	chr7:6312182-6312253	-	GGGCCGCGCGCTGAGGAGC	Yes	No	No	0	0	0	No	
NM_hsa_277	chr14:91167034-91167114	-	CTTACAACTCTGGAAAAGCAGA	Yes	No	No	0	0	0	No	
NM_hsa_278	chr14:91167034-91167114	-	TGCTTTTCCGGAGTTGTAAGTG	Yes	No	No	0	0	0.146572	No	
NM_hsa_279	chr10:123790185-123790239	-	CTCAGCCAGACATGACCCAGAC	Yes	No	No	0	0	0	No	
NM_hsa_28	chr10:107803675-107803737	+	TCTATGACCATGGTTCTCTGTG	Yes	Yes	Yes	0	0	0	No	
NM_hsa_28	chr10:107803665-107803746	+	TCTATGACCATGGTTCTCTGTG	Yes	Yes	Yes	0	0	0	No	
NM_hsa_28	chr10:107803645-107803763	+	TCTATGACCATGGTTCTCTGTG	Yes	Yes	Yes	0	0	0	No	
NM_hsa_280	chr19:40931563-40931619	+	CCCTTCTCGGACTGCGGCGTGA	Yes	Yes	Yes	0	0	0	Yes	
NM_hsa_280	chr19:40931520-40931658	+	CCCTTCTCGGACTGCGGCGTGA	Yes	Yes	Yes	0	0	0	Yes	
NM_hsa_280	chr19:40931550-40931628	+	CCCTTCTCGGACTGCGGCGTGA	Yes	Yes	Yes	0	0	0	Yes	
NM_hsa_281	chr19:40931563-40931619	+	ACGCAGCCGGAGCCAAGAAGCCGG	Yes	No	No	0	0	0	No	
NM_hsa_282	chr15:71884923-71884987	+	ACTTTTATAGATAGGTCTGGGA	Yes	Yes	No	0	0	0	No	
NM_hsa_282	chr15:71884910-71884996	+	ACTTTTATAGATAGGTCTGGGA	Yes	Yes	No	0	0	0	No	
NM_hsa_283	chr15:71884923-71884987	+	CCAGACCTGTCTATAAAAGTGA	Yes	No	No	0	0	0	No	
NM_hsa_284	chr13:96705706-96705802	+	CGGACACTGGGCCACGTCGGA	Yes	Yes	No	0	0	0	No	
NM_hsa_284	chr13:96705730-96705793	+	CGGACACTGGGCCACGTCGGA	Yes	Yes	No	0	0	0	No	
NM_hsa_285	chr13:96705730-96705793	+	CGGCGCGGTTTAGTGGGT	Yes	No	No	0	0	0	No	
NM_hsa_286	chr5:118665445-118665583	+	CATGTCCAGACAAGGTTTGGGG	Yes	Yes	Yes	0	0	0	No	
NM_hsa_286	chr5:118665478-118665553	+	CATGTCCAGACAAGGTTTGGGG	Yes	Yes	Yes	0	0	0	No	
NM_hsa_286	chr5:118665490-118665544	+	CATGTCCAGACAAGGTTTGGGG	Yes	Yes	Yes	0	0	0	No	
NM_hsa_287	chr5:118665490-118665544	+	CCAGCCTTGTCTAGACATGGA	Yes	No	No	0	0	0	No	
NM_hsa_288	chr17:38278611-38278692	-	CGTGGAGGCCGCGGCTGAG	Yes	Yes	No	0	0	0	Yes	
NM_hsa_290	chr13:80583310-80583399	-	TGAGTCATTGTGGAAAGAGTAG	Yes	No	No	0	0	0	No	
NM_hsa_291	chr13:80583310-80583399	-	ACTCTTTCCACAGTGACTCAAA	Yes	No	No	0	0	0	No	
NM_hsa_292	chr3:189900523-189900590	+	GAATTATAACTCGGTGCTCTGT	Yes	Yes	Yes	0.0569857	0	0	Yes	
NM_hsa_292	chr3:189900491-189900629	+	GAATTATAACTCGGTGCTCTGT	Yes	Yes	Yes	0.0569857	0	0	Yes	
NM_hsa_292	chr3:189900510-189900599	+	GAATTATAACTCGGTGCTCTGT	Yes	Yes	Yes	0.0569857	0	0	Yes	
NM_hsa_293	chr3:189900523-189900590	+	AGAGCACTGAGTTATAATGCCA	Yes	No	No	0	0	0	Yes	
NM_hsa_294	chr16:29937602-29937643	+	CCCGTAGAGGTTTGGCGTG	Yes	No	No	0	0	0	No	
NM_hsa_295	chr16:29937602-29937643	+	CGCAGTGGCCTCGCTGGGGG	Yes	No	No	0	0	0	No	
NM_hsa_296	chr5:34538010-34538072	+	AGGGACCCCTGACTGACAACGC	Yes	No	No	0	0.0551854	0	No	
NM_hsa_297	chr5:34538010-34538072	+	ATTGCCAGCCAGGGGTCCCTGC	Yes	No	No	0	0	0	No	
NM_hsa_298	chr9:127964182-127964229	-	ATAGAAACGGGGCTGGGA	Yes	No	No	0	0	0	No	
NM_hsa_299	chr9:127964182-127964229	-	CCGGCCATTTATTTATTT	Yes	No	No	0	0	0	No	
NM_hsa_3	chr2:232321154-232321234	-	ATAGTCAAGTTCTGATCCAG	Yes	No	No	0.113971	0.0662225	0.0732862	Yes	
NM_hsa_300	chr5:102123204-102123260	-	ACTAAGCCCAGGAAATCTGTC	Yes	No	No	0	0	0	No	
NM_hsa_301	chr6:36020929-36021053	-	GAGAATATGGGAGTCTGTGG	Yes	Yes	Yes	0	0	0	No	
NM_hsa_301	chr6:36020960-36021021	+	GAGAATATGGGAGTCTGTGG	Yes	Yes	Yes	0	0	0	No	
NM_hsa_301	chr6:36020920-36021054	+	GAGAATATGGGAGTCTGTGG	Yes	Yes	Yes	0	0	0	No	
NM_hsa_301	chr6:36020957-36021018	-	GAGAATATGGGAGTCTGTGG	Yes	Yes	Yes	0	0	0	No	
NM_hsa_301	chr6:36020949-36021028	-	GAGAATATGGGAGTCTGTGG	Yes	Yes	Yes	0	0	0	No	
NM_hsa_302	chr6:36020960-36021021	+	ACAGACTCCCATATTCTCAT	Yes	No	No	0	0	0	No	
NM_hsa_302	chr6:36020957-36021018	-	ACAGACTCCCATATTCTCAT	Yes	No	No	0	0	0	No	
NM_hsa_303	chr21:41935303-41935365	+	CTCAGAGTTCCACCAGATCAAT	Yes	Yes	Yes	0	0	0	No	
NM_hsa_303	chr21:41935293-41935367	-	CTCAGAGTTCCACCAGATCAAT	Yes	Yes	Yes	0	0	0	No	
NM_hsa_303	chr21:41935263-41935401	-	CTCAGAGTTCCACCAGATCAAT	Yes	Yes	Yes	0	0	0	No	
NM_hsa_303	chr21:41935271-41935399	+	CTCAGAGTTCCACCAGATCAAT	Yes	Yes	Yes	0	0	0	No	
NM_hsa_304	chr21:41935293-41935373	+	ATTGATCTGGTGGAACTCTGAG	Yes	Yes	No	0	0	0	No	
NM_hsa_304	chr21:41935303-41935365	+	ATTGATCTGGTGGAACTCTGAG	Yes	Yes	No	0	0	0	No	
NM_hsa_305	chr9:29035564-29035622	+	TACCTTGGTTTTGACTTCAAGC	Yes	Yes	Yes	0	0	0	Yes	
NM_hsa_305	chr9:29035524-29035662	+	TACCTTGGTTTTGACTTCAAGC	Yes	Yes	Yes	0	0	0	Yes	
NM_hsa_305	chr9:29035561-29035619	-	TACCTTGGTTTTGACTTCAAGC	Yes	Yes	Yes	0	0	0	Yes	
NM_hsa_305	chr9:29035554-29035630	+	TACCTTGGTTTTGACTTCAAGC	Yes	Yes	Yes	0	0	0	Yes	
NM_hsa_305	chr9:29035531-29035649	-	TACCTTGGTTTTGACTTCAAGC	Yes	Yes	Yes	0	0	0	Yes	
NM_hsa_306	chr9:29035564-29035622	+	TTGAAGTCAAAACCAAGGTACA	Yes	No	No	0	0	0	Yes	
NM_hsa_306	chr9:29035561-29035619	-	TTGAAGTCAAAACCAAGGTACA	Yes	No	No	0	0	0	Yes	
NM_hsa_307	chr4:185423377-185423433	+	TGGTGTAGACAATGTGCACGGA	Yes	Yes	Yes	0	0	0	No	
NM_hsa_307	chr4:185423365-185423442	+	TGGTGTAGACAATGTGCACGGA	Yes	Yes	Yes	0	0	0	No	
NM_hsa_307	chr4:185423334-185423472	+	TGGTGTAGACAATGTGCACGGA	Yes	Yes	Yes	0	0	0	No	
NM_hsa_308	chr4:185423377-185423433	+	AGTGCACATGGTCTACACCACG	Yes	No	No	0	0	0	No	
NM_hsa_312	chr13:51614829-51614915	+	TATTTGATTTCTTCTGTAGGAC	Yes	Yes	Yes	0	0	0	No	
NM_hsa_312	chr13:51614816-51614934	-	TATTTGATTTCTTCTGTAGGAC	Yes	Yes	Yes	0	0	0	No	
NM_hsa_312	chr13:51614839-51614907	+	TATTTGATTTCTTCTGTAGGAC	Yes	Yes	Yes	0	0	0	No	
NM_hsa_312	chr13:51614836-51614904	-	TATTTGATTTCTTCTGTAGGAC	Yes	Yes	Yes	0	0	0	No	
NM_hsa_313	chr13:51614839-51614907	+	CCTACAGAAGAAATCAAATAAG	Yes	No	No	0	0	0	No	
NM_hsa_313	chr13:51614836-51614904	-	CCTACAGAAGAAATCAAATAAG	Yes	No	No	0	0	0	No	
NM_hsa_314	chr18:8891641-8891690	+	TTTCAGACATCAGCTGCAAGC	Yes	No	No	0	0	0	No	
NM_hsa_315	chr18:8891641-8891690	+	TTGCAGCTGATGCCTGAAGTG	Yes	No	No	0	0	0	No	
NM_hsa_316	chr3:193631983-193632050	+	ATGCTTTAGTTTGGACTTATGA	Yes	Yes	Yes	0	0	0	No	
NM_hsa_316	chr3:193631943-193632081	+	ATGCTTTAGTTTGGACTTATGA	Yes	Yes	Yes	0	0	0	No	
NM_hsa_316	chr3:193631973-193632058	+	ATGCTTTAGTTTGGACTTATGA	Yes	Yes	Yes	0	0	0	No	
NM_hsa_317	chr3:193631983-193632050	+	ATGAAGTCCAAACTAAAGCACCC	Yes	No	No	0	0	0	No	
NM_hsa_318	chr18:61089817-61089889	+	AGGCGCACTGAGACTCTGG	Yes	No	No	0	0	0	No	
NM_hsa_319	chr18:61089817-61089889	+	GGGAGAAGTAGCCTGG	Yes	No	No	0	0	0	No	
NM_hsa_320	chr15:80987263-80987301	-	GAAGCACCTTCTCGACCTG	Yes	No	No	0	0	0	No	
NM_hsa_321	chr15:80987263-80987301	-	GGCCGACGGGGGCTTCCA	Yes	No	No	0	0	0	No	
NM_hsa_322	chr10:77163631-77163700	+	GGGTCGGGTCTGGAACTGC	Yes	No	No	0	0	0	No	
NM_hsa_323	chr10:77163631-77163700	+	AGACCCGGTTTCGACCGGC	Yes	No	No	0	0	0	No	
NM_hsa_324	chr22:50757156-50757234	+	CTCACCCCTGTGGCACTCTGGGC	Yes	No	No	0	0	0	No	
NM_hsa_325	chr22:50757156-50757234	+	CCAGGCTACAGGGATGCCCA	Yes	No	No	0	0	0	No	
NM_hsa_328	chr14:72172376-72172449	+	CTCCAGCAGGACTGATGCTCC	Yes	Yes	Yes	0	0	0	No	
NM_hsa_328	chr14:72172343-72172479	+	CTCCAGCAGGACTGATGCTCC	Yes	Yes	Yes	0	0	0	No	
NM_hsa_328	chr14:72172389-72172440	+	CTCCAGCAGGACTGATGCTCC	Yes	Yes	Yes	0	0	0	No	
NM_hsa_329	chr14:72172389-72172440	+	AGCGTCAGTCCTGCTCCAGCC	Yes	No	No	0	0	0	No	
NM_hsa_33	chr8:99405896-99405952	-	CCAGCCTACTGGAGGATAAGAG	Yes	Yes	Yes	0	0	0	Yes	
NM_hsa_33	chr8:99405887-99405962	-	CCAGCCTACTGGAGGATAAGAG	Yes	Yes	Yes	0	0	0	Yes	
NM_hsa_33	chr8:99405854-99405992	-	CCAGCCTACTGGAGGATAAGAG	Yes	Yes	Yes	0	0	0	Yes	
NM_hsa_330	chr14:94802187-94802260	-	ATGTGTAGAGCCAGAAAGTGG	Yes	Yes	No	0	0	0	No	
NM_hsa_330	chr14:94802178-94802270	-	ATGTGTAGAGCCAGAAAGTGG	Yes	Yes	No	0	0	0	No	
NM_hsa_330	chr14:94802190-94802263	+	ATGTGTAGAGCCAGAAAGTGG	Yes	Yes	No	0	0	0	No	
NM_hsa_331	chr14:94802187-94802260	-	ACTTTCTGGCTCTACACATTT	Yes	No	No	0	0	0	No	
NM_hsa_331	chr14:94802190-94802263	+	ACTTTCTGGCTCTACACATTT	Yes	No	No	0	0	0	No	
NM_hsa_332	chr12:90300287-90300368	+	TTAACCTACTAGAGTCACTGTG	Yes	Yes	Yes	0	0	0	Yes	
NM_hsa_332	chr12:90300265-90300393	+	TTAACCTACTAGAGTCACTGTG	Yes	Yes	Yes	0	0	0	Yes	
NM_hsa_332	chr12:90300300-90300359	+	TTAACCTACTAGAGTCACTGTG	Yes	Yes	Yes	0	0	0	Yes	
NM_hsa_333	chr12:90300300-90300359	+	CATGACTCTAGTAGGTTGAGAAATA	Yes	No	No	0	0	0	No	
NM_hsa_334	chr9:37804224-37804280	-	TCTTTAGGAACTCTGGCTAACT	Yes	Yes	No	0.0427393	0.0331113	0.109929	Yes	
NM_hsa_334	chr9:37804215-37804293	-	TCTTTAGGAACTCTGGCTAACT	Yes	Yes	No	0.0427393	0.0331113	0.109929	Yes	
NM_hsa_335	chr9:37804224-37804280	-	TTAGCCAGAGTTCCTGAAGAAA	Yes	No	No	0	0	0	Yes	
NM_hsa_336	chr8:79637607-79637686	+	TTCAGCCAGAAAGAGTGCCAAG	Yes	Yes	Yes	0	0	0	No	
NM_hsa_336	chr8:79637587-79637705	+	TTCAGCCAGAAAGAGTGCCAAG	Yes	Yes	Yes	0	0	0	No	
NM_hsa_336	chr8:79637617-79637677	+	TTCAGCCAGAAAGAGTGCCAAG	Yes	Yes	Yes	0	0	0	No	
NM_hsa_337	chr8:79637617-79637677	+	TGGGCATTGTTTCTGGCTGAACA	Yes	No	No	0	0	0	No	
NM_hsa_34	chr8:99405896-99405952	-	TTATCCTCCAGTAGACTAGG	Yes	No	No	0	0	0	Yes	
NM_hsa_340	chr16:22335763-22335834	+	CTGGAGGGGAGGGGGCTGGCT	Yes	Yes	Yes	0	0	0.146572	No	
NM_hsa_340	chr16:22335773-22335826	+	CTGGAGGGGAGGGGGCTGGCT	Yes	Yes	Yes	0	0	0.146572	No	
NM_hsa_340	chr16:22335753-22335849	+	CTGGAGGGGAGGGGGCTGGCT	Yes	Yes	Yes	0	0	0.146572	No	
NM_hsa_341	chr16:22335773-22335826	+	CTGGGCCCCCTCCCCTCCAGCT	Yes	No	No	0	0	0	No	
NM_hsa_346	chr3:195373768-195373832	+	GATGTTCCAGTTTGATGGCCG	Yes	No	No	0	0	0	No	
NM_hsa_346	chr3:195668521-195668585	-	GATGTTCCAGTTTGATGGCCG	Yes	No	No	0	0	0	No	
NM_hsa_346	chr3:197365914-197365978	-	GATGTTCCAGTTTGATGGCCG	Yes	No	No	0	0	0	No	
NM_hsa_347	chr1:1140863-1140957	-	CTGGCCTGTCTCCTGCCCAG	Yes	Yes	No	0	0	0	No	
NM_hsa_347	chr1:1140872-1140927	-	CTGGCCTGTCTCCTGCCCAG	Yes	Yes	No	0	0	0	No	
NM_hsa_348	chr1:1140872-1140927	-	GGGGAGGAGGGGGCCTCAGCC	Yes	No	No	0	0	0	No	
NM_hsa_349	chr17:79485973-79486063	+	CAGGGCGGGCGTGGCTGGGA	Yes	Yes	No	0	0	0	No	
NM_hsa_349	chr17:79486034-79486075	+	CAGGGCGGGCGTGGCTGGGA	Yes	Yes	No	0	0	0	No	
NM_hsa_35	chr19:7533656-7533712	+	TATCTGCTGTTGTCCCCTCAGG	Yes	No	No	0.0284929	0.0662225	0	Yes	
NM_hsa_350	chr17:79486034-79486075	+	CCGGCTGTGCCTCGCCGCC	Yes	No	No	0	0	0	No	
NM_hsa_351	chr16:30406599-30406660	-	CGGCGGTAGCTCTGTGGC	Yes	No	No	0	0	0	No	
NM_hsa_352	chr16:30406599-30406660	-	CCAGGGGCTACTCTAAGCCGAG	Yes	No	No	0	0	0	No	
NM_hsa_353	chr16:3930876-3930952	+	CTCGGGCCGAAGGGCTGAAGG	Yes	No	No	0	0	0	No	
NM_hsa_354	chr16:3930876-3930952	+	GCGGGCCAGGCCTGCGGGG	Yes	No	No	0	0	0	No	
NM_hsa_355	chr1:102074799-102074878	+	TCTTCCAGATGTTACTGACTGC	Yes	Yes	Yes	0	0	0	No	
NM_hsa_355	chr1:102074811-102074869	+	TCTTCCAGATGTTACTGACTGC	Yes	Yes	Yes	0	0	0	No	
NM_hsa_355	chr1:102074795-102074883	+	TCTTCCAGATGTTACTGACTGC	Yes	Yes	Yes	0	0	0	No	
NM_hsa_356	chr1:102074811-102074869	+	AGTTAGTAACATCTGGAAGAGT	Yes	No	No	0	0	0	No	
NM_hsa_357	chrX:21520311-21520372	+	TGGGATTCTGTGCCTATTGTAG	Yes	Yes	Yes	0	0	0	No	
NM_hsa_357	chrX:21520278-21520406	+	TGGGATTCTGTGCCTATTGTAG	Yes	Yes	Yes	0	0	0	No	
NM_hsa_357	chrX:21520298-21520381	+	TGGGATTCTGTGCCTATTGTAG	Yes	Yes	Yes	0	0	0	No	
NM_hsa_358	chrX:21520311-21520372	+	ACAGTAGGCACAGAATCCCACT	Yes	No	No	0	0	0	No	
NM_hsa_36	chr19:7533656-7533712	+	CAGAGGCCGCACAGCAGGAAC	Yes	Yes	Yes	0	0	0	No	
NM_hsa_36	chr19:7533626-7533742	+	CAGAGGCCGCACAGCAGGAAC	Yes	Yes	Yes	0	0	0	No	
NM_hsa_36	chr19:7533646-7533720	+	CAGAGGCCGCACAGCAGGAAC	Yes	Yes	Yes	0	0	0	No	
NM_hsa_361	chr3:186545427-186545482	+	CCGCCAGCGCCGGCTGCCCAGC	Yes	No	No	0	0	0	No	

Additional file 5

Name	locus	strand	sequence	mirdeep2	mireap	miranalyzer	RPM_HU_KapB_cMyc	RPM_HU_KaposinB	RPM_HUVEC	IP_confidence	annotation
NM_hsa_362	chr3:186545427-186545482	+	GAGGAGCCGGAGCTGGCTCGG	Yes	No	No	0	0	0	No	
NM_hsa_363	chr2:174887562-174887628	-	CTTTAGGTAGGAGGCTGTTGA	Yes	Yes	No	0	0	0	No	
NM_hsa_363	chr2:174887553-174887640	-	CTTTAGGTAGGAGGCTGTTGA	Yes	Yes	No	0	0	0	No	
NM_hsa_363	chr2:174887565-174887631	+	CTTTAGGTAGGAGGCTGTTGA	Yes	Yes	No	0	0	0	No	
NM_hsa_364	chr2:174887562-174887628	-	AACAGCCTCCTACCTAAAGCA	Yes	No	No	0	0	0	No	
NM_hsa_364	chr2:174887565-174887631	+	AACAGCCTCCTACCTAAAGCA	Yes	No	No	0	0	0	No	
NM_hsa_367	chr1:1283476-1283563	+	TGGGACTAGGGACTCAGGTTACT	Yes	Yes	No	0	0	0	No	
NM_hsa_367	chr1:1283445-1283499	+	TGGGACTAGGGACTCAGGTTACT	Yes	Yes	No	0	0	0	No	
NM_hsa_367	chr1:1283432-1283508	+	TGGGACTAGGGACTCAGGTTACT	Yes	Yes	No	0	0	0	No	
NM_hsa_368	chr1:1283476-1283563	+	GCTCCTGCTCCCTGTCCCAGA	Yes	No	No	0	0	0	No	
NM_hsa_369	chr8:49427253-49427297	+	ATGGATAGCTGGGCTTGAG	Yes	No	No	0	0	0	No	
NM_hsa_369	chr8:49427212-49427272	+	ATGGATAGCTGGGCTTGAG	Yes	No	No	0	0	0	No	
NM_hsa_37	chr1:52344884-52344922	+	CTACACCCGCCGGATGGA	Yes	No	No	0.0569857	0	0.0732862	No	
NM_hsa_370	chr8:49427212-49427272	+	GCGGCTCCTGCTCCGGGG	Yes	No	No	0	0	0	No	
NM_hsa_371	chr3:143136911-143137029	+	TGCCCAGATTATCACTCACTAG	Yes	Yes	Yes	0	0	0	No	
NM_hsa_371	chr3:143136931-143137003	+	TGCCCAGATTATCACTCACTAG	Yes	Yes	Yes	0	0	0	No	
NM_hsa_371	chr3:143136941-143136995	+	TGCCCAGATTATCACTCACTAG	Yes	Yes	Yes	0	0	0	No	
NM_hsa_372	chr3:143136941-143136995	+	AGTAAGTGATAATCCGGGCATC	Yes	No	No	0	0	0	No	
NM_hsa_373	chr22:38198608-38198708	+	CCGTCTTTCCTCGTCTTGCCCGC	Yes	Yes	Yes	0	0	0.146572	No	
NM_hsa_373	chr22:38198626-38198689	+	CCGTCTTTCCTCGTCTTGCCCGC	Yes	Yes	Yes	0	0	0.146572	No	
NM_hsa_373	chr22:38198619-38198698	+	CCGTCTTTCCTCGTCTTGCCCGC	Yes	Yes	Yes	0	0	0.146572	No	
NM_hsa_374	chr22:38198626-38198689	+	GAGGCGAGGCGAGGCGAGGCGAGGCC	Yes	No	No	0	0	0	No	
NM_hsa_375	chr8:99306385-99306465	+	GAGGAGGCCGCGCCGCAGCCACT	Yes	Yes	No	0	0.331113	0	No	
NM_hsa_375	chr8:99306399-99306456	+	GAGGAGGCCGCGCCGCAGCCACT	Yes	Yes	No	0	0.331113	0	No	
NM_hsa_376	chr8:99306399-99306456	+	GGGCTGCGGCCGCCTCCCCGC	Yes	No	No	0	0	0	No	
NM_hsa_377	chr21:36824270-36824331	-	TGAAACTCTGAGCAAGAACTC	Yes	Yes	Yes	0	0	0	No	
NM_hsa_377	chr21:36824236-36824362	-	TGAAACTCTGAGCAAGAACTC	Yes	Yes	Yes	0	0	0	No	
NM_hsa_377	chr21:36824261-36824344	-	TGAAACTCTGAGCAAGAACTC	Yes	Yes	Yes	0	0	0	No	
NM_hsa_378	chr21:36824270-36824331	-	GTTCTTGCTCAGAGTTTCATG	Yes	No	No	0	0	0	No	
NM_hsa_379	chr3:17783606-17783678	-	TGCCTGCTTTCCCTGCCCCAGG	Yes	Yes	No	0	0	0	Yes	
NM_hsa_379	chr3:17783597-17783693	-	TGCCTGCTTTCCCTGCCCCAGG	Yes	Yes	No	0	0	0	Yes	
NM_hsa_38	chr1:52344884-52344922	+	AGAACGCCGGAAGTATTT	Yes	No	No	0	0	0	No	
NM_hsa_380	chr3:17783606-17783678	-	TGGCGGGCAGGGCGGGCAGGGCGAG	Yes	No	No	0	0	0	No	
NM_hsa_383	chr19:50004045-50004123	+	TGTCCCCCAGGCCTGTACCAG	Yes	Yes	No	0.113971	0	0	No	
NM_hsa_383	chr19:50004055-50004115	+	TGTCCCCCAGGCCTGTACCAG	Yes	Yes	No	0.113971	0	0	No	
NM_hsa_384	chr19:50004055-50004115	+	GGTACAAGGGTTGGGAGACAGG	Yes	No	No	0	0	0	Yes	
NM_hsa_385	chr4:141073442-141073502	+	TTCCCCGCTTCCCCCCTAGGG	Yes	Yes	No	0	0.0662225	0	Yes	
NM_hsa_385	chr4:141073430-141073511	+	TTCCCCGCTTCCCCCCTAGGG	Yes	Yes	No	0	0.0662225	0	Yes	
NM_hsa_386	chr4:141073442-141073502	+	AAAGGGGGTGAGCTGGGGAGA	Yes	No	No	0.170957	0	0	No	
NM_hsa_387	chr20:62321023-62321105	+	CTGGGGTAAGGCGGTCTGGTGA	Yes	No	No	0	0	0	Yes	
NM_hsa_388	chr20:62321023-62321105	+	ACCGAGCCGCCTCGCCCCACAGTT	Yes	No	No	0	0	0	No	
NM_hsa_389	chr3:43220997-43221078	+	TTCCCAAGTGTCTTGGACCAGG	Yes	Yes	No	0	0	0	No	
NM_hsa_389	chr3:43221007-43221070	+	TTCCCAAGTGTCTTGGACCAGG	Yes	Yes	No	0	0	0	No	
NM_hsa_39	chr12:76049413-76049495	+	TTTGCCTTGGTCTTCAGAGAT	Yes	Yes	No	0	0	0	No	
NM_hsa_39	chr12:76049423-76049486	+	TTTGCCTTGGTCTTCAGAGAT	Yes	Yes	No	0	0	0	No	
NM_hsa_39	chr12:76049395-76049444	+	TTTGCCTTGGTCTTCAGAGAT	Yes	Yes	No	0	0	0	No	
NM_hsa_390	chr3:43221007-43221070	+	TGGTCCAAAATATTTGGGAAAT	Yes	No	No	0	0	0	No	
NM_hsa_391	chr5:133840103-133840189	-	TTGCTGGGCTTAGGGACTGTGT	Yes	Yes	Yes	0	0	0	No	
NM_hsa_391	chr5:133840081-133840219	-	TTGCTGGGCTTAGGGACTGTGT	Yes	Yes	Yes	0	0	0	No	
NM_hsa_391	chr5:133840111-133840179	-	TTGCTGGGCTTAGGGACTGTGT	Yes	Yes	Yes	0	0	0	No	
NM_hsa_392	chr5:133840111-133840179	-	ATGGTCTCCAAGCCTAGCAAAC	Yes	No	No	0	0	0	No	
NM_hsa_395	chr22:36309654-36309740	+	TAGATATCATAGAAATTCCAG	Yes	Yes	Yes	0	0	0	Yes	
NM_hsa_395	chr22:36309639-36309765	+	TAGATATCATAGAAATTCCAG	Yes	Yes	Yes	0	0	0	Yes	
NM_hsa_395	chr22:36309664-36309728	-	TAGATATCATAGAAATTCCAG	Yes	Yes	Yes	0	0	0	Yes	
NM_hsa_395	chr22:36309667-36309731	+	TAGATATCATAGAAATTCCAG	Yes	Yes	Yes	0	0	0	Yes	
NM_hsa_396	chr22:36309664-36309728	-	GGAATTTCTATGATATCTATA	Yes	No	No	0	0	0	No	
NM_hsa_396	chr22:36309667-36309731	+	GGAATTTCTATGATATCTATA	Yes	No	No	0	0	0	No	
NM_hsa_398	chr16:4588680-4588751	-	GTGAGTCGACTGGACGCATTCG	Yes	No	No	0	0	0	No	
NM_hsa_399	chr2:70263108-70263246	-	AACTTTGAGGCTGAATATCTGA	Yes	Yes	Yes	0	0	0	Yes	
NM_hsa_399	chr2:70263137-70263206	-	AACTTTGAGGCTGAATATCTGA	Yes	Yes	Yes	0	0	0	Yes	
NM_hsa_399	chr2:70263140-70263209	+	AACTTTGAGGCTGAATATCTGA	Yes	Yes	Yes	0	0	0	Yes	
NM_hsa_399	chr2:70263115-70263223	+	AACTTTGAGGCTGAATATCTGA	Yes	Yes	Yes	0	0	0	Yes	
NM_hsa_399	chr2:70263129-70263216	-	AACTTTGAGGCTGAATATCTGA	Yes	Yes	Yes	0	0	0	Yes	
NM_hsa_40	chr12:76049397-76049515	+	CTCTGAAGACCAAGTCAGAGGC	Yes	Yes	Yes	0	0	0	No	
NM_hsa_40	chr12:76049413-76049495	+	CTCTGAAGACCAAGTCAGAGGC	Yes	Yes	Yes	0	0	0	No	
NM_hsa_40	chr12:76049423-76049486	+	CTCTGAAGACCAAGTCAGAGGC	Yes	Yes	Yes	0	0	0	No	
NM_hsa_400	chr2:70263137-70263206	-	AGATATTCAGCCTCAAAGTTCA	Yes	No	No	0	0	0	No	
NM_hsa_400	chr2:70263140-70263209	+	AGATATTCAGCCTCAAAGTTCA	Yes	No	No	0	0	0	No	
NM_hsa_401	chr17:76553876-76553974	+	TAAGATCTCCAAGTGGAACAGA	Yes	Yes	No	0	0	0	No	
NM_hsa_401	chr17:76553888-76553965	+	TAAGATCTCCAAGTGGAACAGA	Yes	Yes	No	0	0	0	No	
NM_hsa_402	chr17:76553888-76553965	+	TGTTCTGCTTGGAGATCTTACA	Yes	No	No	0	0	0	No	
NM_hsa_403	chr13:55195301-55195363	+	AGAGGGACCAGAACATACTGTG	Yes	Yes	Yes	0	0	0	Yes	
NM_hsa_403	chr13:55195298-55195360	-	AGAGGGACCAGAACATACTGTG	Yes	Yes	Yes	0	0	0	Yes	
NM_hsa_403	chr13:55195291-55195371	+	AGAGGGACCAGAACATACTGTG	Yes	Yes	Yes	0	0	0	Yes	
NM_hsa_403	chr13:55195262-55195400	-	AGAGGGACCAGAACATACTGTG	Yes	Yes	Yes	0	0	0	Yes	
NM_hsa_404	chr13:55195298-55195360	-	CAGTATGTTCTGGTCCCTCTAG	Yes	No	No	0	0	0	No	
NM_hsa_407	chr22:41048556-41048664	+	CTACCTCCTGCCCTTCCCACCG	Yes	Yes	Yes	0	0	0	Yes	
NM_hsa_407	chr22:41048574-41048640	+	CTACCTCCTGCCCTTCCCACCG	Yes	Yes	Yes	0	0	0	Yes	
NM_hsa_407	chr22:41048563-41048649	+	CTACCTCCTGCCCTTCCCACCG	Yes	Yes	Yes	0	0	0	Yes	
NM_hsa_408	chr22:41048574-41048640	+	GTGGGGTCGGGCAGGAGGGCCGGGG	Yes	No	No	0	0	0	No	
NM_hsa_409	chr8:103818795-103818931	+	GCGTCTGATGTCAGCCTGGAG	Yes	No	Yes	0	0	0	No	
NM_hsa_409	chr8:103818834-103818892	+	GCGTCTGATGTCAGCCTGGAG	Yes	No	Yes	0	0	0	No	
NM_hsa_41	chr3:128735318-128735402	+	AGGCAGGTGGAGCTCATGGGCA	Yes	No	No	0.341914	0.0331113	0.0732862	No	
NM_hsa_410	chr8:103818834-103818892	+	TCGGAAACTGTTGACTCAGAGGGTG	Yes	No	No	0	0	0	No	
NM_hsa_411	chr11:133231459-133231531	-	TGCAGGTGCCCCGGAGTAG	Yes	No	No	0	0	0	No	
NM_hsa_412	chr11:133231459-133231531	-	GCCCCGGAATAGCTGCAGG	Yes	No	No	0	0	0	No	
NM_hsa_415	chr11:1857597-1857655	+	CTGGCTCACGCCGCTGCCTCAG	Yes	No	Yes	0	0	0	No	
NM_hsa_415	chr11:1857556-1857694	+	CTGGCTCACGCCGCTGCCTCAG	Yes	No	Yes	0	0	0	No	
NM_hsa_416	chr11:1857597-1857655	+	GGGCAGCTGGGTGGGCCTGAGCTAGGG	Yes	No	No	0	0	0	No	
NM_hsa_417	chr12:29935783-29935923	+	CTCCTGAACCCACGCAGCCCTGT	Yes	Yes	Yes	0	0	0	No	
NM_hsa_417	chr12:29935825-29935884	+	CTCCTGAACCCACGCAGCCCTGT	Yes	Yes	Yes	0	0	0	No	
NM_hsa_417	chr12:29935809-29935893	+	CTCCTGAACCCACGCAGCCCTGT	Yes	Yes	Yes	0	0	0	No	
NM_hsa_418	chr12:29935825-29935884	+	GGGGGCTGGGGGGCTCGGGAGAG	Yes	No	No	0	0	0	No	
NM_hsa_419	chr19:4640481-4640556	+	TTTGAGCTCTGCCACCCGCAT	Yes	Yes	No	0	0	0	No	
NM_hsa_419	chr19:4640498-4640547	+	TTTGAGCTCTGCCACCCGCAT	Yes	Yes	No	0	0	0	No	
NM_hsa_42	chr3:128735318-128735402	+	CCCGTGAGCTCCACCTGCCTGC	Yes	No	No	0.113971	0.132445	0	No	
NM_hsa_420	chr19:4640498-4640547	+	GTGGTGGCGGAGCTTGGACC	Yes	No	No	0	0	0	No	
NM_hsa_421	chr19:57950479-57950542	+	TTAGTGGCTCCCTCTGCCTGCA	Yes	Yes	Yes	1.13971	0.79467	1.09929	Yes	
NM_hsa_421	chr19:57950444-57950572	+	TTAGTGGCTCCCTCTGCCTGCA	Yes	Yes	Yes	1.13971	0.79467	1.09929	Yes	
NM_hsa_421	chr19:57950469-57950549	+	TTAGTGGCTCCCTCTGCCTGCA	Yes	Yes	Yes	1.13971	0.79467	1.09929	Yes	
NM_hsa_422	chr19:57950479-57950542	+	CAGGTGAGGGTGGAGCCACCGATA	Yes	No	No	0	0	0	Yes	
NM_hsa_423	chr9:140117395-140117456	+	CGCTGCCACGCTGTGACCAGC	Yes	Yes	No	0	0	0	No	
NM_hsa_423	chr9:140117385-140117465	+	CGCTGCCACGCTGTGACCAGC	Yes	Yes	No	0	0	0	No	
NM_hsa_424	chr9:140117395-140117456	+	TGGTCACCGTGAGGCAGGGGA	Yes	No	No	0	0	0	No	
NM_hsa_425	chr3:118599704-118599760	-	AGTGATGGGGGCCGATAGA	Yes	No	No	0	0	0	No	
NM_hsa_426	chr3:118599704-118599760	-	TATCAGCCCCCATCACTTG	Yes	No	No	0	0	0	No	
NM_hsa_427	chr1:2266897-2266960	+	CTGGAGCCCTCCTCTGCCCCT	Yes	No	No	0	0	0	No	
NM_hsa_427	chr1:2266835-2266918	+	CTGGAGCCCTCCTCTGCCCCT	Yes	No	No	0	0	0	No	
NM_hsa_428	chr1:2266835-2266918	+	AGGCAGAGGAGGTCTCGAGGGC	Yes	No	No	0	0	0	No	
NM_hsa_429	chr15:85697762-85697823	+	TTTCCTAATCACATATTGCTTC	Yes	Yes	Yes	0	0	0	Yes	
NM_hsa_429	chr15:85697722-85697860	+	TTTCCTAATCACATATTGCTTC	Yes	Yes	Yes	0	0	0	Yes	
NM_hsa_429	chr15:85697752-85697831	+	TTTCCTAATCACATATTGCTTC	Yes	Yes	Yes	0	0	0	Yes	
NM_hsa_43	chr5:156607859-156607902	+	AGGCAAGTTGCCCTTGAGCAG	Yes	No	No	0	0	0	No	
NM_hsa_430	chr15:85697762-85697823	+	AGCAATGTGTGATTAGGAAAAG	Yes	No	No	0	0	0	No	
NM_hsa_431	chr17:29421366-29421443	+	CACCGGGTCTAGCATTGGGAT	Yes	Yes	No	0	0	0	Yes	
NM_hsa_431	chr17:29421378-29421434	+	CACCGGGTCTAGCATTGGGAT	Yes	Yes	No	0	0	0	Yes	
NM_hsa_432	chr17:29421378-29421434	+	CCCAATGCTAGACCTGGTGGC	Yes	No	No	0	0	0	No	
NM_hsa_433	chr4:11784804-11784873	+	AGACCTCTGAACTCTATGTGGG	Yes	Yes	Yes	0	0	0	No	
NM_hsa_433	chr4:11784789-11784877	+	AGACCTCTGAACTCTATGTGGG	Yes	Yes	Yes	0	0	0	No	
NM_hsa_433	chr4:11784794-11784889	+	AGACCTCTGAACTCTATGTGGG	Yes	Yes	Yes	0	0	0	No	
NM_hsa_434	chr4:11784804-11784873	+	CACAGAGAGTTCAGAGGTCTAA	Yes	No	No	0	0	0	No	
NM_hsa_435	chr1:8324253-8324332	+	TCTGTGTTTTGGACATCTCCA	Yes	No	No	0	0	0	No	
NM_hsa_436	chr1:8324253-8324332	+	GAGATGTCCAAAGCACAGATT	Yes	No	No	0	0	0	No	
NM_hsa_437	chr5:155254269-155254369	+	TTCAGGAGTGGGGACATCAGGGA	Yes	Yes	Yes	0	0	0	No	
NM_hsa_437	chr5:155254279-155254354	+	TTCAGGAGTGGGGACATCAGGGA	Yes	Yes	Yes	0	0	0	No	
NM_hsa_437	chr5:155254289-155254346	+	TTCAGGAGTGGGGACATCAGGGA	Yes	Yes	Yes	0	0	0	No	
NM_hsa_438	chr5:155254289-155254346	+	CCTGGTGCATCCACTTCTGAACT	Yes	No	No	0	0	0	No	
NM_hsa_44	chr5:156607859-156607902	+	CTCTGAAGATCAACTGCCTC	Yes	No	No	0	0	0	No	
NM_hsa_441	chr18:29010523-29010606	+	TCAGGTTTGCATTCCAGATGTG	Yes	No	No	0	0	0	No	
NM_hsa_442	chr18:29010523-29010606	+	CATCTGAAATGCAAACCTGAAC	Yes	No	No	0	0	0	No	
NM_hsa_443	chr8:145552437-145552498	+	CTCAGAGAGGTCTGGACCTGG	Yes	Yes	Yes	0	0	0	No	
NM_hsa_443	chr8:145552416-145552522	+	CTCAGAGAGGTCTGGACCTGG	Yes	Yes	Yes	0	0	0	No	
NM_hsa_443	chr8:145552425-145552507	+	CTCAGAGAGGTCTGGACCTGG	Yes	Yes	Yes	0	0	0	No	
NM_hsa_444	chr8:145552437-145552498	+	AGGTAGGCCCAGACCTGTCTGAGTA	Yes	No	No	0	0	0	No	
NM_hsa_445	chr6:41395709-41395802	+	CTGGTGGTGGCTCTCGACCTGC	Yes	Yes	No	0	0	0	No	
NM_hsa_445	chr6:41395725-41395793	+	CTGGTGGTGGCTCTCGACCTGC	Yes	Yes	No	0	0	0	No	
NM_hsa_446	chr6:41395725-41395793	+	AGATCTGTCGAGGGGCACCACCAGGG	Yes	No	No	0	0	0	No	
NM_hsa_448	chr11:66247657-66247700	-	CACACGCGCCGGACCGGG	Yes	No	No	0	0	0	No	
NM_hsa_450	chr13:55195301-55195363	+	CAGTATGTTCTGGTCCCTCTGG	Yes	No	No	0	0	0	No	
NM_hsa_451	chr15:78100655-78100739	+	AGGCCTTTGCCCAGATACAGG	Yes	Yes	Yes	0	0	0	No	
NM_hsa_451	chr15:78100665-78100725	+	AGGCCTTTGCCCAGATACAGG	Yes	Yes	Yes	0	0	0	No	
NM_hsa_451	chr15:78100630-78100756	+	AGGCCTTTGCCCAGATACAGG	Yes	Yes	Yes	0	0	0	No	
NM_hsa_452	chr15:78100665-78100725	+	TGTATCGGGGCAAAGGCCTTG	Yes	No	No	0	0	0	No	
NM_hsa_453	chr2:220110242-220110325	+	CAGGTCAGTCCGGCAGTG	Yes	No	No	0	0	0	No	
NM_hsa_454	chr2:220110242-220110325	+	CTGCCAGGAGTGGGCCTGAG	Yes	No	No	0	0	0	No	
NM_hsa_455	chr1:180768880-180768959	+	TAGCTGCATGCCAGGGGCTAGA	Yes	Yes	Yes	0.341914	0	0.0732862	Yes	
NM_hsa_455	chr1:180768894-180768950	+	TAGCTGCATGCCAGGGGCTAGA	Yes	Yes	Yes	0.341914	0	0.0732862	Yes	
NM_hsa_455	chr1:180768866-180768974	+	TAGCTGCATGCCAGGGGCTAGA	Yes	Yes	Yes	0.341914	0	0.0732862	Yes	
NM_hsa_456	chr1:180768894-180768950	+	TGCTGCCTGGTGAGTAGCTGCA	Yes	No	No	0	0	0	No	
NM_hsa_457	chr1:1283445-1283499	+	AGGCCTGAGCCCGCCCCCAGC	Yes	No	No	0	0	0	No	
NM_hsa_458	chr16:28331334-28331398	+	CTTCCCCCGGCCGCCCGCAGG	Yes	No	No	0	0.132445	0	No	
NM_hsa_459	chr16:28331334-28331398	+	GGAGGGCGGCGGGGCGGGCGCT	Yes	No	No	0.0189952	0.110371	0.0183216	Yes	
NM_hsa_460	chr11:130442135-130442209	+	CTTGCACTGGACTGGGAATGA	Yes	Yes	No	0	0	0	No	
NM_hsa_460	chr11:130442145-130442201	+	CTTGCACTGGACTGGGAATGA	Yes	Yes	No	0	0	0	No	
NM_hsa_461	chr11:130442145-130442201	+	ATTCCCAGTCCAGTGCAAAAG	Yes	No	No	0	0	0	No	
NM_hsa_464	chr4:125105686-125105777	-	TAAATGTGGAATTCTAAGCAT	Yes	Yes	No	0	0	0	No	
NM_hsa_464	chr4:125105695-125105764	-	TAAATGTGGAATTCTAAGCAT	Yes	Yes	No	0	0	0	No	
NM_hsa_465	chr4:125105695-125105764	-	GCTTACAATTCCACATTTATT	Yes	No	No	0	0	0	No	

Additional file 5

Name	locus	strand	sequence	mirdeep2	mireap	miranalyzer	RPM_HU_KapB_cMyc	RPM_HU_KaposinB	RPM_HUVEC	IP_confidence	annotation
NM_hsa_466	chr10:129535431-129535515	+	TCGCCAGTGAGTTTCGGTGCGG	Yes	Yes	No	0	0	0	No	
NM_hsa_466	chr10:129535449-129535506	+	TCGCCAGTGAGTTTCGGTGCGG	Yes	Yes	No	0	0	0	No	
NM_hsa_467	chr10:129535449-129535506	+	GCACCCGGGCGCTGCTGGCGGCC	Yes	No	No	0	0	0	No	
NM_hsa_468	chr18:30350208-30350271	+	AGCAGCTTGTCGTCGGGGGAGG	Yes	No	No	0	0	0	No	
NM_hsa_469	chr18:30350208-30350271	+	TCCTGCGGCGGCGGCTGCTGC	Yes	No	No	0	0	0.146572	No	
NM_hsa_47	chr2:188104481-188104551	+	ATGATCTGCATGCTCTGGACC	Yes	Yes	No	0	0	0	No	
NM_hsa_47	chr2:188104471-188104560	+	ATGATCTGCATGCTCTGGACC	Yes	Yes	No	0	0	0	No	
NM_hsa_470	chr1:237736806-237736886	-	GAACAACCCTATCCTCTTCCAG	Yes	Yes	Yes	0	0	0	Yes	
NM_hsa_470	chr1:237736815-237736874	-	GAACAACCCTATCCTCTTCCAG	Yes	Yes	Yes	0	0	0	Yes	
NM_hsa_470	chr1:237736803-237736891	+	GAACAACCCTATCCTCTTCCAG	Yes	Yes	Yes	0	0	0	Yes	
NM_hsa_470	chr1:237736818-237736877	+	GAACAACCCTATCCTCTTCCAG	Yes	Yes	Yes	0	0	0	Yes	
NM_hsa_470	chr1:237736791-237736899	-	GAACAACCCTATCCTCTTCCAG	Yes	Yes	Yes	0	0	0	Yes	
NM_hsa_471	chr1:237736818-237736877	+	GGAAGAGGATAGGGTTGTTCTA	Yes	No	No	0	0	0	No	
NM_hsa_472	chr13:27259465-27259541	+	TAGCCACTAGCCACATGTCAGTT	Yes	Yes	Yes	0	0.132445	0	Yes	
NM_hsa_472	chr13:27259475-27259533	+	TAGCCACTAGCCACATGTCAGTT	Yes	Yes	Yes	0	0.132445	0	Yes	
NM_hsa_472	chr13:27259440-27259570	+	TAGCCACTAGCCACATGTCAGTT	Yes	Yes	Yes	0	0.132445	0	Yes	
NM_hsa_473	chr13:27259475-27259533	+	CTATATGTGCCTAGTGGCTGCT	Yes	No	No	0	0.397335	0	Yes	
NM_hsa_474	chr1:22170792-22170843	-	CTGACCTCTCCCCTGTGCCCCAG	Yes	No	No	0.683829	0.0662225	0.219859	No	
NM_hsa_475	chr1:22170792-22170843	-	GAGGGCATCAGGGTGGCATCTCAGCT	Yes	No	No	0	0	0	No	
NM_hsa_476	chr8:120220605-120220658	+	CCCGGAGCCCGCGGAGCTGAG	Yes	No	No	0	0	0.0732862	No	
NM_hsa_478	chr11:76156399-76156480	+	TATCCGCTGGCTTACTCTCTCT	Yes	Yes	Yes	0.626843	0.0662225	0.879435	Yes	
NM_hsa_478	chr11:76156409-76156473	+	TATCCGCTGGCTTACTCTCTCT	Yes	Yes	Yes	0.626843	0.0662225	0.879435	Yes	
NM_hsa_478	chr11:76156374-76156502	+	TATCCGCTGGCTTACTCTCTCT	Yes	Yes	Yes	0.626843	0.0662225	0.879435	Yes	
NM_hsa_479	chr11:76156409-76156473	+	AGGGAGTGAGGCGCGGGTGCC	Yes	No	No	0	0	0	No	
NM_hsa_48	chr2:188104481-188104551	+	AACAGAGTACACCAGATCACCA	Yes	Yes	Yes	0	0	0	No	
NM_hsa_48	chr2:188104446-188104574	+	AACAGAGTACACCAGATCACCA	Yes	Yes	Yes	0	0	0	No	
NM_hsa_48	chr2:188104471-188104560	+	AACAGAGTACACCAGATCACCA	Yes	Yes	Yes	0	0	0	No	
NM_hsa_480	chr1:55784366-55784417	+	AGCCTCCCAGTCTGGCCTGAG	Yes	Yes	No	0	0	0	No	
NM_hsa_480	chr1:55784352-55784426	+	AGCCTCCCAGTCTGGCCTGAG	Yes	Yes	No	0	0	0	No	
NM_hsa_481	chr1:55784366-55784417	+	CAGGCCAGGCTGGGAGGATGC	Yes	No	No	0	0	0	No	
NM_hsa_482	chr11:133012742-133012794	+	CTGGGCAAAGAACTCCAAGTGC	Yes	Yes	No	0	0	0	No	
NM_hsa_482	chr11:133012730-133012803	+	CTGGGCAAAGAACTCCAAGTGC	Yes	Yes	No	0	0	0	No	
NM_hsa_483	chr11:133012742-133012794	+	ACTTGGAGGTCTCTGCCCATAC	Yes	No	No	0	0	0	No	
NM_hsa_484	chr3:175325984-175326074	+	AGATTGTGGACTATGGGCTAAT	Yes	Yes	No	0	0	0	No	
NM_hsa_484	chr3:175325997-175326065	+	AGATTGTGGACTATGGGCTAAT	Yes	Yes	No	0	0	0	No	
NM_hsa_484	chr3:175325994-175326062	-	AGATTGTGGACTATGGGCTAAT	Yes	Yes	No	0	0	0	No	
NM_hsa_485	chr3:175325997-175326065	+	TAGCCCATAGTCCACAATCTGA	Yes	No	No	0	0	0	No	
NM_hsa_485	chr3:175325994-175326062	-	TAGCCCATAGTCCACAATCTGA	Yes	No	No	0	0	0	No	
NM_hsa_488	chr17:59438889-59438965	-	CCTGTGGACACATCTGATGGC	Yes	Yes	No	0	0	0	No	
NM_hsa_488	chr17:59438880-59438977	-	CCTGTGGACACATCTGATGGC	Yes	Yes	No	0	0	0	No	
NM_hsa_489	chr17:59438889-59438965	-	CATCAGATGTGTCCACAGATA	Yes	No	No	0	0	0	No	
NM_hsa_490	chr7:29883563-29883636	+	CTTGGCAGCCAGGAAGTCACG	Yes	Yes	No	0	0	0	No	
NM_hsa_490	chr7:29883548-29883645	+	CTTGGCAGCCAGGAAGTCACG	Yes	Yes	No	0	0	0	No	
NM_hsa_491	chr7:29883563-29883636	+	TGACTTCCTGGCTGCCAAGCC	Yes	No	No	0	0	0	No	
NM_hsa_492	chr22:42816210-42816301	+	ACTTTAAGATCAGCCTGCAGA	Yes	Yes	No	0	0	0	No	
NM_hsa_494	chr17:42993683-42993773	+	TCTTGCCTTATCAGGACTTTAGC	Yes	Yes	Yes	0	0	0	No	
NM_hsa_494	chr17:42993701-42993759	+	TCTTGCCTTATCAGGACTTTAGC	Yes	Yes	Yes	0	0	0	No	
NM_hsa_494	chr17:42993685-42993768	+	TCTTGCCTTATCAGGACTTTAGC	Yes	Yes	Yes	0	0	0	No	
NM_hsa_495	chr17:42993701-42993759	+	GGAGGTCCTGATAGGGCAAGAGG	Yes	No	No	0	0	0	No	
NM_hsa_498	chrX:126451083-126451191	-	TCCTTTCAACCACCTCCAGAGG	Yes	Yes	Yes	0	0	0	Yes	
NM_hsa_498	chrX:126451108-126451166	-	TCCTTTCAACCACCTCCAGAGG	Yes	Yes	Yes	0	0	0	Yes	
NM_hsa_498	chrX:126451099-126451176	-	TCCTTTCAACCACCTCCAGAGG	Yes	Yes	Yes	0	0	0	Yes	
NM_hsa_498	chrX:126451086-126451194	+	TCCTTTCAACCACCTCCAGAGG	Yes	Yes	Yes	0	0	0	Yes	
NM_hsa_499	chrX:126451108-126451166	-	TCTGGAGGTGGTTGAAAGGAAA	Yes	No	No	0	0	0	No	
NM_hsa_500	chr5:137673931-137674003	+	CTGGAGGAACCGCGGTAGGT	Yes	No	No	0	0	0	No	
NM_hsa_502	chr2:43454591-43454641	+	GCCGTGCGGGCTTGAAGG	Yes	No	No	0	0	0	No	
NM_hsa_503	chr2:43454591-43454641	+	TTCCGTCTGCTGGTCG	Yes	No	No	0	0	0	No	
NM_hsa_504	chr9:7967909-7967992	+	TTAGGAATGTGGGCTCTGGATT	Yes	Yes	No	0	0	0	No	
NM_hsa_504	chr9:7967921-7967983	+	TTAGGAATGTGGGCTCTGGATT	Yes	Yes	No	0	0	0	No	
NM_hsa_505	chr9:7967921-7967983	+	TCCAGATCCCACATTCCTAACC	Yes	No	No	0	0	0	No	
NM_hsa_506	chr4:141677685-141677756	+	CCGCGGCGCCGACTGGAGCTCC	Yes	No	No	0	0.132445	0.0732862	No	
NM_hsa_507	chr4:141677685-141677756	+	AGCTCCCGAAGTCGCCGCGCCT	Yes	No	No	0	0.132445	0	No	
NM_hsa_508	chr8:18961076-18961133	+	CTCCTGGAGATTCTGAAGTACA	Yes	Yes	Yes	0	0.132445	0	Yes	
NM_hsa_508	chr8:18961039-18961167	+	CTCCTGGAGATTCTGAAGTACA	Yes	Yes	Yes	0	0.132445	0	Yes	
NM_hsa_508	chr8:18961064-18961142	+	CTCCTGGAGATTCTGAAGTACA	Yes	Yes	Yes	0	0.132445	0	Yes	
NM_hsa_509	chr8:18961076-18961133	+	TACTTTAGAATCTCCAGGACTA	Yes	No	No	0	0	0	No	
NM_hsa_51	chr8:54111682-54111808	+	CATGTGAACTCACTTTTCACA	Yes	Yes	Yes	0	0	0	Yes	
NM_hsa_51	chr8:54111717-54111776	+	CATGTGAACTCACTTTTCACA	Yes	Yes	Yes	0	0	0	Yes	
NM_hsa_51	chr8:54111707-54111785	+	CATGTGAACTCACTTTTCACA	Yes	Yes	Yes	0	0	0	Yes	
NM_hsa_510	chr20:3748716-3748807	+	CTTGAACCTTGCTTAGGACG	Yes	Yes	No	0	0	0	No	
NM_hsa_510	chr20:3748726-3748799	+	CTTGAACCTTGCTTAGGACG	Yes	Yes	No	0	0	0	No	
NM_hsa_511	chr20:3748726-3748799	+	TCGGGCGCGTCTCGGGGC	Yes	No	No	0	0	0	No	
NM_hsa_512	chr8:126441993-126442070	-	GGGCGCGAGGCGGGAGCGCGCA	Yes	Yes	No	0	0	0	No	
NM_hsa_512	chr8:126442038-126442095	-	GGGCGCGAGGCGGGAGCGCGCA	Yes	Yes	No	0	0	0	No	
NM_hsa_513	chr8:126442038-126442095	-	CGCGCGCCTCATTCACAGTAAGC	Yes	No	No	0	0	0	No	
NM_hsa_514	chr22:50744254-50744313	-	AGGAGCTCTGGGCCCACCCA	Yes	No	No	0	0	0	No	
NM_hsa_515	chr22:50744254-50744313	-	AGTGGGCCCAAGCCCCTC	Yes	No	No	0	0	0	No	
NM_hsa_518	chr10:102757535-102757604	+	TGGTCCCAGGCCGGACGG	Yes	No	No	0	0	0	No	
NM_hsa_519	chr10:102757535-102757604	+	CTCTCCCGGGAACCGGAG	Yes	No	No	0	0	0	No	
NM_hsa_52	chr8:54111689-54111805	+	TGAAAAGTGGGTTCATAGGGA	Yes	Yes	Yes	0	0	0	No	
NM_hsa_52	chr8:54111717-54111776	+	TGAAAAGTGGGTTCATAGGGA	Yes	Yes	Yes	0	0	0	No	
NM_hsa_52	chr8:54111707-54111785	+	TGAAAAGTGGGTTCATAGGGA	Yes	Yes	Yes	0	0	0	No	
NM_hsa_520	chr19:5978291-5978403	+	CATCTCCCGCCTCCTTTTCCCGCC	Yes	Yes	Yes	0.0569857	0.331113	0	Yes	
NM_hsa_520	chr19:5978306-5978381	+	CATCTCCCGCCTCCTTTTCCCGCC	Yes	Yes	Yes	0.0569857	0.331113	0	Yes	
NM_hsa_520	chr19:5978316-5978373	+	CATCTCCCGCCTCCTTTTCCCGCC	Yes	Yes	Yes	0.0569857	0.331113	0	Yes	
NM_hsa_521	chr19:5978316-5978373	+	CGGGGAAAGGGCCGGGAAGGGC	Yes	No	No	0	0.0662225	0	No	
NM_hsa_522	chr5:179251112-179251183	+	CTCTGCTAATTCCTCCCCCAGG	Yes	No	No	0.0569857	0.0662225	0	No	
NM_hsa_523	chr5:179251112-179251183	+	TGGGGTGGGCTCAGGGTG	Yes	No	No	0	0	0	No	
NM_hsa_524	chr8:145584178-145584244	+	TGCGGGTGGAACTCAGGGCTGG	Yes	No	No	0.0569857	0.0993338	0	No	
NM_hsa_525	chr8:145584178-145584244	+	AGCCCTGACATTCTGCTCGCTCA	Yes	No	No	0	0	0	No	
NM_hsa_526	chr22:41048069-41048125	+	AGTCACCTCCCTCCTGGCCAG	Yes	No	No	0	0	0	No	
NM_hsa_527	chr22:41048069-41048125	+	GGCTGGAGGGGAGGAGACCCA	Yes	No	No	0	0	0	No	
NM_hsa_528	chr10:82906028-82906089	+	CGAGATCTCCTGTGAACTCAGA	Yes	No	No	0.0569857	0	0	No	
NM_hsa_529	chr10:82906028-82906089	+	TGAGTTCACAGACGATCTTGTC	Yes	No	No	0	0	0	No	
NM_hsa_53	chr22:50926246-50926313	+	CACTGCCTTCTCCTTCCCCGCC	Yes	Yes	No	0	0	0	No	
NM_hsa_53	chr22:50926237-50926322	+	CACTGCCTTCTCCTTCCCCGCC	Yes	Yes	No	0	0	0	No	
NM_hsa_530	chr11:130442134-130442208	-	TTTGCACTGGACTGGGAATGA	Yes	Yes	No	0	0	0	No	
NM_hsa_530	chr11:130442142-130442198	-	TTTGCACTGGACTGGGAATGA	Yes	Yes	No	0	0	0	No	
NM_hsa_531	chr11:130442142-130442198	-	ATTCCCAGTCCAGTGCAAGAG	Yes	No	No	0	0	0	No	
NM_hsa_532	chr10:106028716-106028777	-	TGGTTGGCTGTGGGACCCC	Yes	No	No	0	0	0	No	
NM_hsa_533	chr10:106028716-106028777	-	GGTCCTGCCGGCGCCG	Yes	No	No	0	0	0	No	
NM_hsa_534	chr20:30945993-30946064	+	AGGCCGTTGGGACTACGA	Yes	No	No	0	0	0	No	
NM_hsa_535	chr20:30945993-30946064	+	GTGGTCCACGCGCTTCA	Yes	No	No	0	0	0	No	
NM_hsa_536	chrX:20285281-20285353	+	AACAGATCTGGGGGCGTCGG	Yes	No	No	0	0	0	No	
NM_hsa_537	chrX:20285281-20285353	+	GCGCCCCAACCTCAGCTTCTGG	Yes	No	No	0	0	0	Yes	
NM_hsa_538	chr2:178178530-178178613	+	CTACCATTAGAAGGGCTGGAAG	Yes	Yes	No	0	0	0	No	
NM_hsa_538	chr2:178178540-178178606	+	CTACCATTAGAAGGGCTGGAAG	Yes	Yes	No	0	0	0	No	
NM_hsa_539	chr2:178178540-178178606	+	TCCAGCTCTTCTAATGGTAAGA	Yes	No	No	0	0	0	No	
NM_hsa_54	chr22:50926246-50926313	+	AGGGGATGGAGAGGCCTGTGTGG	Yes	No	No	0	0	0	No	
NM_hsa_540	chr3:195373811-195373862	+	GTGCCTCCAGCTGCATGGCTC	Yes	No	No	0	0	0	No	
NM_hsa_540	chr3:195668491-195668542	-	GTGCCTCCAGCTGCATGGCTC	Yes	No	No	0	0	0	No	
NM_hsa_540	chr3:197365884-197365935	-	GTGCCTCCAGCTGCATGGCTC	Yes	No	No	0	0	0	No	
NM_hsa_545	chr11:66512054-66512096	-	CTCTGAGCGCCGCGGGTG	Yes	No	No	0	0	0	No	
NM_hsa_547	chr3:153106191-153106246	-	GTCAATGTCATTGTCACTGAGA	Yes	Yes	Yes	0	0	0	No	
NM_hsa_547	chr3:153106182-153106259	-	GTCAATGTCATTGTCACTGAGA	Yes	Yes	Yes	0	0	0	No	
NM_hsa_547	chr3:153106162-153106280	-	GTCAATGTCATTGTCACTGAGA	Yes	Yes	Yes	0	0	0	No	
NM_hsa_548	chr3:153106191-153106246	-	TCAGTGACAATGACATTGATTG	Yes	No	No	0	0	0	No	
NM_hsa_549	chr7:37824501-37824562	+	CAACAGAACCCAGAGAGTCAG	Yes	No	No	0	0	0	No	
NM_hsa_55	chr1:46872725-46872791	+	TGCAGACCCCAGCTCATGTAG	Yes	Yes	Yes	0	0	0	No	
NM_hsa_55	chr1:46872685-46872821	+	TGCAGACCCCAGCTCATGTAG	Yes	Yes	Yes	0	0	0	No	
NM_hsa_55	chr1:46872715-46872800	+	TGCAGACCCCAGCTCATGTAG	Yes	Yes	Yes	0	0	0	No	
NM_hsa_550	chr7:37824501-37824562	+	ACCTCTCTGGGTTCTGTTTCT	Yes	No	No	0	0	0	No	
NM_hsa_553	chr10:90957414-90957473	+	TCCATATCCCAACCTGTCAGAGT	Yes	Yes	Yes	0.113971	0.132445	0.0732862	No	
NM_hsa_553	chr10:90957399-90957489	+	TCCATATCCCAACCTGTCAGAGT	Yes	Yes	Yes	0.113971	0.132445	0.0732862	No	
NM_hsa_553	chr10:90957404-90957481	+	TCCATATCCCAACCTGTCAGAGT	Yes	Yes	Yes	0.113971	0.132445	0.0732862	No	
NM_hsa_554	chr10:90957414-90957473	+	TCTGCAGGCTGGGATCTGGGAT	Yes	No	No	0.0569857	0.198668	0	Yes	
NM_hsa_555	chr15:62699315-62699372	+	TGGAATTCTGCACCTGGTGATT	Yes	Yes	Yes	0	0	0	Yes	
NM_hsa_555	chr15:62699278-62699406	+	TGGAATTCTGCACCTGGTGATT	Yes	Yes	Yes	0	0	0	Yes	
NM_hsa_555	chr15:62699302-62699381	+	TGGAATTCTGCACCTGGTGATT	Yes	Yes	Yes	0	0	0	Yes	
NM_hsa_556	chr15:62699315-62699372	+	CCAGCAGGTGCAGAATTCCACA	Yes	No	No	0	0	0	Yes	
NM_hsa_557	chr8:143927122-143927206	+	TGCGATCGATAACCTTCACCAG	Yes	Yes	Yes	0	0	0	Yes	
NM_hsa_557	chr8:143927103-143927231	+	TGCGATCGATAACCTTCACCAG	Yes	Yes	Yes	0	0	0	Yes	
NM_hsa_557	chr8:143927135-143927197	+	TGCGATCGATAACCTTCACCAG	Yes	Yes	Yes	0	0	0	Yes	
NM_hsa_558	chr8:143927135-143927197	+	AGTGAAGATTATTGATCACATT	Yes	No	No	0	0	0	No	
NM_hsa_559	chr16:58061414-58061489	+	CTGCGGGCCTGAGCGCCGTT	Yes	Yes	No	0	0	0	No	
NM_hsa_559	chr16:58061424-58061480	+	CTGCGGGCCTGAGCGCCGTT	Yes	Yes	No	0	0	0	No	
NM_hsa_56	chr1:46872725-46872791	+	TACGTGAGCCAGGGTCTGCAAG	Yes	Yes	Yes	0	0	0	Yes	
NM_hsa_56	chr1:46872697-46872825	+	TACGTGAGCCAGGGTCTGCAAG	Yes	Yes	Yes	0	0	0	Yes	
NM_hsa_56	chr1:46872715-46872800	+	TACGTGAGCCAGGGTCTGCAAG	Yes	Yes	Yes	0	0	0	Yes	
NM_hsa_560	chr16:58061424-58061480	+	CGGTGCTCAGGCCTTGGTGCC	Yes	No	No	0	0	0	No	
NM_hsa_561	chr2:242675456-242675510	+	TTGCCTGGCGCAGTGGCTCTGC	Yes	No	No	0.3989	0.397335	0.58629	Yes	
NM_hsa_562	chr2:242675456-242675510	+	GTGAGCCACCCCGCCCTGCCAAC	Yes	No	No	0	0	0	No	
NM_hsa_563	chr1:147829273-147829393	+	ACACTCATCAACTGTGCCTGACA	Yes	Yes	Yes	0	0.26489	0	Yes	
NM_hsa_563	chr1:147829303-147829365	+	ACACTCATCAACTGTGCCTGACA	Yes	Yes	Yes	0	0.26489	0	Yes	
NM_hsa_563	chr1:147829293-147829372	+	ACACTCATCAACTGTGCCTGACA	Yes	Yes	Yes	0	0.26489	0	Yes	
NM_hsa_563	chr1:147803724-147803864	-	ACACTCATCAACTGTGCCTGACA	Yes	Yes	Yes	0	0.26489	0	Yes	
NM_hsa_563	chr1:147803763-147803824	-	ACACTCATCAACTGTGCCTGACA	Yes	Yes	Yes	0	0.26489	0	Yes	
NM_hsa_564	chr1:147803763-147803824	-	GCAGGCACAGTTGATGATGTGC	Yes	No	No	0	0	0	No	
NM_hsa_565	chr2:155586576-155586629	-	TGCTTTCTGCTTATAACTCTGA	Yes	Yes	Yes	0	0	0	No	
NM_hsa_565	chr2:155586567-155586665	-	TGCTTTCTGCTTATAACTCTGA	Yes	Yes	Yes	0	0	0	No	
NM_hsa_565	chr2:155586579-155586632	+	TGCTTTCTGCTTATAACTCTGA	Yes	Yes	Yes	0	0	0	No	
NM_hsa_565	chr2:155586552-155586660	-	TGCTTTCTGCTTATAACTCTGA	Yes	Yes	Yes	0	0	0	No	
NM_hsa_565	chr2:155586538-155586666	+	TGCTTTCTGCTTATAACTCTGA	Yes	Yes	Yes	0	0	0	No	
NM_hsa_566	chr2:155586579-155586632	+	AGAGTTATAAGCAGAAAGCAGT	Yes	No	No	0	0	0	No	
NM_hsa_567	chr22:34199986-34200072	+	TGGCAGTCAGGATTGTTATGT	Yes	No	No	0	0	0	No	
NM_hsa_568	chr22:34199986-34200072	+	ACAGCAATCCTGGCTGCCATC	Yes	No	No	0	0	0	No	
NM_hsa_569	chr7:38351095-38351153	+	CGTCCATGGCCGCAGGGGTAG	Yes	No	No	0	0	0	No	
NM_hsa_57	chr11:131565808-131565867	+	ATCCTGTCCCAAGAGTGACAGC	Yes	Yes	Yes	0	0	0	No	

Additional file 5

Name	locus	strand	sequence	mirdeep2	mireap	miranalyzer	RPM_HU_KapB_cMyc	RPM_HU_KaposinB	RPM_HUVEC	IP_confidence	annotation
NM_hsa_57	chr11:131565798-131565873	+	ATCCTGTCCCAAGAGTGACAGC	Yes	Yes	Yes	0	0	0	No	
NM_hsa_57	chr11:131565768-131565906	+	ATCCTGTCCCAAGAGTGACAGC	Yes	Yes	Yes	0	0	0	No	
NM_hsa_570	chr7:38351095-38351153	+	GCCCCTTCCGGCCAGCC	Yes	No	No	0	0	0	No	
NM_hsa_572	chr6:33175630-33175691	-	GACGTCCAGACTCAACTCTCG	Yes	No	No	0.0569857	0	0	Yes	
NM_hsa_573	chr17:1633600-1633681	+	TGGGGTGGGAGGGGTGATGAGC	Yes	Yes	Yes	0	0	0	No	
NM_hsa_573	chr17:1633610-1633673	+	TGGGGTGGGAGGGGTGATGAGC	Yes	Yes	Yes	0	0	0	No	
NM_hsa_573	chr17:1633570-1633708	+	TGGGGTGGGAGGGGTGATGAGC	Yes	Yes	Yes	0	0	0	No	
NM_hsa_574	chr17:1633610-1633673	+	TCAGGGCCTGCTCCCACCCCGCA	Yes	No	No	0	0	0.0732862	Yes	
NM_hsa_575	chr2:234589698-234589793	-	CGGCTCACCTGTGTCTCTGCAA	Yes	Yes	Yes	0	0	0	No	
NM_hsa_575	chr2:234589718-234589783	-	CGGCTCACCTGTGTCTCTGCAA	Yes	Yes	Yes	0	0	0	No	
NM_hsa_575	chr2:234589705-234589803	-	CGGCTCACCTGTGTCTCTGCAA	Yes	Yes	Yes	0	0	0	No	
NM_hsa_576	chr2:234589718-234589783	-	GCAGAGACACAGGTGAGCCACA	Yes	No	No	0	0	0	No	
NM_hsa_579	chr8:70560149-70560265	+	CTTTTGGATACTAGCAGGACG	Yes	Yes	Yes	0	0	0	No	
NM_hsa_579	chr8:70560181-70560236	+	CTTTTGGATACTAGCAGGACG	Yes	Yes	Yes	0	0	0	No	
NM_hsa_579	chr8:70560168-70560245	+	CTTTTGGATACTAGCAGGACG	Yes	Yes	Yes	0	0	0	No	
NM_hsa_58	chr11:131565808-131565867	+	GCCGCTCAAAGGACAGGGCTGG	Yes	No	No	0	0	0	No	
NM_hsa_580	chr8:70560181-70560236	+	TCCTGCTAGTATCAAAAAGCC	Yes	No	No	0	0	0	No	
NM_hsa_581	chr14:21493471-21493545	+	GGATCCCTCGGCTGCCCTCAGC	Yes	Yes	No	0	0	0	No	
NM_hsa_581	chr14:21493480-21493536	+	GGATCCCTCGGCTGCCCTCAGC	Yes	Yes	No	0	0	0	No	
NM_hsa_582	chr14:21493480-21493536	+	TGTGGGGAGTGCGGGGATCCCT	Yes	No	No	0	0	0	No	
NM_hsa_585	chr22:41621146-41621227	+	CTGCACCCAGAGTCCCTGACTTT	Yes	Yes	No	0	0	0	Yes	
NM_hsa_585	chr22:41621159-41621218	+	CTGCACCCAGAGTCCCTGACTTT	Yes	Yes	No	0	0	0	Yes	
NM_hsa_586	chr22:41621159-41621218	+	CGTCAGGGGGTCTGTGTCCCAGCC	Yes	No	No	0	0	0	No	
NM_hsa_587	chr1:162531046-162531121	-	GTCGGTTGGGTCTGCTAG	Yes	No	No	0	0	0	No	
NM_hsa_587	chr1:162531103-162531146	-	GTCGGTTGGGTCTGCTAG	Yes	No	No	0	0	0	No	
NM_hsa_588	chr1:162531103-162531146	-	TGCGCACTAACTGAGGG	Yes	No	No	0	0	0	No	
NM_hsa_589	chr20:44523532-44523672	+	GACCCACTGTCTGTGCCTTCCAG	Yes	Yes	Yes	0.0569857	0	0	No	
NM_hsa_589	chr20:44523548-44523642	+	GACCCACTGTCTGTGCCTTCCAG	Yes	Yes	Yes	0.0569857	0	0	No	
NM_hsa_589	chr20:44523567-44523633	+	GACCCACTGTCTGTGCCTTCCAG	Yes	Yes	Yes	0.0569857	0	0	No	
NM_hsa_59	chr6:41701281-41701358	-	CCCGGAGAGCTGGGAGCCAGA	Yes	Yes	Yes	0	0	0	No	
NM_hsa_59	chr6:41701251-41701387	-	CCCGGAGAGCTGGGAGCCAGA	Yes	Yes	Yes	0	0	0	No	
NM_hsa_59	chr6:41701290-41701348	-	CCCGGAGAGCTGGGAGCCAGA	Yes	Yes	Yes	0	0	0	No	
NM_hsa_590	chr20:44523567-44523633	+	GGTGAGGTGGGGGCAGGGGGAGG	Yes	No	No	0	0	0	No	
NM_hsa_591	chr3:133785734-133785795	+	TCTGCTGAGAGTTTCTGACTGA	Yes	No	No	1.82354	2.0529	0.806149	Yes	
NM_hsa_592	chr3:133785734-133785795	+	GGTCAAAAACTCTCAGTGGACT	Yes	No	No	0	0.132445	0	No	
NM_hsa_593	chr15:57608855-57608912	+	TCATCCCCAGAAAGGACTTGGT	Yes	Yes	Yes	0	0	0	No	
NM_hsa_593	chr15:57608845-57608920	+	TCATCCCCAGAAAGGACTTGGT	Yes	Yes	Yes	0	0	0	No	
NM_hsa_593	chr15:57608815-57608953	+	TCATCCCCAGAAAGGACTTGGT	Yes	Yes	Yes	0	0	0	No	
NM_hsa_594	chr15:57608855-57608912	+	CAAGTCATTTCTGGGGGTGCCC	Yes	No	No	0	0	0	No	
NM_hsa_597	chr6:25279591-25279649	+	AGGGCTGTAGGTGCGGCG	Yes	No	No	0	0	0	No	
NM_hsa_598	chr6:25279591-25279649	+	CCGCTCTCCCCGCCCTCT	Yes	No	No	0	0	0	No	
NM_hsa_599	chr5:156736971-156737087	+	CAAAAGGGCTCAGAGCACAGC	Yes	Yes	Yes	0.0569857	0	0	Yes	
NM_hsa_599	chr5:156736979-156737067	+	CAAAAGGGCTCAGAGCACAGC	Yes	Yes	Yes	0.0569857	0	0	Yes	
NM_hsa_599	chr5:156736995-156737058	+	CAAAAGGGCTCAGAGCACAGC	Yes	Yes	Yes	0.0569857	0	0	Yes	
NM_hsa_60	chr6:41701250-41701388	-	TGGCTCCCTTCTCTCCGTCTGC	Yes	Yes	Yes	0	0	0	No	
NM_hsa_60	chr6:41701281-41701358	-	TGGCTCCCTTCTCTCCGTCTGC	Yes	Yes	Yes	0	0	0	No	
NM_hsa_60	chr6:41701290-41701348	-	TGGCTCCCTTCTCTCCGTCTGC	Yes	Yes	Yes	0	0	0	No	
NM_hsa_600	chr5:156736995-156737058	+	GGTGCTCTGAGCCCTTTTGTA	Yes	No	No	0	0	0	No	
NM_hsa_601	chr20:9491465-9491536	-	TCTTGTGGGACAGAAACTGA	Yes	Yes	No	0	0	0	No	
NM_hsa_601	chr20:9491457-9491546	-	TCTTGTGGGACAGAAACTGA	Yes	Yes	No	0	0	0	No	
NM_hsa_602	chr20:9491465-9491536	-	AGTTTCTATCCCACAAGGGT	Yes	No	No	0	0	0	No	
NM_hsa_603	chr10:3827731-3827776	+	CTGTGAGGTGCGCGGCCGAG	Yes	No	No	0	0	0	No	
NM_hsa_604	chr10:3827731-3827776	+	CGGCCGCGCGCCGGAGGAGTC	Yes	No	No	0	0	0	No	
NM_hsa_605	chr2:182013372-182013434	+	TCTGTGGATCTGGTATCTGTGG	Yes	Yes	Yes	0	0	0	No	
NM_hsa_605	chr2:182013347-182013455	+	TCTGTGGATCTGGTATCTGTGG	Yes	Yes	Yes	0	0	0	No	
NM_hsa_605	chr2:182013362-182013452	+	TCTGTGGATCTGGTATCTGTGG	Yes	Yes	Yes	0	0	0	No	
NM_hsa_606	chr2:182013372-182013434	+	ACAGATAGCAGATCCACAGATA	Yes	No	No	0	0	0	No	
NM_hsa_608	chr14:45553280-45553345	+	TGACTTTTTCGCCTCCAGGG	Yes	No	No	0	0	0	No	
NM_hsa_609	chr1:44451113-44451190	+	TCTGTCCGTCCCCATCCTCAGG	Yes	No	No	0	0	0	Yes	
NM_hsa_61	chr3:49824155-49824289	+	GCTACTACGAGGCCGGAGCG	Yes	Yes	Yes	0	0	0	No	
NM_hsa_61	chr3:49824193-49824250	+	GCTACTACGAGGCCGGAGCG	Yes	Yes	Yes	0	0	0	No	
NM_hsa_61	chr3:49824183-49824259	+	GCTACTACGAGGCCGGAGCG	Yes	Yes	Yes	0	0	0	No	
NM_hsa_610	chr1:44451113-44451190	+	GGAGGGGGGGTGCAGACT	Yes	No	No	0	0	0	No	
NM_hsa_611	chr10:89307582-89307655	+	AGATTGATTGATGGCTCTGTGG	Yes	Yes	Yes	0	0	0	No	
NM_hsa_611	chr10:89307572-89307670	+	AGATTGATTGATGGCTCTGTGG	Yes	Yes	Yes	0	0	0	No	
NM_hsa_611	chr10:89307592-89307647	+	AGATTGATTGATGGCTCTGTGG	Yes	Yes	Yes	0	0	0	No	
NM_hsa_612	chr10:89307592-89307647	+	ATAGAGCCATCGATCAATATGT	Yes	No	No	0	0	0.0732862	Yes	
NM_hsa_613	chr1:45349964-45350026	-	CCCATACTTTCACCCCTCTC	Yes	Yes	No	0	0	0	Yes	
NM_hsa_613	chr1:45349955-45350039	-	CCCATACTTTCACCCCTCTC	Yes	Yes	No	0	0	0	Yes	
NM_hsa_614	chr1:45349964-45350026	-	GAGGTGGTGGGAAGTATGCA	Yes	No	No	0	0	0	Yes	
NM_hsa_615	chr5:9053926-9054009	+	TAAAGGCTTTGAACACGAGTTC	Yes	Yes	Yes	0	0	0	No	
NM_hsa_615	chr5:9053938-9054000	+	TAAAGGCTTTGAACACGAGTTC	Yes	Yes	Yes	0	0	0	No	
NM_hsa_615	chr5:9053906-9054034	+	TAAAGGCTTTGAACACGAGTTC	Yes	Yes	Yes	0	0	0	No	
NM_hsa_616	chr5:9053938-9054000	+	ACTCGTGCTTGAAGCCTTTAGC	Yes	No	No	0	0	0	No	
NM_hsa_617	chr3:52427956-52428040	+	CCTTCTCGAGCCTTGAGTGTGC	Yes	Yes	No	0	0	0	Yes	
NM_hsa_617	chr3:52427970-52428031	+	CCTTCTCGAGCCTTGAGTGTGC	Yes	Yes	No	0	0	0	Yes	
NM_hsa_618	chr3:52427970-52428031	+	TGGCTCAAGGTTCAAGAAGGCC	Yes	No	No	0	0	0	No	
NM_hsa_619	chr6:31856313-31856375	-	GTGGGGCCGTGGGCTGGTGG	Yes	Yes	No	0	0	0	No	
NM_hsa_619	chr6:31856311-31856385	-	GTGGGGCCGTGGGCTGGTGG	Yes	Yes	No	0	0	0	No	
NM_hsa_62	chr3:49824193-49824250	+	TTCGTTCCCGGAGTGGCTGG	Yes	Yes	Yes	0	0	0	Yes	
NM_hsa_62	chr3:49824183-49824259	+	TTCGTTCCCGGAGTGGCTGG	Yes	Yes	Yes	0	0	0	Yes	
NM_hsa_62	chr3:49824153-49824287	+	TTCGTTCCCGGAGTGGCTGG	Yes	Yes	Yes	0	0	0	Yes	
NM_hsa_620	chr6:31856313-31856375	-	CCCGGCCCCAGCCTCACGC	Yes	No	No	0	0	0	No	
NM_hsa_621	chr1:2266897-2266960	+	GGGTCTGGTTCTCCACCC	Yes	No	No	0	0	0	No	
NM_hsa_622	chr3:126751433-126751525	+	GTAGGTGGAGGGTGCAGGGGT	Yes	Yes	No	0	0	0	No	
NM_hsa_622	chr3:126751443-126751518	+	GTAGGTGGAGGGTGCAGGGGT	Yes	Yes	No	0	0	0	No	
NM_hsa_623	chr3:126751443-126751518	+	TCATGTGCCCACCTGGCCA	Yes	No	No	0	0	0	No	
NM_hsa_624	chr3:153106182-153106258	+	ATCAATGTCATTGTCACTGAGA	Yes	Yes	Yes	0	0	0	No	
NM_hsa_624	chr3:153106175-153106263	+	ATCAATGTCATTGTCACTGAGA	Yes	Yes	Yes	0	0	0	No	
NM_hsa_624	chr3:153106194-153106249	+	ATCAATGTCATTGTCACTGAGA	Yes	Yes	Yes	0	0	0	No	
NM_hsa_625	chr3:153106194-153106249	+	TCAGTGACAATGACATTGACTA	Yes	No	No	0	0	0	No	
NM_hsa_626	chr22:37942071-37942110	-	TGGCTTGACCAGGCTCTGG	Yes	No	No	0	0	0	No	
NM_hsa_627	chr22:37942071-37942110	-	GGGGCCGGAGGCTTCA	Yes	No	No	0	0	0	No	
NM_hsa_628	chr4:1737218-1737289	+	AGCCCAGGATGAAACTCTGACA	Yes	Yes	No	4.72982	17.0854	37.9623	No	H>KapB>KapB+cMyc
NM_hsa_628	chr4:1737207-1737298	+	AGCCCAGGATGAAACTCTGACA	Yes	Yes	No	4.72982	17.0854	37.9623	No	H>KapB>KapB+cMyc
NM_hsa_629	chr4:1737218-1737289	+	TCTAAGTGGTGTCCTGTGGCAGG	Yes	No	No	0	0	0	Yes	
NM_hsa_63	chr15:101460156-101460250	+	TTCCTGGGGTTGCGGGGCGGCC	Yes	Yes	No	0	0.132445	0	No	
NM_hsa_63	chr15:101460166-101460241	+	TTCCTGGGGTTGCGGGGCGGCC	Yes	Yes	No	0	0.132445	0	No	
NM_hsa_630	chr11:65190840-65190882	+	TTTTCGCTCGGCCTGGGAC	Yes	No	No	0.0569857	0.0662225	0.0732862	No	H>KapB>KapB+cMyc
NM_hsa_631	chr11:65190840-65190882	+	CTCTGGCCCAGGGTGGTATGT	Yes	No	No	0	0	0	No	
NM_hsa_632	chr17:73402150-73402240	-	TCCGGTCCGTACAAACTCTGCA	Yes	No	No	0.227943	0.0662225	0.366431	Yes	
NM_hsa_634	chr16:57693229-57693308	+	TTCCCACACTGGCCCACCAGG	Yes	No	No	0	0	0	No	
NM_hsa_635	chr16:57693229-57693308	+	TGGGGGGCAGTCTGGGAAGG	Yes	No	No	0	0	0	No	
NM_hsa_636	chr2:101618547-101618615	-	GCAGGCGTCGTGGCGGGG	Yes	No	No	0	0	0	No	
NM_hsa_636	chr2:101618597-101618657	-	GCAGGCGTCGTGGCGGGG	Yes	No	No	0	0	0	No	
NM_hsa_637	chr2:101618547-101618615	-	CCGCTTCTCGACGGATTCCTT	Yes	No	No	0	0	0	No	
NM_hsa_64	chr15:101460156-101460250	+	TCACGCTTCTTGCCCAGGTGGT	Yes	Yes	No	0	0	0	No	
NM_hsa_64	chr15:101460166-101460241	+	TCACGCTTCTTGCCCAGGTGGT	Yes	Yes	No	0	0	0	No	
NM_hsa_641	chr17:48270171-48270242	-	CCCTGAGTTCCCCTTTGC	Yes	No	No	0	0	0	No	
NM_hsa_642	chr9:132250928-132250997	-	GCCGCAGGCCAGCGTCGGG	Yes	No	No	0	0	0	No	
NM_hsa_643	chr9:132250928-132250997	-	CGAGACGGGCCGGGAAGCGGAAA	Yes	No	No	0	0	0	No	
NM_hsa_644	chr12:117257315-117257372	-	ACCGCGTCGGGCGGCCGG	Yes	No	No	0	0	0	No	
NM_hsa_645	chr12:117257315-117257372	-	GGCCGCCTGCCTCCTC	Yes	No	No	0	0	0	No	
NM_hsa_646	chr4:139228328-139228385	-	CCTAGCAGTCTCAGGACACAC	Yes	Yes	Yes	0	0	0	Yes	
NM_hsa_646	chr4:139228299-139228415	-	CCTAGCAGTCTCAGGACACAC	Yes	Yes	Yes	0	0	0	Yes	
NM_hsa_646	chr4:139228319-139228398	-	CCTAGCAGTCTCAGGACACAC	Yes	Yes	Yes	0	0	0	Yes	
NM_hsa_647	chr4:139228328-139228385	-	GTGCCCTGAGACTGCTAAGTA	Yes	No	No	0	0	0	No	
NM_hsa_648	chr7:129793405-129793465	+	TTCCTCCCTCTCCCTGCCTGGT	Yes	Yes	Yes	0	0	0	No	
NM_hsa_648	chr7:129793390-129793474	+	TTCCTCCCTCTCCCTGCCTGGT	Yes	Yes	Yes	0	0	0	No	
NM_hsa_648	chr7:129793371-129793499	+	TTCCTCCCTCTCCCTGCCTGGT	Yes	Yes	Yes	0	0	0	No	
NM_hsa_649	chr7:129793405-129793465	+	CGGTAGGAGGAAGGAGGAAGG	Yes	No	No	0	0	0	No	
NM_hsa_65	chr22:29665129-29665192	+	CTGGTCTCTCTGACCTAGGAGG	Yes	Yes	No	0	0	0	No	
NM_hsa_65	chr22:29665119-29665201	+	CTGGTCTCTCTGACCTAGGAGG	Yes	Yes	No	0	0	0	No	
NM_hsa_650	chr19:52185341-52185423	+	TTCTCAGGATTCACACTTGTAG	Yes	Yes	Yes	0	0	0	No	
NM_hsa_650	chr19:52185353-52185414	+	TTCTCAGGATTCACACTTGTAG	Yes	Yes	Yes	0	0	0	No	
NM_hsa_650	chr19:52185340-52185428	+	TTCTCAGGATTCACACTTGTAG	Yes	Yes	Yes	0	0	0	No	
NM_hsa_650	chr19:52185392-52185457	+	TTCTCAGGATTCACACTTGTAG	Yes	Yes	Yes	0	0	0	No	
NM_hsa_651	chr19:52185353-52185414	+	ACGAGTGTGAATCCTGAGAGCA	Yes	No	No	0	0	0	No	
NM_hsa_654	chr2:677749-677795	+	ACTCCTCGGACCCTGCAGGAC	Yes	No	No	0	0	0	No	
NM_hsa_655	chr2:677749-677795	+	CCTGCAGGAGCCGGGGACATT	Yes	No	No	0	0	0	No	
NM_hsa_656	chr19:48981470-48981533	+	TCTGTCCACCTTGCTTCTTCAGG	Yes	No	No	0.512871	0	0	Yes	
NM_hsa_657	chr19:48981470-48981533	+	TGAGGGAGGTGGGGTGGGGTGAGGAC	Yes	No	No	0	0	0	No	
NM_hsa_658	chr20:3451505-3451576	-	CTGACGTGGACCGTAGAA	Yes	No	No	0	0	0	No	
NM_hsa_659	chr20:3451505-3451576	-	GTGCGGGGGCGCAGAC	Yes	No	No	0	0	0	No	
NM_hsa_66	chr22:29665129-29665192	+	GCCTGGGTCGAGAGACGAGA	Yes	Yes	No	0	0	0.0732862	No	
NM_hsa_66	chr22:29665119-29665201	+	GCCTGGGTCGAGAGACGAGA	Yes	Yes	No	0	0	0.0732862	No	
NM_hsa_662	chr19:1876127-1876171	-	CTGGCGTCAGGCTGTGGA	Yes	No	No	0	0	0	No	
NM_hsa_663	chr19:1876127-1876171	-	GGCAGTGGAGACGGCGGCG	Yes	No	No	0	0	0	No	
NM_hsa_664	chr19:4058540-4058595	+	TCCTCCAGGCCGGCCGGTGG	Yes	Yes	No	0	0	0	No	
NM_hsa_664	chr19:4058565-4058646	+	TCCTCCAGGCCGGCCGGTGG	Yes	Yes	No	0	0	0	No	
NM_hsa_664	chr19:4058575-4058636	+	TCCTCCAGGCCGGCCGGTGG	Yes	Yes	No	0	0	0	No	
NM_hsa_665	chr19:4058575-4058636	+	CAAGGCCCCTGGGGAGAGG	Yes	No	No	0	0	0	No	
NM_hsa_666	chr12:116822700-116822760	+	TTGCGTGAACCTGAGAATGAGC	Yes	Yes	Yes	0	0	0	No	
NM_hsa_666	chr12:116822661-116822799	+	TTGCGTGAACCTGAGAATGAGC	Yes	Yes	Yes	0	0	0	No	
NM_hsa_666	chr12:116822687-116822769	+	TTGCGTGAACCTGAGAATGAGC	Yes	Yes	Yes	0	0	0	No	
NM_hsa_667	chr12:116822700-116822760	+	TTGTTCTCAGGGCCATGCATGA	Yes	No	No	0	0	0	No	
NM_hsa_668	chr19:7705722-7705787	+	CCCACTCCTGCCTCACCCCAGC	Yes	No	No	0	0	0	Yes	
NM_hsa_669	chr19:7705722-7705787	+	GTGGGTGGGGCAAGGAGGTGTGGGGGC	Yes	No	No	0	0	0	No	
NM_hsa_67	chr15:49622618-49622694	+	TCAGATATTCTGGATTACTCAG	Yes	Yes	Yes	0	0	0	No	
NM_hsa_67	chr15:49622598-49622716	+	TCAGATATTCTGGATTACTCAG	Yes	Yes	Yes	0	0	0	No	
NM_hsa_67	chr15:49622628-49622685	+	TCAGATATTCTGGATTACTCAG	Yes	Yes	Yes	0	0	0	No	
NM_hsa_670	chr19:54940996-54941064	+	TGCTGTGGGAGTGTGGAATCGG	Yes	Yes	No	0	0	0	No	
NM_hsa_670	chr19:54940986-54941074	+	TGCTGTGGGAGTGTGGAATCGG	Yes	Yes	No	0	0	0	No	
NM_hsa_671	chr19:54940996-54941064	+	TTTTCTGCTGCCTCACGCAGGGCC	Yes	No	No	0	0	0	No	
NM_hsa_672	chr17:15466647-15466729	-	CGGCTCCTGGGACTGGCTCTG	Yes	Yes	No	0	0	0	No	
NM_hsa_674	chr7:150778042-150778122	+	CGCAGTCCGTGCCCTTCTCT	Yes	Yes	No	0	0	0	Yes	
NM_hsa_674	chr7:150778063-150778113	+	CGCAGTCCGTGCCCTTCTCT	Yes	Yes	No	0	0	0	Yes	
NM_hsa_675	chr7:150778063-150778113	+	AGAAGGGAAGGCGGAGGGCC	Yes	No	No	0	0	0	No	

Additional file 5

Name	locus	strand	sequence	mirdeep2	mireap	miranalyzer	RPM_HU_KapB_cMyc	RPM_HU_KaposinB	RPM_HUVEC	IP_confidence	annotation
NM_hsa_676	chr19:12900667-12900748	-	CTGCCAGGTCCCAGGGCGCG	Yes	Yes	Yes	0	0	0	No	
NM_hsa_676	chr19:12900657-12900751	-	CTGCCAGGTCCCAGGGCGCG	Yes	Yes	Yes	0	0	0	No	
NM_hsa_676	chr19:12900676-12900737	-	CTGCCAGGTCCCAGGGCGCG	Yes	Yes	Yes	0	0	0	No	
NM_hsa_677	chr19:12900676-12900737	-	CTGTCCTGGGGCCCCGAGCCAGAA	Yes	No	No	0	0	0	No	
NM_hsa_68	chr15:49622618-49622694	+	AGTGGTGGAATATCTGATACT	Yes	Yes	Yes	0	0	0	No	
NM_hsa_68	chr15:49622598-49622714	+	AGTGGTGGAATATCTGATACT	Yes	Yes	Yes	0	0	0	No	
NM_hsa_68	chr15:49622628-49622685	+	AGTGGTGGAATATCTGATACT	Yes	Yes	Yes	0	0	0	No	
NM_hsa_680	chr17:2497441-2497524	+	CCCGGAGGAGGGGGACTGT	Yes	No	No	0	0	0	No	
NM_hsa_681	chr17:2497441-2497524	+	CCTCCTCCTTCTTCTTCTC	Yes	No	No	0	0	0	No	
NM_hsa_682	chr2:112662460-112662524	+	TCTGGCACTCTGGGCATTGGCT	Yes	Yes	Yes	0	0	0	No	
NM_hsa_682	chr2:112662425-112662563	+	TCTGGCACTCTGGGCATTGGCT	Yes	Yes	Yes	0	0	0	No	
NM_hsa_682	chr2:112662448-112662533	+	TCTGGCACTCTGGGCATTGGCT	Yes	Yes	Yes	0	0	0	No	
NM_hsa_683	chr2:112662460-112662524	+	CCAACCCTCAGGTGCCCGGGAGG	Yes	No	No	0	0	0	No	
NM_hsa_686	chr8:138335349-138335424	+	TGTGTCAGTGGGACTGAAATGA	Yes	Yes	No	0	0	0	No	
NM_hsa_686	chr8:138335359-138335416	+	TGTGTCAGTGGGACTGAAATGA	Yes	Yes	No	0	0	0	No	
NM_hsa_687	chr8:138335359-138335416	+	ATTTCAGTTCCCACTGACACAGC	Yes	No	No	0	0	0	No	
NM_hsa_688	chr19:1003059-1003146	+	CACCCCTCTCCCAGAACCCATT	Yes	Yes	No	0.113971	0	0	Yes	
NM_hsa_688	chr19:1003074-1003137	+	CACCCCTCTCCCAGAACCCATT	Yes	Yes	No	0.113971	0	0	Yes	
NM_hsa_689	chr19:1003074-1003137	+	AGGGTTTTGTGGGGGTGGA	Yes	No	No	0	0	0	No	
NM_hsa_69	chr9:129202398-129202492	+	CCTCCTGTAACGGGCTCTGG	Yes	No	Yes	0	0	0	No	
NM_hsa_69	chr9:129202413-129202473	+	CCTCCTGTAACGGGCTCTGG	Yes	No	Yes	0	0	0	No	
NM_hsa_690	chr1:110302413-110302499	+	GACTCTCTGACTCCCCTGCTTGC	Yes	Yes	Yes	0	0	0	No	
NM_hsa_690	chr1:110302423-110302481	+	GACTCTCTGACTCCCCTGCTTGC	Yes	Yes	Yes	0	0	0	No	
NM_hsa_690	chr1:110302393-110302513	+	GACTCTCTGACTCCCCTGCTTGC	Yes	Yes	Yes	0	0	0	No	
NM_hsa_691	chr1:110302423-110302481	+	CTGCAGGGAGAGGGGGAGTCCT	Yes	No	No	0	0	0	No	
NM_hsa_693	chr10:113943427-113943506	-	CTGCCAGGGCAGGTGT	Yes	No	No	0	0	0	No	
NM_hsa_694	chr1:1560296-1560356	+	AGGGGCGGGGTCAGGGCTGGG	Yes	No	No	0	0	0	No	
NM_hsa_695	chr1:1560296-1560356	+	CAGCCTTAGCCTGCTGGGGG	Yes	No	No	0	0	0	No	
NM_hsa_696	chr1:237736815-237736874	-	GGAAGAGGATAGGGTTGTTCCT	Yes	No	No	0	0	0	No	
NM_hsa_697	chr7:73770655-73770761	+	CTTGGGTCTCTCCTGCTTCCA	Yes	Yes	Yes	0	0	0	No	
NM_hsa_697	chr7:73770674-73770737	+	CTTGGGTCTCTCCTGCTTCCA	Yes	Yes	Yes	0	0	0	No	
NM_hsa_697	chr7:73770661-73770746	+	CTTGGGTCTCTCCTGCTTCCA	Yes	Yes	Yes	0	0	0	No	
NM_hsa_698	chr7:73770674-73770737	+	GAGGCAGGGGAGGCTCTCCAGGCT	Yes	No	No	0	0	0	Yes	
NM_hsa_7	chr14:94579955-94580073	+	TTTTGTGTGTCAGGGTGCAGGT	Yes	Yes	Yes	1.25369	1.58934	2.1253	Yes	H>KapB>KapB+cMyc
NM_hsa_7	chr14:94579986-94580044	+	TTTTGTGTGTCAGGGTGCAGGT	Yes	Yes	Yes	1.25369	1.58934	2.1253	Yes	H>KapB>KapB+cMyc
NM_hsa_7	chr14:94579976-94580053	+	TTTTGTGTGTCAGGGTGCAGGT	Yes	Yes	Yes	1.25369	1.58934	2.1253	Yes	H>KapB>KapB+cMyc
NM_hsa_70	chr9:129202393-129202491	+	GCGAGCCCCTTGCTGTATGGAG	Yes	No	Yes	0	0	0	No	
NM_hsa_70	chr9:129202413-129202473	+	GCGAGCCCCTTGCTGTATGGAG	Yes	No	Yes	0	0	0	No	
NM_hsa_701	chr15:68992906-68992955	+	CCGAGGGAGGGCTGGGGGCC	Yes	No	No	0	0	0	No	
NM_hsa_702	chr15:68992906-68992955	+	ACCCAGGCCCTTCCCCAGGGG	Yes	No	No	0	0	0	No	
NM_hsa_703	chr8:143557199-143557254	+	CTCTGAGAAGCAGGACCTAA	Yes	No	No	0	0	0	No	
NM_hsa_704	chr8:143557199-143557254	+	GGGCCTGCAGGAAGAGTG	Yes	No	No	0	0	0	No	
NM_hsa_705	chr19:14192511-14192567	+	CGAGGCCGGGCTGCACTGCGA	Yes	No	No	0.484379	0.629114	0.622933	Yes	
NM_hsa_706	chr19:14192511-14192567	+	GCAGACAGGTCAGCTCGGCCTGGCC	Yes	No	No	0	0	0	No	
NM_hsa_707	chr9:139887231-139887339	+	TCTGCCCGCCCTGTAGCTGTAG	Yes	Yes	Yes	0	0	0	Yes	
NM_hsa_707	chr9:139887256-139887315	+	TCTGCCCGCCCTGTAGCTGTAG	Yes	Yes	Yes	0	0	0	Yes	
NM_hsa_707	chr9:139887246-139887340	+	TCTGCCCGCCCTGTAGCTGTAG	Yes	Yes	Yes	0	0	0	Yes	
NM_hsa_708	chr9:139887256-139887315	+	AGGGCTCTGGGGTCGGGCAGGTT	Yes	No	No	0	0	0	No	
NM_hsa_709	chr5:167592864-167592923	+	CTCTCACCTGGCATAAGCAATT	Yes	Yes	No	0	0	0	No	
NM_hsa_709	chr5:167592851-167592932	+	CTCTCACCTGGCATAAGCAATT	Yes	Yes	No	0	0	0	No	
NM_hsa_71	chr3:57678917-57678981	+	CTCTTTGGGACTGAAATGG	Yes	No	No	0	0	0	No	
NM_hsa_710	chr5:167592864-167592923	+	TTGCTTATGTCAGGTGAGAGGG	Yes	No	No	0	0	0	No	
NM_hsa_711	chr14:93581816-93581868	+	CTGAGTCTGAGTCGGAGCTGG	Yes	No	No	0	0	0	No	
NM_hsa_712	chr14:93581816-93581868	+	CCTGCCGGCTGAGGCTGGGGC	Yes	No	No	0	0	0	No	
NM_hsa_713	chr5:315514-315594	+	TGCGTCTGGTTTTGGGGCTGCAG	Yes	Yes	Yes	0	0	0	Yes	
NM_hsa_713	chr5:315494-315614	+	TGCGTCTGGTTTTGGGGCTGCAG	Yes	Yes	Yes	0	0	0	Yes	
NM_hsa_713	chr5:315525-315585	+	TGCGTCTGGTTTTGGGGCTGCAG	Yes	Yes	Yes	0	0	0	Yes	
NM_hsa_714	chr5:315525-315585	+	CCAGCCTCATTCTCCAGACGCTTC	Yes	No	No	0	0	0	Yes	
NM_hsa_715	chr8:49427253-49427297	+	CAGGCCGCCGAGTCCACCC	Yes	No	No	0	0	0	No	
NM_hsa_718	chr11:67069157-67069203	+	CTGTGGGCTCTGGTGGCTGCAG	Yes	No	No	0	0	0	Yes	
NM_hsa_719	chr11:67069157-67069203	+	GCAGCCACACGGCAGAAGTG	Yes	No	No	0	0	0	No	
NM_hsa_72	chr3:57678917-57678981	+	CTTGTCTCGGATTGTGAAGC	Yes	No	No	0	0	0	No	
NM_hsa_720	chr1:8086566-8086635	+	GCGTCTCCGGGCTGACCG	Yes	No	No	0	0	0	No	
NM_hsa_722	chrX:48673316-48673370	+	CTGACCCTCCATGTCCCCCCAGG	Yes	No	No	0	0	0	No	
NM_hsa_723	chrX:48673316-48673370	+	TGGGGCGGCAGGTGGGTTGCA	Yes	No	No	0	0	0	No	
NM_hsa_726	chr12:98044116-98044189	+	ATAACAGAAAGCTAGACTGGA	Yes	Yes	Yes	0	0	0	No	
NM_hsa_726	chr12:98044106-98044202	+	ATAACAGAAAGCTAGACTGGA	Yes	Yes	Yes	0	0	0	No	
NM_hsa_726	chr12:98044126-98044181	+	ATAACAGAAAGCTAGACTGGA	Yes	Yes	Yes	0	0	0	No	
NM_hsa_727	chr12:98044126-98044181	+	CAGTTTGGCTTCCTGTTATCT	Yes	No	No	0	0	0	No	
NM_hsa_73	chr15:66927503-66927586	+	AAGGGCTGAACCTAGAATTCT	Yes	Yes	No	0	0	0	No	
NM_hsa_73	chr15:66927513-66927578	+	AAGGGCTGAACCTAGAATTCT	Yes	Yes	No	0	0	0	No	
NM_hsa_730	chr10:104229664-104229760	+	CTGTCTCCCCTCTGCCTGCAGA	Yes	Yes	No	0	0	0	Yes	
NM_hsa_730	chr10:104229675-104229751	+	CTGTCTCCCCTCTGCCTGCAGA	Yes	Yes	No	0	0	0	Yes	
NM_hsa_731	chr10:104229675-104229751	+	AGCAGGCTGAGGGGGGAGGA	Yes	No	No	0	0	0	Yes	
NM_hsa_732	chr1:78149132-78149170	+	CTCGTTGGGACTGGCGGG	Yes	No	No	0	0	0	No	
NM_hsa_733	chr1:78149132-78149170	+	TTCCGGTCCCCTCCTTTC	Yes	No	No	0	0	0	No	
NM_hsa_734	chr22:37172546-37172602	+	AGGCCGCCGCTGTAGAGGT	Yes	No	No	0	0	0	No	
NM_hsa_735	chr22:37172546-37172602	+	CTCTAGGCGGTCTGG	Yes	No	No	0	0	0	No	
NM_hsa_736	chr19:7506733-7506799	+	CCACACCTCGCTCTTCCCTCAGA	Yes	No	No	0	0	0	Yes	
NM_hsa_737	chr19:7506733-7506799	+	TACGGGAGGGAATTGGGGTGGCATGGCT	Yes	No	No	0	0	0	No	
NM_hsa_738	chr16:9375882-9375939	+	CTTCTGCCCCTTCTCCAGCGC	Yes	Yes	No	0	0	0	No	
NM_hsa_738	chr16:9375872-9375947	+	CTTCTGCCCCTTCTCCAGCGC	Yes	Yes	No	0	0	0	No	
NM_hsa_739	chr16:9375882-9375939	+	CCTCGGAGAAGGGTCAGGGCT	Yes	No	No	0	0	0.219859	No	
NM_hsa_74	chr15:66927513-66927578	+	AATTCTAGGTTCAGCCCTCGG	Yes	No	No	0	0	0	No	
NM_hsa_740	chr14:105293075-105293136	+	TCCGTCCCGGGGCTGATGAG	Yes	No	No	0	0	0	No	
NM_hsa_741	chr14:105293075-105293136	+	AATGCAACCCTTGGACACGGT	Yes	No	No	0	0	0	No	
NM_hsa_742	chr2:161053361-161053428	-	TCACTAGATTTGGGGATTGACA	Yes	Yes	Yes	0	0	0	No	
NM_hsa_742	chr2:161053330-161053468	-	TCACTAGATTTGGGGATTGACA	Yes	Yes	Yes	0	0	0	No	
NM_hsa_742	chr2:161053352-161053438	-	TCACTAGATTTGGGGATTGACA	Yes	Yes	Yes	0	0	0	No	
NM_hsa_743	chr2:161053361-161053428	-	TCAATTCCCAAATCTAGTGATA	Yes	No	No	0	0	0	No	
NM_hsa_746	chr13:36104474-36104537	+	GGCGGCCTGTCCCCAGTGC	Yes	No	No	0.370407	0.198668	0.256502	Yes	
NM_hsa_747	chr13:36104474-36104537	+	GATGGGGACAGCCGCCCC	Yes	No	No	0	0	0	No	
NM_hsa_75	chr22:23736584-23736637	-	TCAGACAGGGCTCCCCGCACCG	Yes	Yes	Yes	0	0	0.0732862	No	
NM_hsa_75	chr22:23736575-23736648	-	TCAGACAGGGCTCCCCGCACCG	Yes	Yes	Yes	0	0	0.0732862	No	
NM_hsa_75	chr22:23736550-23736678	-	TCAGACAGGGCTCCCCGCACCG	Yes	Yes	Yes	0	0	0.0732862	No	
NM_hsa_75	chr22:20136497-20136550	+	TCAGACAGGGCTCCCCGCACCG	Yes	Yes	Yes	0	0	0.0732862	No	
NM_hsa_754	chr6:5773857-5773938	+	TGAGGGAACAGAGGACTGAAGA	Yes	No	No	0	0	0	No	
NM_hsa_755	chr6:5773857-5773938	+	TTCAGGCCTCTGTTCCCTTGTC	Yes	No	No	0	0	0	No	
NM_hsa_756	chr1:44045061-44045116	+	CTCAGGAACGGACCAGGCCAGG	Yes	Yes	No	0	0	0	No	
NM_hsa_756	chr1:44045051-44045123	+	CTCAGGAACGGACCAGGCCAGG	Yes	Yes	No	0	0	0	No	
NM_hsa_757	chr1:44045061-44045116	+	TGGCCAGGTCTTCCTGGAGC	Yes	No	No	0	0	0	No	
NM_hsa_758	chr9:37804215-37804292	+	TCTTCAGGAACTCTGGCTAACT	Yes	Yes	No	0.0712322	0.0331113	0.146572	Yes	
NM_hsa_758	chr9:37804227-37804283	+	TCTTCAGGAACTCTGGCTAACT	Yes	Yes	No	0.0712322	0.0331113	0.146572	Yes	
NM_hsa_759	chr9:37804227-37804283	+	TTAGCCAGAGTTCCTAAAGAAA	Yes	No	No	0	0	0	Yes	
NM_hsa_76	chr22:23736584-23736637	-	GGCGGGGATCCCTGGCTTTGGGA	Yes	No	No	0	0	0	No	
NM_hsa_760	chr1:59574578-59574636	+	TAGAGAGAGGTCTGGACTAGA	Yes	Yes	Yes	0	0	0	No	
NM_hsa_760	chr1:59574564-59574650	+	TAGAGAGAGGTCTGGACTAGA	Yes	Yes	Yes	0	0	0	No	
NM_hsa_760	chr1:59574565-59574645	+	TAGAGAGAGGTCTGGACTAGA	Yes	Yes	Yes	0	0	0	No	
NM_hsa_761	chr1:59574578-59574636	+	TAGTCCAGACCTCTCTTCTACT	Yes	No	No	0	0	0	No	
NM_hsa_762	chr11:64037672-64037744	+	CCCACCAGGACTGGAAGACTCG	Yes	No	No	0	0	0.0732862	No	
NM_hsa_763	chr11:64037672-64037744	+	GAGCGAAGGTCACTGGGAGGGGG	Yes	No	No	0	0	0	No	
NM_hsa_764	chr16:820411-820506	+	AGTGGGGGTATGCTGGCTGTGA	Yes	Yes	No	0	0	0	No	
NM_hsa_764	chr16:820421-820493	+	AGTGGGGGTATGCTGGCTGTGA	Yes	Yes	No	0	0	0	No	
NM_hsa_765	chr16:820421-820493	+	ACAGCCCTGCACCCCCACCCC	Yes	No	No	0	0	0	No	
NM_hsa_766	chr20:60941171-60941255	+	CTAGAGCGGCCGGTTGGGAG	Yes	Yes	No	0	0	0	No	
NM_hsa_766	chr20:60941181-60941248	+	CTAGAGCGGCCGGTTGGGAG	Yes	Yes	No	0	0	0	No	
NM_hsa_766	chr20:60941121-60941201	+	CTAGAGCGGCCGGTTGGGAG	Yes	Yes	No	0	0	0	No	
NM_hsa_767	chr20:60941121-60941201	+	TACACAGGCCCTCAGGGC	Yes	No	No	0	0	0	No	
NM_hsa_768	chr20:12440504-12440632	+	CAAATGAGAACCCAGATATCTG	Yes	Yes	Yes	0	0	0	Yes	
NM_hsa_768	chr20:12440539-12440606	+	CAAATGAGAACCCAGATATCTG	Yes	Yes	Yes	0	0	0	Yes	
NM_hsa_768	chr20:12440529-12440614	+	CAAATGAGAACCCAGATATCTG	Yes	Yes	Yes	0	0	0	Yes	
NM_hsa_769	chr20:12440539-12440606	+	GATATCTGGTATATCTCATTTGAA	Yes	No	No	0	0	0	Yes	
NM_hsa_77	chr13:55161773-55161831	+	TTCATAGATTTCGTGTGTGTG	Yes	Yes	No	0	0	0	No	
NM_hsa_77	chr13:55161760-55161840	+	TTCATAGATTTCGTGTGTGTG	Yes	Yes	No	0	0	0	No	
NM_hsa_770	chr6:27219409-27219466	+	TCTGCACCCTCTGAGTCCCGCT	Yes	No	No	0	0	0	No	
NM_hsa_771	chr6:27219409-27219466	+	TGGGGAACCCAAGGAGGACGCAGGTC	Yes	No	No	0	0	0	No	
NM_hsa_772	chr4:103790501-103790588	+	CTGGGGAACGCCTGTGAGG	Yes	No	No	0	0	0	No	
NM_hsa_773	chr4:103790501-103790588	+	TCCGGTGTCATTCCGTGGC	Yes	No	No	0	0	0	No	
NM_hsa_774	chr6:21686164-21686292	+	TAGGAGCATGAACCTGGTGAGC	Yes	Yes	Yes	0.0569857	0	0.146572	Yes	
NM_hsa_774	chr6:21686199-21686258	+	TAGGAGCATGAACCTGGTGAGC	Yes	Yes	Yes	0.0569857	0	0.146572	Yes	
NM_hsa_774	chr6:21686189-21686266	+	TAGGAGCATGAACCTGGTGAGC	Yes	Yes	Yes	0.0569857	0	0.146572	Yes	
NM_hsa_775	chr6:21686199-21686258	+	ACCTAGGTTGCATGCTCTTTATG	Yes	No	No	0	0	0	No	
NM_hsa_776	chr22:24360166-24360216	-	TTTCCAGATGGAGGGCTGT	Yes	No	No	0	0	0	No	
NM_hsa_777	chr22:24360166-24360216	-	GGCCCTCCATCTGGAAACA	Yes	No	No	0	0	0	No	
NM_hsa_778	chr14:24541934-24541998	+	TCCCCTGCTCAGAACTCTGGTG	Yes	No	No	0	0	0	No	
NM_hsa_779	chr14:24541934-24541998	+	CAGGGCTTCTGAGCAGGAGTGGGAGG	Yes	No	No	0	0	0	Yes	
NM_hsa_78	chr13:55161773-55161831	+	CACACACAAAGTCTATGAATA	Yes	No	No	0	0	0	No	
NM_hsa_781	chr12:56546391-56546436	+	GGTGAGTTTTTGGGGGCTGAG	Yes	No	No	0	0	0	No	
NM_hsa_782	chr16:21831664-21831718	-	GTTGTTTGTCGGGAGATG	Yes	No	No	0	0	0	No	
NM_hsa_784	chr7:77166136-77166177	-	AGGGAATGCGACTCTGAGC	Yes	No	No	0.227943	0.132445	0.58629	No	
NM_hsa_785	chr7:77166136-77166177	-	GGAGAGTTGGTTCATTTG	Yes	No	No	0	0	0	No	
NM_hsa_786	chr19:50386150-50386226	+	GTGAGTGCCAGGACAGGTGGA	Yes	No	No	0	0	0	No	
NM_hsa_787	chr19:50386150-50386226	+	TGCCTGCCCTCCTGCACCC	Yes	No	No	0	0	0	No	
NM_hsa_79	chr5:173278563-173278621	+	GAGGGTCTGACTGTCACTTGGA	Yes	Yes	No	0	0	0	No	
NM_hsa_79	chr5:173278553-173278629	+	GAGGGTCTGACTGTCACTTGGA	Yes	Yes	No	0	0	0	No	
NM_hsa_790	chr2:161350460-161350523	+	CTCCCAGGCTCGCGGTTGGA	Yes	No	No	0	0	0	No	
NM_hsa_791	chr2:161350460-161350523	+	CAGATCGCTTTGTGTCGGGGGT	Yes	No	No	0	0	0	No	
NM_hsa_796	chr6:43745951-43746035	+	TGCAGCCATGTCTTGGCCTCAAG	Yes	Yes	Yes	0	0	0	Yes	
NM_hsa_796	chr6:43745931-43746051	+	TGCAGCCATGTCTTGGCCTCAAG	Yes	Yes	Yes	0	0	0	Yes	
NM_hsa_796	chr6:43745961-43746021	+	TGCAGCCATGTCTTGGCCTCAAG	Yes	Yes	Yes	0	0	0	Yes	
NM_hsa_797	chr6:43745961-43746021	+	TGAGGCCAGCAGGGTTGGGGG	Yes	No	No	0	0	0	No	
NM_hsa_798	chr15:20213635-20213709	+	ACTGGGGCTGTGTCACTGTGGG	Yes	No	No	0	0	0	No	
NM_hsa_799	chr15:20213635-20213709	+	CACAGTGACACAAGCCCCCACAA	Yes	No	No	0	0	0	No	
NM_hsa_8	chr14:94579956-94580074	+	AGGCCTCCAGACACACCGCAGC	Yes	Yes	Yes	0.170957	0.0662225	0.366431	No	
NM_hsa_8	chr14:94579986-94580044	+	AGGCCTCCAGACACACCGCAGC	Yes	Yes	Yes	0.170957	0.0662225	0.366431	No	
NM_hsa_8	chr14:94579976-94580053	+	AGGCCTCCAGACACACCGCAGC	Yes	Yes	Yes	0.170957	0.0662225	0.366431	No	
NM_hsa_80	chr5:173278563-173278621	+	CAAGTGGCCATCAGACCCTCTT	Yes	No	No	0	0	0	No	
NM_hsa_800	chr7:27155186-27155241	+	CTGGGCCAAGGGGACCGAGGGC	Yes	No	No	0.341914	0	0.146572	No	

Additional file 5

Name	locus	strand	sequence	mirdeep2	mireap	miranalyzer	RPM_HU_KapB_cMyc	RPM_HU_KaposinB	RPM_HUVEC	IP_confidence	annotation
NM_hsa_801	chr7:27155186-27155241	+	TCTCCGTAAGAACCTGCCAGGGG	Yes	No	No	0	0	0	No	
NM_hsa_802	chr19:4215924-4215976	+	ACTCTGCTCCCTCCCCCCAGA	Yes	No	Yes	0	0	0	No	
NM_hsa_802	chr19:4215899-4215995	+	ACTCTGCTCCCTCCCCCCAGA	Yes	No	Yes	0	0	0	No	
NM_hsa_803	chr19:4215924-4215976	+	CACGGGGGCTGAGAGCAGAACC	Yes	No	No	0	0	0.0732862	Yes	
NM_hsa_804	chr16:78414402-78414452	+	ACTCTTTAAGGATAGGGCTGA	Yes	Yes	No	0	0	0	Yes	
NM_hsa_804	chr16:78414389-78414461	+	ACTCTTTAAGGATAGGGCTGA	Yes	Yes	No	0	0	0	Yes	
NM_hsa_805	chr16:78414402-78414452	+	AGCCGTATCAGTGAAGAGTGA	Yes	No	No	0	0	0	No	
NM_hsa_806	chr8:71365470-71365551	+	CTTCCCCTTACCCTGGCGTTGG	Yes	Yes	Yes	0	0	0	No	
NM_hsa_806	chr8:71365480-71365543	+	CTTCCCCTTACCCTGGCGTTGG	Yes	Yes	Yes	0	0	0	No	
NM_hsa_806	chr8:71365445-71365573	+	CTTCCCCTTACCCTGGCGTTGG	Yes	Yes	Yes	0	0	0	No	
NM_hsa_807	chr8:71365480-71365543	+	AATGTCAGAGTCTGGGGCAGGT	Yes	No	No	0	0	0	No	
NM_hsa_808	chr22:31284149-31284211	+	AAGCTCTGTCTGTGTCTCTAGG	Yes	No	No	0	0.132445	0	Yes	
NM_hsa_809	chr22:31284149-31284211	+	TAGGGAGGCGAGATTGAGCCTTT	Yes	No	No	0	0	0	Yes	
NM_hsa_812	chr19:10946715-10946755	+	CGGCGCGGACTGGTAGAAA	Yes	No	No	0	0	0	No	
NM_hsa_813	chr19:10946715-10946755	+	ACTGCTTCCTCCCCGCCGGC	Yes	No	No	0	0	0	No	
NM_hsa_814	chr3:47453513-47453585	+	TGGCCCCATCCTGTCCCACAGA	Yes	No	No	0	0	0	No	
NM_hsa_815	chr3:47453513-47453585	+	TGCTGGGAGGGATGAGAGCCTCAGG	Yes	No	No	0	0	0	No	
NM_hsa_816	chr4:1022123-1022167	+	ACCCCAAGGAGTCAGGACAGAC	Yes	No	No	0	0	0	No	
NM_hsa_817	chr4:1022123-1022167	+	ATCTCCTGCCTATGGGGCCC	Yes	No	No	0	0	0	No	
NM_hsa_818	chr8:124171290-124171350	+	TTTCTCCCAGATCTGCAGGCCCGA	Yes	No	No	0.113971	0	0	No	
NM_hsa_819	chr8:124171290-124171350	+	GGACTGCGGACTGGGAGTGTCA	Yes	No	No	0	0	0	No	
NM_hsa_820	chr17:56412670-56412723	+	AGAGGGATCTGGGCTTGGAAT	Yes	Yes	No	0	0	0	Yes	
NM_hsa_820	chr17:56412660-56412751	+	AGAGGGATCTGGGCTTGGAAT	Yes	Yes	No	0	0	0	Yes	
NM_hsa_821	chr17:56412670-56412723	+	TCCAGGCTCATGGTCCCTCACT	Yes	No	No	0	0	0	No	
NM_hsa_822	chr12:124921989-124922046	-	CCCCCTTTCCCTGAGCCTGC	Yes	Yes	No	0	0	0	No	
NM_hsa_822	chr12:124921980-124922058	-	CCCCCTTTCCCTGAGCCTGC	Yes	Yes	No	0	0	0	No	
NM_hsa_823	chr12:124921989-124922046	-	AGGCTCAGGGAAACTGAGAC	Yes	No	No	0	0	0	No	
NM_hsa_824	chr9:97553116-97553194	+	AATTTGGTCAGTGATTGGATGT	Yes	Yes	No	0	0	0	No	
NM_hsa_824	chr9:97553128-97553185	+	AATTTGGTCAGTGATTGGATGT	Yes	Yes	No	0	0	0	No	
NM_hsa_825	chr9:97553128-97553185	+	ATCCAATCACCGACCAAATTGG	Yes	No	No	0	0	0	No	
NM_hsa_826	chr15:41861017-41861078	+	CCCAGCTGGAGGCCTTCTCCC	Yes	No	No	0	0	0	No	
NM_hsa_827	chr15:41861017-41861078	+	GTGGGGGCTTTTCCTGGCTGCAGGA	Yes	No	No	0	0	0	No	
NM_hsa_828	chr16:28858317-28858378	+	TGCTAGACCTCACAGGGCCAGA	Yes	Yes	Yes	0	0.132445	0	No	
NM_hsa_828	chr16:28858294-28858387	+	TGCTAGACCTCACAGGGCCAGA	Yes	Yes	Yes	0	0.132445	0	No	
NM_hsa_828	chr16:28858284-28858412	+	TGCTAGACCTCACAGGGCCAGA	Yes	Yes	Yes	0	0.132445	0	No	
NM_hsa_829	chr16:28858317-28858378	+	GGGCCCTGTGAGGTCAGGCAGC	Yes	No	No	0	0	0	No	
NM_hsa_83	chr22:31502901-31502999	-	AGGGCTGGAGGCTCCAGAAAGTC	Yes	Yes	Yes	0	0	0	No	
NM_hsa_83	chr22:31502889-31503029	-	AGGGCTGGAGGCTCCAGAAAGTC	Yes	Yes	Yes	0	0	0	No	
NM_hsa_83	chr22:31502914-31502989	-	AGGGCTGGAGGCTCCAGAAAGTC	Yes	Yes	Yes	0	0	0	No	
NM_hsa_830	chr3:48357870-48357935	+	TCTAGCCTCCATGAATTCTGAT	Yes	Yes	No	0.370407	0.430446	0.659576	Yes	H>KapB>KapB+cMyc
NM_hsa_830	chr3:48357860-48357943	+	TCTAGCCTCCATGAATTCTGAT	Yes	Yes	No	0.370407	0.430446	0.659576	Yes	H>KapB>KapB+cMyc
NM_hsa_831	chr3:48357870-48357935	+	CAGGAGTCATGGAAGCTGGAAG	Yes	No	No	0	0	0	No	
NM_hsa_833	chr16:29985169-29985251	+	CCTGCGCGCTCCCGCCC	Yes	No	No	0	0	0	No	
NM_hsa_836	chr19:54651796-54651892	+	TCTGCTCTCTCCCACCCGCAG	Yes	Yes	No	0.0569857	0	0.0732862	Yes	
NM_hsa_836	chr19:54651814-54651883	+	TCTGCTCTCTCCCACCCGCAG	Yes	Yes	No	0.0569857	0	0.0732862	Yes	
NM_hsa_837	chr19:54651814-54651883	+	GAGGTGGGGGTGGTGAGG	Yes	No	No	0	0	0	No	
NM_hsa_839	chr20:32951086-32951167	+	CCCGAGTCCCGGTAAACCCCCG	Yes	No	No	0	0	0	No	
NM_hsa_84	chr22:31502914-31502989	-	CTTTCTGGGTGCCTCCTCCCATA	Yes	No	No	0	0	0	Yes	
NM_hsa_840	chr7:8249160-8249230	+	TTGGGTTCGAGGATGTGTAAGA	Yes	Yes	No	0	0	0	No	
NM_hsa_840	chr7:8249170-8249222	+	TTGGGTTCGAGGATGTGTAAGA	Yes	Yes	No	0	0	0	No	
NM_hsa_841	chr7:8249170-8249222	+	TTACACATTCTTGAACCCCAAT	Yes	No	No	0	0	0	No	
NM_hsa_842	chr21:30444343-30444477	-	AAATGCCAGGCTCAGGGACT	Yes	Yes	Yes	0	0	0	No	
NM_hsa_842	chr21:30444339-30444412	-	AAATGCCAGGCTCAGGGACT	Yes	Yes	Yes	0	0	0	No	
NM_hsa_842	chr21:30444382-30444445	-	AAATGCCAGGCTCAGGGACT	Yes	Yes	Yes	0	0	0	No	
NM_hsa_843	chr21:30444382-30444445	-	CCCCTGAGCCTGGGCTGTTTCC	Yes	No	No	0	0	0	No	
NM_hsa_846	chr12:41844865-41844930	+	GAGAAAGTCAGACCGTAACAGT	Yes	Yes	No	0	0	0	No	
NM_hsa_846	chr12:41844853-41844939	+	GAGAAAGTCAGACCGTAACAGT	Yes	Yes	No	0	0	0	No	
NM_hsa_847	chr12:41844865-41844930	+	TGTTATAGTCTAACTTTCTCTT	Yes	No	No	0	0	0	No	
NM_hsa_849	chr11:43902347-43902407	+	GCCTGTGATTGGGAGTTGC	Yes	No	No	0	0	0	No	
NM_hsa_85	chrX:44855632-44855718	-	AGTGACAAGGCTGTGATTCTGA	Yes	Yes	Yes	0	0	0	Yes	
NM_hsa_85	chrX:44855625-44855733	-	AGTGACAAGGCTGTGATTCTGA	Yes	Yes	Yes	0	0	0	Yes	
NM_hsa_85	chrX:44855640-44855708	-	AGTGACAAGGCTGTGATTCTGA	Yes	Yes	Yes	0	0	0	Yes	
NM_hsa_850	chr14:105147019-105147147	+	CTGGGCTTGTGGGGACCCCTGG	Yes	No	Yes	0.341914	0.397335	0.366431	Yes	
NM_hsa_850	chr14:105147059-105147113	+	CTGGGCTTGTGGGGACCCCTGG	Yes	No	Yes	0.341914	0.397335	0.366431	Yes	
NM_hsa_851	chr14:105147059-105147113	+	AGGGATCCCCAGACAGCCCCGAGCT	Yes	No	No	0	0	0	No	
NM_hsa_852	chr1:9886185-9886243	+	AAATACCAGGCTTTATGTGG	Yes	Yes	No	0	0	0	No	
NM_hsa_852	chr1:9886175-9886250	+	AAATACCAGGCTTTATGTGG	Yes	Yes	No	0	0	0	No	
NM_hsa_853	chr1:9886185-9886243	+	ACATAGAGCCTGGTGTTTCT	Yes	No	No	0	0	0	No	
NM_hsa_854	chr9:98645103-98645177	+	TGATTGTTTGTGGAATAAATGA	Yes	Yes	Yes	0	0	0	Yes	
NM_hsa_854	chr9:98645113-98645170	+	TGATTGTTTGTGGAATAAATGA	Yes	Yes	Yes	0	0	0	Yes	
NM_hsa_854	chr9:98645083-98645201	+	TGATTGTTTGTGGAATAAATGA	Yes	Yes	Yes	0	0	0	Yes	
NM_hsa_855	chr9:98645113-98645170	+	GTTTATTCCACAAACTATCATT	Yes	No	No	0	0	0	Yes	
NM_hsa_856	chr5:98268282-98268336	+	ACTGTTGGTCCTCTGTACCTGC	Yes	No	No	0	0.0662225	0	No	
NM_hsa_857	chr5:98268282-98268336	+	AGGTACAGAGGCTCAACTATAA	Yes	No	No	0.0569857	0	0	No	
NM_hsa_858	chr10:43932339-43932404	+	CGTGGGGAAGCCGGACTG	Yes	No	No	0	0	0	No	
NM_hsa_859	chr10:43932339-43932404	+	GTCCGGCCAGCGGA	Yes	No	No	0	0	0	No	
NM_hsa_86	chrX:44855640-44855708	-	AGAATCAAAGCCTTGTCACTTA	Yes	No	No	0	0	0	No	
NM_hsa_860	chr22:46773632-46773722	-	TGAAGCTGACCCGCCTCTGCC	Yes	Yes	No	0.0569857	0	0	No	
NM_hsa_860	chr22:46773641-46773699	-	TGAAGCTGACCCGCCTCTGCC	Yes	Yes	No	0.0569857	0	0	No	
NM_hsa_861	chr22:46773641-46773699	-	CTGGAGGAGGGCAGCGGCCTC	Yes	No	No	0	0	0	No	
NM_hsa_862	chr1:45273867-45273946	+	AAGCCAGGTCGGGGCCCTGACC	Yes	No	No	0	0	0	No	
NM_hsa_863	chr1:45273867-45273946	+	TTTCCCCGGCCTGGCTAAC	Yes	No	No	0	0	0	No	
NM_hsa_864	chr10:103662357-103662414	-	CTTTCTACCACAGCTCTTTGTT	Yes	Yes	Yes	0	0	0	Yes	
NM_hsa_864	chr10:103662338-103662436	-	CTTTCTACCACAGCTCTTTGTT	Yes	Yes	Yes	0	0	0	Yes	
NM_hsa_864	chr10:103662348-103662432	-	CTTTCTACCACAGCTCTTTGTT	Yes	Yes	Yes	0	0	0	Yes	
NM_hsa_865	chr10:103662357-103662414	-	CAAAGAGCTGTGGTAGAAAGTA	Yes	No	No	0	0	0	No	
NM_hsa_866	chr19:39282615-39282690	+	ATGCGGTCGGGCGGACCTG	Yes	No	No	0	0	0	No	
NM_hsa_867	chr19:39282615-39282690	+	GGTCCTGGTGCCCCCACTGCAGAT	Yes	No	No	0	0	0	No	
NM_hsa_868	chr19:35652419-35652495	+	TCATGTCTGAACCAATGAGAGC	Yes	Yes	No	0.256436	0.0331113	0	Yes	KapB+cMyc>KapB>H
NM_hsa_868	chr19:35652429-35652488	+	TCATGTCTGAACCAATGAGAGC	Yes	Yes	No	0.256436	0.0331113	0	Yes	KapB+cMyc>KapB>H
NM_hsa_868	chr19:35652401-35652451	+	TCATGTCTGAACCAATGAGAGC	Yes	Yes	No	0.256436	0.0331113	0	Yes	KapB+cMyc>KapB>H
NM_hsa_869	chr19:35652429-35652488	+	TCTCATTGGTCAGGCCTGAGT	Yes	No	No	0	0	0	No	
NM_hsa_870	chr19:38882791-38882853	+	CTGCCTCTCTCCCTGCCCCAGG	Yes	No	No	0	0.0662225	0.0732862	No	H>KapB>KapB+cMyc
NM_hsa_871	chr19:38882791-38882853	+	TGGGAGCGGGAGGAGAAGCTGGG	Yes	No	No	0	0	0	No	
NM_hsa_872	chr1:31627900-31627959	+	CTGGATCTTCGGAACGAG	Yes	No	No	0	0	0	No	
NM_hsa_873	chr1:31627900-31627959	+	AGGGCGGGGAGCGGCC	Yes	No	No	0	0	0	No	
NM_hsa_874	chr11:132590114-132590174	-	TGGACTGTGGCTCAGGGCCTGG	Yes	Yes	Yes	0	0	0	No	
NM_hsa_874	chr11:132590090-132590198	-	TGGACTGTGGCTCAGGGCCTGG	Yes	Yes	Yes	0	0	0	No	
NM_hsa_874	chr11:132590105-132590203	-	TGGACTGTGGCTCAGGGCCTGG	Yes	Yes	Yes	0	0	0	No	
NM_hsa_875	chr11:132590114-132590174	-	AGCTCCTGGGGTACAGATGCCATC	Yes	No	No	0	0	0	No	
NM_hsa_876	chr17:5015332-5015399	+	CTGCACGACTGGACTGGAG	Yes	No	No	0	0	0	No	
NM_hsa_877	chr17:5015332-5015399	+	GCAGTCCTTGCGCAGGT	Yes	No	No	0	0	0	No	
NM_hsa_878	chr2:36596670-36596747	-	AGTTCTGTAGAAATGTTTGCA	Yes	Yes	No	0	0	0	No	
NM_hsa_878	chr2:36596677-36596737	-	AGTTCTGTAGAAATGTTTGCA	Yes	Yes	No	0	0	0	No	
NM_hsa_879	chr2:36596677-36596737	-	CAAACATTTTTACAGAACTTT	Yes	No	No	0	0	0	No	
NM_hsa_88	chr7:114562189-114562238	+	GGGTGGGGAGCCCGAGCCAGC	Yes	No	No	0	0	0	No	
NM_hsa_880	chr6:89791338-89791420	+	TGCGCTGGCGCTTCTGATTGG	Yes	Yes	No	0	0	0	No	
NM_hsa_880	chr6:89791348-89791403	+	TGCGCTGGCGCTTCTGATTGG	Yes	Yes	No	0	0	0	No	
NM_hsa_881	chr6:89791348-89791403	+	AATGAGAAGCGCCGCTGC	Yes	No	No	0	0	0	No	
NM_hsa_882	chr1:154781495-154781553	+	TTACTGATCTCTGGTTTGTGG	Yes	Yes	No	0	0	0	No	
NM_hsa_882	chr1:154781485-154781560	+	TTACTGATCTCTGGTTTGTGG	Yes	Yes	No	0	0	0	No	
NM_hsa_883	chr1:154781495-154781553	+	ACAGGTCAGTGGTCATAGCC	Yes	No	No	0	0	0	No	
NM_hsa_884	chr1:150207085-150207139	+	TGAGGCCGAGAAGGCAACCGCGA	Yes	Yes	Yes	0.455886	0.397335	0.146572	Yes	KapB+cMyc>KapB>H
NM_hsa_884	chr1:150207071-150207148	+	TGAGGCCGAGAAGGCAACCGCGA	Yes	Yes	Yes	0.455886	0.397335	0.146572	Yes	KapB+cMyc>KapB>H
NM_hsa_884	chr1:150207063-150207153	+	TGAGGCCGAGAAGGCAACCGCGA	Yes	Yes	Yes	0.455886	0.397335	0.146572	Yes	KapB+cMyc>KapB>H
NM_hsa_885	chr1:150207085-150207139	+	GCGTTGTCCTCGCGCCTTCGCC	Yes	No	No	0	0	0	No	
NM_hsa_886	chr7:99647443-99647530	+	CTCTGATTCATGGTGAGCCTG	Yes	Yes	No	0	0	0	No	
NM_hsa_886	chr7:99647453-99647512	+	CTCTGATTCATGGTGAGCCTG	Yes	Yes	No	0	0	0	No	
NM_hsa_887	chr7:99647453-99647512	+	GGCGGACAGGAGAGGGAGCC	Yes	No	No	0	0	0.0732862	No	
NM_hsa_89	chr10:125670967-125671103	-	GAAAGTAATGGTGGGTTTTGC	Yes	Yes	Yes	0	0	0	Yes	
NM_hsa_89	chr10:125670997-125671073	-	GAAAGTAATGGTGGGTTTTGC	Yes	Yes	Yes	0	0	0	Yes	
NM_hsa_892	chr14:102553275-102553339	-	GTGGACTGGGGACCGGGA	Yes	No	No	0	0	0	No	
NM_hsa_893	chr14:102553275-102553339	-	GCGGTCACTTAGCCAAGG	Yes	No	No	0	0	0	No	
NM_hsa_894	chr12:53856935-53857015	+	TGGGCTGTGGTGCCGTGACCTTT	Yes	Yes	No	0	0.0662225	0.439717	Yes	H>KapB>KapB+cMyc
NM_hsa_894	chr12:53856945-53857009	+	TGGGCTGTGGTGCCGTGACCTTT	Yes	Yes	No	0	0.0662225	0.439717	Yes	H>KapB>KapB+cMyc
NM_hsa_895	chr12:53856945-53857009	+	AGCACCACCGTGGCCTCTC	Yes	No	No	0	0	0	No	
NM_hsa_897	chr1:162467592-162467671	+	TCCATTTCCGGCTTCTGGGACTCGG	Yes	No	No	0	0	0	No	
NM_hsa_898	chr15:75495340-75495407	+	AGAGCCCAGAGCCTCAGGTAG	Yes	Yes	No	0	0	0	No	
NM_hsa_898	chr15:75495328-75495416	+	AGAGCCCAGAGCCTCAGGTAG	Yes	Yes	No	0	0	0	No	
NM_hsa_899	chr15:75495340-75495407	+	TCCACTGGAGACTTTGGGCTGCCCC	Yes	No	No	0.455886	0.463558	0.0732862	Yes	
NM_hsa_900	chr11:721514-721566	+	CTGACCCCCTCTGACCCCAGC	Yes	No	No	0	0	0	No	
NM_hsa_901	chr11:721514-721566	+	CGGGGCGGTGGGGAGTGTCAGGG	Yes	No	No	0	0	0	No	
NM_hsa_902	chr10:51558538-51558610	+	GAGGGGCCAAGGAATCTGAATT	Yes	Yes	No	0	0	0	No	
NM_hsa_902	chr10:51558526-51558619	+	GAGGGGCCAAGGAATCTGAATT	Yes	Yes	No	0	0	0	No	
NM_hsa_903	chr10:51558538-51558610	+	TACAGATTCCTGGGCTCCATCCC	Yes	No	No	0	0	0	No	
NM_hsa_904	chr6:127588386-127588451	-	CACCCAGGGCAGGGGCTGCGG	Yes	No	No	0	0	0	No	
NM_hsa_904	chr6:127588319-127588407	-	CACCCAGGGCAGGGGCTGCGG	Yes	No	No	0	0	0	No	
NM_hsa_905	chr6:127588319-127588407	-	GCAGCCAAGGCGGGGGTGGC	Yes	No	No	0	0	0	No	
NM_hsa_906	chr2:85935682-85935763	-	CATCTATGAACACTGAAGTTA	Yes	Yes	Yes	0	0	0	No	
NM_hsa_906	chr2:85935691-85935751	-	CATCTATGAACACTGAAGTTA	Yes	Yes	Yes	0	0	0	No	
NM_hsa_906	chr2:85935677-85935763	-	CATCTATGAACACTGAAGTTA	Yes	Yes	Yes	0	0	0	No	
NM_hsa_907	chr2:85935691-85935751	-	ACTTCAGTGTTCATTGATGGT	Yes	No	No	0	0	0	No	
NM_hsa_908	chr4:188884701-188884790	+	TAATATTGTTCTCTGAGCAAGC	Yes	Yes	No	0	0	0	No	
NM_hsa_908	chr4:188884713-188884781	+	TAATATTGTTCTCTGAGCAAGC	Yes	Yes	No	0	0	0	No	
NM_hsa_909	chr4:188884713-188884781	+	TTGCTCAGAGAACAATATTGTA	Yes	No	No	0	0	0	No	
NM_hsa_91	chrX:153038286-153038342	+	CAGCCTGCGCTGTTCGCCAGG	Yes	No	No	0	0	0	No	
NM_hsa_910	chr11:64128547-64128607	+	GCACCCCTCTTGCTCCTACCAGC	Yes	No	No	0	0	0	Yes	
NM_hsa_911	chr11:64128547-64128607	+	TGGAGGAGCTGGGGAGGGTTTCG	Yes	No	No	0	0	0	No	
NM_hsa_912	chr22:42477769-42477866	+	TGGGTCTGAGGGTATGTCTGTGA	Yes	Yes	No	0	0	0	No	
NM_hsa_912	chr22:42477834-42477925	+	TGGGTCTGAGGGTATGTCTGTGA	Yes	Yes	No	0	0	0	No	
NM_hsa_913	chr22:42477834-42477925	+	ACAGCTGCCACTCTGACCCTCT	Yes	No	No	0	0	0	No	
NM_hsa_914	chr12:122905560-122905650	-	TTCTGACTTCAGGTGCTTAGT	Yes	Yes	No	0	0	0	No	
NM_hsa_914	chr12:122905569-122905638	-	TTCTGACTTCAGGTGCTTAGT	Yes	Yes	No	0	0	0	No	
NM_hsa_915	chr12:122905569-122905638	-	TAAGCACCTGAAGTCAGAATA	Yes	No	No	0	0	0	No	
NM_hsa_916	chrY:1464284-1464338	+	CAGCAGGGTGGTGCTGTCTGT	Yes	Yes	No	0.0569857	0	0.0732862	Yes	
NM_hsa_916	chrX:1514307-1514399	+	CAGCAGGGTGGTGCTGTCTGT	Yes	Yes	No	0.0569857	0	0.0732862	Yes	
NM_hsa_917	chrY:1464284-1464338	+	AGCCTGCACTGGCTTGGTGGC	Yes	No	No	0	0	0	Yes	
NM_hsa_918	chr19:16198771-16198838	+	TGGGCCTTTCTGTCTCTGCAGG	Yes	No	No	0.227943	0	0.366431	Yes	

Additional file 5

Name	locus	strand	sequence	mirdeep2	mireap	miranalyzer	RPM_HU_KapB_cMyc	RPM_HU_KaposinB	RPM_HUVEC	IP_confidence	annotation
NM_hsa_919	chr19:16198771-16198838	+	TGCAGTGGATGGGAGAGGACACGGC	Yes	No	No	0	0	0	No	
NM_hsa_920	chr17:39676693-39676783	+	TCGTTGGGCATCAGAACATG	Yes	Yes	No	0	0	0	No	
NM_hsa_920	chr17:39676703-39676760	+	TCGTTGGGCATCAGAACATG	Yes	Yes	No	0	0	0	No	
NM_hsa_921	chr17:39676703-39676760	+	TGTTCTTAGGCTCAACGGCA	Yes	No	No	0	0	0	No	
NM_hsa_922	chr2:219292454-219292543	+	TAAGGTTGCTCTCACTGTCAGG	Yes	No	No	0	0	0	No	
NM_hsa_923	chr2:219292454-219292543	+	TGACAGTGAGTGACTCCCTAAA	Yes	No	No	0	0	0	No	
NM_hsa_924	chr10:31607430-31607485	+	ACTTCCCATCCCGGTTCGCTTGG	Yes	No	No	0	0	0	No	
NM_hsa_925	chr10:31607430-31607485	+	AGGCGTCCCTGGAAGGGAAGGGA	Yes	No	No	0	0	0	Yes	
NM_hsa_926	chr8:59223570-59223668	+	TTCATTCCAGGTCAGTCAGAGT	Yes	Yes	No	0	0	0	No	
NM_hsa_926	chr8:59223581-59223659	+	TTCATTCCAGGTCAGTCAGAGT	Yes	Yes	No	0	0	0	No	
NM_hsa_927	chr8:59223581-59223659	+	TCTGACTGACCTGGAATGAATA	Yes	No	No	0	0	0	No	
NM_hsa_928	chr17:26322115-26322181	+	AGCTGTGATGACTTTTGCACCAG	Yes	Yes	No	0	0	0	No	
NM_hsa_928	chr17:26322102-26322190	+	AGCTGTGATGACTTTTGCACCAG	Yes	Yes	No	0	0	0	No	
NM_hsa_929	chr17:26322115-26322181	+	GGTGCAAAAGTCATTGCGTTTT	Yes	No	No	0	0	0	No	
NM_hsa_93	chr17:18066989-18067053	+	AGTGGGGCCAGAAGGACAGAG	Yes	Yes	No	0	0	0	No	
NM_hsa_93	chr17:18066979-18067064	+	AGTGGGGCCAGAAGGACAGAG	Yes	Yes	No	0	0	0	No	
NM_hsa_930	chr16:54127346-54127437	+	CTGTGTTCTCCTGACTCACCGC	Yes	Yes	No	0	0	0	No	
NM_hsa_930	chr16:54127373-54127428	+	CTGTGTTCTCCTGACTCACCGC	Yes	Yes	No	0	0	0	No	
NM_hsa_931	chr16:54127373-54127428	+	GGCAGGAGGTCACAGCA	Yes	No	No	0	0	0	No	
NM_hsa_932	chr12:125010972-125011022	-	CTCCAGACTCCAGGTCCTGTA	Yes	No	No	0.0189952	0.0662225	0.0610719	Yes	
NM_hsa_933	chr12:125010972-125011022	-	TGGGGCCTGAACCCTTGGGAT	Yes	No	No	0	0	0	No	
NM_hsa_934	chr15:78566479-78566540	+	CTGCCTCCCCTGACCCTGCAGG	Yes	No	No	0	0	0	No	
NM_hsa_935	chr15:78566479-78566540	+	AGCAGGGACACTGGTAAGGGA	Yes	No	No	0	0	0	No	
NM_hsa_936	chr16:30670064-30670131	+	ACTGAACTCGGCGGACTG	Yes	No	No	0	0	0	No	
NM_hsa_938	chr10:104185998-104186048	-	TTGGGAGTCAAAAGCATGTAGA	Yes	Yes	No	0	0	0	Yes	
NM_hsa_938	chr10:104185989-104186064	-	TTGGGAGTCAAAAGCATGTAGA	Yes	Yes	No	0	0	0	Yes	
NM_hsa_939	chr10:104185998-104186048	-	CATATGCTCCTGACTCCCAAAC	Yes	No	No	0	0	0	No	
NM_hsa_94	chr17:18066989-18067053	+	CATCTCTTCCGCCCCACCCC	Yes	No	No	0	0	0	No	
NM_hsa_940	chr3:52557363-52557451	+	CAACAGGCCTTGCTCTGCTCACA	Yes	Yes	No	0.626843	0.927115	2.19859	No	H>KapB>KapB+cMyc
NM_hsa_940	chr3:52557374-52557442	+	CAACAGGCCTTGCTCTGCTCACA	Yes	Yes	No	0.626843	0.927115	2.19859	No	H>KapB>KapB+cMyc
NM_hsa_941	chr3:52557374-52557442	+	TGGGCAGAGCAGAGCCTGCATTGGC	Yes	No	No	0.0569857	0	0.293145	No	
NM_hsa_942	chr16:72142304-72142384	+	TGTTGGGATGAGGGGAGGGTCG	Yes	No	No	0	0	0	No	
NM_hsa_943	chr16:72142304-72142384	+	TCTCCTGCCCTAGGTCCGGGAGGTG	Yes	No	No	0	0	0	No	
NM_hsa_944	chr12:126468276-126468346	+	CTTAAAACTGGCCAGGGGTC	Yes	No	No	0	0	0	No	
NM_hsa_945	chr12:126468276-126468346	+	CCTCTCCCACAGTAAGAAGGGG	Yes	No	No	0	0	0	No	
NM_hsa_946	chr2:155586576-155586629	-	AGAGTTATAAGCAGAAAGCAGC	Yes	No	No	0	0	0	No	
NM_hsa_949	chr3:47043351-47043416	+	GTGAGATGTCGGGAGCTCC	Yes	No	No	0	0	0.0732862	No	
NM_hsa_95	chr2:28563452-28563533	+	ACTGAGCCCGGACTTGCTTCTG	Yes	Yes	No	0	0	0	No	
NM_hsa_95	chr2:28563462-28563522	+	ACTGAGCCCGGACTTGCTTCTG	Yes	Yes	No	0	0	0	No	
NM_hsa_950	chr3:47043351-47043416	+	TGCGCCCACAGCTGCAT	Yes	No	No	0	0	0	No	
NM_hsa_953	chr6:35453806-35453875	+	TATACGGGCTGTGGGAGTCA	Yes	Yes	No	0	0	0	No	
NM_hsa_953	chr6:35453818-35453866	+	TATACGGGCTGTGGGAGTCA	Yes	Yes	No	0	0	0	No	
NM_hsa_954	chr6:35453818-35453866	+	ACCTCCCACAGCCCGCACTGGA	Yes	No	No	0	0	0	No	
NM_hsa_959	chr6:31621155-31621216	+	AACCTTTCTGGGATTCGTAGT	Yes	Yes	Yes	0	0	0	No	
NM_hsa_959	chr6:31621129-31621245	+	AACCTTTCTGGGATTCGTAGT	Yes	Yes	Yes	0	0	0	No	
NM_hsa_959	chr6:31621133-31621225	+	AACCTTTCTGGGATTCGTAGT	Yes	Yes	Yes	0	0	0	No	
NM_hsa_96	chr2:28563462-28563522	+	GAAGCAAGCCCAGGCTCAGTCA	Yes	No	No	0	0	0	No	
NM_hsa_960	chr6:31621155-31621216	+	TGCGAGTCCTCCAGAAAGACACA	Yes	No	No	0	0	0	No	
NM_hsa_961	chr4:185306082-185306150	+	ATAACTCTGGCCCCAGGCACGT	Yes	Yes	No	0	0	0	No	
NM_hsa_961	chr4:185306072-185306159	+	ATAACTCTGGCCCCAGGCACGT	Yes	Yes	No	0	0	0	No	
NM_hsa_962	chr4:185306082-185306150	+	CAGCCTGGGGACAGAGGGAGGTCA	Yes	No	No	0	0	0	No	
NM_hsa_965	chr13:33161420-33161558	+	AAGAGCCCAGCACTGGCGTGGA	Yes	No	Yes	0	0	0	No	
NM_hsa_965	chr13:33161460-33161520	+	AAGAGCCCAGCACTGGCGTGGA	Yes	No	Yes	0	0	0	No	
NM_hsa_966	chr13:33161460-33161520	+	CAGGCCACAACTGGGTTCTAGCTTTCC	Yes	No	No	0	0	0	No	
NM_hsa_967	chr14:69356853-69356892	-	ACGAGGCCTGGACTGACGGT	Yes	No	No	0	0.0662225	0	No	
NM_hsa_967	chr19:39208678-39208717	+	ACGAGGCCTGGACTGACGGT	Yes	No	No	0	0.0662225	0	No	
NM_hsa_969	chr17:37043284-37043351	+	CAGGGTTTTGCAGACCGGGTCT	Yes	Yes	Yes	0.284929	0.26489	0.0732862	Yes	KapB+cMyc>KapB>H
NM_hsa_969	chr17:37043257-37043385	+	CAGGGTTTTGCAGACCGGGTCT	Yes	Yes	Yes	0.284929	0.26489	0.0732862	Yes	KapB+cMyc>KapB>H
NM_hsa_969	chr17:37043272-37043360	+	CAGGGTTTTGCAGACCGGGTCT	Yes	Yes	Yes	0.284929	0.26489	0.0732862	Yes	KapB+cMyc>KapB>H
NM_hsa_97	chr8:85320827-85320911	+	ACAGCTGGGTTCCTGAATGATT	Yes	Yes	Yes	0	0	0	No	
NM_hsa_97	chr8:85320839-85320902	+	ACAGCTGGGTTCCTGAATGATT	Yes	Yes	Yes	0	0	0	No	
NM_hsa_97	chr8:85320803-85320941	+	ACAGCTGGGTTCCTGAATGATT	Yes	Yes	Yes	0	0	0	No	
NM_hsa_970	chr17:37043284-37043351	+	GCCTGGCCTGTTTTAACCCTGTG	Yes	No	No	0.170957	0.26489	0	Yes	
NM_hsa_971	chr14:23342455-23342521	+	TGTCCTACCTTCCTCTCTCCAGC	Yes	No	No	0	0	0	Yes	
NM_hsa_972	chr14:23342455-23342521	+	CGGAAGGGGAAGGGTGGGATGCA	Yes	No	No	0	0	0	No	
NM_hsa_973	chr2:101618597-101618657	-	GAGCTATGATGTAAGCGG	Yes	No	No	0	0	0	No	
NM_hsa_974	chr4:25661109-25661198	+	TCATGAACAATAAACAAGAGTT	Yes	Yes	No	0	0	0	No	
NM_hsa_974	chr4:25661125-25661189	+	TCATGAACAATAAACAAGAGTT	Yes	Yes	No	0	0	0	No	
NM_hsa_975	chr4:25661125-25661189	+	CTCTTGTTTATTGTTCATGACA	Yes	No	No	0	0	0	No	
NM_hsa_976	chr14:23447395-23447447	-	GAGGTCCGCAGCTGCTCTGC	Yes	No	No	0	0	0	No	
NM_hsa_977	chr14:23447395-23447447	-	AGAGCAGCTGTTGGATCCCCA	Yes	No	No	0	0	0	No	
NM_hsa_98	chr8:85320839-85320902	+	TCATTCAAGAACCCAGGTGGGTTG	Yes	No	No	0	0	0	No	
NM_hsa_980	chr2:230195439-230195491	-	TTTATTTCAAAGGACAGCTGG	Yes	Yes	No	0	0	0	No	
NM_hsa_980	chr2:230195430-230195502	-	TTTATTTCAAAGGACAGCTGG	Yes	Yes	No	0	0	0	No	
NM_hsa_981	chr2:230195439-230195491	-	TGCTGTCCTTTGAAATAAATA	Yes	No	No	0	0	0	No	
NM_hsa_982	chr2:29352285-29352362	-	GCTATAAGATCTCTGTGTCTGT	Yes	Yes	Yes	0	0	0	No	
NM_hsa_982	chr2:29352279-29352367	-	GCTATAAGATCTCTGTGTCTGT	Yes	Yes	Yes	0	0	0	No	
NM_hsa_982	chr2:29352294-29352352	-	GCTATAAGATCTCTGTGTCTGT	Yes	Yes	Yes	0	0	0	No	
NM_hsa_982	chr2:29352297-29352355	+	GCTATAAGATCTCTGTGTCTGT	Yes	Yes	Yes	0	0	0	No	
NM_hsa_982	chr2:29352272-29352380	+	GCTATAAGATCTCTGTGTCTGT	Yes	Yes	Yes	0	0	0	No	
NM_hsa_983	chr2:29352297-29352355	+	AGACACAGAGATCTTATAGCTT	Yes	No	No	0	0	0	No	
NM_hsa_984	chr20:61507159-61507208	+	AGGCTGTAGGTCCGAGAGC	Yes	No	No	0	0.26489	0.0732862	No	
NM_hsa_985	chr20:61507159-61507208	+	GCTCTGACTGGAAGCCCCA	Yes	No	No	0	0	0	No	
NM_hsa_986	chr17:76136837-76136896	+	GAAGCAGCGCCTGTCGCAACTCG	Yes	Yes	No	0	0	0	Yes	
NM_hsa_986	chr17:76136812-76136905	+	GAAGCAGCGCCTGTCGCAACTCG	Yes	Yes	No	0	0	0	Yes	
NM_hsa_987	chr17:76136837-76136896	+	AGTTGCTGCCGCTGCTGTCCT	Yes	No	No	0	0	0	No	
NM_hsa_988	chr2:11484926-11484986	+	CTCTGAGGCGAATAGCCGGG	Yes	No	No	0	0	0	No	
NM_hsa_989	chr2:11484926-11484986	+	CCTTCCTCGCCCGCCCTC	Yes	No	No	0	0	0	No	
NM_hsa_99	chr22:20136497-20136550	+	GGCAGGGATCCCTGGCTTTGGGA	Yes	No	No	0	0	0	No	
NM_hsa_990	chr15:40632542-40632608	+	CTCTGGCAGCGGAACCTG	Yes	No	No	0	0	0	No	
NM_hsa_991	chr15:40632542-40632608	+	GGTCGAGGGGGGAGCT	Yes	No	No	0	0	0	No	
NM_hsa_992	chr12:679670-679762	-	CTCTGAGCGGGAGCGGGGGC	Yes	Yes	No	0	0	0	No	
NM_hsa_992	chr12:679679-679743	-	CTCTGAGCGGGAGCGGGGGC	Yes	Yes	No	0	0	0	No	
NM_hsa_993	chr12:679679-679743	-	CTCTGTTCCCGACCCGCCTGGG	Yes	No	No	0	0	0	No	
NM_hsa_994	chr1:160092522-160092607	+	TAGCCCAGGGTTCAAGGATGCAG	Yes	Yes	No	0	0	0	No	
NM_hsa_997	chr11:63606382-63606441	+	CGCTTCGTGGATTTCCGTA	Yes	No	No	0	0	0	No	
NM_hsa_998	chrX:151128103-151128182	+	TTCTTTGCAGATGAGACTGAGA	Yes	Yes	Yes	0	0	0	No	
NM_hsa_998	chrX:151128078-151128206	+	TTCTTTGCAGATGAGACTGAGA	Yes	Yes	Yes	0	0	0	No	
NM_hsa_998	chrX:151128113-151128174	+	TTCTTTGCAGATGAGACTGAGA	Yes	Yes	Yes	0	0	0	No	
NM_hsa_999	chrX:151128113-151128174	+	TCAGTTTCCTCTGCAAACAGTTGT	Yes	No	No	0	0	0	No	
NM_hsa_2173	chr10:181364-181443	+	AGAGGCGCCGGGCGGCGGGAA	No	Yes	No	0.0569857	0	0	Yes	
NM_hsa_2174	chr10:1095187-1095260	+	TCAGACGTCTCGAGGCTCGCG	No	Yes	No	0	0	0	Yes	
NM_hsa_2177	chr10:6019424-6019513	+	CGCGCGCCCGCCGCGGGGCC	No	Yes	No	0	0	0	No	
NM_hsa_2181	chr10:28592226-28592295	+	CAGGTGGCTGCAGACTGGAG	No	Yes	No	0	0	0	No	
NM_hsa_2182	chr10:29333102-29333178	+	TGGAGTGTGACAATGGTGTTAT	No	Yes	No	0.313421	0.0331113	0	Yes	KapB+cMyc>KapB>H
NM_hsa_2183	chr10:34905155-34905231	+	AACCCAGGAGGTGGACTTTGCAGT	No	Yes	No	0	0	0	No	
NM_hsa_2185	chr10:35929773-35929871	+	GCCGGGCGGCGGCGGCGGCAGGC	No	Yes	No	0.374742	0.268832	0.0875969	Yes	KapB+cMyc>KapB>H
NM_hsa_2186	chr10:38265718-38265789	+	CTGCACGTCTGGGCTTGAAGA	No	Yes	No	0	0	0	No	
NM_hsa_2187	chr10:38383744-38383829	+	CAGGCGCGGACTCCACACCCAG	No	Yes	No	0.0284929	0.0662225	0	No	
NM_hsa_2188	chr10:43248825-43248891	+	CTAGTATATCTCTGAAGCCTC	No	Yes	No	0	0	0	No	
NM_hsa_2189	chr10:49715996-49716074	+	TTGCCTGAAGCCCTGTAGCT	No	Yes	No	0	0	0	No	
NM_hsa_2190	chr10:50156128-50156225	+	CCTGTCTTCCATTACTTGGCG	No	Yes	No	0	0	0	No	
NM_hsa_2192	chr10:54715277-54715355	+	GGATCTCGCTGGGGCCTCCA	No	Yes	No	418.446	317.173	301.28	Yes	KapB+cMyc>KapB>H
NM_hsa_2193	chr10:60184162-60184240	+	TATGTTGTTTCATGTTGGTGG	No	Yes	No	0	0	0	No	
NM_hsa_2194	chr10:65390384-65390471	+	CTCTGTTCCAGCGCTGAGACG	No	Yes	No	0	0	0	No	
NM_hsa_2195	chr10:71992394-71992483	+	ATTCCCAGCATCGGCGCTGAG	No	Yes	No	0	0	0	No	
NM_hsa_2198	chr10:73341619-73341700	+	ACTGAACTTGGAGTCAGAAA	No	Yes	No	0.283374	0.464059	0.202092	Yes	
NM_hsa_2199	chr10:73550492-73550569	+	ACTTTCCTGTCCTCCAACCAGA	No	Yes	No	0	0	0	Yes	
NM_hsa_2200	chr10:73550492-73550569	+	CTGGTCCTGGACAGGAGGCCG	No	Yes	Yes	0	0	0	No	
NM_hsa_2200	chr10:73550468-73550594	+	CTGGTCCTGGACAGGAGGCCG	No	Yes	Yes	0	0	0	No	
NM_hsa_2202	chr10:74033836-74033927	+	TAGAGCTCGCGGTCTGGTCTG	No	Yes	No	0	0	0	No	
NM_hsa_2206	chr10:77162613-77162690	+	CGCCTGCCCTCTCGGTGTCGGA	No	Yes	No	0.740814	0.26489	0.219859	Yes	KapB+cMyc>KapB>H
NM_hsa_2207	chr10:79829482-79829576	+	TGAAGGCTCTGAGCCGGGAGA	No	Yes	No	0	0	0	Yes	
NM_hsa_2208	chr10:88809997-88810076	+	GGAAGTTTCTCTGAACGTGTAA	No	Yes	No	0.284929	0.447002	0.458039	Yes	H>KapB>KapB+cMyc
NM_hsa_2209	chr10:89790373-89790439	+	TGAGTACTGGTAGAATTCAC	No	Yes	No	0	0	0	No	
NM_hsa_2210	chr10:91404849-91404930	+	CACGGCGCCGGCCTGGAGCA	No	Yes	No	0	0	0	No	
NM_hsa_2212	chr10:102422359-102422441	+	TATGAGGTCCAGCTCTGAGC	No	Yes	No	0	0	0	No	
NM_hsa_2220	chr10:105001705-105001800	+	TGAAGTTGGACTCCAAGAACCG	No	Yes	No	0	0	0	No	
NM_hsa_2221	chr10:107025231-107025310	+	GTGGATTGTAGAATTCCAGGT	No	Yes	No	0	0	0	Yes	
NM_hsa_2222	chr10:112573150-112573238	+	CATTCTGTCCTCCCTCCTGCCA	No	Yes	No	0	0	0.0244288	No	
NM_hsa_2225	chr10:114583697-114583778	+	AACAGGTCCAGGAGTAGATT	No	Yes	No	0	0.132445	0	No	
NM_hsa_2226	chr10:115803626-115803713	+	GAAGTCCCGGCGGCTCTTCCAG	No	Yes	No	0.0569857	0	0	No	
NM_hsa_2227	chr10:118003185-118003274	+	CTGTCCTGAGAACCACTGTGA	No	Yes	No	0	0	0	No	
NM_hsa_2228	chr10:118073348-118073437	+	CATTTGATTGTGGCTCCTGGA	No	Yes	No	0	0	0	No	
NM_hsa_2229	chr10:120514777-120514852	+	CTCGCCGGTTCCTGAGGACGCG	No	Yes	No	0	0	0	No	
NM_hsa_2230	chr10:120706677-120706754	+	CTTGTTCCCCATTACTTGGCTCA	No	Yes	No	0	0	0	Yes	
NM_hsa_2231	chr10:121025558-121025636	+	CTCCCCCAGAAGGCTGTCCTGA	No	Yes	Yes	0.0569857	0	0	No	
NM_hsa_2231	chr10:121025528-121025666	+	CTCCCCCAGAAGGCTGTCCTGA	No	Yes	Yes	0.0569857	0	0	No	
NM_hsa_2232	chr10:121086991-121087084	+	CACTGAAGGCTGGGCACGGG	No	Yes	No	0	0	0	Yes	
NM_hsa_2234	chr10:124110351-124110428	+	TCTGGCCCTGTGTCTTCACTCT	No	Yes	No	0	0	0	Yes	
NM_hsa_2237	chr10:127730698-127730767	+	AGTGTCAGTGAGGCAGTAGA	No	Yes	No	0	0	0	No	
NM_hsa_2239	chr10:3526053-3526143	-	CGGAGTGGGTCGGGAGGAGA	No	Yes	No	0	0	0	No	
NM_hsa_2240	chr10:6370152-6370243	-	ATTGCCATTTCTGAGGCAGAGC	No	Yes	No	0.0569857	0	0	No	
NM_hsa_2241	chr10:15031579-15031650	-	AAGAGTATCACAGAGCTGTGGA	No	Yes	No	0	0	0	No	
NM_hsa_2243	chr10:50377361-50377454	-	AGGGTTGGGTGGAGGATGTCT	No	Yes	No	0	0	0.109929	Yes	
NM_hsa_2244	chr10:68805357-68805424	-	GTGCGGAGTGCCCCTAGTCCTG	No	Yes	No	0	0	0	No	
NM_hsa_2246	chr10:74480786-74480859	-	GAGCCAGTGGTGAAACAGTGAG	No	Yes	No	0	0	0	Yes	
NM_hsa_2247	chr10:75615772-75615852	-	TTATCAGAATCTGATGGGGT	No	Yes	No	0	0	0	No	
NM_hsa_2248	chr10:75634372-75634462	-	CCCCTCCCCGGGGGCGGGCC	No	Yes	No	0	0	0	No	
NM_hsa_2249	chr10:76852045-76852139	-	GGTGCAGTGCTTCATGTCTGT	No	Yes	No	0	0	0	No	
NM_hsa_2250	chr10:81330834-81330907	-	TCGTCTCAGAGGGCACCTGGCT	No	Yes	No	0.0854786	0.198668	0.109929	Yes	
NM_hsa_2251	chr10:91174666-91174750	-	CGCCGCGCGCCGGGACAGAG	No	Yes	No	0.313421	0.198668	0.293145	No	
NM_hsa_2256	chr10:97849917-97850003	-	CTGCTGTCAGTGAGGGCCCG	No	Yes	No	0.113971	0	0	No	
NM_hsa_2260	chr10:101295022-101295110	-	CGGCGGCGGCGGGGGCGCGTG	No	Yes	No	0.00712322	0.00413891	0.082447	Yes	
NM_hsa_2261	chr10:102286581-102286680	-	GTCCAGTGATGTGAGATCTGAGA	No	Yes	No	2.33641	3.11246	3.66431	No	H>KapB>KapB+cMyc
NM_hsa_2262	chr10:102550400-102550484	-	TCTCCAAACCCTTTTTCCAG	No	Yes	No	0	0	0	No	
NM_hsa_2265	chr10:120819518-120819594	-	TGCTGCCAGTCTCCTTCAGA	No	Yes	No	0	0	0	Yes	
NM_hsa_2266	chr10:121781390-121781472	-	AATGGATTTTTGGTACTTGG	No	Yes	No	0	0	0	Yes	

Additional file 5

Name	locus	strand	sequence	mirdeep2	mireap	miranalyzer	RPM_HU_KapB_cMyc	RPM_HU_KaposinB	RPM_HUVEC	IP_confidence	annotation
NM_hsa_2267	chr10:123790178-123790250	-	CTGGGTCATGTCTGGCTGAG	No	Yes	No	0	0	0	No	
NM_hsa_2268	chr10:125818975-125819064	-	CAGGTCAAAGTGGGCAAGGGG	No	Yes	No	0	0	0	No	
NM_hsa_2270	chr10:130735313-130735398	-	TTGGACTGAGCCGTGCTACATA	No	Yes	No	0	0	0	Yes	
NM_hsa_2273	chr11:404451-404543	+	TTGGGCCTCTCTCCACTGTAG	No	Yes	No	0	0	0	No	
NM_hsa_2274	chr11:448252-448349	+	CTCTGCACCCTGGAGGGACGG	No	Yes	No	0	0	0	No	
NM_hsa_2276	chr11:610406-610493	+	CTGTAGGGCCCAGTGCTCAGC	No	Yes	No	0.113971	0	0	Yes	
NM_hsa_2277	chr11:777577-777655	+	CGGTCCGGGTCGGCTGGGCTGC	No	Yes	Yes	0	0	0	No	
NM_hsa_2277	chr11:777535-777618	+	CGGTCCGGGTCGGCTGGGCTGC	No	Yes	Yes	0	0	0	No	
NM_hsa_2278	chr11:833261-833369	+	GGCGCCTCCCGGGCCCTTGGAC	No	Yes	Yes	0	0	0	No	
NM_hsa_2278	chr11:833276-833363	+	GGCGCCTCCCGGGCCCTTGGAC	No	Yes	Yes	0	0	0	No	
NM_hsa_2281	chr11:3862949-3863087	+	TGCGCTCGGAGCTTTGACTTGA	No	Yes	Yes	0	0.0662225	0.146572	No	H>KapB>KapB+cMyc
NM_hsa_2281	chr11:3862979-3863062	+	TGCGCTCGGAGCTTTGACTTGA	No	Yes	Yes	0	0.0662225	0.146572	No	H>KapB>KapB+cMyc
NM_hsa_2285	chr11:11642548-11642640	+	ACTCTGAGCCGGACTTGTGTGC	No	Yes	No	0	0	0	No	
NM_hsa_2287	chr11:16968511-16968609	+	TGGGTCATCTGGGCTGCTGGTT	No	Yes	No	0	0	0	No	
NM_hsa_2288	chr11:18287930-18288023	+	AAGCAGGGTCGGGCCTGGTAG	No	Yes	No	0.968757	0.463558	1.09929	Yes	
NM_hsa_2290	chr11:20792492-20792569	+	TTTCTGGAAAGACCCCTCTAGC	No	Yes	No	0	0	0	No	
NM_hsa_2291	chr11:22850970-22851058	+	GTCAGTGGAAGGTAGAAAGA	No	Yes	No	0	0	0	No	
NM_hsa_2292	chr11:25410245-25410322	+	CACTAGATCATGAGCTCCTGCAG	No	Yes	No	0.0284929	0	0	Yes	
NM_hsa_2294	chr11:36175509-36175595	+	TTCATCCCAGACTGAAGGCCAGA	No	Yes	Yes	0.113971	0	0.0732862	No	
NM_hsa_2294	chr11:36175505-36175605	+	TTCATCCCAGACTGAAGGCCAGA	No	Yes	Yes	0.113971	0	0.0732862	No	
NM_hsa_2296	chr11:43600200-43600288	+	GTCGGGGCGGGAGTGGTGGT	No	Yes	No	0	0	0	Yes	
NM_hsa_2298	chr11:45006552-45006648	+	CCAGCTTTCCCTTCCCCCGC	No	Yes	No	0	0	0	No	
NM_hsa_2299	chr11:46182255-46182351	+	AGGGGAGAAGCACTGTAGCT	No	Yes	No	0	0	0	No	
NM_hsa_2300	chr11:46397990-46398074	+	CCTGATCCTCAGCTGCCCTCT	No	Yes	No	0.0569857	0	0	No	
NM_hsa_2302	chr11:47235998-47236077	+	CTCCCCGGACCCGCAGAGGC	No	Yes	No	0	0	0	No	
NM_hsa_2303	chr11:54750504-54750590	+	CTAGAAGGAAGCTCTTTGAGA	No	Yes	No	0	0	0	No	
NM_hsa_2306	chr11:58940659-58940731	+	GCCGGCACCTTGGCTGACCTGA	No	Yes	No	0	0	0	No	
NM_hsa_2307	chr11:58956798-58956893	+	TTGTCTCGTGGGGTACTGAG	No	Yes	No	0	0	0	No	
NM_hsa_2308	chr11:60508603-60508699	+	TGAGGTAAGCATTGGACTCT	No	Yes	No	0.284929	0.927115	1.24587	No	H>KapB>KapB+cMyc
NM_hsa_2309	chr11:61520105-61520190	+	GAGCCCAGCCGGGACTGTCGC	No	Yes	No	0	0	0	No	
NM_hsa_2312	chr11:62761657-62761748	+	TGATGGAGTAGGTTGTGGGG	No	Yes	No	13.0402	9.00626	1.79551	No	KapB+cMyc>KapB>H
NM_hsa_2314	chr11:63536105-63536195	+	GCCCCGGGGCGGCGGGGCGC	No	Yes	No	0	0	0	No	
NM_hsa_2315	chr11:64018944-64019014	+	CTCTGGTCGGTCCGGGACAGA	No	Yes	No	0	0	0	No	
NM_hsa_2316	chr11:64031423-64031502	+	TGGCCTTCTGCCTCCCCCCAG	No	Yes	No	0	0	0	Yes	
NM_hsa_2317	chr11:64072814-64072905	+	GGCTCGGCGGGCTGCGGGGC	No	Yes	No	0	0	0	No	
NM_hsa_2319	chr11:64128534-64128615	+	GCACCCCTCTTGCTCCTACCAG	No	Yes	No	0	0	0	No	
NM_hsa_2323	chr11:65167989-65168061	+	GGCTCTGATGAAAGCTGAGG	No	Yes	No	0	0	0	No	
NM_hsa_2324	chr11:65202608-65202686	+	AATGGATGGGGCTGTGGAGT	No	Yes	No	0	0	0	No	
NM_hsa_2325	chr11:65271877-65271949	+	AATGCATTGTGAAACGACTGGAG	No	Yes	No	0.0569857	0	0	Yes	
NM_hsa_2326	chr11:65430748-65430823	+	AGGCCGGGTGGTTGAACTCCG	No	Yes	No	0.170957	0.132445	0	No	KapB+cMyc>KapB>H
NM_hsa_2328	chr11:65659226-65659300	+	CCCATCGGGCGCCGGGACCGG	No	Yes	No	0	0	0	No	
NM_hsa_2329	chr11:65744041-65744116	+	CTGGCTGGGGCCTGGGCCGAC	No	Yes	No	0	0	0	Yes	
NM_hsa_2330	chr11:66115682-66115769	+	GGGGGGGAAGCATCTACATTTT	No	Yes	No	0	0	0	No	
NM_hsa_2331	chr11:66138873-66138953	+	GTCGGCTGATGGGAATTGTAGT	No	Yes	No	0	0	0	Yes	
NM_hsa_2332	chr11:66360675-66360763	+	TCCTGCGCCGGAGGAGTTCTG	No	Yes	No	0	0	0	No	
NM_hsa_2334	chr11:67397703-67397771	+	GGACGGAGGGCGGCGGGGCGG	No	Yes	No	0.212747	0.101541	0.0671791	No	KapB+cMyc>KapB>H
NM_hsa_2336	chr11:68608479-68608572	+	CTCACGGCAGCGCTCGGACC	No	Yes	No	0	0.0662225	0.0732862	No	H>KapB>KapB+cMyc
NM_hsa_2339	chr11:75986937-75987008	+	CGGGGTGGGTGAGGTGAGGGA	No	Yes	No	0	0	0	No	
NM_hsa_2340	chr11:76850623-76850705	+	CATTCTCACCTCCCTCCTGCCT	No	Yes	No	0	0	0.0244288	No	
NM_hsa_2341	chr11:77706131-77706222	+	GTAGGTTTCCTGAGCACTGC	No	Yes	No	0	0	0	No	
NM_hsa_2342	chr11:79113064-79113157	+	CTCTAGAAGCTCACAGTCTAGT	No	Yes	No	4.39285	14.1363	8.83309	Yes	
NM_hsa_2344	chr11:93063783-93063876	+	TTGGTCGCGCGGCGGGAGGGCC	No	Yes	No	0	0	0	No	
NM_hsa_2346	chr11:100041472-100041544	+	TCTGAGACTGATCTTGATGA	No	Yes	No	0	0	0	No	
NM_hsa_2347	chr11:102216848-102216929	+	CGGATTCAACGGGCAAAGAG	No	Yes	No	0	0	0	No	
NM_hsa_2348	chr11:104131597-104131694	+	TAAGTAGATTCATGTTGTTGT	No	Yes	No	0	0	0	Yes	
NM_hsa_2349	chr11:108338391-108338474	+	TTCGATTGTCAGTGGCTTTGA	No	Yes	No	0	0	0	No	
NM_hsa_2350	chr11:110902182-110902276	+	GTGATTGGATCTTGGGAGCA	No	Yes	No	0	0	0	No	
NM_hsa_2355	chr11:112097373-112097465	+	CGGCTTCGTGGGGCTTCGGA	No	Yes	No	0	0	0	No	
NM_hsa_2356	chr11:113390477-113390549	+	TGCTGTAGGAGGTTGTAAAG	No	Yes	No	1.39615	0.761559	0.329788	No	KapB+cMyc>KapB>H
NM_hsa_2362	chr11:124837914-124837988	+	TCGTCTCAGAGGGCACCTGGCTGT	No	Yes	No	0.940265	0.79467	0.696219	Yes	KapB+cMyc>KapB>H
NM_hsa_2363	chr11:129149583-129149657	+	CTGCTGTTGTCCCGGGAGCG	No	Yes	No	0	0	0	No	
NM_hsa_2364	chr11:134131135-134131217	+	AACGTCGGCGCTCTTCCCCTCT	No	Yes	Yes	0.284929	0	0	Yes	
NM_hsa_2364	chr11:134131083-134131191	+	AACGTCGGCGCTCTTCCCCTCT	No	Yes	Yes	0.284929	0	0	Yes	
NM_hsa_2366	chr11:1780805-1780899	-	CGGACCCTGTCCCCTGCCAG	No	Yes	No	0	0	0	No	
NM_hsa_2370	chr11:11005776-11005875	-	ACCAGATTGTGAGCTCCTTGAGG	No	Yes	No	0.296326	0.200875	0.249173	Yes	
NM_hsa_2371	chr11:14521045-14521144	-	GCGGGTCGGGGTGGGGGCGGAGG	No	Yes	No	0	0	0	No	
NM_hsa_2372	chr11:17298021-17298107	-	CTGAGCCAGGAGGGACGTGG	No	Yes	No	0	0	0	No	
NM_hsa_2373	chr11:18342206-18342292	-	AGGTTGACTGGAACAGAGGGTTGG	No	Yes	No	0	0	0	No	
NM_hsa_2375	chr11:21022414-21022489	-	ACCGGGTGCTGTAGGCTGTC	No	Yes	No	77.4151	50.6657	31.8001	Yes	KapB+cMyc>KapB>H
NM_hsa_2376	chr11:22851226-22851290	-	CCCACGCCGGACCTGGTGAG	No	Yes	No	0	0	0	No	
NM_hsa_2377	chr11:26611102-26611170	-	AGGGACTTTTGGGGGAAAATA	No	Yes	No	0	0	0	Yes	
NM_hsa_2378	chr11:32379135-32379214	-	CTGGACTTGGGGTCAGAAGAC	No	Yes	No	0.0241758	0.00551854	0.00916078	Yes	
NM_hsa_2379	chr11:33902810-33902896	-	TTTCCAGATGGAGGGCCACG	No	Yes	No	0	0	0	No	
NM_hsa_2380	chr11:39089310-39089382	-	CCACTGCCCCAGGTGATGCCA	No	Yes	No	1.03524	0.982301	1.05044	Yes	
NM_hsa_2381	chr11:47170429-47170498	-	TCCCTGGTCTAGTGGCACGG	No	Yes	No	0	0.132445	0	No	
NM_hsa_2382	chr11:47429705-47429775	-	GGGCTGGGAGGACTACTGTG	No	Yes	No	0	0	0	No	
NM_hsa_2383	chr11:49504384-49504470	-	TGAGGTATGAGGTTTTATATTT	No	Yes	No	0	0	0	No	
NM_hsa_2386	chr11:64109305-64109392	-	TTGTGGTCTGGGGTCTTGGGC	No	Yes	Yes	0	0	0	Yes	
NM_hsa_2386	chr11:64109301-64109407	-	TTGTGGTCTGGGGTCTTGGGC	No	Yes	Yes	0	0	0	Yes	
NM_hsa_2387	chr11:64109305-64109392	-	CCCAAGACCTCAGACCACAACC	No	Yes	No	0	0	0	Yes	
NM_hsa_2388	chr11:64359146-64359223	-	CTGGCCTGAGCCGTGCTGTT	No	Yes	No	0	0	0	No	
NM_hsa_2389	chr11:67140040-67140112	-	GCCCCCGCCCCCGCCGCGGGG	No	Yes	No	0.0284929	0	0	No	
NM_hsa_2390	chr11:68079986-68080063	-	CGTCTCCGCCCCCCGCCGCCG	No	Yes	No	0.170957	0.26489	0.439717	No	H>KapB>KapB+cMyc
NM_hsa_2391	chr11:70592483-70592581	-	CACCCTGCATCCCCCACCCTGA	No	Yes	No	0	0	0	No	
NM_hsa_2393	chr11:76388047-76388140	-	GCAGGATGTATGTAACCATGTG	No	Yes	No	0	0	0	No	
NM_hsa_2396	chr11:86488902-86488980	-	CAAATGAGAACTTTGCAGAA	No	Yes	No	0	0	0	Yes	
NM_hsa_2397	chr11:86667052-86667125	-	TGGGGGCGGGGAGAGGCCGGG	No	Yes	No	0	0	0	Yes	
NM_hsa_2398	chr11:91656157-91656241	-	TTTCAGTCTGATGTCTGCTGT	No	Yes	No	0	0	0	No	
NM_hsa_2399	chr11:97593310-97593396	-	TGGTAGTAGATTGTTTAGTG	No	Yes	No	0.256436	0.110371	0.0610719	Yes	KapB+cMyc>KapB>H
NM_hsa_2400	chr11:100120361-100120447	-	TCTCAGTGATGACAAATGTG	No	Yes	No	0	0	0	No	
NM_hsa_2402	chr11:116643806-116643882	-	GAGCGCGTCCCGGTGGCCGCA	No	Yes	No	0	0	0	No	
NM_hsa_2403	chr11:118120436-118120518	-	TGGTAGACTAGGGGACCTAGG	No	Yes	No	0	0	0	No	
NM_hsa_2409	chr11:125797773-125797865	-	AGCTTCTCAGACTCATGTTGC	No	Yes	No	0	0	0	Yes	
NM_hsa_2410	chr11:125932755-125932822	-	CAGCCGCGGGGGCGCGCCCC	No	Yes	No	0.0569857	0.132445	0	No	
NM_hsa_2411	chr11:133306823-133306907	-	GTCGGCACATTACCCATCTGACA	No	Yes	No	0.0284929	0.0331113	0.0366431	Yes	H>KapB>KapB+cMyc
NM_hsa_2413	chr12:3068102-3068181	+	GGACGCCGGGTGGCGGGGCGG	No	Yes	No	0	0	0	No	
NM_hsa_2417	chr12:7073201-7073285	+	CTGTCGGCCGGCCCTGGGTC	No	Yes	No	0	0	0	No	
NM_hsa_2419	chr12:9216969-9217054	+	GTGGCTGCTGTGGGACATCTG	No	Yes	No	0	0	0	No	
NM_hsa_2420	chr12:12715716-12715803	+	TTCCCTTCCAGAGTCCGGCGA	No	Yes	No	0.113971	0.132445	0.0732862	Yes	
NM_hsa_2421	chr12:13066595-13066661	+	CATTAGACTCTGGGGGATAGA	No	Yes	No	0	0	0	No	
NM_hsa_2423	chr12:25404197-25404288	+	CGCGGCGGGCTGTGCGGATG	No	Yes	No	0	0	0	No	
NM_hsa_2424	chr12:27840862-27840955	+	GAGGATCCGTCTGAGCCTGG	No	Yes	No	0	0	0	No	
NM_hsa_2425	chr12:28291420-28291507	+	ATCTTCTGGGTTGCGGACTGC	No	Yes	No	0	0	0	No	
NM_hsa_2426	chr12:28487173-28487239	+	GGAGTTAGGAGTTTGTATAGTT	No	Yes	No	0.0256436	0.079467	0.0513004	Yes	
NM_hsa_2428	chr12:49719458-49719539	+	CAGCCTCTGTTGGTGTCCCAG	No	Yes	No	0	0	0	Yes	
NM_hsa_2429	chr12:49752850-49752930	+	CATGTTAGAACTTTGAACGCAC	No	Yes	No	0	0	0	No	
NM_hsa_2431	chr12:50222402-50222493	+	GTAGAGGACGCCGGGCTGGG	No	Yes	No	0.113971	0	0	No	
NM_hsa_2432	chr12:51663552-51663633	+	CTTCTGGCTCGCCTCGGACG	No	Yes	No	0	0	0	No	
NM_hsa_2437	chr12:54427665-54427760	+	GCGACTCGGGAGGGGCGGGAG	No	Yes	No	0	0	0	No	
NM_hsa_2443	chr12:62997439-62997507	+	TCCCCGACACCATGGCCCTGGC	No	Yes	No	0.968757	0.26489	0.58629	No	
NM_hsa_2445	chr12:64237870-64237936	+	CGCGGCGCCGGCTCTGGAGA	No	Yes	No	0	0	0	No	
NM_hsa_2446	chr12:68588268-68588350	+	TTTTGAGATGTCTTACATGGA	No	Yes	No	0	0	0	Yes	
NM_hsa_2449	chr12:76353137-76353217	+	AGTCTGCCAGAATTTGGGCTTT	No	Yes	No	0	0	0	No	
NM_hsa_2451	chr12:83277795-83277869	+	CTAAACAAGATTACTCTGAAG	No	Yes	No	0	0	0	No	
NM_hsa_2452	chr12:91307091-91307171	+	AGATTTAGGTTGGGACACAA	No	Yes	No	0	0.198668	0	No	
NM_hsa_2454	chr12:96183537-96183615	+	CACCAGGTCAGGGACACCAGCGA	No	Yes	No	0	0	0.0732862	No	
NM_hsa_2458	chr12:98931166-98931262	+	TTTCTCCAATGTTATTTCCAG	No	Yes	No	0	0	0	No	
NM_hsa_2460	chr12:104226131-104226225	+	GAATCAGAATCTCCAGGGTTAG	No	Yes	No	0	0	0	No	
NM_hsa_2464	chr12:105061639-105061727	+	GAATCAGAATCTCTAGGGGTA	No	Yes	No	0	0	0	No	
NM_hsa_2466	chr12:105672281-105672357	+	ACTTGGCTTTTTGTTCCCCTGC	No	Yes	No	0	0	0	No	
NM_hsa_2467	chr12:109022384-109022474	+	GAGTCAGGGCCGGACTTGCAG	No	Yes	No	0	0	0	No	
NM_hsa_2468	chr12:109747808-109747901	+	CCCTTCCCCGCCGCCCGGCCC	No	Yes	No	0	0	0	No	
NM_hsa_2470	chr12:112354552-112354633	+	AGATGTAGTAGTTTGTGCTTT	No	Yes	No	178.804	162.278	72.678	Yes	KapB+cMyc>KapB>H
NM_hsa_2474	chr12:114318856-114318929	+	CCTTACACACGCAAGGCTTGC	No	Yes	No	0	0	0	No	
NM_hsa_2476	chr12:120799470-120799561	+	GGCCCCCCGCCGCGGGGCCC	No	Yes	No	0	0	0	No	
NM_hsa_2478	chr12:121124657-121124729	+	CAGCTGTGGGATTCCGGGGC	No	Yes	No	0	0	0	No	
NM_hsa_2480	chr12:122657141-122657214	+	TTTGTTGTCTAGCTGTCTGA	No	Yes	No	0	0	0	No	
NM_hsa_2481	chr12:123340441-123340536	+	CACGTCACACCTCCTCCCGCAG	No	Yes	No	0.113971	0	0.146572	Yes	
NM_hsa_2482	chr12:123756348-123756444	+	GCGGGGCGGCCGGGGCCGGG	No	Yes	No	0	0.0331113	0.0732862	Yes	H>KapB>KapB+cMyc
NM_hsa_2484	chr12:124945972-124946053	+	GCAGAGACTCTGGGTCTGCGTG	No	Yes	No	0	0	0	No	
NM_hsa_2486	chr12:131611924-131612017	+	AGTGATGATGAGACCTCTGAGC	No	Yes	No	0	0	0	No	
NM_hsa_2487	chr12:132579001-132579079	+	TGCTTGAGAGGGGCTGACTGTGG	No	Yes	No	0	0	0	No	
NM_hsa_2488	chr12:133140648-133140724	+	CTCCTGTGATGTTTCTGAGGC	No	Yes	No	2.50737	3.2449	3.07802	Yes	
NM_hsa_2489	chr12:1907784-1907864	-	ACTGGACTTGGAGTCCCAGGC	No	Yes	No	0	0	0	Yes	
NM_hsa_2490	chr12:4541745-4541820	-	TCCTCTGCCCTGCAAATGTCA	No	Yes	No	0	0	0	No	
NM_hsa_2492	chr12:29387550-29387639	-	AGAACTTTGAGGAGGGAAGT	No	Yes	No	0	0	0	No	
NM_hsa_2493	chr12:33793708-33793806	-	TGAAAACATCCTTGAAAGGAAG	No	Yes	No	0	0	0	No	
NM_hsa_2494	chr12:41387687-41387767	-	CACTCTGGACTCTGAATACTT	No	Yes	No	72.03	63.6067	81.5309	Yes	
NM_hsa_2495	chr12:52772192-52772280	-	CTGACCCCACTCCCTCCCCACC	No	Yes	No	0	0	0	No	
NM_hsa_2497	chr12:56109296-56109412	-	TGGCGGGCTGTGTGAATGTAG	No	Yes	Yes	0	0	0	No	
NM_hsa_2497	chr12:56109316-56109411	-	TGGCGGGCTGTGTGAATGTAG	No	Yes	Yes	0	0	0	No	
NM_hsa_2498	chr12:65563151-65563220	-	CTCTCACCTTGAGGTGTCGG	No	Yes	No	0	0	0	No	
NM_hsa_2499	chr12:71003209-71003296	-	CCACCTCCCCTGCAAGCCTCT	No	Yes	No	0	0	0	No	
NM_hsa_2500	chr12:102717963-102718046	-	ATTGGTGATGGGGATGGGAG	No	Yes	No	0	0	0	No	
NM_hsa_2501	chr12:104142572-104142670	-	TGCCCTGTGGCCTCTGTGCTGG	No	Yes	No	0	0	0	No	
NM_hsa_2503	chr12:116756412-116756488	-	CTAACCAGTCAGTAGGAAGG	No	Yes	No	0	0	0	No	
NM_hsa_2505	chr12:121837641-121837706	-	ACCTGCGGACCTGGGCGTGG	No	Yes	No	0	0	0	No	
NM_hsa_2506	chr12:127650709-127650800	-	TCGGTTGGCCTCGGATGGCCTC	No	Yes	No	3.81804	3.70846	0.806149	Yes	KapB+cMyc>KapB>H
NM_hsa_2508	chr13:21277814-21277903	+	GCTCGGGGCGGCGGCGGCGG	No	Yes	No	0.256436	0.307462	0.205899	No	
NM_hsa_2509	chr13:22427384-22427457	+	TTTCTTTGGAGTGAAGTTCAGT	No	Yes	No	0	0	0	No	
NM_hsa_2511	chr13:25085448-25085531	+	ATGAGATGTGGCGGACACTGG	No	Yes	No	0	0	0	No	
NM_hsa_2512	chr13:31039108-31039192	+	CGCACGGAGAACGCGGGGCTGT	No	Yes	No	0.170957	0.132445	0	No	KapB+cMyc>KapB>H
NM_hsa_2513	chr13:33161256-33161339	+	CCCCCGCCGCCGGGTCCCCGCGC	No	Yes	Yes	0.0569857	0	0	No	
NM_hsa_2513	chr13:33161244-33161354	+	CCCCCGCCGCCGGGTCCCCGCGC	No	Yes	Yes	0.0569857	0	0	No	
NM_hsa_2514	chr13:34530769-34530838	+	CTCTTCCCACTCTTAGCTGTG	No	Yes	No	0	0	0	No	
NM_hsa_2515	chr13:40810837-40810911	+	AATGCCCTCGGGCAAGGATTA	No	Yes	No	0	0	0	No	
NM_hsa_2516	chr13:41238962-41239045	+	CCCGCGCGGCCAGGCGCCGG	No	Yes	No	0	0	0	No	
NM_hsa_2519	chr13:53962031-53962127	+	TTCCCACTTGGGGTTGTAGG	No	Yes	No	0	0	0	No	

Additional file 5

Name	locus	strand	sequence	mirdeep2	mireap	miranalyzer	RPM_HU_KapB_cMyc	RPM_HU_KaposinB	RPM_HUVEC	IP_confidence	annotation
NM_hsa_2520	chr13:77524175-77524261	+	TCAGTGTCCAGGTTGAAGGA	No	Yes	No	0	0	0	No	
NM_hsa_2521	chr13:80596381-80596466	+	GTCTGCAGGGAAGCACTTTAT	No	Yes	No	0	0	0	No	
NM_hsa_2522	chr13:80915894-80915966	+	GGGGCTGGGGCGGGGGGAGAAG	No	Yes	No	0	0	0	No	
NM_hsa_2526	chr13:92003261-92003335	+	ATGTGACAGCTTCTGTAGCAC	No	Yes	No	0.3989	0.860893	1.24587	Yes	H>KapB>KapB+cMyc
NM_hsa_2530	chr13:97079472-97079558	+	TTATGTGACCTCGGATGAAT	No	Yes	No	0	0	0.146572	Yes	
NM_hsa_2531	chr13:99739001-99739088	+	ACGGCGGGCCAGGCAGGTCGG	No	Yes	No	0	0	0	No	
NM_hsa_2532	chr13:113863070-113863148	+	GGAGAACCTGGGGACCGGCT	No	Yes	No	0	0	0	No	
NM_hsa_2533	chr13:114542970-114543058	+	TGCTGGCCTAGGGCTGACAG	No	Yes	No	0	0	0	No	
NM_hsa_2534	chr13:20357031-20357121	-	GGGCGTGAGTTGGGGGAGGA	No	Yes	No	0	0	0	No	
NM_hsa_2535	chr13:20981173-20981259	-	TTTGTAGAAATAAATGTCCCTT	No	Yes	No	0	0	0	No	
NM_hsa_2536	chr13:24012704-24012790	-	CTGCTGTGATGACCATACAA	No	Yes	No	0	0	0	No	
NM_hsa_2538	chr13:28546456-28546521	-	TGCTGCCCTTAGATGTCAGC	No	Yes	No	0	0	0	No	
NM_hsa_2539	chr13:42030048-42030148	-	GTTCGATTCCCGGTCAGGGAACAA	No	Yes	No	13.1067	13.9729	32.6124	Yes	H>KapB>KapB+cMyc
NM_hsa_2540	chr13:50570803-50570878	-	CGGGGAGGGCCGGGCTGCGG	No	Yes	No	0	0	0	No	
NM_hsa_2543	chr13:52554493-52554589	-	CTGTGGCCTCCTCTGCTTCCAGT	No	Yes	No	0	0	0	No	
NM_hsa_2544	chr13:62980438-62980527	-	GAGGAACAGATGGAGTCTAAT	No	Yes	No	0	0	0	No	
NM_hsa_2547	chr13:103046759-103046827	-	GCGCGGGTCGGGGCTAGGGC	No	Yes	No	0	0	0	No	
NM_hsa_2548	chr13:113830028-113830118	-	CCTGTGCCTGTGGAGTTCATG	No	Yes	No	0	0	0	Yes	
NM_hsa_2549	chr14:19285183-19285267	+	AACACAGTGGCTAGGTTCTG	No	Yes	No	0.303924	0.198668	0.268716	Yes	
NM_hsa_2550	chr14:21151502-21151572	+	CTCGAAGGAGACGCTGCTGTTT	No	Yes	No	0.0569857	0	0	Yes	
NM_hsa_2551	chr14:21152187-21152272	+	GCCCTCGTGGCTACTGTTTT	No	Yes	No	0.0569857	0	0.0732862	No	
NM_hsa_2553	chr14:24657549-24657683	+	TTCAGTCGTCTGCGGGTCAG	No	Yes	Yes	0	0	0	No	
NM_hsa_2553	chr14:24657579-24657653	+	TTCAGTCGTCTGCGGGTCAG	No	Yes	Yes	0	0	0	No	
NM_hsa_2554	chr14:24657549-24657683	+	GCGGATCTAGGACGAGGAAG	No	Yes	Yes	0	0	0	No	
NM_hsa_2554	chr14:24657579-24657653	+	GCGGATCTAGGACGAGGAAG	No	Yes	Yes	0	0	0	No	
NM_hsa_2557	chr14:33843882-33843968	+	TCTGCTGATAGACACCTTCTTTA	No	Yes	No	0	0	0	No	
NM_hsa_2558	chr14:34488656-34488728	+	GTGCAAACCAGGCTGGGCGTGG	No	Yes	No	0	0	0	No	
NM_hsa_2559	chr14:52073519-52073599	+	TGCTCAGTCTGAGGCCAAAG	No	Yes	No	0	0	0	No	
NM_hsa_2561	chr14:55823063-55823159	+	CTGATGTTATCTAGCTGTATG	No	Yes	No	0	0	0	Yes	
NM_hsa_2563	chr14:64108824-64108904	+	CTTCCTCGTGGCTGACTGGATG	No	Yes	No	0	0	0	No	
NM_hsa_2564	chr14:65092767-65092905	+	TGGGAGCCGGGAGCCACTGTAG	No	Yes	Yes	0	0	0	No	
NM_hsa_2564	chr14:65092799-65092875	+	TGGGAGCCGGGAGCCACTGTAG	No	Yes	Yes	0	0	0	No	
NM_hsa_2565	chr14:65213845-65213933	+	GTCTGTGGAGTTGCTTGCTGGA	No	Yes	Yes	0	0	0	No	
NM_hsa_2565	chr14:65213811-65213891	+	GTCTGTGGAGTTGCTTGCTGGA	No	Yes	Yes	0	0	0	No	
NM_hsa_2566	chr14:65937572-65937650	+	CTGCACAGAGGGCGGCGGGCTGT	No	Yes	No	0.626843	0.26489	0.146572	Yes	KapB+cMyc>KapB>H
NM_hsa_2567	chr14:65937572-65937650	+	TGTCCGCCTCTCCCTGTGCCGT	No	Yes	Yes	0	0	0.146572	No	
NM_hsa_2567	chr14:65937557-65937665	+	TGTCCGCCTCTCCCTGTGCCGT	No	Yes	Yes	0	0	0.146572	No	
NM_hsa_2570	chr14:72398981-72399071	+	CGGTCCCCGCCCGCCGGCCC	No	Yes	No	0	0	0	No	
NM_hsa_2571	chr14:75593844-75593914	+	CCCGCTTTCGGGGAAGGCCTG	No	Yes	No	0	0	0	No	
NM_hsa_2574	chr14:77494949-77495027	+	ACGCTCGCCCGGAATGTGGG	No	Yes	No	0	0	0	No	
NM_hsa_2577	chr14:93152517-93152611	+	GCCCTGTACTAGCTTGCTGT	No	Yes	No	0	0	0	No	
NM_hsa_2615	chr14:104094651-104094718	+	GCCGCAGGACTTGACCCAGG	No	Yes	No	0.0569857	0	0.0732862	No	
NM_hsa_2617	chr14:104314127-104314211	+	ACCGCGGACGGTGGGAGGACTGA	No	Yes	No	0	0	0	No	
NM_hsa_2619	chr14:105282492-105282580	+	CGCGAGGACGGGCGCTGCCAG	No	Yes	No	0	0	0	No	
NM_hsa_2620	chr14:105559750-105559841	+	GCGCGGGGCGGGGTCGGGGG	No	Yes	No	0	0	0	No	
NM_hsa_2622	chr14:105886342-105886420	+	TACAGGGTCGGAGGTAAGGCCGC	No	Yes	No	0	0.198668	0	Yes	
NM_hsa_2623	chr14:21675672-21675742	-	TATTTAATGTGATCAGCTCAGG	No	Yes	No	0	0	0	No	
NM_hsa_2624	chr14:22791151-22791232	-	TCTGGCTCCGTGTCCTCTCT	No	Yes	No	0	0	0	Yes	
NM_hsa_2626	chr14:34269620-34269698	-	GGCCGCGCCGTCGGCGCCGGG	No	Yes	No	0	0	0	No	
NM_hsa_2627	chr14:35015618-35015690	-	CCCGGACCGGGGACCTACCGA	No	Yes	No	0	0.0662225	0	No	
NM_hsa_2628	chr14:35451629-35451704	-	GCTTCGCACTGTGGCTCCTG	No	Yes	No	0.0569857	0	0.366431	No	
NM_hsa_2629	chr14:48147155-48147253	-	CACTAGATTGTGTGCTTCTTGA	No	Yes	No	0	0	0	Yes	
NM_hsa_2631	chr14:74892786-74892879	-	GGGGCGGGGCGGGCGGCGGCGGCG	No	Yes	No	0.498625	0.463558	0.0732862	Yes	KapB+cMyc>KapB>H
NM_hsa_2633	chr14:77843371-77843459	-	GCAGATGTCTGACCTGCAGG	No	Yes	No	0	0	0	No	
NM_hsa_2634	chr14:93581351-93581415	-	GGACGCGGCGCCGGCGCCCG	No	Yes	No	0	0	0	No	
NM_hsa_2635	chr14:96505001-96505077	-	TGCTCAGGTTGCACAGCGGGT	No	Yes	No	0	0	0	Yes	
NM_hsa_2638	chr14:105636831-105636928	-	CCGGGCTGGGGGCTCCTGAGG	No	Yes	No	0	0	0	No	
NM_hsa_2640	chr15:29284764-29284846	+	TTCTCTGTGGCTGAGTTCTGCT	No	Yes	No	0	0	0	No	
NM_hsa_2641	chr15:38856428-38856524	+	TCGGCGGGCGGGCGCCGGCT	No	Yes	No	0.142464	0	0	Yes	
NM_hsa_2642	chr15:40750566-40750659	+	TTGCCCACCTTCTGTCCTGCAG	No	Yes	No	0	0	0	Yes	
NM_hsa_2644	chr15:42534005-42534100	+	TAATTTAGTAGGTTGTAAAA	No	Yes	No	0	0	0	No	
NM_hsa_2646	chr15:52122214-52122293	+	CGCGAACCTGGCGTTCCCAGT	No	Yes	No	0	0	0	No	
NM_hsa_2647	chr15:59725246-59725336	+	GTGTGAACTCGGGAGGTGGAA	No	Yes	No	0	0	0	No	
NM_hsa_2651	chr15:69578447-69578508	+	GAACTGAATCGGAGGACACCC	No	Yes	No	0.3989	0.0662225	0.146572	Yes	
NM_hsa_2652	chr15:70286201-70286271	+	ATCTAGATTTTGAGCTCCTGG	No	Yes	No	0.142464	0	0.0732862	Yes	
NM_hsa_2653	chr15:72409959-72410025	+	GACGTGGGCCAGGACGGGCTG	No	Yes	No	0	0	0	No	
NM_hsa_2654	chr15:72523986-72524081	+	ACGGAAAGTTGGGGAGAGCTG	No	Yes	No	0	0	0	No	
NM_hsa_2657	chr15:75504622-75504715	+	CAGATGGCTGTGGCTCTGTGGA	No	Yes	No	0	0	0	No	
NM_hsa_2658	chr15:78423940-78424019	+	CCCGCCGTGGGAGTCCAGGTCG	No	Yes	No	0	0	0	No	
NM_hsa_2662	chr15:83424751-83424828	+	AAGGTAGATAGAACAGGTCTTG	No	Yes	No	13.819	13.7743	15.3168	Yes	
NM_hsa_2663	chr15:83629135-83629227	+	TGTTGAACAGAGGTGGGGCTGAG	No	Yes	No	0	0	0	No	
NM_hsa_2664	chr15:85924258-85924348	+	GGGTGGGGGGCGCGCCCGGC	No	Yes	No	0	0	0	No	
NM_hsa_2666	chr15:89525983-89526067	+	ACTCTCTGGGCTGTGGTTGACA	No	Yes	No	0	0	0	No	
NM_hsa_2671	chr15:90836837-90836920	+	AGACCCGGCTCATTGGACCAG	No	Yes	No	0	0	0	No	
NM_hsa_2675	chr15:91900054-91900139	+	TAATGCCCTTGACCCGGCCT	No	Yes	No	0	0	0	No	
NM_hsa_2677	chr15:97298885-97298953	+	TGGTGAAAAAGGCAGTTCCTT	No	Yes	No	0	0	0	No	
NM_hsa_2678	chr15:99191725-99191823	+	CGCGGCGAGGCTGGGGCTCTTG	No	Yes	No	0	0	0	No	
NM_hsa_2680	chr15:99791692-99791769	+	GCGCTGAGGTGAGTAGAGCTG	No	Yes	No	0	0	0	No	
NM_hsa_2681	chr15:22466012-22466106	-	CTGAGATCTGAGGACACGGCCGT	No	Yes	No	0	0	0	No	
NM_hsa_2682	chr15:28518177-28518257	-	TAAGCATGCTGTCTTTCTGGAG	No	Yes	No	0	0	0	Yes	
NM_hsa_2690	chr15:66586037-66586119	-	TGCAGGCGGCGGGCTGCGGGC	No	Yes	No	0	0	0	No	
NM_hsa_2691	chr15:69452666-69452741	-	GTAGTAGTGACCAGAAGTAG	No	Yes	No	0	0	0	No	
NM_hsa_2693	chr15:75494173-75494250	-	GCTGGGGGCGGGGCGGGGGCG	No	Yes	No	0	0.0331113	0	Yes	
NM_hsa_2694	chr15:78017608-78017698	-	TGGGCTTGAGTGATCCTCCTGC	No	Yes	No	0	0	0	No	
NM_hsa_2695	chr15:89911257-89911330	-	TTCGGTTATCTAGCTTTATGAC	No	Yes	No	0	0	0	Yes	
NM_hsa_2696	chr15:90684480-90684559	-	TATGGCATTTTATGGCTATGTG	No	Yes	No	0	0	0	No	
NM_hsa_2697	chr15:96875213-96875305	-	TGCGGGCGGCGGCGGGGCGG	No	Yes	No	0	0.0662225	0	No	
NM_hsa_2704	chr16:2128380-2128457	+	ATCCACCTCCTCACCTCAGGCC	No	Yes	No	0	0	0	Yes	
NM_hsa_2705	chr16:2185901-2185976	+	CGGCTCCTCCTCCTCCTCCCCG	No	Yes	No	0.0569857	0.0331113	0.0366431	Yes	
NM_hsa_2707	chr16:2222119-2222202	+	TCAGCACGCCCTCCTCTCCCAGA	No	Yes	No	0.284929	0.132445	0.0732862	Yes	KapB+cMyc>KapB>H
NM_hsa_2708	chr16:2228652-2228734	+	CCCCGACCAGGACACGCCCACG	No	Yes	No	0	0	0	No	
NM_hsa_2710	chr16:2550726-2550814	+	CCACTGACCTGAGCATCCTGCAG	No	Yes	No	0.113971	0	0.146572	Yes	
NM_hsa_2711	chr16:2618926-2619003	+	TGGGCTGTGCTCTGGGCATTTT	No	Yes	No	0	0	0	No	
NM_hsa_2713	chr16:3174843-3174906	+	AGAAGAAACGCGCGGGCTGTG	No	Yes	No	0	0	0	No	
NM_hsa_2714	chr16:3208908-3208995	+	GTCCTGGCTCGTTGGTCTAGGGG	No	Yes	No	0.455886	0.816744	2.87038	Yes	H>KapB>KapB+cMyc
NM_hsa_2715	chr16:3214929-3215020	+	GCCTGGCTAGCTCAGTCGGC	No	Yes	No	10.4854	13.1783	42.506	Yes	H>KapB>KapB+cMyc
NM_hsa_2716	chr16:4233345-4233422	+	CTGTTTGCCCTGGGCGCCGGC	No	Yes	No	0	0	0	No	
NM_hsa_2717	chr16:4556830-4556910	+	TTCTGGAAAGCTGGGAAGGGCT	No	Yes	No	0	0.0662225	0.0732862	Yes	H>KapB>KapB+cMyc
NM_hsa_2718	chr16:5083660-5083746	+	CGGGGGAGGCGCGCGCGCGCG	No	Yes	No	0.313421	0.0662225	0.0732862	Yes	
NM_hsa_2719	chr16:9057259-9057333	+	CGCGGCGGGCGGCGGCGGCGGC	No	Yes	No	1.36006	0.805707	0.382957	Yes	KapB+cMyc>KapB>H
NM_hsa_2722	chr16:22308483-22308567	+	TTAAGGCTCCAGTCTCTTCGG	No	Yes	No	0.170957	0.662225	0.146572	Yes	
NM_hsa_2723	chr16:22308483-22308567	+	GCTGCCAGGGTTGCTGTCTTTT	No	Yes	Yes	0	0	0.0732862	No	
NM_hsa_2723	chr16:22308459-22308597	+	GCTGCCAGGGTTGCTGTCTTTT	No	Yes	Yes	0	0	0.0732862	No	
NM_hsa_2725	chr16:26747552-26747632	+	AAAAACCGCAATTACTTTAGC	No	Yes	No	0.00712322	0	0	Yes	
NM_hsa_2726	chr16:27085142-27085226	+	TCAGGGTGGCAGGAGGGCTG	No	Yes	No	0	0	0	No	
NM_hsa_2727	chr16:27135578-27135642	+	GCTGTGGTGGTTCAGTGGTTC	No	Yes	No	0.284929	0.331113	0.293145	No	
NM_hsa_2728	chr16:27201799-27201876	+	ACAGCTGTAGGCTTGCACAGA	No	Yes	Yes	0	0	0	No	
NM_hsa_2728	chr16:27201784-27201890	+	ACAGCTGTAGGCTTGCACAGA	No	Yes	Yes	0	0	0	No	
NM_hsa_2729	chr16:28190478-28190548	+	GTTCTCTATAGGACGCCATAGC	No	Yes	No	0.0569857	0.331113	0.146572	Yes	
NM_hsa_2730	chr16:28214647-28214735	+	AGAGGTGGGAGGTTGAAAAA	No	Yes	No	0	0	0	No	
NM_hsa_2731	chr16:28746010-28746084	+	GTGATGGGTCGGGGAAGAAGAGAC	No	Yes	No	0	0	0	No	
NM_hsa_2732	chr16:28884685-28884777	+	TTTTCTTACCATTCCTATCCAG	No	Yes	No	0.0569857	0	0	No	
NM_hsa_2734	chr16:30076437-30076506	+	CTGGAACTCGGATGGGGAGGT	No	Yes	No	0	0	0	No	
NM_hsa_2735	chr16:30670430-30670516	+	CCCCTGGCAGGGGGTGGCCACCG	No	Yes	No	0	0	0	No	
NM_hsa_2736	chr16:32687639-32687738	+	CTCGCCAGTGAGGAGGATGTGTT	No	Yes	No	0	0	0	No	
NM_hsa_2737	chr16:50678499-50678568	+	CTCGGCCTGTGGGCTCTCAGC	No	Yes	No	0	0	0	No	
NM_hsa_2739	chr16:52575017-52575108	+	GAATCAGAATCTCCAAGGGTA	No	Yes	No	0	0	0	No	
NM_hsa_2742	chr16:57690349-57690434	+	AGCCCCGTGCTGTCCCCTCCTCA	No	Yes	No	0.284929	0.0662225	0.0732862	No	
NM_hsa_2743	chr16:57831640-57831724	+	CAGCTTGGCCAGAGGGAATG	No	Yes	No	0	0	0	No	
NM_hsa_2744	chr16:58457316-58457403	+	CAGAGATGGAGGACTGCTAGAGT	No	Yes	No	0	0	0	No	
NM_hsa_2745	chr16:59525415-59525498	+	GTAGATGAAGCACTGTTGCT	No	Yes	No	0	0.0882967	0.097715	No	H>KapB>KapB+cMyc
NM_hsa_2746	chr16:66583779-66583861	+	CGCGCGTTACCGGGAGGCCAG	No	Yes	No	0	0	0	No	
NM_hsa_2747	chr16:66982132-66982211	+	CTGTGGGGATCCTGAACGTG	No	Yes	No	0	0	0	No	
NM_hsa_2748	chr16:67035539-67035629	+	AGCACTGATGAGAAAACTGAGC	No	Yes	No	0.170957	1.19201	1.6123	No	H>KapB>KapB+cMyc
NM_hsa_2749	chr16:67237174-67237264	+	CGGGGCTGCCAGGGCTGCAGT	No	Yes	No	0	0	0	No	
NM_hsa_2750	chr16:67315536-67315656	+	CACAACACCTTCTTCTCCTGTAG	No	Yes	Yes	0	0	0	No	
NM_hsa_2750	chr16:67315552-67315636	+	CACAACACCTTCTTCTCCTGTAG	No	Yes	Yes	0	0	0	No	
NM_hsa_2751	chr16:67438923-67439003	+	GAGCTGATGACCAGACCCTGG	No	Yes	No	0	0	0	No	
NM_hsa_2752	chr16:67969870-67969956	+	GCGCAGGATGCGAGTGACCG	No	Yes	No	0	0	0	No	
NM_hsa_2754	chr16:68506469-68506544	+	CTGAGGGGCTCTGGCTTCCG	No	Yes	No	0	0	0	No	
NM_hsa_2755	chr16:69796269-69796365	+	TCTGTGGCCACGGCAGGTAG	No	Yes	No	0	0	0	No	
NM_hsa_2757	chr16:70328919-70329014	+	TGATTTCAGGGGAAACTGAGA	No	Yes	No	0	0	0	No	
NM_hsa_2760	chr16:79854241-79854326	+	GACTGGCGGGGTGGAACCTGG	No	Yes	No	0	0	0	No	
NM_hsa_2761	chr16:81511727-81511806	+	TATCCCAGGGCCAGTCTCCACT	No	Yes	No	0.170957	1.12578	0.513004	Yes	
NM_hsa_2763	chr16:83667740-83667825	+	TTTGTGTGACCACCACCACCA	No	Yes	No	0	0	0	No	
NM_hsa_2766	chr16:85655547-85655626	+	CAGGTCTGCACCTGACTGGGA	No	Yes	No	0	0	0	No	
NM_hsa_2768	chr16:87887502-87887570	+	TCACGTCTGCGGCTGTCACG	No	Yes	No	0	0	0	Yes	
NM_hsa_2770	chr16:88572897-88572966	+	TGGGGGCGGGCGGGCAGGGCA	No	Yes	No	0	0	0	No	
NM_hsa_2771	chr16:88877879-88877960	+	CCCTAGATGCGGCCACTGGG	No	Yes	No	0	0	0	No	
NM_hsa_2772	chr16:89297532-89297619	+	AAGGTCATCAGATTAGCGGGA	No	Yes	No	0.227943	0.132445	0.0732862	Yes	KapB+cMyc>KapB>H
NM_hsa_2773	chr16:89298089-89298186	+	TAGGAGTTAGTAGTGTCTGTGA	No	Yes	No	0	0	0	No	
NM_hsa_2774	chr16:89642093-89642173	+	CCCGGCCCCGCCCCCCGGCCG	No	Yes	No	0	0.0331113	0	No	
NM_hsa_2775	chr16:89988969-89989048	+	TCTTGCCTGGCTTGTGCGTCCTTG	No	Yes	No	0.142464	0	0.0732862	Yes	
NM_hsa_2776	chr16:89988960-89989058	+	ACGGATGCGCACCGGGCAGTGG	No	Yes	Yes	0	0.0662225	0	No	
NM_hsa_2776	chr16:89988969-89989048	+	ACGGATGCGCACCGGGCAGTGG	No	Yes	Yes	0	0.0662225	0	No	
NM_hsa_2777	chr16:402670-402751	-	GCGCTCGGGCCTGGGCTTCGG	No	Yes	No	0	0	0	No	
NM_hsa_2778	chr16:1282798-1282863	-	TTGGATCCGTCTGGGCTTTGCT	No	Yes	No	0	0.0331113	0	No	
NM_hsa_2779	chr16:3786807-3786902	-	TGTGTGCGTGGGTCCTGCAG	No	Yes	Yes	0	0	0	Yes	
NM_hsa_2779	chr16:3786777-3786911	-	TGTGTGCGTGGGTCCTGCAG	No	Yes	Yes	0	0	0	Yes	
NM_hsa_2780	chr16:5860500-5860594	-	GTGCTGAGCTGAGCATCGCCT	No	Yes	No	0	0	0	No	
NM_hsa_2781	chr16:15736789-15736869	-	GTCCTGCGACCAGACCCCGG	No	Yes	No	0	0	0	No	
NM_hsa_2782	chr16:16403903-16403990	-	CCGCGGCTCCTCCTCCTCCTCCC	No	Yes	No	0	0.0331113	0.0366431	No	H>KapB>KapB+cMyc
NM_hsa_2785	chr16:28917190-28917275	-	CTCTTTCTTTCCTTCCCACAG	No	Yes	No	0	0	0	Yes	
NM_hsa_2788	chr16:51645132-51645231	-	TCTTTGATTATCTAGGAGTATGA	No	Yes	No	0	0	0	Yes	
NM_hsa_2789	chr16:63778244-63778321	-	GCAGTGCATGGGCACATATAC	No	Yes	No	0	0	0	Yes	
NM_hsa_2790	chr16:66617809-66617877	-	TCAAGTACAGTACAGTGGGGG	No	Yes	No	0	0	0	No	
NM_hsa_2791	chr16:66785531-66785618	-	GGTCGGCGGGCGGCGCGGCG	No	Yes	No	0.07978	0.0132445	0	No	KapB+cMyc>KapB>H
NM_hsa_2797	chr16:87723415-87723507	-	GCGGGCGCGGGCGGCGGGGT	No	Yes	No	0	0	0	No	

Additional file 5

Name	locus	strand	sequence	mirdeep2	mireap	miranalyzer	RPM_HU_KapB_cMyc	RPM_HU_KaposinB	RPM_HUVEC	IP_confidence	annotation
NM_hsa_2798	chr16:88519967-88520040	-	GGGCGGCGGCGGGCGGCGGGGGGC	No	Yes	No	0.968758	0.695336	0.366431	Yes	KapB+cMyc>KapB>H
NM_hsa_2799	chr17:1133028-1133113	+	GGGGGCGGGCGCCCCGGGGA	No	Yes	No	0.284929	0.26489	0.366431	No	
NM_hsa_2802	chr17:2266213-2266293	+	CTGGCTGTCTCTGTCTCCACAGT	No	Yes	No	0	0	0	Yes	
NM_hsa_2803	chr17:2441320-2441417	+	TGCTGAGGCTGCTGATCTGAGG	No	Yes	No	0	0	0	No	
NM_hsa_2804	chr17:2615300-2615387	+	ACTGTGACCTTGGGTGGAGA	No	Yes	No	0	0	0	No	
NM_hsa_2805	chr17:2885704-2885771	+	TTTCAGTGTTCAGGATTGGG	No	Yes	No	0	0	0	No	
NM_hsa_2808	chr17:4621152-4621235	+	CTGCCCCTACTCTGATCCCAG	No	Yes	No	0.227943	0	0	No	
NM_hsa_2809	chr17:4794684-4794761	+	TCCCTGGTCTCTCCCTGCAGA	No	Yes	No	0	0	0	Yes	
NM_hsa_2810	chr17:6347800-6347869	+	TGAGTGCGGGCTGGGCACAAGT	No	Yes	No	0.0284929	0	0	Yes	
NM_hsa_2811	chr17:6843859-6843928	+	AAGGGATGAATCGGGCTTGGGT	No	Yes	Yes	0	0	0	No	
NM_hsa_2811	chr17:6843859-6843937	+	AAGGGATGAATCGGGCTTGGGT	No	Yes	Yes	0	0	0	No	
NM_hsa_2812	chr17:6974383-6974476	+	CTCTGGGGTCTTCTGCTTGT	No	Yes	No	0	0	0	No	
NM_hsa_2816	chr17:8090489-8090581	+	CCGTGTTTCCCCCACGCTTT	No	Yes	No	0.227943	0.198668	0.146572	Yes	KapB+cMyc>KapB>H
NM_hsa_2820	chr17:16342755-16342849	+	TGTCCTGATGATACTTGTAAT	No	Yes	No	0	0.26489	0	Yes	
NM_hsa_2821	chr17:17598203-17598283	+	AGTCCCAGCGTTGCGGCCGGCG	No	Yes	Yes	0	0	0	No	
NM_hsa_2821	chr17:17598188-17598296	+	AGTCCCAGCGTTGCGGCCGGCG	No	Yes	Yes	0	0	0	No	
NM_hsa_2822	chr17:17598203-17598283	+	AGGCGGCGCGCATCTCTGGGC	No	Yes	Yes	0.227943	0.132445	0.0732862	Yes	KapB+cMyc>KapB>H
NM_hsa_2822	chr17:17598192-17598298	+	AGGCGGCGCGCATCTCTGGGC	No	Yes	Yes	0.227943	0.132445	0.0732862	Yes	KapB+cMyc>KapB>H
NM_hsa_2823	chr17:17717159-17717243	+	CAATGCAACAGCAATGCACCGC	No	Yes	No	0	0.26489	0	Yes	
NM_hsa_2824	chr17:17991719-17991792	+	CTGGGGCCTGGGTCTACTCTG	No	Yes	No	0	0	0	Yes	
NM_hsa_2825	chr17:18156856-18156949	+	TGCACAGGGACAGCTCCGTG	No	Yes	No	0	0	0	No	
NM_hsa_2826	chr17:18266961-18267058	+	AGAACTGACCCGCTTTGGCTG	No	Yes	No	0	0	0	No	
NM_hsa_2827	chr17:18684606-18684682	+	CAGGTGAGGGGCTGAGGGCTCG	No	Yes	No	0	0	0	No	
NM_hsa_2828	chr17:18965290-18965366	+	CCCCGAGGAAGAGAGGTAGCG	No	Yes	No	0.569857	0.52978	0.146572	Yes	KapB+cMyc>KapB>H
NM_hsa_2829	chr17:19266387-19266487	+	ACAGCTGTCGGGGCTAACTCTGG	No	Yes	Yes	0	0	0	No	
NM_hsa_2829	chr17:19266397-19266478	+	ACAGCTGTCGGGGCTAACTCTGG	No	Yes	Yes	0	0	0	No	
NM_hsa_2830	chr17:19651358-19651446	+	CGCGGATCCCTGGGACCTCTGCC	No	Yes	Yes	0	0	0	No	
NM_hsa_2830	chr17:19651338-19651458	+	CGCGGATCCCTGGGACCTCTGCC	No	Yes	Yes	0	0	0	No	
NM_hsa_2831	chr17:27070921-27070993	+	GGGCGGGGCCGCGAGGGCGCG	No	Yes	No	0	0	0	Yes	
NM_hsa_2832	chr17:27074779-27074869	+	TGGGTCTAGGGGTGGGAACTGG	No	Yes	No	0	0	0	Yes	
NM_hsa_2836	chr17:29892015-29892097	+	CCGCTGCCTGATTTTCCTCTGA	No	Yes	No	0	0	0	No	
NM_hsa_2839	chr17:31149603-31149699	+	CGCGCCGAGCCGGGCCCGTGG	No	Yes	No	0	0	0	No	
NM_hsa_2841	chr17:35969612-35969678	+	GCTCTCGGGCTGGTGGGGCGGT	No	Yes	No	0	0	0	No	
NM_hsa_2842	chr17:36875913-36876031	+	AAGGGCCAGAGGAGCCTGGAGT	No	Yes	Yes	0.0854786	0.0331113	0	Yes	KapB+cMyc>KapB>H
NM_hsa_2842	chr17:36875933-36876012	+	AAGGGCCAGAGGAGCCTGGAGT	No	Yes	Yes	0.0854786	0.0331113	0	Yes	KapB+cMyc>KapB>H
NM_hsa_2843	chr17:36875912-36876032	+	ACCCAGGTTCCCTCTGGCCGCAG	No	Yes	Yes	0	0	0	Yes	
NM_hsa_2843	chr17:36875933-36876012	+	ACCCAGGTTCCCTCTGGCCGCAG	No	Yes	Yes	0	0	0	Yes	
NM_hsa_2844	chr17:36955615-36955713	+	GGGGCGGCGGCGGCGGCGGCGA	No	Yes	No	0.500817	0.534315	0.36844	Yes	
NM_hsa_2845	chr17:37202419-37202510	+	TTGGAACTGGTAGGCTTTCATT	No	Yes	No	0	0	0	No	
NM_hsa_2846	chr17:37896686-37896775	+	CCAGGCCGGGCATTGCCTCTCT	No	Yes	No	0	0	0	Yes	
NM_hsa_2849	chr17:39184971-39185044	+	AATGGCAATGGTAGAACTCA	No	Yes	No	0	0	0	Yes	
NM_hsa_2850	chr17:39653271-39653336	+	GCACCAGGAGTCACAGAACAG	No	Yes	No	0	0	0	No	
NM_hsa_2851	chr17:39805762-39805844	+	CTCTGAGTGTCTCTGGGGTTGC	No	Yes	No	0	0	0	No	
NM_hsa_2852	chr17:39845788-39845882	+	AAGCTGGCCGTCCCGGACTGAC	No	Yes	No	0	0	0	No	
NM_hsa_2853	chr17:39967644-39967712	+	CCCGGGTGGGGGCGGGCTCC	No	Yes	No	0	0	0	No	
NM_hsa_2855	chr17:41446157-41446246	+	CAGGTTCGAGGGCTGGGCGGAG	No	Yes	No	0	0	0	No	
NM_hsa_2856	chr17:42432096-42432164	+	GGTCGAAGGGCGGCGGCGGC	No	Yes	No	0.303924	0.110371	0	Yes	KapB+cMyc>KapB>H
NM_hsa_2857	chr17:46100953-46101041	+	CTGCGCCCCCAGTTGGCTCTG	No	Yes	No	0	0	0	No	
NM_hsa_2858	chr17:47781712-47781794	+	CCCACCCCCGCCCCCCGGCC	No	Yes	No	0	0	0	No	
NM_hsa_2859	chr17:48005564-48005663	+	GACGGCCCTGCCCTCCGGGACCAG	No	Yes	No	0	0	0	No	
NM_hsa_2861	chr17:53497607-53497694	+	CACTGATCATGACTGGAGTG	No	Yes	No	0	0	0	No	
NM_hsa_2862	chr17:55062308-55062448	+	AACCCCAGAGGTAGAAGCTGTGG	No	Yes	Yes	0	0	0	No	
NM_hsa_2862	chr17:55062327-55062418	+	AACCCCAGAGGTAGAAGCTGTGG	No	Yes	Yes	0	0	0	No	
NM_hsa_2864	chr17:56085681-56085752	+	TTCGACGTCTGAAATCTGGAT	No	Yes	No	0.113971	0	0	Yes	
NM_hsa_2865	chr17:56715137-56715230	+	GTTTACAACCTACTGCCTTT	No	Yes	No	0	0	0	No	
NM_hsa_2870	chr17:59407920-59407996	+	AGTCCATGATGAAGCTGAGA	No	Yes	No	0	0	0	No	
NM_hsa_2871	chr17:61523780-61523850	+	GCGGCCACGCTCGGGACCGG	No	Yes	No	0	0	0	No	
NM_hsa_2872	chr17:61777033-61777119	+	CTGCCCCGACCTGACCCCGGC	No	Yes	No	0	0	0	No	
NM_hsa_2873	chr17:62971766-62971863	+	CGCGCCAGAGCTGGAGTTCGG	No	Yes	No	0	0	0	No	
NM_hsa_2874	chr17:63557142-63557216	+	GGAACGGCGCGCCCCGCCGC	No	Yes	No	0	0	0	No	
NM_hsa_2875	chr17:65989441-65989530	+	CGCGGGGCGGCGGGGCGGCG	No	Yes	No	0	0	0	No	
NM_hsa_2876	chr17:73084285-73084362	+	AGCCCAAGGGCGCTCGGACT	No	Yes	No	0	0	0	No	
NM_hsa_2878	chr17:74902025-74902109	+	TCTGTGTTCCGCCCACCCAG	No	Yes	No	0	0	0	No	
NM_hsa_2879	chr17:76349039-76349136	+	CTCTGCAGGGCGCAGCGGCGG	No	Yes	No	0	0	0	No	
NM_hsa_2880	chr17:77102261-77102335	+	AGGCCAGGGCTGGGGCTGCTGC	No	Yes	No	0	0	0	No	
NM_hsa_2881	chr17:78234296-78234370	+	CGCGGGGCCGGCGGCGGCGG	No	Yes	No	0.0427393	0.087193	0.128251	Yes	H>KapB>KapB+cMyc
NM_hsa_2887	chr17:78428687-78428769	+	GTGCTCGGGGCTTGGCCGTAG	No	Yes	No	0	0	0	No	
NM_hsa_2889	chr17:79373923-79374016	+	CCTCGCCACCCTCCCCCGCCCC	No	Yes	No	0	0	0	No	
NM_hsa_2890	chr17:79982468-79982606	+	ACTACCGGCCTGTTTCTCTCCT	No	Yes	Yes	0	0	0	Yes	
NM_hsa_2890	chr17:79982491-79982576	+	ACTACCGGCCTGTTTCTCTCCT	No	Yes	Yes	0	0	0	Yes	
NM_hsa_2892	chr17:80376560-80376654	+	GCGGGTGCGGCTCTGGCTCTG	No	Yes	No	0	0	0	No	
NM_hsa_2893	chr17:81053666-81053744	+	ACATTGGTCACTGGATTGGGG	No	Yes	No	0	0	0	No	
NM_hsa_2895	chr17:1174216-1174303	-	CGGTGTCCCCGCGCGCGGCC	No	Yes	No	0	0	0	No	
NM_hsa_2897	chr17:7164926-7165021	-	CGGCTGGGGGCGGCGGGGCTGGG	No	Yes	No	0	0	0.0732862	No	
NM_hsa_2898	chr17:8534381-8534460	-	GCGCCGGCCGCCGCGGGGCC	No	Yes	No	0.0569857	0	0	Yes	
NM_hsa_2904	chr17:27507289-27507362	-	CGGCGGCGGCGGCCGCTCGG	No	Yes	No	0	0	0	Yes	
NM_hsa_2907	chr17:32706138-32706226	-	AGGTAGTAGGTTGCGTAGTTT	No	Yes	No	0.0455886	0.0662225	0.0439718	Yes	
NM_hsa_2908	chr17:36433249-36433323	-	GTCTAGTTTTCCCTGGAATTC	No	Yes	No	0	0	0	No	
NM_hsa_2909	chr17:36888003-36888086	-	GAAATACCAGGTGCTGTAGGCTT	No	Yes	No	65.9779	39.8119	39.6344	Yes	KapB+cMyc>KapB>H
NM_hsa_2910	chr17:38278611-38278692	-	GCCTCGCCGCCGCCTCTGCTGC	No	Yes	No	0.341914	0.26489	0.146572	Yes	KapB+cMyc>KapB>H
NM_hsa_2911	chr17:41907466-41907555	-	CGCCTGTCTCATTCTCTGCAG	No	Yes	No	0.113971	0	0.0732862	Yes	
NM_hsa_2920	chr17:58154393-58154488	-	CACTAGTTTGTGAGCTCTTTGAG	No	Yes	No	0.0142464	0	0	No	
NM_hsa_2921	chr17:63052312-63052386	-	CGGCGGGTGCGGGGCGGGGC	No	Yes	No	0	0	0	No	
NM_hsa_2922	chr17:65376258-65376340	-	AATCCAGGTGGCGGAGGTTGC	No	Yes	No	0	0	0	Yes	
NM_hsa_2923	chr17:70417105-70417181	-	TCCAGGAGGAGTGGGCTGTGG	No	Yes	No	0	0	0	No	
NM_hsa_2927	chr17:80304926-80304998	-	GGGGACCGGGTCGGGGTCGC	No	Yes	No	0	0	0	No	
NM_hsa_2931	chr18:5295513-5295593	+	CTCCCCGAGCCCGCTCGCCGG	No	Yes	No	0	0	0	No	
NM_hsa_2935	chr18:22731481-22731565	+	AAGCAGAACTTTGAAGACTG	No	Yes	No	0	0	0	Yes	
NM_hsa_2936	chr18:24128979-24129074	+	GGGGGGTCGGGGCGGCGGCT	No	Yes	No	1.36766	1.35756	2.74823	Yes	
NM_hsa_2937	chr18:24405104-24405180	+	AGTGATGAAGAGGGCTGAGG	No	Yes	No	0	0	0	No	
NM_hsa_2939	chr18:42260720-42260817	+	GAGTGGACTCGGGAGGGGGAGGG	No	Yes	No	0	0	0	No	
NM_hsa_2940	chr18:43423095-43423176	+	TCTGACCTCTCTGACCTGCAGC	No	Yes	No	0	0	0	No	
NM_hsa_2941	chr18:45456878-45456945	+	GGCCCGGGCGCGCGGGAGGG	No	Yes	No	0.227943	0.397335	0.146572	No	
NM_hsa_2942	chr18:45711093-45711177	+	TGACTGTCCGCCAGGAGCTCA	No	Yes	No	0	0	0	No	
NM_hsa_2943	chr18:46479149-46479215	+	TTGTGCACAGCTCTGGAATG	No	Yes	No	0	0	0	No	
NM_hsa_2944	chr18:48086405-48086498	+	TGGAGCAGCGAGCCGGGCTGT	No	Yes	No	0	0	0	No	
NM_hsa_2946	chr18:55289155-55289228	+	CGTTTGTGCAGCTCGGAAGG	No	Yes	No	0	0	0	No	
NM_hsa_2949	chr18:61034534-61034630	+	GCTTCCAGGGGGCGAGTAGG	No	Yes	No	0	0	0	No	
NM_hsa_2950	chr18:61110210-61110303	+	TGTGAGCTTTGCATCTGAGGCA	No	Yes	No	0	0	0	No	
NM_hsa_2951	chr18:68879684-68879769	+	GGCGGCGGGGGGCGGGGCGGGGC	No	Yes	No	1.2005	0.708581	0.323681	Yes	KapB+cMyc>KapB>H
NM_hsa_2952	chr18:72408731-72408822	+	TGCTTCCTGGTAATGATGACA	No	Yes	No	0	0	0	No	
NM_hsa_2953	chr18:72920665-72920738	+	TGGGCACGCGGGCGGGCTGGG	No	Yes	No	0	0	0	No	
NM_hsa_2955	chr18:77202574-77202660	+	GACGTGCGGTGGCGGCGGCGG	No	Yes	No	0	0	0	No	
NM_hsa_2956	chr18:77905941-77906035	+	CTGCCTTGCGGGTCACTGTGG	No	Yes	No	0	0	0	No	
NM_hsa_2957	chr18:985314-985403	-	TGACATGATGCACAGTAGAT	No	Yes	No	0	0	0	No	
NM_hsa_2958	chr18:7567766-7567863	-	CCGCTCCCCGGCGGCGGTGGG	No	Yes	No	0	0	0	No	
NM_hsa_2959	chr18:9137652-9137731	-	ACAGAACTGTAGAGTGGCTG	No	Yes	No	0	0	0	No	
NM_hsa_2960	chr18:12906954-12907035	-	CTTTGGTGACTCTCGATTGCC	No	Yes	No	0.0569857	0	0.0732862	Yes	
NM_hsa_2965	chr18:53146445-53146531	-	ATTGGACTGCTGATGGCCTGT	No	Yes	No	0	0	0.0244288	No	
NM_hsa_2966	chr18:66870602-66870692	-	GTCTATGTAGCTTTACTCCT	No	Yes	No	0	0	0	No	
NM_hsa_2968	chr18:73128100-73128170	-	AACATTGATTGCTGTCGAGG	No	Yes	No	0	0.0331113	0	Yes	
NM_hsa_2970	chr19:589828-589925	+	CGGCCGGCGGCGGCGGCGGCGGCT	No	Yes	No	0.869641	0.701711	0.519899	Yes	KapB+cMyc>KapB>H
NM_hsa_2971	chr19:804919-805016	+	GACGTCTCACGGTCCCTCTCC	No	Yes	No	0	0	0	Yes	
NM_hsa_2973	chr19:1253351-1253436	+	CTGGGAGGCGGTCGGTTCTGAG	No	Yes	No	0	0	0	No	
NM_hsa_2975	chr19:2271716-2271790	+	TTGGGCTGCACCCCCTGGACAG	No	Yes	No	0.113971	0.0662225	0.146572	Yes	
NM_hsa_2976	chr19:2339977-2340115	+	CTCACGGCCCTGCCCCTGCAGT	No	Yes	Yes	0	0	0	Yes	
NM_hsa_2976	chr19:2339987-2340085	+	CTCACGGCCCTGCCCCTGCAGT	No	Yes	Yes	0	0	0	Yes	
NM_hsa_2977	chr19:3099451-3099535	+	CTCCTGCCGCCAGGCCCTGTGT	No	Yes	No	0	0	0	No	
NM_hsa_2978	chr19:3192355-3192448	+	GGGCTGGGGCTGGGGCTGCTG	No	Yes	No	0	0	0	No	
NM_hsa_2981	chr19:6464341-6464430	+	GCCCGGGCCAGGTAGGAAGG	No	Yes	No	0	0	0	No	
NM_hsa_2982	chr19:7506709-7506807	+	CCACACCTCGCTCTTCCCTCAG	No	Yes	Yes	0	0	0	Yes	
NM_hsa_2982	chr19:7506699-7506837	+	CCACACCTCGCTCTTCCCTCAG	No	Yes	Yes	0	0	0	Yes	
NM_hsa_2983	chr19:7533646-7533720	+	TATCTGCTGTTGTCCCCTCAG	No	Yes	Yes	0.0284929	0	0	Yes	
NM_hsa_2983	chr19:7533639-7533725	+	TATCTGCTGTTGTCCCCTCAG	No	Yes	Yes	0.0284929	0	0	Yes	
NM_hsa_2984	chr19:7553993-7554073	+	GCCTTTGTCCCCCTGACCGG	No	Yes	No	0	0	0	No	
NM_hsa_2985	chr19:7573004-7573102	+	CTGTCCACCCTGTCTCCACAGT	No	Yes	No	0	0	0	No	
NM_hsa_2986	chr19:7620062-7620135	+	CCTGGGGGATCCGCCGGACC	No	Yes	No	0.0569857	0.198668	0.0732862	No	
NM_hsa_2989	chr19:8578840-8578920	+	AGGCTGTAGGCGCGACAGGGC	No	Yes	No	0	0	0	No	
NM_hsa_2990	chr19:8590860-8590947	+	AAGTCTGTCTCTCTGGCCGGG	No	Yes	No	0	0	0	No	
NM_hsa_2991	chr19:9653379-9653476	+	TTACAGTGGAGGGCAAGTCTG	No	Yes	No	0.170957	0.132445	0.0732862	Yes	KapB+cMyc>KapB>H
NM_hsa_2993	chr19:10750129-10750201	+	GCAGGTGATGTTGGATCTGAGA	No	Yes	No	0.0569857	0.0662225	0	No	
NM_hsa_2994	chr19:10939643-10939722	+	TCCCCAACCCCTGCCCGCAGA	No	Yes	Yes	0	0	0.0732862	Yes	
NM_hsa_2994	chr19:10939616-10939752	+	TCCCCAACCCCTGCCCGCAGA	No	Yes	Yes	0	0	0.0732862	Yes	
NM_hsa_2995	chr19:12897930-12898019	+	TAGCTGAACTCTAGCCTGAGC	No	Yes	No	0	0	0.0244288	No	
NM_hsa_2996	chr19:13044705-13044781	+	GGCGTCTGGTTGACCAGAGGGG	No	Yes	No	0	0	0	No	
NM_hsa_2997	chr19:13063409-13063537	+	CCTTCCCCACCCTCTCCTGCAG	No	Yes	Yes	0.170957	0	0	Yes	
NM_hsa_2997	chr19:13063430-13063512	+	CCTTCCCCACCCTCTCCTGCAG	No	Yes	Yes	0.170957	0	0	Yes	
NM_hsa_2998	chr19:13961414-13961508	+	GGCGCCCAGCAGCCTGGCCGG	No	Yes	No	0	0	0	No	
NM_hsa_3001	chr19:13990109-13990197	+	CAGCAGACTGTGAGCTCCTGGA	No	Yes	No	0	0	0	Yes	
NM_hsa_3003	chr19:14071688-14071761	+	TTGTCTCCACCTGTCCCTGTAG	No	Yes	No	0	0	0	No	
NM_hsa_3004	chr19:14270047-14270122	+	TGCAGAACGAGACTCTGGCA	No	Yes	No	9.74456	17.1516	22.9386	Yes	H>KapB>KapB+cMyc
NM_hsa_3005	chr19:14629606-14629690	+	CCCGGGAGGCGATGAACCTG	No	Yes	No	0	0	0	No	
NM_hsa_3008	chr19:19844017-19844084	+	GTCCGGCTTGTCCCCGCCAGG	No	Yes	No	0	0	0	No	
NM_hsa_3009	chr19:20654056-20654125	+	GTTTTGAATCCTGTTCGTGACGC	No	Yes	No	0.0284929	0	0	No	
NM_hsa_3010	chr19:21623434-21623499	+	CTGCTCCTGGCTGGCTCGCCA	No	Yes	No	131.523	129.763	187.356	Yes	
NM_hsa_3011	chr19:30433609-30433679	+	CCTGCCTGTGCTCTGGGCCGC	No	Yes	No	0	0	0	No	
NM_hsa_3016	chr19:35500716-35500801	+	TCACCTCTCAAACTTCCTCAG	No	Yes	No	0	0	0	Yes	
NM_hsa_3018	chr19:36223043-36223125	+	TAACACCAACCCTCCCCCCACAG	No	Yes	No	0	0	0	Yes	
NM_hsa_3019	chr19:36540004-36540077	+	AGCTGGTCCGAGTGTTGTGG	No	Yes	No	0.142464	0.165556	0.109929	Yes	
NM_hsa_3020	chr19:36870163-36870259	+	TCCGCGCTGCGTGGGGCTGAG	No	Yes	No	0	0	0	Yes	
NM_hsa_3022	chr19:37782737-37782836	+	GGTTGCGGCGGCGGCGGTGGCGG	No	Yes	No	0.0772595	0.0633996	0.0897756	No	
NM_hsa_3025	chr19:39214980-39215076	+	TCCCACACCGCGTCTCCTCTG	No	Yes	No	0.0569857	0	0.0366431	Yes	
NM_hsa_3026	chr19:39976949-39977046	+	TCGCTCCCTTCCCACCCCCAG	No	Yes	No	0	0	0	Yes	
NM_hsa_3027	chr19:41049415-41049508	+	ACTCTGCACTACAGAACTTTG	No	Yes	No	0	0	0	Yes	
NM_hsa_3029	chr19:42734386-42734474	+	GTGGGGTGAGGAGGGAGTAGA	No	Yes	No	0	0	0	No	
NM_hsa_3030	chr19:42788576-42788660	+	CGCGCGCGCCGGGGGGCGGGGC	No	Yes	No	0	0.0662225	0	Yes	
NM_hsa_3033	chr19:44413906-44413997	+	ATGAGATAAAGCTCTGTAGCT	No	Yes	No	0	0	0	No	
NM_hsa_3034	chr19:44711830-44711910	+	CCTCTCGGCTGAGCCTCACGTT	No	Yes	No	0.0569857	0	0	Yes	

Additional file 5

Name	locus	strand	sequence	mirdeep2	mireap	miranalyzer	RPM_HU_KapB_cMyc	RPM_HU_KaposinB	RPM_HUVEC	IP_confidence	annotation
NM_hsa_3035	chr19:44861166-44861231	+	TCTGACCCTGTGGATCTTCTGG	No	Yes	No	0	0	0	No	
NM_hsa_3036	chr19:45260190-45260283	+	CTCACAGTCACTGTTCCCCAG	No	Yes	No	0.0284929	0	0	No	
NM_hsa_3041	chr19:46477190-46477288	+	CCCGGGGGGCGGCGGGCGCGGGC	No	Yes	No	0	0	0	No	
NM_hsa_3043	chr19:46891729-46891814	+	TGTCTCTCCCTTCTGCCCACC	No	Yes	Yes	0	0	0	No	
NM_hsa_3043	chr19:46891718-46891834	+	TGTCTCTCCCTTCTGCCCACC	No	Yes	Yes	0	0	0	No	
NM_hsa_3045	chr19:47880101-47880181	+	ATGGAAGGGTGTCTCGCTGCAG	No	Yes	No	0	0	0	Yes	
NM_hsa_3046	chr19:48199644-48199718	+	CACTCGCCTTTCTTCTCCCAGT	No	Yes	No	0.0569857	0	0	Yes	
NM_hsa_3047	chr19:48259161-48259229	+	GCTGCCCCTGGGCTGGGCCAG	No	Yes	No	0	0	0	No	
NM_hsa_3048	chr19:48259161-48259229	+	CAATGCCCAGGGGCTGAGGGCA	No	Yes	No	0.0569857	0.0662225	0	No	
NM_hsa_3050	chr19:50167878-50167975	+	CGCGCAGCTCCGGGTTTCCAG	No	Yes	No	0	0	0	No	
NM_hsa_3052	chr19:50393137-50393200	+	TTGACCGCCGTCTCCACCCAGC	No	Yes	No	0	0	0	No	
NM_hsa_3053	chr19:51925369-51925465	+	GCGGCACCGCGGAGCCTCGG	No	Yes	No	0	0	0	Yes	
NM_hsa_3061	chr19:53104501-53104584	+	AGATCAGCGCGAACCTGGAAG	No	Yes	No	0	0	0	No	
NM_hsa_3062	chr19:53700319-53700395	+	CGGATCGCGGGGAGTGAAGGC	No	Yes	No	0	0	0	No	
NM_hsa_3088	chr19:54485222-54485310	+	CGGGGGCGGGGCCGGGGGTGG	No	Yes	No	0.968757	0.397335	0.293145	Yes	KapB+cMyc>KapB>H
NM_hsa_3090	chr19:56112095-56112182	+	TTGCTCCTCTCTGGACAGCAGT	No	Yes	No	0	0.0662225	0	No	
NM_hsa_3094	chr19:59061035-59061102	+	TCTGGGTGTTGGGCTGTCTGGA	No	Yes	No	0.0569857	0.132445	0	Yes	
NM_hsa_3096	chr19:1469558-1469651	-	GCGCCGTCGGGCCTGCGGGC	No	Yes	No	0	0	0	No	
NM_hsa_3097	chr19:1652254-1652351	-	GCCGCCCCCCCCCACGCGCCG	No	Yes	No	0.0569857	0	0	No	
NM_hsa_3098	chr19:2762628-2762702	-	TCCTGGAGCTGGGCAGATGGGA	No	Yes	No	0	0	0	Yes	
NM_hsa_3101	chr19:8152823-8152908	-	CACCTCCACTGGCCTCCCCA	No	Yes	No	0	0	0	No	
NM_hsa_3103	chr19:10926297-10926377	-	TGGCTGTGCCATCCCAGGGAC	No	Yes	No	0	0.0662225	0	No	
NM_hsa_3109	chr19:16199195-16199288	-	GTTAAGATGGCACAGCCCCCT	No	Yes	No	61.6586	96.7511	104.286	Yes	H>KapB>KapB+cMyc
NM_hsa_3111	chr19:18631499-18631582	-	GGGGGGGTGCCGCGGGCGGGG	No	Yes	No	0	0.0662225	0.0732862	No	H>KapB>KapB+cMyc
NM_hsa_3113	chr19:22227450-22227532	-	GTTTATGTGACTTTCCTCTT	No	Yes	No	0	0	0	No	
NM_hsa_3114	chr19:32659206-32659303	-	TGAGGTAGGAGGTTCTGTATT	No	Yes	No	0	0	0	Yes	
NM_hsa_3115	chr19:33967952-33968017	-	TGGAGAGACAGGCAGTGGCTG	No	Yes	No	0	0	0	Yes	
NM_hsa_3117	chr19:42112979-42113044	-	CTGGTACAGGCCTGGAGGGT	No	Yes	No	0	0	0	No	
NM_hsa_3118	chr19:45504632-45504706	-	GGCGGCGGGCGGGGGCGGGGCGG	No	Yes	No	0	0.132445	0	Yes	
NM_hsa_3120	chr19:47734457-47734530	-	GCGGGGGCGGGGCGGGGCGGGGC	No	Yes	No	0.0569857	0	0	Yes	
NM_hsa_3121	chr19:49149595-49149692	-	CGGCTGGGAGCCGAGGACCAG	No	Yes	No	0	0	0	Yes	
NM_hsa_3122	chr19:49899167-49899254	-	AAAGGTCCCTGGTTCCTACC	No	Yes	No	0	0	0	No	
NM_hsa_3125	chr1:875614-875697	+	GCGCCGCCGCCCCCCCCCCC	No	Yes	No	0.0854786	0.165556	0.0732862	Yes	
NM_hsa_3126	chr1:1102442-1102513	+	CCCAGCTCGGGCAGCCGTGGC	No	Yes	No	0	0	0	No	
NM_hsa_3130	chr1:1271612-1271709	+	GGCGGGGGGAGCCCCGGGGCG	No	Yes	No	2.16546	2.0529	3.15131	Yes	
NM_hsa_3131	chr1:2158685-2158776	+	GGCCGGCGGGCGGGGCGGGGCC	No	Yes	No	0	0	0	Yes	
NM_hsa_3132	chr1:6268875-6268950	+	TGAGCCCGCGCTCGGCCCGCAG	No	Yes	No	0	0	0	Yes	
NM_hsa_3133	chr1:6685748-6685830	+	CAGGGTCGCGGGAGGACGCTGGA	No	Yes	No	0	0	0	No	
NM_hsa_3136	chr1:9371339-9371424	+	TGATGGGTCTCGGCGTGGCTGA	No	Yes	No	0	0.0662225	0.0366431	No	
NM_hsa_3137	chr1:9750281-9750352	+	CTTCCTCCCCGGGGGAGCGCC	No	Yes	No	2.84929	2.84757	0.732862	No	KapB+cMyc>KapB>H
NM_hsa_3141	chr1:10571690-10571772	+	TGTGGACCCAGGGACCTCTGA	No	Yes	No	0.284929	0.860893	0.146572	Yes	
NM_hsa_3142	chr1:10684310-10684404	+	CGCCGTGACTGCTTTCTCCTCGC	No	Yes	No	0	0.0662225	0	Yes	
NM_hsa_3143	chr1:10688264-10688337	+	ACTGAAAGTCCAGTGTCCAGG	No	Yes	No	0	0	0	No	
NM_hsa_3144	chr1:12251751-12251851	+	TGCCGCCCTCTCGCTGCTCTAGA	No	Yes	Yes	0	0	0	No	
NM_hsa_3144	chr1:12251760-12251841	+	TGCCGCCCTCTCGCTGCTCTAGA	No	Yes	Yes	0	0	0	No	
NM_hsa_3145	chr1:19229446-19229530	+	TTCGATGGGAGTCCGACTGA	No	Yes	No	0	0	0	No	
NM_hsa_3146	chr1:19243614-19243690	+	ACAGGATGATGAGCGTGACTGGG	No	Yes	No	0	0	0	No	
NM_hsa_3147	chr1:21672033-21672107	+	GGCGGGGCCGCCGCGGGGCC	No	Yes	No	0	0	0	No	
NM_hsa_3148	chr1:23702353-23702432	+	GCATGAGCCACTGTGCCTGACA	No	Yes	No	0	0.0662225	0	No	
NM_hsa_3149	chr1:25174649-25174743	+	AGATCTGTCAGGGCTGCTGCG	No	Yes	No	0	0	0	No	
NM_hsa_3150	chr1:26881016-26881096	+	CCTCCTGCCCTCCTTGCTGTAG	No	Yes	No	0.0854786	0.0331113	0	Yes	KapB+cMyc>KapB>H
NM_hsa_3152	chr1:28833867-28833937	+	TCCAACGTGGATACACCCGG	No	Yes	No	0.0569857	0.596003	0.513004	Yes	
NM_hsa_3154	chr1:31381667-31381758	+	AGGAGGGGCCCGGGGCGGGG	No	Yes	No	0.455886	0.331113	0.0732862	Yes	KapB+cMyc>KapB>H
NM_hsa_3155	chr1:32686769-32686853	+	AGTGTGATGGCCTGACTGAG	No	Yes	No	0	0	0	No	
NM_hsa_3156	chr1:32817165-32817271	+	GCGCCGTAGCAGCTGGGCCAG	No	Yes	Yes	0	0	0	No	
NM_hsa_3156	chr1:32817174-32817256	+	GCGCCGTAGCAGCTGGGCCAG	No	Yes	Yes	0	0	0	No	
NM_hsa_3160	chr1:35545055-35545146	+	GTCGGTCGGAGGGAGGATGA	No	Yes	No	0	0	0	No	
NM_hsa_3162	chr1:40505743-40505825	+	GACTGCCAGCCGGGCTGGGG	No	Yes	No	0	0	0	No	
NM_hsa_3163	chr1:41220011-41220095	+	CTTTCTGGGCAGTCTTTGCTAC	No	Yes	No	0	0	0.219859	No	
NM_hsa_3166	chr1:41283765-41283852	+	CTGTGACCTGGGCCCCAGTTCT	No	Yes	No	0	0	0	No	
NM_hsa_3167	chr1:41931843-41931931	+	CTGCTTACTCCAGGTGCTGTG	No	Yes	No	0	0	0	No	
NM_hsa_3168	chr1:42801097-42801167	+	CAGTTCCGAGCGACTTGGGG	No	Yes	No	0	0	0	No	
NM_hsa_3172	chr1:43997256-43997335	+	TGCGGTCCCGGGCTGCGCCGG	No	Yes	Yes	0	0	0	No	
NM_hsa_3172	chr1:43997244-43997350	+	TGCGGTCCCGGGCTGCGCCGG	No	Yes	Yes	0	0	0	No	
NM_hsa_3174	chr1:44066129-44066210	+	CTCAGGACTGGGTCATTCTGTT	No	Yes	Yes	0.0569857	0	0.0732862	Yes	
NM_hsa_3174	chr1:44066099-44066237	+	CTCAGGACTGGGTCATTCTGTT	No	Yes	Yes	0.0569857	0	0.0732862	Yes	
NM_hsa_3176	chr1:46856415-46856501	+	GGAGGTCCCTTCGTGGTCGCCA	No	Yes	No	18.6343	15.165	23.4516	Yes	
NM_hsa_3177	chr1:47908900-47908987	+	TAGGTTCCGTCTGTGCTTGG	No	Yes	No	0	0	0	No	
NM_hsa_3178	chr1:49378415-49378494	+	CTCTTCAACCTAGTGCCTTCC	No	Yes	No	0	0	0	No	
NM_hsa_3180	chr1:54594286-54594370	+	CATTAGACAGTGAGCTCCTGG	No	Yes	No	0	0	0	No	
NM_hsa_3181	chr1:61516056-61516146	+	CCGCTTCGCCGGCAGCTGTAG	No	Yes	No	0	0	0	No	
NM_hsa_3185	chr1:67529202-67529298	+	TCAGACCTTTGAAGAGAGAG	No	Yes	No	0.227943	0.26489	0	Yes	
NM_hsa_3189	chr1:90306760-90306854	+	AGACTGCGAGTGCCTAGTGG	No	Yes	No	0	0	0	Yes	
NM_hsa_3191	chr1:96079530-96079609	+	GCGCGCGCGCGTGTGTGGTGTG	No	Yes	No	0.113971	0.0551854	0.329788	Yes	
NM_hsa_3192	chr1:108742995-108743089	+	GAGCTGGGTCTCGGGAGGTG	No	Yes	No	0	0	0	No	
NM_hsa_3193	chr1:109814148-109814223	+	AGGGAGGCACCTGGGCTGTGGA	No	Yes	No	0	0	0	No	
NM_hsa_3196	chr1:110769823-110769897	+	TGTGATGCCTCTGTCCCTCTGA	No	Yes	No	0	0	0	No	
NM_hsa_3197	chr1:110773305-110773396	+	CCCAGATAGCAGGAGGGCTGAG	No	Yes	No	0	0	0	No	
NM_hsa_3198	chr1:111742964-111743063	+	CAGTCGGCGTTGGAAGAGCCTGA	No	Yes	No	0.0569857	0	0	No	
NM_hsa_3199	chr1:112162347-112162425	+	TCTGGAGGAGGCGCCGCCGC	No	Yes	No	7.23719	7.48314	28.0686	Yes	H>KapB>KapB+cMyc
NM_hsa_3200	chr1:113162478-113162575	+	GTGTCGGATGAGGAGAAGGTAA	No	Yes	No	0	0	0	No	
NM_hsa_3201	chr1:117082050-117082138	+	GTGCCACATGCAGGGTTTGT	No	Yes	No	0	0	0	No	
NM_hsa_3203	chr1:120165844-120165941	+	GGCCGCAGCGCCGCCGCCCCC	No	Yes	No	0	0	0	No	
NM_hsa_3204	chr1:120190942-120191034	+	GCCCTGGAGGTAGCTCGGGCCAG	No	Yes	No	0	0	0	No	
NM_hsa_3206	chr1:143910905-143910972	+	ACAATGAATCACAGAACTTTGT	No	Yes	No	0	0	0	Yes	
NM_hsa_3208	chr1:145726408-145726493	+	TGGGATTGCGATGAACTCAGGT	No	Yes	No	0	0	0	Yes	
NM_hsa_3210	chr1:149832910-149832987	+	TCCGGATTCTGTAGTGGCTTT	No	Yes	No	0	0	0	No	
NM_hsa_3211	chr1:150039001-150039070	+	CCCGCCGGAGTTAGAGCTGG	No	Yes	No	0	0	0	No	
NM_hsa_3212	chr1:150316568-150316648	+	TTGTCCTCTCTTCACTGCAGT	No	Yes	No	0	0	0	Yes	
NM_hsa_3214	chr1:151512267-151512353	+	CTGAGTGAGCTGGACCTTGG	No	Yes	No	0	0	0	No	
NM_hsa_3215	chr1:152286115-152286207	+	CACGATCGCTGACTGCAGATGA	No	Yes	No	0	0	0	No	
NM_hsa_3216	chr1:153508714-153508844	+	TCACCCCTGCTTCCTCTGCCAGA	No	Yes	Yes	0.170957	0.397335	0.0732862	Yes	
NM_hsa_3216	chr1:153508740-153508819	+	TCACCCCTGCTTCCTCTGCCAGA	No	Yes	Yes	0.170957	0.397335	0.0732862	Yes	
NM_hsa_3217	chr1:153750862-153750948	+	TACCCAGGTCTCCCTTTCCCCAG	No	Yes	Yes	0	0	0	Yes	
NM_hsa_3217	chr1:153750853-153750963	+	TACCCAGGTCTCCCTTTCCCCAG	No	Yes	Yes	0	0	0	Yes	
NM_hsa_3218	chr1:153939964-153940045	+	CCCGCCGGCCGGACGCAGTGG	No	Yes	No	0	0	0	No	
NM_hsa_3219	chr1:154095036-154095127	+	GTGTGAACTCGGGAGGTGGAGGT	No	Yes	No	0	0	0	Yes	
NM_hsa_3225	chr1:156186286-156186380	+	CTGGCCTACCGGTGACCCGG	No	Yes	No	0	0	0.0732862	Yes	
NM_hsa_3226	chr1:156390138-156390214	+	TTTCGGTTATCTAGCTTTATGA	No	Yes	No	0	0	0	Yes	
NM_hsa_3227	chr1:156426459-156426548	+	CTGGCAAGACCTGGCAAGACC	No	Yes	No	0	0	0	No	
NM_hsa_3228	chr1:156836344-156836436	+	ATGGACCCCTTCCCCCAGCTGT	No	Yes	No	0	0	0	No	
NM_hsa_3230	chr1:158057709-158057804	+	TGGGGAGGCCAGGTGTCATGGAT	No	Yes	No	0	0	0	No	
NM_hsa_3231	chr1:158064019-158064113	+	TCCCACTCCTGCTGCTCCACAGT	No	Yes	No	0	0	0	Yes	
NM_hsa_3233	chr1:158969794-158969881	+	CTTGACTGAGAAACGGACCGGG	No	Yes	No	0	0.132445	0.0732862	No	
NM_hsa_3234	chr1:160387216-160387354	+	TGGCCACCTTCTTGCCTCCAGA	No	Yes	Yes	0	0	0	Yes	
NM_hsa_3234	chr1:160387246-160387327	+	TGGCCACCTTCTTGCCTCCAGA	No	Yes	Yes	0	0	0	Yes	
NM_hsa_3235	chr1:160387246-160387327	+	TGTAGACATTTAGATGGCCCAG	No	Yes	Yes	0	0	0	Yes	
NM_hsa_3235	chr1:160387219-160387357	+	TGTAGACATTTAGATGGCCCAG	No	Yes	Yes	0	0	0	Yes	
NM_hsa_3236	chr1:161179581-161179656	+	TTCCCAGCACGTTCTATGAAG	No	Yes	No	0	0	0	Yes	
NM_hsa_3241	chr1:164604509-164604584	+	CTGCCACCGCTGCCACCATG	No	Yes	No	34.2579	16.2797	12.6907	Yes	KapB+cMyc>KapB>H
NM_hsa_3242	chr1:165325703-165325781	+	CTGGCTCTGCTCCTCGGCGCGC	No	Yes	Yes	0	0	0	No	
NM_hsa_3242	chr1:165325698-165325786	+	CTGGCTCTGCTCCTCGGCGCGC	No	Yes	Yes	0	0	0	No	
NM_hsa_3243	chr1:168212209-168212285	+	AACAAGTGACTCTGCTTTAGA	No	Yes	No	0	0	0	No	
NM_hsa_3244	chr1:169933454-169933540	+	TAAGCCTCGGGCCAGCAATGTGG	No	Yes	No	0	0	0	No	
NM_hsa_3245	chr1:171070869-171070950	+	TCACCCAGATCTGCGGCCTAAT	No	Yes	No	0	0	0	No	
NM_hsa_3246	chr1:171070869-171070950	+	AATAGGCCACGGATCTGGGCAAA	No	Yes	Yes	0	0	0	No	
NM_hsa_3246	chr1:171070860-171070960	+	AATAGGCCACGGATCTGGGCAAA	No	Yes	Yes	0	0	0	No	
NM_hsa_3250	chr1:180170414-180170482	+	ATCCCGGGCCAGGTGTGAGAGG	No	Yes	No	0	0	0	No	
NM_hsa_3251	chr1:182174959-182175046	+	AGAAAGCTGTAGATCCCTGA	No	Yes	No	0	0	0	No	
NM_hsa_3253	chr1:198625944-198626019	+	CAGATGGAGATAAAACTGTGG	No	Yes	No	0	0	0	No	
NM_hsa_3254	chr1:198906766-198906859	+	GTTAAGAAAGGGACTGAAGT	No	Yes	No	0	0	0	No	
NM_hsa_3255	chr1:198988191-198988269	+	CTTGCTGTAGCTTACCTCCT	No	Yes	No	0	0	0	No	
NM_hsa_3256	chr1:199998325-199998424	+	CTTGGGCTCCTGCTGCGCGCAGC	No	Yes	No	2.10847	2.18534	1.6123	Yes	
NM_hsa_3259	chr1:201858494-201858583	+	CCCCCAATCCCTGCCCGCAGT	No	Yes	No	0	0	0	No	
NM_hsa_3260	chr1:204965444-204965524	+	TTAGCTCCCTCTTCCCATGTTC	No	Yes	No	0	0	0	Yes	
NM_hsa_3261	chr1:205082126-205082219	+	TGTGATCGTGCCTGTGACTAGC	No	Yes	No	0	0	0	No	
NM_hsa_3262	chr1:215043890-215043988	+	GCTGCGGTCGGGCGGCGGCGGC	No	Yes	No	1.02574	0.397335	0.293145	Yes	KapB+cMyc>KapB>H
NM_hsa_3263	chr1:216774166-216774245	+	CTCTGGTTGGACGCAGGCTGC	No	Yes	No	0	0	0	No	
NM_hsa_3264	chr1:223242900-223242974	+	CCACTGTGATCAGTTTGTGG	No	Yes	No	0	0	0	No	
NM_hsa_3266	chr1:224415222-224415311	+	TCTCTGCCGGCTTGATGGGCTAG	No	Yes	No	0	0	0	Yes	
NM_hsa_3270	chr1:231558361-231558437	+	CTGCTCTTCCTCCAGGCCGG	No	Yes	No	0	0	0	No	
NM_hsa_3271	chr1:234735989-234736071	+	CGCGGAGACTGCGCTGCTCCAG	No	Yes	No	0	0	0	Yes	
NM_hsa_3272	chr1:235107512-235107628	+	TGAGAACCGCGATTACTTTCA	No	Yes	Yes	0	0	0	No	
NM_hsa_3272	chr1:235107528-235107608	+	TGAGAACCGCGATTACTTTCA	No	Yes	Yes	0	0	0	No	
NM_hsa_3274	chr1:242663809-242663899	+	GTGCCGAACTGTGATAACTGA	No	Yes	No	0	0	0	No	
NM_hsa_3276	chr1:244013765-244013839	+	TCCTCCCGGGCTGCGCCTCGCT	No	Yes	Yes	2.3934	3.31113	1.97873	Yes	
NM_hsa_3276	chr1:244013731-244013869	+	TCCTCCCGGGCTGCGCCTCGCT	No	Yes	Yes	2.3934	3.31113	1.97873	Yes	
NM_hsa_3277	chr1:247580627-247580715	+	ATAGAGCTCTGTGCAGGTTAGA	No	Yes	No	0	0	0	No	
NM_hsa_3278	chr1:249200355-249200453	+	CCCTCTGGTGCCGGACGTTGCG	No	Yes	No	0	0	0	No	
NM_hsa_3279	chr1:566084-566158	-	CAACTTACTGAGGGCTTTGA	No	Yes	No	0	0	0	Yes	
NM_hsa_3280	chr1:1243849-1243943	-	CCCCCCCGGCCCCGGCCGCT	No	Yes	No	0	0.0662225	0.0732862	Yes	H>KapB>KapB+cMyc
NM_hsa_3283	chr1:6663157-6663246	-	CCCCGGTCCCGGCGCGCGGC	No	Yes	No	0	0	0	No	
NM_hsa_3284	chr1:6844946-6845041	-	GTGCTCCCGGGGCTGAAGGA	No	Yes	No	0	0	0	No	
NM_hsa_3288	chr1:11586559-11586652	-	TCAGGCTCCGTCCCCTCCCTC	No	Yes	No	0.379905	0.0662225	0.171001	Yes	
NM_hsa_3289	chr1:21763562-21763634	-	CTGCATGACCAGGGACCTGT	No	Yes	No	0	0	0	No	
NM_hsa_3290	chr1:22175274-22175370	-	TGGTGGAGGGCTGGGGGACC	No	Yes	No	0.227943	0.331113	0.439717	No	H>KapB>KapB+cMyc
NM_hsa_3291	chr1:22183747-22183822	-	TGTGCAGGTCAGGGAGTGGG	No	Yes	No	0	0	0	No	
NM_hsa_3292	chr1:26438064-26438158	-	CGGGAGTCGTCGGAGGTGTG	No	Yes	No	0	0	0	No	
NM_hsa_3293	chr1:27831519-27831609	-	CTGGACTTGGAGTCCAAAGAC	No	Yes	No	0	0	0	Yes	
NM_hsa_3294	chr1:28483695-28483778	-	TGAGGTAGGAGGTTGGCTTG	No	Yes	No	0	0	0	Yes	
NM_hsa_3295	chr1:33177935-33178017	-	GGGCGCGGGGTCGGCGTGGCGGC	No	Yes	No	0	0	0	No	
NM_hsa_3296	chr1:35893406-35893490	-	TTGTGGGTTATTGTTAAGCTGAT	No	Yes	No	14.4744	10.3307	3.81089	Yes	KapB+cMyc>KapB>H
NM_hsa_3298	chr1:38261819-38261912	-	AGAGTTGAGTCTGCAGGTCCCT	No	Yes	No	0	0	0	No	
NM_hsa_3299	chr1:41847284-41847364	-	TGCAGCGACAGGTTGTGCCGGAT	No	Yes	No	0.341914	0.26489	0.219859	Yes	KapB+cMyc>KapB>H
NM_hsa_3301	chr1:44466717-44466788	-	TTGGGGAGCCCGGGCTGCAG	No	Yes	No	0	0	0	No	
NM_hsa_3302	chr1:45196504-45196600	-	TTAGACTAGACTGTAGTGAG	No	Yes	No	0	0	0	No	
NM_hsa_3304	chr1:45797218-45797292	-	TGGAGGGAATCGGCAGCTGAG	No	Yes	No	0	0	0	No	
NM_hsa_3305	chr1:47579102-47579196	-	AAGGAGCTCACAGTCCATTGGAA	No	Yes	No	9.13671	8.5632	7.39842	Yes	KapB+cMyc>KapB>H

Additional file 5

Name	locus	strand	sequence	mirdeep2	mireap	miranalyzer	RPM_HU_KapB_cMyc	RPM_HU_KaposinB	RPM_HUVEC	IP_confidence	annotation
NM_hsa_3306	chr1:47799494-47799574	-	GGGCGGGGCGGGGAGGCGGCG	No	Yes	No	0	0	0	No	
NM_hsa_3308	chr1:60809985-60810083	-	CACTAGTTTGTGAGCTCCTTAA	No	Yes	No	33.4858	29.5816	11.9017	Yes	KapB+cMyc>KapB>H
NM_hsa_3311	chr1:74272637-74272710	-	GTTTCCATAGTGTAGTGGTT	No	Yes	No	1.25369	0.79467	0.806149	Yes	
NM_hsa_3312	chr1:77578428-77578500	-	TTGGTTCTCTAGCTGTATAT	No	Yes	No	0	0	0	No	
NM_hsa_3313	chr1:85345214-85345303	-	CGAGGTGGGTGGATCACGAGGCC	No	Yes	No	0	0.26489	0	No	
NM_hsa_3315	chr1:104314745-104314827	-	ATACCTACTTATCTATCAACAC	No	Yes	No	0	0	0	Yes	
NM_hsa_3316	chr1:117210401-117210485	-	GGCGGGGCCGGGGGCGGGGCCG	No	Yes	No	0	0	0.0732862	Yes	
NM_hsa_3317	chr1:120114212-120114292	-	AGACTGGGTTGACTCTGGAG	No	Yes	No	0	0	0	No	
NM_hsa_3319	chr1:142667290-142667361	-	ATTTTCATAATGCAGTCACATA	No	Yes	No	0	0	0	Yes	
NM_hsa_3320	chr1:145195999-145196081	-	CTGTGGACTGGAGAGTTATG	No	Yes	No	0	0	0	No	
NM_hsa_3321	chr1:145379281-145379353	-	AGAGTTCAAGTCTCACTGGGA	No	Yes	No	0	0.0331113	0	Yes	
NM_hsa_3322	chr1:146082680-146082775	-	CCGGAAGTCAGACTATGGGG	No	Yes	No	0	0	0	No	
NM_hsa_3323	chr1:147803755-147803834	-	ACACTCATCAACTGTGCCTGA	No	Yes	No	0	0	0	No	
NM_hsa_3324	chr1:149937797-149937881	-	TTCAGAAATAGGAAGGTGGAA	No	Yes	No	0	0	0	Yes	
NM_hsa_3325	chr1:153278768-153278852	-	GAGAGGGAAAGAGAGAGTGGG	No	Yes	No	0	0	0.0732862	Yes	
NM_hsa_3331	chr1:160013109-160013198	-	TGGGGGTAGAGCAGAGAATG	No	Yes	No	0	0	0	No	
NM_hsa_3332	chr1:167153912-167153981	-	TGAGGTAGTAGGTTGGGAGT	No	Yes	No	10.2448	7.33598	1.62858	Yes	KapB+cMyc>KapB>H
NM_hsa_3336	chr1:173532545-173532614	-	AAAAGCTGGGTTGAGGGAGC	No	Yes	No	0.00518052	0.147161	0.0162858	Yes	
NM_hsa_3341	chr1:206136822-206136912	-	CTTCCTCCAGCCGCGGGAAGTGG	No	Yes	No	0	0	0	No	
NM_hsa_3342	chr1:207693035-207693100	-	GCAGCTGGGCTGGAGTCTCTGC	No	Yes	No	0	0	0	No	
NM_hsa_3345	chr1:210168948-210169029	-	TCTGTAGTTGTTGGGTGGAGT	No	Yes	No	0	0	0	No	
NM_hsa_3350	chr1:225615430-225615514	-	GGTCGGGGCGGCGGCGGGCGA	No	Yes	No	0	0.0220742	0.0244288	Yes	H>KapB>KapB+cMyc
NM_hsa_3353	chr1:233126337-233126428	-	CTCTGAGGTGCTGGGCTGGG	No	Yes	No	0	0	0	No	
NM_hsa_3354	chr1:237227164-237227252	-	GGGGTGGGGTCGGGGGTGGA	No	Yes	No	0	0	0	No	
NM_hsa_3355	chr1:240910395-240910463	-	TCTGGTAGTAGGTTTTGAGA	No	Yes	No	0	0	0	No	
NM_hsa_3356	chr20:611399-611496	+	CTAGCCAGTGAGCTCCTGGAG	No	Yes	No	0	0	0	No	
NM_hsa_3359	chr20:3194751-3194835	+	CAAAATGATGAGGTACCTGATA	No	Yes	No	3.64709	10.132	12.1655	No	H>KapB>KapB+cMyc
NM_hsa_3363	chr20:4527579-4527675	+	TAGCACAGTGAGTAATAATG	No	Yes	No	0	0	0	No	
NM_hsa_3365	chr20:13909706-13909788	+	TGGAACTGAGGCTTCTCTGAG	No	Yes	No	0	0	0	No	
NM_hsa_3366	chr20:17486149-17486232	+	TGCCCGGCGGTGTGCGGCCACA	No	Yes	No	0.911772	0.132445	0.58629	Yes	
NM_hsa_3367	chr20:17662487-17662571	+	GCGGCGGGCGGCGGGCGGGCCG	No	Yes	No	0.0854786	0.18763	0.0366431	Yes	
NM_hsa_3368	chr20:19739049-19739113	+	GGGTTCCAGACGTGGGTGCTG	No	Yes	No	0	0	0	No	
NM_hsa_3371	chr20:21503232-21503323	+	GCGGGCGGTCGGGCGGCGGC	No	Yes	No	2.87778	2.31779	0.769506	No	KapB+cMyc>KapB>H
NM_hsa_3372	chr20:23342853-23342950	+	GCCGGCTGGGAGGTGAGTGCG	No	Yes	No	0	0	0	No	
NM_hsa_3373	chr20:25262596-25262678	+	TCCCTGTTCTCTGTGTTTGCAG	No	Yes	No	0	0.0662225	0	No	
NM_hsa_3374	chr20:30194978-30195067	+	CTCCTGCGTAGGATCTGAGGA	No	Yes	No	0.0284929	0.0772596	0	Yes	
NM_hsa_3377	chr20:35374639-35374735	+	CAGATTTTGGAGGCGCTGGG	No	Yes	No	0	0	0	No	
NM_hsa_3378	chr20:36602737-36602813	+	CATGAATAGGGTTTAAAAAAA	No	Yes	No	0	0	0	No	
NM_hsa_3381	chr20:44563720-44563800	+	TGGGCCGGCGCGCGCGCGCGC	No	Yes	No	0.0284929	0	0	No	
NM_hsa_3384	chr20:47250126-47250208	+	AAGCCCCCAGGTGCTGCTGGT	No	Yes	No	0	0	0	No	
NM_hsa_3385	chr20:57227577-57227666	+	TGGTCCTAAGGGCTGGGTCG	No	Yes	No	0	0	0	No	
NM_hsa_3390	chr20:60962291-60962386	+	GCGCCGCGCGGTGACTCCCCAG	No	Yes	No	0	0	0	Yes	
NM_hsa_3394	chr20:61918196-61918278	+	AGGTCGGGGGCCAGGGGACAGGA	No	Yes	Yes	0.170957	0	0.0732862	Yes	
NM_hsa_3394	chr20:61918186-61918286	+	AGGTCGGGGGCCAGGGGACAGGA	No	Yes	Yes	0.170957	0	0.0732862	Yes	
NM_hsa_3395	chr20:62711083-62711168	+	GCTCCCGAAGCTCCCGCCCAG	No	Yes	No	0	0	0	No	
NM_hsa_3398	chr20:23078418-23078499	-	TTTGCCAGATCCTTGCACGG	No	Yes	No	0	0	0	No	
NM_hsa_3400	chr20:26189887-26189969	-	GTGCTCCCCTCTGGCGGGTG	No	Yes	No	0	0	0	No	
NM_hsa_3402	chr20:44151625-44151707	-	TGTCAGTTGGTCACATACCCAA	No	Yes	No	0	0	0	No	
NM_hsa_3403	chr20:54606257-54606348	-	TGAGGTAGTAGTTTGTGTCTT	No	Yes	No	325.458	293.394	147.771	Yes	KapB+cMyc>KapB>H
NM_hsa_3405	chr20:59177120-59177204	-	TCTTTGGTTAGCTAGATGGA	No	Yes	No	0	0	0	No	
NM_hsa_3406	chr20:61201246-61201328	-	GACCGCGTGGCCTGCTGGAA	No	Yes	No	0	0	0	No	
NM_hsa_3407	chr20:61809854-61809937	-	CAAGGTCCGCTGTGAACACGCA	No	Yes	No	0	0	0	No	
NM_hsa_3408	chr20:61999828-61999925	-	CTCCTGACTCGAGGTCCCGTGG	No	Yes	No	0.0189952	0	0.0244288	Yes	
NM_hsa_3409	chr21:10906339-10906422	+	CGGAATGAAAGGGAGTGTGTG	No	Yes	No	0	0	0	No	
NM_hsa_3410	chr21:15399757-15399824	+	TCAGCCAGAGAGCGGGGTTAG	No	Yes	No	0	0	0	No	
NM_hsa_3419	chr21:30375146-30375234	+	CTGCGAGTGCTCGGGAGCTGC	No	Yes	No	0	0	0	No	
NM_hsa_3421	chr21:34103288-34103363	+	TCAGGATCAGGATCTGCAGAGT	No	Yes	No	0	0	0	Yes	
NM_hsa_3423	chr21:34852454-34852530	+	CCTCCCGGAGCCGGAGTTCAGA	No	Yes	No	0	0	0	No	
NM_hsa_3424	chr21:38071426-38071511	+	CTCCGGCGCTGCGTCTCCAGGC	No	Yes	No	0	0.198668	0	Yes	
NM_hsa_3425	chr21:38639949-38640017	+	CTGTCTGTCGGTTGCAGCCTG	No	Yes	No	0	0	0	No	
NM_hsa_3426	chr21:41935293-41935373	+	CTCAGAGTTCCACCAGATCAA	No	Yes	No	0	0	0	No	
NM_hsa_3430	chr21:45742814-45742907	+	CGGATGTGTCTTTGACTGCAG	No	Yes	No	0	0	0	No	
NM_hsa_3431	chr21:46301281-46301367	+	GTCACCTGCCAGGGATTTCC	No	Yes	No	0	0	0	No	
NM_hsa_3435	chr21:41935293-41935367	-	TGGTGGAACTCTGAGAATTTC	No	Yes	No	0	0	0	No	
NM_hsa_3436	chr21:43243805-43243893	-	CCCAGAGGAGCCTGATGGAGA	No	Yes	No	0	0	0	No	
NM_hsa_3437	chr22:19951658-19951732	+	GGCACTGACAGGCGCTGTTCCAG	No	Yes	No	0.0569857	0	0	No	
NM_hsa_3442	chr22:22090411-22090488	+	CGCGCTCGGACGCAGGCTGG	No	Yes	No	0	0	0	No	
NM_hsa_3443	chr22:23267723-23267805	+	GGGCTCTGGGTCTGGGGCTG	No	Yes	No	0	0	0	No	
NM_hsa_3445	chr22:24237106-24237202	+	CGCGCGGCCAGGCCCGGGACTGA	No	Yes	No	0.227943	0.0662225	0	No	KapB+cMyc>KapB>H
NM_hsa_3446	chr22:24303502-24303593	+	ACTCCACCACCAGATCCGTG	No	Yes	No	0	0	0	No	
NM_hsa_3447	chr22:24577371-24577461	+	TGTCTGGCCGCCTCGCCTCGGA	No	Yes	No	0	0	0	No	
NM_hsa_3448	chr22:24583848-24583937	+	CTGCACTCTGTCCCCATCCCAGT	No	Yes	Yes	0.170957	0.331113	0.659576	No	H>KapB>KapB+cMyc
NM_hsa_3448	chr22:24583827-24583967	+	CTGCACTCTGTCCCCATCCCAGT	No	Yes	Yes	0.170957	0.331113	0.659576	No	H>KapB>KapB+cMyc
NM_hsa_3450	chr22:26725720-26725813	+	AATGTTTTTTCTCTGACCACC	No	Yes	No	0.113971	1.25823	0.659576	No	
NM_hsa_3454	chr22:31284134-31284219	+	AAGCTCTGTCTGTGTCTCTAG	No	Yes	No	0	0	0	Yes	
NM_hsa_3455	chr22:31742836-31742911	+	CAGGTCGAGCGGGCGTCGTC	No	Yes	No	0	0	0	No	
NM_hsa_3458	chr22:36784390-36784486	+	ACTGGGAAGGTCTGGAACTCT	No	Yes	No	0	0	0	No	
NM_hsa_3459	chr22:38012864-38012949	+	TAGGGTGACCGGGACTCCGTGG	No	Yes	No	0	0	0.219859	No	
NM_hsa_3462	chr22:39638464-39638557	+	GCCGAGCCCCGCGTCCTCCC	No	Yes	No	0	0	0	No	
NM_hsa_3464	chr22:39917748-39917818	+	ATCACTCAAATGTTTTGCAG	No	Yes	No	0	0	0	No	
NM_hsa_3465	chr22:40706252-40706335	+	TTGCAGATGCTTTTCCCTCCAAT	No	Yes	No	0	0	0	Yes	
NM_hsa_3468	chr22:41543902-41543981	+	GTTAGTTTGACGTCTTTGGTA	No	Yes	No	0	0	0	No	
NM_hsa_3475	chr22:46984162-46984248	+	CCGTCCCTGTCTGGCTGTCCGT	No	Yes	No	0	0.0993338	0	No	
NM_hsa_3476	chr22:49483780-49483848	+	TCACGCGGGGCCCGGGGCGG	No	Yes	No	0	0	0	No	
NM_hsa_3479	chr22:50913603-50913682	+	CCTCGGGCCGGGCACCTGGAC	No	Yes	No	0	0	0	No	
NM_hsa_3480	chr22:50969225-50969294	+	TTGTAGACCCCTGGACTCACGA	No	Yes	No	0	0	0	No	
NM_hsa_3481	chr22:51066716-51066804	+	CTTGAAACGGGCTCTGGGAG	No	Yes	No	0	0	0	No	
NM_hsa_3482	chr22:17082682-17082764	-	GGGAGCGGGCGGGCGGGCGGC	No	Yes	No	0.0284929	0	0	Yes	
NM_hsa_3484	chr22:20786266-20786339	-	TGTCCTCCCTCTGCTCGCAG	No	Yes	Yes	0	0	0	No	
NM_hsa_3484	chr22:20786241-20786365	-	TGTCCTCCCTCTGCTCGCAG	No	Yes	Yes	0	0	0	No	
NM_hsa_3485	chr22:21871756-21871829	-	CGCTGGGCGGGCGGCTGTGAG	No	Yes	No	0	0	0	No	
NM_hsa_3486	chr22:23089530-23089608	-	TTGGCCATGGGGCTGAGCTGGGCT	No	Yes	No	0	0	0	Yes	
NM_hsa_3487	chr22:24407605-24407699	-	GGCGGCGCGCGCGCGCGTGCGC	No	Yes	No	0.0569857	0	0.146572	Yes	
NM_hsa_3491	chr22:39711178-39711250	-	ACTGCCTTCCTTCTGAGAACA	No	Yes	No	0.341914	0.26489	0.0732862	Yes	KapB+cMyc>KapB>H
NM_hsa_3492	chr22:39715026-39715103	-	CGGACAGAAACTGTGTGCTGA	No	Yes	No	0	0	0	No	
NM_hsa_3494	chr22:42519490-42519567	-	AAGGAGAGAGAACAGGCTGAGG	No	Yes	No	0.284929	0.231779	0.146572	Yes	KapB+cMyc>KapB>H
NM_hsa_3495	chr22:42531647-42531723	-	AAGGAGAGAGAACAGGCTGAG	No	Yes	No	0	0.0331113	0	Yes	
NM_hsa_3496	chr22:42739544-42739640	-	GGACGCGGCGGCCGCGGGGCG	No	Yes	No	0	0	0	No	
NM_hsa_3498	chr22:46508621-46508711	-	CTATACAACCTACTACCTCAC	No	Yes	No	0	0.0220742	0.097715	Yes	H>KapB>KapB+cMyc
NM_hsa_3499	chr22:48972588-48972680	-	CCCCGACCCCGCGCCCTGCC	No	Yes	No	0	0	0	No	
NM_hsa_3500	chr2:271694-271784	+	TACCCTCACTGGTTGGACAAAGG	No	Yes	No	0	0	0	Yes	
NM_hsa_3501	chr2:987353-987448	+	GTTCAGGTTTCCCAGGAATGA	No	Yes	No	0	0	0	No	
NM_hsa_3502	chr2:5832525-5832617	+	CTGGGAGAGCTCGGGGTCCGG	No	Yes	No	0	0	0	No	
NM_hsa_3503	chr2:6114782-6114890	+	CTGAACTAGAGATTGGGCCCAG	No	Yes	Yes	0	0	0	No	
NM_hsa_3503	chr2:6114781-6114875	+	CTGAACTAGAGATTGGGCCCAG	No	Yes	Yes	0	0	0	No	
NM_hsa_3504	chr2:8821291-8821359	+	CTGTGAGAGCTCCTTAGCGAC	No	Yes	No	0	0	0	No	
NM_hsa_3505	chr2:9695180-9695272	+	TTTCTGGCTCTGGTAGGAACAC	No	Yes	No	0.3989	0.198668	0.219859	Yes	
NM_hsa_3507	chr2:12481145-12481218	+	CAGCCTTTTACAGGGATATGA	No	Yes	No	0	0	0	No	
NM_hsa_3508	chr2:20189913-20190004	+	GACGTCGATGGGTTTGTGGG	No	Yes	No	0	0	0	No	
NM_hsa_3509	chr2:21151731-21151808	+	CAAAAACTATAGTTACTTTTGC	No	Yes	No	0	0	0	Yes	
NM_hsa_3512	chr2:24714124-24714210	+	CTGTTCTTCAGGCCGGGCGAG	No	Yes	No	0	0	0	No	
NM_hsa_3513	chr2:27456412-27456504	+	TAAGCTCCCTGCCTCCTGTAGT	No	Yes	Yes	0	0	0	Yes	
NM_hsa_3513	chr2:27456396-27456534	+	TAAGCTCCCTGCCTCCTGTAGT	No	Yes	Yes	0	0	0	Yes	
NM_hsa_3515	chr2:28019397-28019480	+	TGACACTTCTGGGTTCTGAGT	No	Yes	No	0	0	0	No	
NM_hsa_3517	chr2:28624798-28624893	+	CTGTGCAACTCTCCTCTTGA	No	Yes	No	0	0	0	No	
NM_hsa_3519	chr2:34133267-34133351	+	GTGCAATGATGATGATCTGAGCC	No	Yes	No	0	0.132445	0	No	
NM_hsa_3520	chr2:42068451-42068540	+	TGCAGTCCGGATGGAGCTCGGT	No	Yes	No	0	0	0	No	
NM_hsa_3521	chr2:43036472-43036551	+	GTGGGCACTCGGGAGGTGGC	No	Yes	No	0	0	0	No	
NM_hsa_3522	chr2:43386219-43386297	+	AGAAGTTGGCAGTGACTCTG	No	Yes	No	0	0	0	No	
NM_hsa_3523	chr2:43386294-43386381	+	CCGGATGGCTCAGGGCTGTGG	No	Yes	No	0	0	0	No	
NM_hsa_3524	chr2:43396727-43396815	+	CGGATCAGAGGGTTGGAGATA	No	Yes	No	0	0	0	No	
NM_hsa_3525	chr2:47572303-47572382	+	AAGAGCTGTAGAGTCTAGCC	No	Yes	No	0	0	0	No	
NM_hsa_3528	chr2:56216106-56216190	+	ATAATCCCAGAGACCACTGTGA	No	Yes	No	0	0	0	No	
NM_hsa_3530	chr2:64977977-64978061	+	GGGCGAGCGGCGGCGGGCGGC	No	Yes	No	0	0	0	No	
NM_hsa_3531	chr2:69053036-69053128	+	TTCTTTTCTTTCCTTCCTTGTAG	No	Yes	No	0	0	0	Yes	
NM_hsa_3534	chr2:70370016-70370102	+	GCCCCAGCCAGGCGCGGTCGG	No	Yes	No	0	0	0	No	
NM_hsa_3536	chr2:73246462-73246545	+	GCCGAAGTAGCTCAGTTGGGAG	No	Yes	No	48.9222	48.5742	11.396	Yes	KapB+cMyc>KapB>H
NM_hsa_3539	chr2:79476084-79476180	+	AGGCTTTGGAGTCAGACAGT	No	Yes	No	0	0	0.146572	No	
NM_hsa_3541	chr2:85788728-85788817	+	ATCTCCAGGGGCATTGTTAGA	No	Yes	No	0	0	0	No	
NM_hsa_3543	chr2:86422608-86422703	+	CTCTTAGAGGGTGGGCTGTG	No	Yes	No	0	0	0	No	
NM_hsa_3544	chr2:87141955-87142032	+	TGGCCGGGCGGCGGCGGCGGCGGC	No	Yes	No	0.745855	0.873784	0.262873	Yes	
NM_hsa_3546	chr2:88749435-88749533	+	TCTGAGACTGGGTCTCCCTCTG	No	Yes	No	0	0	0	Yes	
NM_hsa_3547	chr2:88927783-88927873	+	TCTGACTCTGACCTGGCACG	No	Yes	No	0	0	0.0732862	No	
NM_hsa_3548	chr2:89953077-89953165	+	TTGCCAGGCGAGTCAGGACA	No	Yes	No	0	0	0	No	
NM_hsa_3549	chr2:95691299-95691395	+	GGGGCGGGGTTGGGCGGCGG	No	Yes	No	0	0	0	No	
NM_hsa_3550	chr2:95712290-95712373	+	GGCGAGGAGCGGGGCTGGGC	No	Yes	No	0	0	0	No	
NM_hsa_3551	chr2:95967814-95967908	+	CCAGTGACAGCAAGCAGTGCT	No	Yes	No	0	0	0	Yes	
NM_hsa_3553	chr2:99347744-99347850	+	GCGGAGCGTTCGGGAGGCTGG	No	Yes	Yes	0	0	0	No	
NM_hsa_3553	chr2:99347759-99347840	+	GCGGAGCGTTCGGGAGGCTGG	No	Yes	Yes	0	0	0	No	
NM_hsa_3554	chr2:99347759-99347840	+	AGGTCCGGAGCGGAGCCGCGG	No	Yes	No	0	0	0	No	
NM_hsa_3555	chr2:100106808-100106881	+	GCGCTTCGTTGACCGGGTCG	No	Yes	No	0	0	0	No	
NM_hsa_3556	chr2:101619083-101619167	+	TCTGAGCCTCCTTGTCGCCTGC	No	Yes	No	0.626843	0.331113	1.09929	Yes	
NM_hsa_3557	chr2:102244446-102244519	+	TGGTCTTTGACCAGGGTCTG	No	Yes	No	0	0	0	No	
NM_hsa_3559	chr2:105946291-105946360	+	CCTGCTCCGGGCCTCGGCGTC	No	Yes	No	0	0	0	Yes	
NM_hsa_3560	chr2:111634134-111634231	+	ACAGCCAGGAGTCAGGACAGA	No	Yes	No	0	0	0	No	
NM_hsa_3562	chr2:114514335-114514414	+	CTCTGGTCGCCGAGCGAGGG	No	Yes	No	0	0	0	No	
NM_hsa_3563	chr2:123382882-123382964	+	TGGGTTAGGATGTTGTATAG	No	Yes	No	0	0	0	No	
NM_hsa_3564	chr2:131035881-131035955	+	CTCAAAAAGGATTAGACTGAAC	No	Yes	No	0	0	0	Yes	
NM_hsa_3567	chr2:140049605-140049689	+	AGTAGAAATGCAGAACCTGATT	No	Yes	No	0	0	0	No	
NM_hsa_3568	chr2:140049586-140049714	+	TCAGGTTCTACAATTCTACCAG	No	Yes	Yes	0	0	0	No	
NM_hsa_3568	chr2:140049605-140049689	+	TCAGGTTCTACAATTCTACCAG	No	Yes	Yes	0	0	0	No	
NM_hsa_3570	chr2:157191309-157191396	+	CTTGATCCCCGCGGTGGGCCAG	No	Yes	No	0	0	0	No	
NM_hsa_3573	chr2:168125824-168125894	+	TTTGGCTCTGTGTCTTCACTGA	No	Yes	No	0	0	0	Yes	
NM_hsa_3574	chr2:173263368-173263450	+	AGTACTTCCTAGCTGGCTCTGA	No	Yes	No	0	0.0662225	0	No	
NM_hsa_3576	chr2:174887553-174887640	+	TGCTTTAGGTAGGAGGCTGTTGA	No	Yes	No	0	0	0	Yes	
NM_hsa_3577	chr2:175464717-175464814	+	CTCTGAGAATCTAATGCGCAG	No	Yes	No	0	0	0	No	
NM_hsa_3580	chr2:177959755-177959840	+	ATCGCAGCCTTTGGAGTAGA	No	Yes	No	0	0	0	No	
NM_hsa_3581	chr2:178059701-178059783	+	GGCTGGTCCGAAGGAAGTGAGTT	No	Yes	No	2.67833	1.85423	3.07802	Yes	

Additional file 5

Name	locus	strand	sequence	mirdeep2	mireap	miranalyzer	RPM_HU_KapB_cMyc	RPM_HU_KaposinB	RPM_HUVEC	IP_confidence	annotation
NM_hsa_3583	chr2:180999601-180999675	+	TCTTTGTGTATCTAGCAGTAT	No	Yes	No	0	0	0	No	
NM_hsa_3586	chr2:187541216-187541302	+	GAGGCTGAAGCATGGAGGTTGC	No	Yes	No	0	0	0	Yes	
NM_hsa_3589	chr2:191879017-191879102	+	GCCGCCGGCTGGGTGAGCTG	No	Yes	No	0	0	0	No	
NM_hsa_3590	chr2:191885284-191885382	+	ACGCTCGCCCTGGGGCTGCAGA	No	Yes	No	0	0	0	No	
NM_hsa_3591	chr2:196522756-196522821	+	CTTTGTTCCGGAAAGCCCTT	No	Yes	No	0	0	0	No	
NM_hsa_3592	chr2:198318719-198318797	+	AGAAGGGTTGCCGGCTGCTGG	No	Yes	No	0.0569857	0.132445	0	No	
NM_hsa_3593	chr2:202563846-202563929	+	AGTGCTCAGTGGGGAGAGCATTTG	No	Yes	No	0	0	0	Yes	
NM_hsa_3594	chr2:204126219-204126316	+	ACAGGATGCAGTGCTCAGTGA	No	Yes	No	0	0.26489	0.146572	Yes	
NM_hsa_3595	chr2:204126219-204126316	+	CACTGCGCACTGCATCCTGTAG	No	Yes	No	0.170957	0	0	Yes	
NM_hsa_3599	chr2:218905708-218905775	+	CTTTCAGATGGTTCTCTAGAG	No	Yes	No	0	0	0	No	
NM_hsa_3600	chr2:218905708-218905775	+	CTCTAGAGAACCATCTGAAAG	No	Yes	No	0	0	0	No	
NM_hsa_3601	chr2:219206630-219206714	+	TCCCCTGCTCCCTTGTTCCCCAGT	No	Yes	No	0	0	0	Yes	
NM_hsa_3602	chr2:219271110-219271179	+	AGAACTTGTTGGCCTGGGTG	No	Yes	No	0	0	0	No	
NM_hsa_3603	chr2:219925417-219925517	+	GCGGGGGGCGGCGGGCGGCGGGGC	No	Yes	No	0	0.0110371	0	No	
NM_hsa_3605	chr2:220148015-220148106	+	AACTGGGACCAGCGCCTGCAG	No	Yes	No	0	0	0	Yes	
NM_hsa_3606	chr2:229156765-229156851	+	ATGGGAATGAGGATCTGAGG	No	Yes	No	0	0	0	No	
NM_hsa_3609	chr2:232379278-232379356	+	CGCGGTGTCCCAGGTCTTCGC	No	Yes	No	0	0	0	No	
NM_hsa_3610	chr2:234184307-234184403	+	GGTCGATGATGATTGGTAAAA	No	Yes	No	0.227943	0.331113	0.219859	No	
NM_hsa_3611	chr2:234197478-234197559	+	TTTGATTGTTGGGAGTGCTGGT	No	Yes	No	0	0	0	No	
NM_hsa_3612	chr2:235709414-235709507	+	GCTGCCTCCACTCCTGTCTGAC	No	Yes	No	0	0	0	No	
NM_hsa_3615	chr2:238886934-238887024	+	CACCTGTGATGTGGGCTCTGATG	No	Yes	Yes	0	0	0	No	
NM_hsa_3615	chr2:238886939-238887016	+	CACCTGTGATGTGGGCTCTGATG	No	Yes	Yes	0	0	0	No	
NM_hsa_3618	chr2:239404146-239404232	+	ACTCGGACTGAGCGCTTGGAAG	No	Yes	No	0	0	0	No	
NM_hsa_3619	chr2:240322061-240322158	+	ACCCCCGCGCCCCCGCCCCGGC	No	Yes	No	10.2859	5.76136	1.90544	Yes	KapB+cMyc>KapB>H
NM_hsa_3622	chr2:857876-857952	-	TCAGTTATGTCAGCGTGTGG	No	Yes	No	0	0	0	No	
NM_hsa_3623	chr2:12122675-12122754	-	ACCCCAGAGATGGAGGCTGCAGT	No	Yes	No	0	0	0	No	
NM_hsa_3625	chr2:29862748-29862824	-	CTGGGAGGATATGTGGATGA	No	Yes	No	0	0	0	No	
NM_hsa_3626	chr2:37195372-37195464	-	ACTTAAAGGAATTGTCGGAT	No	Yes	No	0.113971	0	0	Yes	
NM_hsa_3627	chr2:37883958-37884037	-	TAGTGAGTTAGAGATGGAGGGA	No	Yes	No	0	0	0	Yes	
NM_hsa_3628	chr2:48372001-48372079	-	CTAGCATCACCAGCATTTGAG	No	Yes	No	0	0	0	No	
NM_hsa_3633	chr2:69413998-69414092	-	CATCCATTCTCTGCCTGCCTTCT	No	Yes	No	0	0	0	Yes	
NM_hsa_3634	chr2:75340449-75340537	-	GTGAGCCTTGAAGCCTTGAG	No	Yes	No	0.0569857	0.0662225	0	Yes	
NM_hsa_3636	chr2:85783034-85783123	-	AAAGGATTCAGTCTGTGCTT	No	Yes	No	0	0	0	No	
NM_hsa_3638	chr2:88519242-88519312	-	TGTCACAGCAACTGCCTGTG	No	Yes	No	0	0	0	No	
NM_hsa_3639	chr2:90052219-90052305	-	CAGCACTGTGGGTACTTGAG	No	Yes	No	0.227943	0.0993338	0.183216	Yes	
NM_hsa_3640	chr2:101440034-101440115	-	TCAGTGCATCCCAAAACCTTG	No	Yes	No	0	0	0	No	
NM_hsa_3641	chr2:102313765-102313857	-	CGGGGAGGAAGCGGGCTGGG	No	Yes	No	0	0	0	No	
NM_hsa_3642	chr2:103048746-103048828	-	CTCTGATCAGGCAAAGTTGCAGG	No	Yes	No	0	0	0	No	
NM_hsa_3644	chr2:113994289-113994373	-	TGACTTCTCTTTGCTCCCCAG	No	Yes	No	0	0	0	No	
NM_hsa_3645	chr2:117475329-117475394	-	TCGAATCCTGTTCGTGACGCC	No	Yes	No	1.05424	1.65556	2.78488	Yes	H>KapB>KapB+cMyc
NM_hsa_3646	chr2:118874367-118874439	-	AGGGACTTTCAGGGACAGCAGA	No	Yes	No	0.341914	0.298001	0.256502	Yes	KapB+cMyc>KapB>H
NM_hsa_3649	chr2:146888421-146888508	-	CTTCAGAGGAACCACTCAAG	No	Yes	No	0	0	0	No	
NM_hsa_3651	chr2:169145566-169145658	-	TTTGGGACCTGGTCCAGTTAC	No	Yes	No	0	0	0	No	
NM_hsa_3653	chr2:197550903-197550996	-	TTTGCAGTAACAGGTGTAAGC	No	Yes	No	0.0569857	0.132445	0.183216	Yes	H>KapB>KapB+cMyc
NM_hsa_3654	chr2:204907212-204907293	-	TCAGATGAAGCACTGAAGTC	No	Yes	No	0	0	0	No	
NM_hsa_3658	chr2:208634359-208634444	-	GGGACGCGGCGGCCGGCGCTC	No	Yes	No	0	0	0	No	
NM_hsa_3659	chr2:209190758-209190848	-	CCTGTTCTCCATTTCTTTGA	No	Yes	No	0	0	0	No	
NM_hsa_3664	chr2:241587123-241587210	-	GAGCCCCTCTCTGCCTTCCAGT	No	Yes	No	0	0	0	No	
NM_hsa_3666	chr3:3841000-3841082	+	GCGAAGAGGGTCGGGCGGGA	No	Yes	No	0	0	0	No	
NM_hsa_3667	chr3:5018997-5019093	+	GTTCTCTAGGTCGTCAAGGG	No	Yes	No	0	0	0	No	
NM_hsa_3668	chr3:9439307-9439385	+	GCAGCGGGCTGAGTGAGCTG	No	Yes	No	0	0	0	No	
NM_hsa_3670	chr3:12598629-12598700	+	TTGCAGGTCAGTGCGCTGGAG	No	Yes	No	0	0	0	No	
NM_hsa_3672	chr3:16259795-16259883	+	AGTGGGAACTCTGACATGGC	No	Yes	No	0	0	0	No	
NM_hsa_3675	chr3:37902139-37902236	+	AGAGTGTGGCCTGCAAGTTGGAAG	No	Yes	No	0.0569857	0	0.0732862	No	
NM_hsa_3678	chr3:38012172-38012248	+	CTACTCTTTGCACCCCTCTGC	No	Yes	No	0.7978	0.397335	0	Yes	KapB+cMyc>KapB>H
NM_hsa_3681	chr3:43663823-43663907	+	AGGCAGAGGCCTGGTTGGGCTGT	No	Yes	No	0	0	0	No	
NM_hsa_3684	chr3:45883723-45883820	+	AAGGTAGCGGGAGGGTTGGGCT	No	Yes	No	0	0.132445	0	No	
NM_hsa_3687	chr3:49751453-49751532	+	CACTGACCCCCACCTCCACAG	No	Yes	No	0	0	0	Yes	
NM_hsa_3688	chr3:51229354-51229492	+	ACAGGTTGTCTGTTTACTCAGG	No	Yes	Yes	0	0	0	Yes	
NM_hsa_3688	chr3:51229384-51229463	+	ACAGGTTGTCTGTTTACTCAGG	No	Yes	Yes	0	0	0	Yes	
NM_hsa_3689	chr3:52525295-52525375	+	CCTGACCAGCCCCTTCTCGTGC	No	Yes	No	0	0	0	No	
NM_hsa_3690	chr3:52535112-52535187	+	ACAGGGTCAGGACTGGGCGTGG	No	Yes	No	0.0569857	0.26489	0	No	
NM_hsa_3691	chr3:53222649-53222745	+	TTACCTGTCCCCTGTCCTTAG	No	Yes	No	0	0	0	Yes	
NM_hsa_3693	chr3:64674698-64674778	+	TCCGCTTGCTTTCAGCCTGAGA	No	Yes	No	0	0	0	No	
NM_hsa_3694	chr3:66025083-66025162	+	GCTTGCGGGTTCGGGTCCTG	No	Yes	No	0	0.0662225	0.219859	No	H>KapB>KapB+cMyc
NM_hsa_3695	chr3:69063227-69063322	+	GTCTTCTGCGTTGCACTGAGG	No	Yes	No	0	0	0	No	
NM_hsa_3698	chr3:73779437-73779531	+	CCAAGGTGCCAGTCTCTAGG	No	Yes	No	0.113971	0.331113	0.366431	No	H>KapB>KapB+cMyc
NM_hsa_3700	chr3:77180423-77180505	+	CCTTCAGTAGCTTGCTGTTT	No	Yes	No	0	0	0	No	
NM_hsa_3702	chr3:80101686-80101779	+	GCTACAGTCTCTTCGGAGGCGTG	No	Yes	No	0.3989	0.397335	0.439717	Yes	
NM_hsa_3703	chr3:98312719-98312798	+	TAGGGCTGGGAGGCTGAGGGC	No	Yes	No	0	0	0	No	
NM_hsa_3704	chr3:100428819-100428895	+	GTGCGCCAGCTAATGCCTTG	No	Yes	No	0	0	0	No	
NM_hsa_3705	chr3:101567970-101568050	+	GCCGCGCGCCCTCTCCCCGGG	No	Yes	No	0	0	0	No	
NM_hsa_3706	chr3:107856084-107856167	+	TGGAAGGTGAGGGCTGGATGA	No	Yes	No	0	0	0	No	
NM_hsa_3709	chr3:118838729-118838814	+	CTTGAATGACTTTGACAGCAC	No	Yes	No	0	0	0	No	
NM_hsa_3711	chr3:122813448-122813514	+	CCAGTCCGTGTCTGTGGGTC	No	Yes	No	0	0	0	No	
NM_hsa_3713	chr3:128567501-128567592	+	ACCCTGGGGTCTAGTGTTAG	No	Yes	No	0	0	0	No	
NM_hsa_3714	chr6:37880282-37880379	+	AGTTGGTCCAAGTGTTGTGGGTT	No	Yes	No	0.788303	0.87193	0.818363	Yes	
NM_hsa_3714	chr3:132289115-132289191	+	AGTTGGTCCAAGTGTTGTGGGTT	No	Yes	No	0.788303	0.87193	0.818363	Yes	
NM_hsa_3715	chr3:135915784-135915858	+	CTCGGCTGGCTGGCAGGCTCT	No	Yes	No	0.0569857	0.0662225	0	No	
NM_hsa_3716	chr3:135929710-135929809	+	CCGGTGGTATAGTGGTGAGCATA	No	Yes	No	0.170957	0	0	Yes	
NM_hsa_3717	chr3:141205842-141205917	+	ACGCAGGCGGCAGGGCTGCGG	No	Yes	No	0.0569857	0	0.146572	No	
NM_hsa_3719	chr3:155572540-155572610	+	CTGAAGTCTTCGTGGGTGTAG	No	Yes	No	0	0	0	No	
NM_hsa_3720	chr3:156534821-156534905	+	TTGCGTGACCTGCCGGGCTGC	No	Yes	No	0	0	0	No	
NM_hsa_3721	chr3:156878029-156878118	+	CTGCGAGGGGAGTGTTGGGTG	No	Yes	No	0	0	0	No	
NM_hsa_3724	chr3:159605671-159605741	+	TGAAATGCAAGGAAGTGTTTGG	No	Yes	No	0	0	0	No	
NM_hsa_3727	chr3:169483059-169483136	+	CTCTGCAGACCAGACGCGGTT	No	Yes	No	0	0	0	No	
NM_hsa_3728	chr3:176232880-176232951	+	TTTCCCAGCCAACGCACCAA	No	Yes	No	50.6603	48.7729	84.0227	Yes	
NM_hsa_3729	chr3:178866226-178866356	+	CGCCCGCTCTCCTCTCCCTCGGC	No	Yes	Yes	0	0	0.0732862	Yes	
NM_hsa_3729	chr3:178866243-178866331	+	CGCCCGCTCTCCTCTCCCTCGGC	No	Yes	Yes	0	0	0.0732862	Yes	
NM_hsa_3731	chr3:181301731-181301813	+	AGTTGGCTAAGAGGGAGTGGA	No	Yes	No	0.0569857	0	0	No	
NM_hsa_3733	chr3:183907524-183907615	+	TTCCAGAGTGGGGGATAGTGG	No	Yes	No	0	0	0	No	
NM_hsa_3736	chr3:186504502-186504594	+	CAGCACTAGATTGTAAAGACTGG	No	Yes	No	0	0	0.293145	Yes	
NM_hsa_3737	chr3:187329742-187329812	+	CACAGGGATCAGCATTGAGG	No	Yes	No	0	0	0	No	
NM_hsa_3738	chr3:187463609-187463698	+	GTGACATAAGAGGGGGAGTG	No	Yes	No	0	0	0	No	
NM_hsa_3743	chr3:193921970-193922057	+	TCCGGCGCGGGCGTGTCTCTGC	No	Yes	No	0	0	0	No	
NM_hsa_3748	chr3:13874774-13874844	-	TGTAGGGATGGACGGCATGAGA	No	Yes	No	0	0	0	No	
NM_hsa_3749	chr3:14928720-14928811	-	AAGGAGCTCAGAGTTTAGTGA	No	Yes	No	0	0	0	No	
NM_hsa_3751	chr3:20801583-20801679	-	TGAAAAGCACTTTGAAAAGAGAT	No	Yes	No	1.53861	3.70846	4.47046	Yes	H>KapB>KapB+cMyc
NM_hsa_3754	chr3:32735656-32735741	-	AAGTTTCTCTGAAAGTGTAGAGT	No	Yes	No	7.02824	4.12235	6.9683	Yes	
NM_hsa_3756	chr3:49058873-49058937	-	AAGGGCAAGCTGTGATAGTG	No	Yes	No	0	0	0	No	
NM_hsa_3759	chr3:51449740-51449832	-	TGCTGTAGGAGGTTTTAAAG	No	Yes	No	0	0	0	No	
NM_hsa_3761	chr3:53292044-53292133	-	CTGGAGCTCCAGGTGGTGGG	No	Yes	No	0	0	0	No	
NM_hsa_3762	chr3:56384091-56384173	-	TCTAGCATCAGTGATATTGC	No	Yes	No	0	0	0	No	
NM_hsa_3763	chr3:64057619-64057704	-	AAAGTTCTCTGAATGTGTAAAGC	No	Yes	No	0	0	0	No	
NM_hsa_3765	chr3:104974866-104974953	-	GCTGTGATGGCCGAGTGGTTAAGG	No	Yes	No	0.113971	0.331113	0.0732862	Yes	
NM_hsa_3766	chr3:105289178-105289254	-	CAAATTGACATTTTGGAACTGA	No	Yes	No	0	0	0	No	
NM_hsa_3767	chr3:126249243-126249333	-	CTGTCTCCGCCCCAGGGCCC	No	Yes	No	0	0	0	No	
NM_hsa_3768	chr3:127317018-127317107	-	TGGAGCGGAGGTAGAAGCTGA	No	Yes	No	0	0	0	No	
NM_hsa_3769	chr3:131071267-131071337	-	TGGTTCCTTTGGTTGCTGGG	No	Yes	No	0.113971	0	0	Yes	
NM_hsa_3770	chr3:132776828-132776910	-	TGGAAGGATGCAGGGCAGAGG	No	Yes	No	0	0	0	No	
NM_hsa_3772	chr3:134131054-134131145	-	TTGTCGGTGTTCTGGATTTTGGC	No	Yes	No	0	0	0	No	
NM_hsa_3773	chr3:138624104-138624175	-	TGAGGTAGGAGGTTGCAGTGA	No	Yes	No	1.49113	0.960226	0.207644	Yes	KapB+cMyc>KapB>H
NM_hsa_3774	chr3:174158685-174158750	-	GGGCGCGAGCCGCGGCGCGA	No	Yes	No	0	0	0	No	
NM_hsa_3775	chr3:175325987-175326073	-	CAGATTGTGGACTATGGGCTA	No	Yes	No	0	0	0	No	
NM_hsa_3776	chr3:179332553-179332637	-	GTAGGCACATTACCCATCTGACA	No	Yes	No	0.0284929	0.0331113	0.0366431	Yes	H>KapB>KapB+cMyc
NM_hsa_3777	chr3:184429809-184429895	-	GCGCGGGCGCGGACTGAGGC	No	Yes	No	0	0	0	No	
NM_hsa_3778	chr3:188648161-188648233	-	CTCTGATCAGGGTGAGACTC	No	Yes	No	0	0.132445	0.0732862	Yes	
NM_hsa_3779	chr3:195676618-195676699	-	ACTAGATTGTGAGCTGCGTGA	No	Yes	No	0.296326	0.14569	0.175887	Yes	
NM_hsa_3782	chr4:1005482-1005552	+	CGGCCCCGGCCCCCGGCCCCGC	No	Yes	No	0.370407	0.26489	0.256502	Yes	KapB+cMyc>KapB>H
NM_hsa_3784	chr4:1807719-1807788	+	TGCCTCCCACCCCTTCCCCAGT	No	Yes	No	0	0.132445	0	Yes	
NM_hsa_3785	chr4:1858118-1858198	+	ACCGTGGACGCCGGGGCTCGCAG	No	Yes	No	0.0569857	0	0.219859	No	
NM_hsa_3786	chr4:2061880-2061950	+	AGCGCCCGCCGCGGCTGGGC	No	Yes	No	0	0	0	Yes	
NM_hsa_3787	chr4:2780044-2780126	+	TTTCTGGCCCTGTGTCTTAGG	No	Yes	No	0	0	0	No	
NM_hsa_3789	chr4:3728701-3728781	+	CCCCTCTGAGCCTCTGCCCCCCA	No	Yes	Yes	0	0	0	No	
NM_hsa_3789	chr4:3728681-3728801	+	CCCCTCTGAGCCTCTGCCCCCCA	No	Yes	Yes	0	0	0	No	
NM_hsa_3790	chr4:3728701-3728781	+	AGGGGAGGGTCTCAGGCGGAGA	No	Yes	Yes	0	0	0	No	
NM_hsa_3790	chr4:3728698-3728786	+	AGGGGAGGGTCTCAGGCGGAGA	No	Yes	Yes	0	0	0	No	
NM_hsa_3791	chr4:3767392-3767486	+	CTCGCCGAGCGCGCGCCCCGC	No	Yes	No	0	0	0	No	
NM_hsa_3793	chr4:7087104-7087184	+	AACTGGGCATGTTGGAACTAAG	No	Yes	No	0.0379905	0	0.0244288	No	
NM_hsa_3794	chr4:8201020-8201112	+	CAGCCCCGTGGGGCCAGCCAG	No	Yes	No	0	0	0	No	
NM_hsa_3796	chr4:8780482-8780578	+	ATCAGTTTTGGGGGTAGAGC	No	Yes	No	0	0	0	No	
NM_hsa_3797	chr4:13629077-13629147	+	CGGGGCCCGGCGGCGGCGGCGGT	No	Yes	No	0.0633175	0.0412051	0	Yes	KapB+cMyc>KapB>H
NM_hsa_3798	chr4:14945094-14945157	+	CTGTTTGGATCACTGTGGTA	No	Yes	No	0	0	0	No	
NM_hsa_3799	chr4:20253503-20253582	+	CGCCCGGACGGCGGAGCTTGG	No	Yes	No	0	0	0	No	
NM_hsa_3802	chr4:39529329-39529419	+	GCCGCAGGTGTAGGAGCTTTG	No	Yes	No	0	0	0	No	
NM_hsa_3803	chr4:40428380-40428463	+	AGAGGCCTGGAAGGCTGAGG	No	Yes	No	0	0	0	No	
NM_hsa_3804	chr4:42142140-42142207	+	TCCCAGTGACACTTTAACCTGT	No	Yes	No	0	0	0	No	
NM_hsa_3808	chr4:78210509-78210591	+	ATGTCATGAGTGTAGGACTGT	No	Yes	No	0	0.132445	0	No	
NM_hsa_3809	chr4:83298440-83298528	+	GTTCCTGCCCACCCAGGGACGCC	No	Yes	No	0.3989	0	0.146572	Yes	
NM_hsa_3810	chr4:83719306-83719387	+	GGGCCGCGGCGCGCAGCGGG	No	Yes	No	0	0	0	No	
NM_hsa_3815	chr4:103682221-103682288	+	GTCGCGGGTGCGGGAGCTGC	No	Yes	No	0	0	0	No	
NM_hsa_3817	chr4:118623798-118623875	+	AACATCTGGGTTGAGAGGGTTA	No	Yes	No	0	0	0	Yes	
NM_hsa_3818	chr4:119976086-119976169	+	CCTCTGTAGACCACTGTTGTTC	No	Yes	No	0	0	0	No	
NM_hsa_3819	chr4:121439183-121439271	+	TCCCTTCCTTCCTTTTTTGC	No	Yes	No	0	0	0	No	
NM_hsa_3820	chr4:122618347-122618432	+	CTGCTGGAACCAGGAGGAGGCG	No	Yes	No	0	0	0	No	
NM_hsa_3822	chr4:141073295-141073361	+	GTCGCGAGCAAAGAGCGGAG	No	Yes	No	0	0	0	No	
NM_hsa_3823	chr4:142053610-142053687	+	TGCCGCGGGTCGGGGCGGCG	No	Yes	No	0.655336	0.463558	0.256502	No	KapB+cMyc>KapB>H
NM_hsa_3824	chr4:147607357-147607441	+	TTGTCTTTTTCTCTGACCAC	No	Yes	No	0.3989	0.397335	0.732862	Yes	
NM_hsa_3828	chr4:170191769-170191855	+	CAGAGGCCGCGGGCTGCTCCG	No	Yes	No	0	0	0	No	
NM_hsa_3829	chr4:175064447-175064529	+	TGATTAAGGAGGTTGTATAG	No	Yes	No	0.0569857	0	0	No	
NM_hsa_3832	chr4:1006319-1006406	-	CGGGCGGCGGCGGCCGGCGGGA	No	Yes	No	0.940265	0.695336	0.366431	Yes	KapB+cMyc>KapB>H
NM_hsa_3834	chr4:4861712-4861783	-	TGGCCGCGGCGGCGCTGGGG	No	Yes	No	0	0	0	No	
NM_hsa_3835	chr4:5209389-5209477	-	AGAAAGTGTCTGAGACTGCATC	No	Yes	No	0	0	0	No	
NM_hsa_3837	chr4:17448673-17448758	-	TCGAGGAGCTCACAGTCTAGTG	No	Yes	No	1850.92	1689.91	1203.25	Yes	KapB+cMyc>KapB>H
NM_hsa_3838	chr4:56481080-56481169	-	TGAGGTATTAGATAGTAAAG	No	Yes	No	0	0.0662225	0	No	
NM_hsa_3840	chr4:84437523-84437597	-	ATCCCATTGCCAGGATTTAT	No	Yes	No	0	0	0.0732862	Yes	
NM_hsa_3843	chr4:108910786-108910883	-	CTGCTGAGCAGCGGGACCTGG	No	Yes	No	0	0	0	No	
NM_hsa_3846	chr4:125944950-125945044	-	TAATTTTATGTATAAACTAATA	No	Yes	No	0.0284929	0.0993338	0.109929	Yes	H>KapB>KapB+cMyc

Additional file 5

Name	locus	strand	sequence	mirdeep2	mireap	miranalyzer	RPM_HU_KapB_cMyc	RPM_HU_KaposinB	RPM_HUVEC	IP_confidence	annotation
NM_hsa_3848	chr4:156681086-156681165	-	CGCGCCCGCGACCCGGCCAG	No	Yes	No	0	0	0	No	
NM_hsa_3849	chr4:170109194-170109260	-	CTGAAGGCTCTAGGAAGAATCC	No	Yes	No	0	0	0	No	
NM_hsa_3850	chr4:175204224-175204314	-	GACTCCAAGTAGTAACCCAG	No	Yes	No	0	0	0	No	
NM_hsa_3851	chr4:176881945-176882036	-	TCATTGCATCACAGAATTTTA	No	Yes	No	1.51012	0.860893	0.769506	Yes	KapB+cMyc>KapB>H
NM_hsa_3852	chr4:187010236-187010327	-	TGTGACTGGTTGATGTGAGGG	No	Yes	No	0	0	0	No	
NM_hsa_3856	chr5:6713980-6714067	+	AGGCCCGTCCGTCCGTCCGT	No	Yes	No	1.89952	1.53415	1.55123	Yes	
NM_hsa_3858	chr5:14441417-14441517	+	TGCAGATGCTCGGGCTGGCGCTGC	No	Yes	No	0	0	0	No	
NM_hsa_3859	chr5:14712519-14712612	+	CTCTCGGACTGCATCTCTGCT	No	Yes	No	0	0	0	No	
NM_hsa_3860	chr5:14871905-14871990	+	ACGAGGCGCCGCCGCCGCCG	No	Yes	No	0	0	0.0732862	Yes	
NM_hsa_3861	chr5:21495751-21495842	+	TGATCAGAATCTCCAGGGGA	No	Yes	No	1.82354	1.72179	1.75887	Yes	
NM_hsa_3862	chr5:21505868-21505964	+	TCTCCCTCCCTCCCCGCCCC	No	Yes	No	0	0.0662225	0	Yes	
NM_hsa_3863	chr5:34537998-34538080	+	CAGGGACCCCTGACTGACAACG	No	Yes	No	0	0.0220742	0	No	
NM_hsa_3864	chr5:38403574-38403661	+	ACAGTGAATCTGACAACTGA	No	Yes	No	0.0569857	0.0662225	0	No	
NM_hsa_3865	chr5:39074595-39074658	+	CTGAGGGAGCACTGAGCCGAC	No	Yes	No	0	0	0	No	
NM_hsa_3866	chr5:56205885-56205967	+	GAGCTCGCCAGCCGGATGTG	No	Yes	No	0	0	0	No	
NM_hsa_3867	chr5:62451159-62451242	+	TCAGGGTTCCCTCAAGTGACAG	No	Yes	Yes	0	0	0	No	
NM_hsa_3867	chr5:62451129-62451267	+	TCAGGGTTCCCTCAAGTGACAG	No	Yes	Yes	0	0	0	No	
NM_hsa_3868	chr5:75480014-75480106	+	ACTAGGCTGTGATCTCCTGGAG	No	Yes	No	0	0	0	No	
NM_hsa_3869	chr5:78104703-78104777	+	GGGCAGCACACAGCAAGACG	No	Yes	No	0	0	0	No	
NM_hsa_3870	chr5:78280877-78280962	+	AAGCCGACGTCGTTCCAGCCTAG	No	Yes	No	0	0	0	No	
NM_hsa_3871	chr5:91140647-91140736	+	TAGAAAGGTGTGTGCCACCA	No	Yes	No	0	0	0	No	
NM_hsa_3872	chr5:92916188-92916286	+	GTGCCCACGACGCACGGTCCAG	No	Yes	Yes	0	0	0	No	
NM_hsa_3872	chr5:92916198-92916279	+	GTGCCCACGACGCACGGTCCAG	No	Yes	Yes	0	0	0	No	
NM_hsa_3874	chr5:103289513-103289574	+	CAGCGGGCAGAGCCCTGCCG	No	Yes	No	0	0	0	No	
NM_hsa_3875	chr5:104899421-104899514	+	AAGTGCTGGGTAGAGAGGGCG	No	Yes	No	0	0	0	Yes	
NM_hsa_3876	chr5:107735053-107735130	+	TTAAACGTCCTTGCCTGGCAG	No	Yes	No	0	0	0	No	
NM_hsa_3878	chr5:116376612-116376689	+	GCATGTTCTGAACTGGGTCAT	No	Yes	No	0	0	0	No	
NM_hsa_3880	chr5:122403755-122403839	+	GCGCGCACGCGCGCGTGTGTGGTG	No	Yes	No	0.0189952	0.0220742	0.0732862	Yes	H>KapB>KapB+cMyc
NM_hsa_3881	chr5:126366412-126366511	+	GCCGGCGGGCGCTGCGGGACTCT	No	Yes	No	0.113971	0	0	No	
NM_hsa_3882	chr5:127418874-127418967	+	CTCTGAAGCCGTGGCTGGGG	No	Yes	No	0	0	0	No	
NM_hsa_3883	chr5:129606615-129606694	+	TGATGGAAAGTGGAGTCTGAG	No	Yes	No	0	0	0	No	
NM_hsa_3884	chr5:132299919-132299988	+	ATAGATCTCGCTGTTGTCGCC	No	Yes	No	0	0	0	No	
NM_hsa_3885	chr5:132458948-132459035	+	CTCCTTGCTCCAGGTCCTGTCT	No	Yes	No	0	0	0	No	
NM_hsa_3886	chr5:133340525-133340610	+	GCGTCCAGGCCGGGCCAGGGT	No	Yes	No	0	0	0	No	
NM_hsa_3889	chr5:134261642-134261738	+	TGTGTTGGCATCTGCTCGGGCGTA	No	Yes	No	0.569857	0.463558	0.659576	Yes	
NM_hsa_3890	chr5:135395110-135395193	+	CAAGGGAATAGAGCATCTGA	No	Yes	No	0	0	0	No	
NM_hsa_3893	chr5:139090301-139090374	+	ATTCGCGGTTGGGACCTGGCA	No	Yes	No	0	0	0	Yes	
NM_hsa_3894	chr5:141229306-141229381	+	TCTCTGTTTTCCGACCTCTCCGG	No	Yes	No	0.0569857	0.132445	0.0732862	Yes	
NM_hsa_3895	chr5:141229550-141229623	+	TATCCAGTTCCGCAATTCACACT	No	Yes	No	0	0	0	No	
NM_hsa_3896	chr5:148686130-148686224	+	CGCTGGAATGGGAGTCAGAAGGC	No	Yes	No	0	0	0	Yes	
NM_hsa_3900	chr5:149492511-149492607	+	ACACAGTGATGGATCTGAGG	No	Yes	No	0	0	0	No	
NM_hsa_3903	chr5:155765690-155765769	+	GGCTTCTCAGACTGGTGTTGA	No	Yes	No	0	0.0331113	0	Yes	
NM_hsa_3904	chr5:158453266-158453353	+	GTTAATGAAGACTGTTTTTTTTT	No	Yes	No	0	0	0	No	
NM_hsa_3906	chr5:166405925-166406021	+	GTGCGCGCGCGCGCGTGTGTTGTG	No	Yes	No	0.132967	0.0551854	0.329788	Yes	
NM_hsa_3907	chr5:166592236-166592332	+	GCAGAGAGAAGCATTGGACA	No	Yes	No	0.227943	0.463558	1.17258	No	H>KapB>KapB+cMyc
NM_hsa_3909	chr5:176853053-176853143	+	AAGAGGTCGGGGAACGGCTGC	No	Yes	No	0	0	0	Yes	
NM_hsa_3912	chr5:180229852-180229924	+	GGCGGGGCGGGGCGGACTGA	No	Yes	No	0	0	0.0732862	Yes	
NM_hsa_3914	chr5:180689712-180689778	+	TACCCCAGTGCTGAGGGCCAAGA	No	Yes	No	0	0	0	Yes	
NM_hsa_3915	chr5:473010-473106	-	CTCGCGCCCTTTCCCACCGC	No	Yes	No	0	0	0	Yes	
NM_hsa_3916	chr5:38556867-38556952	-	CCCGCGCGCGCGGGCGCGCG	No	Yes	No	0	0	0	No	
NM_hsa_3917	chr5:40832754-40832843	-	TATCAGTGATGTTGTAAAAAT	No	Yes	No	0	0	0	Yes	
NM_hsa_3918	chr5:40832754-40832843	-	TTTCAGCATTTAACTGAGA	No	Yes	No	0	0	0	No	
NM_hsa_3919	chr5:41904043-41904130	-	GTGACGTCTGGCTGGCAGGA	No	Yes	No	0	0	0	No	
NM_hsa_3924	chr5:76376313-76376407	-	GTGGAGGACTGAGAAGGTGA	No	Yes	No	0	0	0.146572	No	
NM_hsa_3925	chr5:77623407-77623501	-	TGAGGTAGTAGATAATAAAGT	No	Yes	No	0	0.0220742	0.0244288	Yes	H>KapB>KapB+cMyc
NM_hsa_3926	chr5:82360127-82360219	-	TTGCATGTGGAAATGTCTGC	No	Yes	No	0	0	0	No	
NM_hsa_3927	chr5:84845774-84845865	-	TCCCCGGCATCTCCTCCCAC	No	Yes	No	28.6638	21.5223	24.4043	Yes	
NM_hsa_3930	chr5:113181176-113181272	-	TGAGGAAGCACTGTAGCTCA	No	Yes	No	0	0.0882967	0.097715	No	H>KapB>KapB+cMyc
NM_hsa_3932	chr5:138840660-138840735	-	CGCAGGCCAGAGTTGCAGTGG	No	Yes	Yes	0	0	0	No	
NM_hsa_3932	chr5:138840644-138840750	-	CGCAGGCCAGAGTTGCAGTGG	No	Yes	Yes	0	0	0	No	
NM_hsa_3933	chr5:142783411-142783497	-	GCGCCGTCGGGGCCGGGGTG	No	Yes	No	3.1912	3.04624	2.85816	Yes	KapB+cMyc>KapB>H
NM_hsa_3936	chr5:148808494-148808573	-	AGATGCAGCACTGCACCTCA	No	Yes	No	0	0	0	No	
NM_hsa_3937	chr5:155106164-155106248	-	TGTTAATTGCAGGGGACATTG	No	Yes	No	0	0	0	No	
NM_hsa_3942	chr5:169356028-169356118	-	CAGGGCATGACAGAACTTCA	No	Yes	No	0	0	0	Yes	
NM_hsa_3944	chr5:173241625-173241724	-	TGAGGTAGTAGGGTTTGAGGTTT	No	Yes	No	0	0.0331113	0	Yes	
NM_hsa_3945	chr5:175158177-175158263	-	ATGGGAACTGGAGTCCCAGCCT	No	Yes	No	0.0569857	0	0	No	
NM_hsa_3946	chr5:176305523-176305603	-	CCTGCCACACCCACAGCTTGCA	No	Yes	No	0	0	0	No	
NM_hsa_3948	chr5:179316784-179316863	-	CTACAGCCTCCATCTCCCGGACT	No	Yes	No	0.0569857	0	0.146572	Yes	
NM_hsa_3949	chr6:393374-393467	+	AGCCGGCGGGGGCGCGCCGG	No	Yes	No	232.559	137.345	15.2435	Yes	KapB+cMyc>KapB>H
NM_hsa_3950	chr6:2784455-2784595	+	TCTCCTTTGTCTCTGTGTGTGGC	No	Yes	Yes	0.113971	0	0	Yes	
NM_hsa_3950	chr6:2784478-2784565	+	TCTCCTTTGTCTCTGTGTGTGGC	No	Yes	Yes	0.113971	0	0	Yes	
NM_hsa_3951	chr6:3725451-3725536	+	TGCCCGTCCTCCCTGCCCAGA	No	Yes	No	0	0	0	No	
NM_hsa_3952	chr6:7120217-7120313	+	GTGGATGATGATGCCTCTGACA	No	Yes	No	0.7978	1.05956	1.09929	No	H>KapB>KapB+cMyc
NM_hsa_3953	chr6:12012411-12012492	+	CCCCCGCCCGCGCGCCCCGCG	No	Yes	No	0	0	0	No	
NM_hsa_3958	chr6:18836381-18836455	+	ACTCTGGACTCTGAATCCAGC	No	Yes	No	38.1234	36.897	47.4528	Yes	
NM_hsa_3959	chr6:21686023-21686094	+	TCACTTCCTGTTCTGTGGCCTGT	No	Yes	No	0	0	0.439717	Yes	
NM_hsa_3960	chr6:21768023-21768090	+	CATTTTGTTGGGCAGCATTGGA	No	Yes	No	0	0	0	No	
NM_hsa_3961	chr6:24721902-24721968	+	GTTCAGACGTTGCTGCTGTGG	No	Yes	No	0	0.132445	0	No	
NM_hsa_3962	chr6:26172292-26172388	+	AGGCCGCTAACTGGTGGTTCCC	No	Yes	No	0	0	0	No	
NM_hsa_3963	chr6:26172292-26172388	+	GCCACCAGATCGCGGGATAT	No	Yes	No	0	0	0	No	
NM_hsa_3964	chr6:26328352-26328450	+	GTCAGAGGGCCAGTGGCGCAATGGA	No	Yes	No	17.8365	23.1117	60.3879	Yes	H>KapB>KapB+cMyc
NM_hsa_3965	chr6:26328352-26328450	+	CAGGTTCGACTCCTGGCTGGCTCG	No	Yes	No	13.9615	9.90026	3.51774	Yes	KapB+cMyc>KapB>H
NM_hsa_3966	chr6:27107051-27107122	+	AGACCTTTGTTCTCTGACCA	No	Yes	No	0	0	0	No	
NM_hsa_3967	chr6:27219345-27219439	+	TCTGCACCCTCTGAGTCCCGC	No	Yes	No	0	0	0	No	
NM_hsa_3969	chr6:28687504-28687569	+	GCATGCACGAGGCCCCGGGT	No	Yes	No	2.67833	2.78135	1.17258	Yes	
NM_hsa_3970	chr6:28732097-28732196	+	GCCAAAATTGCTCAGTTGGGAGA	No	Yes	No	4.87228	3.67535	0.329788	Yes	KapB+cMyc>KapB>H
NM_hsa_3971	chr6:28918819-28918903	+	TCGGGCGGGAGTGGTGGCTTTT	No	Yes	No	0.968757	1.72179	2.08866	Yes	H>KapB>KapB+cMyc
NM_hsa_3973	chr6:30685433-30685523	+	GTTGCAGTCGTGGCTGGAGG	No	Yes	No	0	0	0	No	
NM_hsa_3975	chr6:31515849-31515949	+	CTGCTGCCTTTTTTCCTTCCCCAG	No	Yes	No	0	0	0	Yes	
NM_hsa_3978	chr6:32147587-32147664	+	TTTCTCTCTCCACTTCCTCAGA	No	Yes	No	0	0	0.0732862	Yes	
NM_hsa_3985	chr6:35465534-35465612	+	TTCCAGCTCGCGCCTCGGACG	No	Yes	No	0	0	0	No	
NM_hsa_3986	chr6:43224601-43224671	+	TCTGACTCCGAGTCCAGGGCTCT	No	Yes	No	2.82079	2.98001	2.45509	Yes	
NM_hsa_3987	chr6:43395613-43395687	+	AAGCCGGCTGTTACCCTGCA	No	Yes	No	0	0	0.0732862	No	
NM_hsa_3989	chr6:43762884-43762978	+	CCAGGCTCGGCTCCTGGCTG	No	Yes	No	1.22519	1.05956	0.622933	Yes	KapB+cMyc>KapB>H
NM_hsa_3995	chr6:90062710-90062796	+	TCTCGTCCCGGCCCTGCCCGCT	No	Yes	No	0.170957	0.397335	0.146572	Yes	
NM_hsa_3996	chr6:91419277-91419348	+	ACTGCGGGCTATAGGACTTGC	No	Yes	No	0	0	0	No	
NM_hsa_3999	chr6:107781153-107781227	+	GCCGTGGCCGGGCGTGAAGG	No	Yes	No	0	0	0	No	
NM_hsa_4000	chr6:108396253-108396325	+	ATGGCCGTCGGCTGCGTTCG	No	Yes	No	0	0	0	No	
NM_hsa_4003	chr6:127440552-127440625	+	CACCCTTGCCTCTCGGCGCCC	No	Yes	No	3.36216	3.64224	5.64304	Yes	H>KapB>KapB+cMyc
NM_hsa_4004	chr6:131122598-131122671	+	GCCGGGGTGGCGGCGGCGGTGGC	No	Yes	No	0.00876704	0.00946036	0.0104695	No	H>KapB>KapB+cMyc
NM_hsa_4005	chr6:136067112-136067204	+	AGTGGCATAGTGTAGTGGTT	No	Yes	No	0	0	0	No	
NM_hsa_4006	chr6:136610583-136610665	+	CTGTGGGTCTCCAGGCAGGT	No	Yes	No	0	0	0	No	
NM_hsa_4007	chr6:139485567-139485634	+	GCTTCCTGTAGTGTGCTGGAC	No	Yes	Yes	0	0	0	No	
NM_hsa_4007	chr6:139485542-139485668	+	GCTTCCTGTAGTGTGCTGGAC	No	Yes	Yes	0	0	0	No	
NM_hsa_4008	chr6:152313103-152313177	+	CAGGAAACTTCCCTGACACCAG	No	Yes	Yes	0	0	0	No	
NM_hsa_4008	chr6:152313088-152313196	+	CAGGAAACTTCCCTGACACCAG	No	Yes	Yes	0	0	0	No	
NM_hsa_4009	chr6:155055412-155055492	+	CTTCCCGGGTCCTCACTGAG	No	Yes	No	0	0.132445	0.146572	No	H>KapB>KapB+cMyc
NM_hsa_4010	chr6:156474325-156474418	+	AGAAGTGTGGCTCAGCCTGG	No	Yes	No	0	0	0	No	
NM_hsa_4011	chr6:167810009-167810093	+	TGGGGTCAGTTTGTGCTGTTA	No	Yes	No	0	0	0	No	
NM_hsa_4012	chr6:169263977-169264072	+	GTCCCCACTCCAGGTCCTGTG	No	Yes	No	0	0	0	No	
NM_hsa_4013	chr6:170577779-170577875	+	GGGTGACATCTGGGGTGCCA	No	Yes	No	0	0	0	No	
NM_hsa_4014	chr6:1390130-1390224	-	GGGGCCGGCGGCGGCCCGCC	No	Yes	No	0	0	0	No	
NM_hsa_4015	chr6:13771400-13771477	-	TTATGGTTGCCTGGAACTGG	No	Yes	No	0	0	0	No	
NM_hsa_4016	chr6:26027443-26027511	-	TTTGTTGTCAGCTGGTTAGA	No	Yes	No	0	0	0.146572	No	
NM_hsa_4017	chr6:27095039-27095124	-	CGCTGCGGGCTGGAGAGCTG	No	Yes	No	0	0.0993338	0	No	
NM_hsa_4018	chr6:28626018-28626100	-	GAGGTGGGGATGTAGCTCAGTGGT	No	Yes	No	140.517	196.239	593.179	Yes	H>KapB>KapB+cMyc
NM_hsa_4021	chr6:39510663-39510733	-	TGTATGGGAGTCAGAGTGGA	No	Yes	No	0	0	0	No	
NM_hsa_4023	chr6:44197358-44197420	-	TTGCTCAGTTCAGCAGTGAC	No	Yes	No	0	0	0	Yes	
NM_hsa_4024	chr6:51098886-51098955	-	GCTTAAGTGTCTTGATGCTGT	No	Yes	No	0	0	0	No	
NM_hsa_4026	chr6:71150956-71151033	-	CACCTCTGAGTCTCAAGCAA	No	Yes	No	0	0	0	No	
NM_hsa_4030	chr6:72159259-72159340	-	ATTTGGATCTTGGGGGCGGAC	No	Yes	No	0	0	0	Yes	
NM_hsa_4031	chr6:75355656-75355743	-	CTACACTGAAGCTGCTTGGGT	No	Yes	No	0	0	0	Yes	
NM_hsa_4032	chr6:86746532-86746603	-	GCGCGCGCGCGTGTGTTATT	No	Yes	No	0	0	0	No	
NM_hsa_4033	chr6:94778300-94778392	-	TCTTGGGTTATCTGGCTCTAT	No	Yes	No	0	0	0	No	
NM_hsa_4034	chr6:118610382-118610471	-	ACCAGGTGACTGGTTAAATAAA	No	Yes	No	0	0	0	No	
NM_hsa_4035	chr6:157519560-157519654	-	TACAGTGCTGTGATGACAGG	No	Yes	No	0	0	0	No	
NM_hsa_4037	chr6:167527500-167527579	-	TGTGTGACCTGGTCCACCCAC	No	Yes	No	0	0	0	No	
NM_hsa_4038	chr6:170921670-170921763	-	GCGTCGGGTTCTGTTGGAGTG	No	Yes	No	0	0	0	No	
NM_hsa_4039	chr7:780250-780368	+	CCGGCCACGCGCCTCTGTCTTG	No	Yes	Yes	0.0569857	0.0662225	0.366431	Yes	H>KapB>KapB+cMyc
NM_hsa_4039	chr7:780218-780311	+	CCGGCCACGCGCCTCTGTCTTG	No	Yes	Yes	0.0569857	0.0662225	0.366431	Yes	H>KapB>KapB+cMyc
NM_hsa_4040	chr7:1875935-1876000	+	CGTGCCTGGCTGACCAGAGG	No	Yes	No	0	0	0	No	
NM_hsa_4042	chr7:3699075-3699164	+	AGAAATGGGGCTGGACATCTG	No	Yes	No	0	0	0	No	
NM_hsa_4043	chr7:4471411-4471507	+	CTTTCAGGACGTGACTGATG	No	Yes	No	0	0	0	No	
NM_hsa_4045	chr7:6629658-6629749	+	GCTGCGCGGCGGCAGCTCCCGG	No	Yes	No	0	0.0662225	0	Yes	
NM_hsa_4046	chr7:6703865-6703940	+	CCGGCGGGTGCGGCGGGTGT	No	Yes	No	0	0	0	No	
NM_hsa_4047	chr7:7298817-7298896	+	GGCCGAGGTGGGCGGCTCACG	No	Yes	No	0	0	0	No	
NM_hsa_4048	chr7:20257431-20257504	+	TTCCCAGGTGAGTGACAGTG	No	Yes	No	0	0	0	No	
NM_hsa_4049	chr7:22052327-22052396	+	TGGAAGTTCAGAGGGCTCTGA	No	Yes	No	0	0.0331113	0	No	
NM_hsa_4050	chr7:24099542-24099615	+	GCGGGGAGGGGTGGCGGCGGC	No	Yes	No	0	0	0	No	
NM_hsa_4051	chr7:24613244-24613318	+	GCCGCGCGCCGGGCCGGCCGG	No	Yes	No	0	0	0	No	
NM_hsa_4055	chr7:44150424-44150542	+	CTGAGGCCTGCCTCTCCCCAGA	No	Yes	Yes	0.0569857	0	0	No	
NM_hsa_4055	chr7:44150441-44150522	+	CTGAGGCCTGCCTCTCCCCAGA	No	Yes	Yes	0.0569857	0	0	No	
NM_hsa_4056	chr7:44622154-44622233	+	ACGGAATCTGACCAGGCTTGG	No	Yes	No	0	0.198668	0	No	
NM_hsa_4057	chr7:46780718-46780802	+	AGGGAGGCTATGGAACGTAG	No	Yes	No	0	0	0	No	
NM_hsa_4058	chr7:55137017-55137099	+	TTAGCTCGTGTGGTGATTGCTG	No	Yes	No	0	0	0	No	
NM_hsa_4059	chr7:55434001-55434089	+	CGGGCCTTCCCGATGAGCCG	No	Yes	No	0	0	0	No	
NM_hsa_4061	chr7:64459687-64459767	+	TCCCCTCCTGGCTGGCTCGCCA	No	Yes	No	94.2829	98.4729	145.253	Yes	H>KapB>KapB+cMyc
NM_hsa_4062	chr7:73271516-73271590	+	TAGGGGCTGTGGGACAGAGTG	No	Yes	No	0	0	0	No	
NM_hsa_4064	chr7:74438663-74438744	+	CATCTGCCTCCTCTACCCCTGCA	No	Yes	No	0.0284929	0	0	Yes	
NM_hsa_4065	chr7:74444391-74444481	+	TATGGGACTGGCTCTTGGAGT	No	Yes	No	0	0	0	No	
NM_hsa_4066	chr7:75983286-75983356	+	CTGCAGTGAGCCATGCTCCA	No	Yes	No	0	0	0	No	
NM_hsa_4067	chr7:88119707-88119780	+	TCACTTTGGTATTTCTAGAGC	No	Yes	No	0.0569857	0	0	No	
NM_hsa_4069	chr7:93194424-93194501	+	ACTAGATGTGGAGTCAGAAGGC	No	Yes	No	0	0	0	No	
NM_hsa_4074	chr7:100033800-100033936	+	GGGCTGTCGGGCCGCGGGCTC	No	Yes	Yes	0	0.0662225	0.0732862	No	H>KapB>KapB+cMyc
NM_hsa_4074	chr7:100033774-100033870	+	GGGCTGTCGGGCCGCGGGCTC	No	Yes	Yes	0	0.0662225	0.0732862	No	H>KapB>KapB+cMyc
NM_hsa_4076	chr7:101692234-101692329	+	GAGATCCTTCCTCGCTGGGG	No	Yes	No	0	0	0	No	
NM_hsa_4078	chr7:104624707-104624797	+	CTCTGGCCAGCGGGCATGCG	No	Yes	No	0	0	0	No	
NM_hsa_4081	chr7:121945711-121945807	+	CGGTTGCCGGTGTCTGCAAG	No	Yes	No	0	0	0	No	

Additional file 5

Name	locus	strand	sequence	mirdeep2	mireap	miranalyzer	RPM_HU_KapB_cMyc	RPM_HU_KaposinB	RPM_HUVEC	IP_confidence	annotation
NM_hsa_4088	chr7:134220449-134220518	+	CCGCTGTGATGTTATCTGAGG	No	Yes	No	0	0	0	No	
NM_hsa_4089	chr7:134855552-134855624	+	CTCAGTCGGAGGTCCGCGGG	No	Yes	No	0	0	0	No	
NM_hsa_4091	chr7:138720942-138721027	+	CTGGACGTGGACTCAGGCGGG	No	Yes	No	0	0	0	No	
NM_hsa_4093	chr7:139876420-139876499	+	CGGGCGGCCGGCGGCGGCGG	No	Yes	No	0.0427393	0.087193	0.0183216	Yes	
NM_hsa_4094	chr7:143085697-143085782	+	GTGAGTCGGGCTGTGCTGGGC	No	Yes	No	0.0569857	0	0.0732862	Yes	
NM_hsa_4095	chr7:148638570-148638672	+	AGTTGGTCCGAGTGTTGTGGGTTATT	No	Yes	No	2.35541	1.76593	1.80773	Yes	
NM_hsa_4096	chr7:148638570-148638672	+	GCGCTTGACTAGCTTGCTGTTT	No	Yes	No	5.01474	3.50979	6.22933	Yes	
NM_hsa_4097	chr7:148762972-148763044	+	CTCTGCCTTTCCTGTGCCTGGA	No	Yes	No	0	0	0	No	
NM_hsa_4098	chr7:148845165-148845260	+	GACGCGCAGCGGGGTTGGGC	No	Yes	No	0	0	0	No	
NM_hsa_4099	chr7:149195257-149195333	+	TGCTGCCCGAGGTCCTGGGT	No	Yes	No	0	0.132445	0	No	
NM_hsa_4100	chr7:150783538-150783623	+	CGGGATCACGGCGCTGGGAAG	No	Yes	No	0	0	0	No	
NM_hsa_4106	chr7:157310013-157310102	+	GGTGAGTGGGTCGGGGGAGC	No	Yes	No	0	0	0	No	
NM_hsa_4107	chr7:157520512-157520600	+	CCTGCACTGCTGCATCTCTGG	No	Yes	No	0	0	0	No	
NM_hsa_4110	chr7:1488540-1488624	-	TGCAATGATGGGTAAGCTGAGG	No	Yes	No	0.0569857	0	0.219859	No	
NM_hsa_4112	chr7:15962506-15962596	-	AGTGTGCGGAAATGCAACTGA	No	Yes	No	0	0	0	Yes	
NM_hsa_4113	chr7:26331600-26331683	-	GGCGCGCGGCGGCAGCGGCGG	No	Yes	No	0.186787	0.163717	0.0183216	Yes	KapB+cMyc>KapB>H
NM_hsa_4114	chr7:29198723-29198792	-	CAGGAGTCTGCACAATGCTT	No	Yes	No	0	0	0	No	
NM_hsa_4115	chr7:37823130-37823210	-	CAGAACCCAGAGAGGCAGCCTG	No	Yes	No	0	0	0	No	
NM_hsa_4119	chr7:73228271-73228350	-	AAGCCCAGAAGCTTGAAGCTGCAG	No	Yes	No	0	0	0	No	
NM_hsa_4120	chr7:74807665-74807744	-	CATCTGCCTCCTCTACCCCTGC	No	Yes	No	0	0	0	Yes	
NM_hsa_4121	chr7:91763389-91763469	-	GGGCGCGCGCCGCAGCTGAG	No	Yes	No	0.341914	0.728448	0.439717	Yes	
NM_hsa_4122	chr7:92165483-92165576	-	TGCCTCAGTTCAGCTGTAAC	No	Yes	No	0	0	0	No	
NM_hsa_4123	chr7:97910948-97911033	-	GGCGGGGCGGGCGCGAGCCGCG	No	Yes	No	0	0	0	No	
NM_hsa_4124	chr7:99063655-99063748	-	CCCAGGTAAGGCTGTTTGGA	No	Yes	No	0	0	0	No	
NM_hsa_4126	chr7:99691220-99691311	-	TGGTCTCCCTCACAGGACAGC	No	Yes	No	0	0	0	Yes	
NM_hsa_4127	chr7:105924959-105925045	-	GGCGCGCGGCGGCGGCGGCGCG	No	Yes	No	0.265933	0.362384	0.0427503	Yes	
NM_hsa_4128	chr7:107301483-107301573	-	TCCCCAAGAGGCGGACGCTG	No	Yes	No	0	0	0	No	
NM_hsa_4130	chr7:128784777-128784872	-	CTCCTCCCCGGAGGCGGCCGG	No	Yes	No	0.740814	0.860893	0.146572	No	
NM_hsa_4135	chr7:142157342-142157418	-	TCGGCTGTGTATCTCTGTGCC	No	Yes	No	0	0	0	No	
NM_hsa_4136	chr7:142625031-142625113	-	CTGGGCCTGAGACACTTCCC	No	Yes	No	0	0	0	No	
NM_hsa_4139	chr7:151216355-151216444	-	GGTCGGGGCGGCGTCCGCGCC	No	Yes	No	0	0	0	No	
NM_hsa_4140	chr7:152133530-152133619	-	GCGAGTTGGAGAGGTCTGTGG	No	Yes	No	0	0	0	No	
NM_hsa_4141	chr7:156411736-156411832	-	GGCCAGTCGGTCCAGAGAGC	No	Yes	No	0	0	0	No	
NM_hsa_4142	chr7:158763287-158763377	-	GGTCAGCATTGCAGCTGAGGGG	No	Yes	No	0	0	0	No	
NM_hsa_4143	chr8:9760899-9760980	+	TCAAGGTCCGCTGTGAACACGG	No	Yes	No	0	0	0	No	
NM_hsa_4145	chr8:12584738-12584821	+	CTGCTTTTTGGCCAGCTCCATT	No	Yes	No	0	0	0	No	
NM_hsa_4147	chr8:27325578-27325662	+	TGTGGCAGTAGGTTGTGTTA	No	Yes	No	0	0	0.0732862	Yes	
NM_hsa_4150	chr8:40884866-40884937	+	GTAGCCAGCTGAAGAACTGT	No	Yes	No	0.142464	0.165556	0.219859	Yes	H>KapB>KapB+cMyc
NM_hsa_4151	chr8:41353302-41353395	+	TAGCAAAATTTGAAATCTGTT	No	Yes	No	0	0	0	No	
NM_hsa_4155	chr8:54935841-54935938	+	AATCGTTCGTGAGCTCCTGGA	No	Yes	No	0	0	0	No	
NM_hsa_4156	chr8:56884019-56884091	+	CACACTTGTACAGGGCTATG	No	Yes	No	0	0	0	No	
NM_hsa_4157	chr8:67025521-67025598	+	TTCGGTTGCCTGGACTAGCG	No	Yes	No	0	0	0	No	
NM_hsa_4158	chr8:70604046-70604143	+	TGTGTACTGAATACCATAGGC	No	Yes	No	0	0	0	No	
NM_hsa_4159	chr8:71951311-71951394	+	CTAAGGCTGAGAGGGCTCTGA	No	Yes	No	0	0	0	No	
NM_hsa_4160	chr8:84333363-84333434	+	TTAACATTGGCTAGGCTAAGC	No	Yes	No	0	0	0	No	
NM_hsa_4161	chr8:99879737-99879816	+	TCTACTACACTACCCTGCCTCA	No	Yes	No	0	0	0	No	
NM_hsa_4162	chr8:102218079-102218167	+	GACGTCGCCTTCTGTCCCCAG	No	Yes	No	0	0	0	No	
NM_hsa_4163	chr8:102218345-102218429	+	CGCGGGGCCGCGGTGGCTGAG	No	Yes	No	0	0	0	No	
NM_hsa_4164	chr8:102218345-102218429	+	CCAGGACACCGCGGCCCCGGC	No	Yes	Yes	0	0	0.0732862	No	
NM_hsa_4164	chr8:102218323-102218459	+	CCAGGACACCGCGGCCCCGGC	No	Yes	Yes	0	0	0.0732862	No	
NM_hsa_4166	chr8:103425233-103425322	+	TTTCGCTCGGATTCCCCTGGGGC	No	Yes	No	0.142464	0.198668	0.0732862	Yes	
NM_hsa_4167	chr8:103818897-103818987	+	CAGGGCGGGCGGCGCTGGGA	No	Yes	No	0	0	0	No	
NM_hsa_4168	chr8:104008494-104008562	+	CTTCAGCATTGCCTCTGAGG	No	Yes	No	0	0	0	No	
NM_hsa_4169	chr8:106155317-106155413	+	TGAAACTGACCAGATAGTCCAC	No	Yes	No	0	0	0	No	
NM_hsa_4170	chr8:125486958-125487033	+	GCGGTTCGCTTGACTGACGG	No	Yes	No	0	0	0	No	
NM_hsa_4171	chr8:126473402-126473472	+	GACCCGAATGCCATAGGCTGT	No	Yes	No	0	0	0	No	
NM_hsa_4174	chr8:134058714-134058802	+	CAGCAGAACTGGGAGTGTGG	No	Yes	No	0	0	0	No	
NM_hsa_4175	chr8:134145639-134145723	+	TTTCTTTCTTCTCATTTGCCCAG	No	Yes	No	0	0	0	No	
NM_hsa_4176	chr8:143823097-143823173	+	CCATCCCACCTGCTGCCTTT	No	Yes	No	0	0	0	No	
NM_hsa_4177	chr8:144905786-144905867	+	CTGGAATGCTCTGGAGGCGGG	No	Yes	No	0	0	0	No	
NM_hsa_4178	chr8:144911634-144911728	+	CGCGGGACTCGAGTCGGGCG	No	Yes	No	0	0	0	No	
NM_hsa_4179	chr8:145169753-145169844	+	GGTTGGGCCGGCAGGGGCCGG	No	Yes	No	0	0	0	No	
NM_hsa_4185	chr8:146052940-146053013	+	TCCTCAGGGAGGGTGAGTCGG	No	Yes	No	0	0	0	No	
NM_hsa_4186	chr8:6333106-6333174	-	CTGGGGATAGAGCATTGCCCT	No	Yes	No	0.113971	0.132445	0	Yes	
NM_hsa_4187	chr8:11334673-11334739	-	GCCTTGGGGACCGGCTCTGTGG	No	Yes	No	0	0	0	No	
NM_hsa_4188	chr8:11840580-11840671	-	CAAAGTGCTTAGATTGCAGGC	No	Yes	No	0	0	0	Yes	
NM_hsa_4192	chr8:25986983-25987052	-	CACTGAAGATGTTAAGACAG	No	Yes	No	0	0	0	No	
NM_hsa_4194	chr8:37264509-37264593	-	GCAGCCCAGCTGAGGACACTGA	No	Yes	No	0	0	0	No	
NM_hsa_4195	chr8:38239233-38239325	-	GCGCGCGCGCGTGTGTAGTGC	No	Yes	No	0	0	0	No	
NM_hsa_4199	chr8:56028279-56028346	-	TGAGATGAAGCTCTGTCACC	No	Yes	No	0	0	0	No	
NM_hsa_4201	chr8:56418717-56418789	-	TGCCCTGTGGACTGAGAGAA	No	Yes	No	0	0	0	No	
NM_hsa_4202	chr8:68522861-68522955	-	AACATGGTTCCCTTCACAGTGT	No	Yes	No	0	0	0	No	
NM_hsa_4204	chr8:81781151-81781229	-	TGCATTACACTACGTTGCCTCT	No	Yes	No	0	0	0	No	
NM_hsa_4205	chr8:87178202-87178292	-	TTGTAGACTATGGGAAGTAG	No	Yes	No	0	0	0	No	
NM_hsa_4207	chr8:94955044-94955121	-	GTAAAGATGGCAGAGCACAG	No	Yes	No	0	0.0662225	0	No	
NM_hsa_4210	chr8:124926723-124926804	-	AACATTCATTGCTGTGCATGG	No	Yes	No	0	0	0	Yes	
NM_hsa_4212	chr8:133754177-133754260	-	TCCTTGGTCTAGTGGTGACG	No	Yes	No	0	0	0	Yes	
NM_hsa_4215	chr8:141519339-141519416	-	ACAAGTCAGGCTCTAGGGAG	No	Yes	No	0.142464	0.231779	0.219859	Yes	
NM_hsa_4218	chr8:142583597-142583684	-	GAGTGAAGCATAGGCCTGTG	No	Yes	No	0	0	0	No	
NM_hsa_4221	chr9:15306372-15306462	+	ACCAAAGGAGGCCGGAGTCGG	No	Yes	No	0	0	0	No	
NM_hsa_4223	chr9:34126774-34126847	+	CTCTGGTGTTTCCCGGCCGC	No	Yes	No	0	0	0	No	
NM_hsa_4224	chr9:35489881-35489955	+	CTCTGCGCGTGACTCGGCTGC	No	Yes	No	0	0	0	No	
NM_hsa_4225	chr9:35728433-35728528	+	ATTTAGAGCTGGACTGGGCAG	No	Yes	No	0	0	0	No	
NM_hsa_4227	chr9:35749111-35749194	+	CAGGTGCCAGCCCGTGGGAAG	No	Yes	No	0	0	0	No	
NM_hsa_4228	chr9:35755370-35755448	+	TCCTGGCATAGACCTCTGCACC	No	Yes	No	0	0	0	No	
NM_hsa_4229	chr9:35756452-35756530	+	CCGTTTCAGAGGATTGCCAAGA	No	Yes	No	0	0.0662225	0	No	
NM_hsa_4231	chr9:35829191-35829277	+	GAGCCGGCCGGGGGGCGGGGC	No	Yes	No	1.1967	1.52312	0.732862	No	
NM_hsa_4234	chr9:39894541-39894618	+	TCTCACCAGGAGCCTGGCACTGA	No	Yes	No	0	0	0	No	
NM_hsa_4235	chr9:43128014-43128100	+	AAAAGGAGAACTTTGAAGGA	No	Yes	No	2.50737	1.32445	3.95746	Yes	
NM_hsa_4236	chr9:72287446-72287532	+	TCCGTGGGCCGCCGGCTGCAG	No	Yes	No	0	0	0	No	
NM_hsa_4237	chr9:73035324-73035409	+	CTGGGTCGAGACGTGTGTAGG	No	Yes	No	0	0	0	No	
NM_hsa_4238	chr9:86535927-86536016	+	TGCGCTCTGAAAGGCCGGGTCC	No	Yes	No	0.170957	0.132445	0.293145	Yes	
NM_hsa_4241	chr9:91768732-91768812	+	GCTCAGACTGCGGACCTTCAG	No	Yes	No	0.113971	0	0	No	
NM_hsa_4242	chr9:94877793-94877882	+	GACGGGGTCAGGTGGGATGC	No	Yes	No	0	0	0	No	
NM_hsa_4243	chr9:95862071-95862170	+	CCAGATGATGGAGTGGCTGATGG	No	Yes	No	0	0.198668	0	No	
NM_hsa_4244	chr9:96421763-96421829	+	TGGGTCCGGTTGTCCTGCAG	No	Yes	No	0	0	0	No	
NM_hsa_4251	chr9:98117997-98118083	+	AGAATTGTGGCTGGAAATCTGA	No	Yes	No	0	0	0	No	
NM_hsa_4252	chr9:99181138-99181216	+	GCCCGGCGGCTGGAGTCTCTG	No	Yes	No	0	0	0.0732862	No	
NM_hsa_4253	chr9:100168900-100168979	+	TTTCCAGTGTGCTGTGGCTGT	No	Yes	No	0	0	0	No	
NM_hsa_4254	chr9:101314705-101314780	+	AAAGGCCCCAGGCTGTGTGT	No	Yes	No	0	0	0	No	
NM_hsa_4255	chr9:108633988-108634061	+	AACTGGGTTGGCAGAATCAG	No	Yes	No	0.341914	0.331113	0.219859	No	KapB+cMyc>KapB>H
NM_hsa_4256	chr9:110861842-110861932	+	CTTCATAGGGATGCACTTTT	No	Yes	No	0	0	0	No	
NM_hsa_4257	chr9:112542486-112542577	+	GCGCGGCCGGCGGCGGCGGCGA	No	Yes	No	0.0174123	0	0	Yes	
NM_hsa_4260	chr9:117072741-117072838	+	TGCACTTTTCCTTCTGCCTTCAG	No	Yes	No	0	0	0	No	
NM_hsa_4262	chr9:127117172-127117246	+	CGTGCCAGTGGACTGCAAGAC	No	Yes	No	0	0	0	No	
NM_hsa_4269	chr9:131343169-131343262	+	CCATGTCTCCTATGCCCCCAAG	No	Yes	No	0	0	0	No	
NM_hsa_4270	chr9:132094650-132094732	+	CAGGACTTAGAGGAACTCCGG	No	Yes	No	0	0	0	No	
NM_hsa_4273	chr9:134153375-134153457	+	TCCGCAGGCGGCGGCGGCGGCGGC	No	Yes	No	0.205757	0.137716	0.0374307	Yes	KapB+cMyc>KapB>H
NM_hsa_4274	chr9:136266010-136266096	+	TTCCTGATGATCCTGCCAGT	No	Yes	No	0	0	0	No	
NM_hsa_4275	chr9:136664003-136664097	+	CCAGGGCAGGCTCTTGGGACC	No	Yes	No	0	0	0	No	
NM_hsa_4276	chr9:137389666-137389764	+	ATTTTATTTGTTTTGATGATGG	No	Yes	No	0	0	0	No	
NM_hsa_4278	chr9:138296633-138296722	+	GATTCCACTTCTCCCGGCCTG	No	Yes	No	0.0189952	0.0441483	0.0244288	Yes	
NM_hsa_4280	chr9:139159167-139159256	+	TCACCCTCGTCGATGCTCCAG	No	Yes	No	0	0	0	No	
NM_hsa_4281	chr9:139434407-139434502	+	GCAGGGCTAGTAGGAATTCTG	No	Yes	No	0	0	0	No	
NM_hsa_4283	chr9:139566289-139566387	+	CACTGCCCTTCTGCACCCACAG	No	Yes	No	0.968757	0.463558	0.439717	No	KapB+cMyc>KapB>H
NM_hsa_4285	chr9:14315503-14315574	-	CGCGCGCGCCCGCCGGCCCGC	No	Yes	No	0	0	0	No	
NM_hsa_4287	chr9:28641358-28641446	-	TTCTGTAGTAGGTGATGTGA	No	Yes	No	0	0	0	No	
NM_hsa_4288	chr9:29035553-29035629	-	TACCTTGGTTTTGACTTCAAG	No	Yes	No	0	0	0	No	
NM_hsa_4289	chr9:40524378-40524458	-	GTAGTGTTTCCTACTAATTG	No	Yes	No	0	0	0	Yes	
NM_hsa_4290	chr9:45231132-45231202	-	GTACAGTAGTGTGATCACTGC	No	Yes	No	0	0	0	No	
NM_hsa_4291	chr9:69000992-69001070	-	TTCTGGAATTCTGTGTGAGAG	No	Yes	No	0	0	0	Yes	
NM_hsa_4296	chr9:91925631-91925727	-	AGGGCTTTGAGCGGAAGCTG	No	Yes	No	0	0	0	No	
NM_hsa_4297	chr9:96938234-96938324	-	CTATACAACCTACTACCTCA	No	Yes	No	0	0.0220742	0.097715	Yes	H>KapB>KapB+cMyc
NM_hsa_4298	chr9:111142835-111142919	-	ATGTGAAATTCTTGGAACAG	No	Yes	No	0	0	0	No	
NM_hsa_4300	chr9:113652880-113652959	-	TGAGGGAGTAGTTTTTGCTGG	No	Yes	No	0.256436	0.667744	0.207644	Yes	
NM_hsa_4302	chr9:114245620-114245699	-	CGGTGGGCGGGCGGCGGCCGC	No	Yes	No	0.0284929	0	0	No	
NM_hsa_4303	chr9:116037812-116037897	-	CCGCCCCCGGAGCGGCGCGG	No	Yes	No	0	0	0	No	
NM_hsa_4304	chr9:124445662-124445735	-	CCTCCCACACCCCAGGCCTGCCA	No	Yes	No	0.0854786	0	0	No	
NM_hsa_4305	chr9:126232637-126232735	-	CTTTCTTGGGACTTTGGATGCA	No	Yes	No	0	0	0	No	
NM_hsa_4308	chr9:131619711-131619796	-	GCTGAGTGAAGCGGTGGCCTGT	No	Yes	No	0	0	0	No	
NM_hsa_4310	chr9:139492760-139492848	-	ACTGCCCCAGGTGCTCCTGGTGT	No	Yes	No	1.57661	1.51208	1.63673	Yes	
NM_hsa_4311	chr9:140917988-140918076	-	CCGCGCGCGCCGCTCGCCCT	No	Yes	No	0	0	0	No	
NM_hsa_4312	chrM:8153-8229	-	TAGGACGATGGGCATGAAACTGTG	No	Yes	No	0.0569857	0.198668	0	Yes	
NM_hsa_4313	chrM:10965-11049	-	CATGATTGTGAGGGGTAGGA	No	Yes	No	0.113971	0.0662225	0	No	KapB+cMyc>KapB>H
NM_hsa_4314	chrM:11417-11516	-	ACTATTGACCCAGCGATGGGGGC	No	Yes	No	0.113971	0.198668	0.0732862	No	
NM_hsa_4315	chrX:70177-70250	+	CGCAGGTCGGTGGGACTGAGC	No	Yes	No	0	0	0	No	
NM_hsa_4316	chrX:7065812-7065905	+	GCGGCCACGCGCGCCCGCCAG	No	Yes	No	0	0	0	No	
NM_hsa_4317	chrX:9023522-9023617	+	TTCAAGCATTTCAAGATAGGTT	No	Yes	No	0	0	0	No	
NM_hsa_4318	chrX:9525128-9525203	+	ACTCTGGCTGCCACCAATGG	No	Yes	No	0	0	0	No	
NM_hsa_4319	chrX:15618895-15618989	+	AACTTTGATAGAACAGGTCTT	No	Yes	No	0	0	0	No	
NM_hsa_4321	chrX:29628076-29628163	+	AAATTAATTTGAAGAGAGAGT	No	Yes	No	0	0	0	No	
NM_hsa_4322	chrX:37706895-37706965	+	ACGCCAGGCGTCACTTCAGGC	No	Yes	No	0	0	0	No	
NM_hsa_4323	chrX:38533408-38533480	+	TTGGAGTCAGTTTGGGCTGTGG	No	Yes	No	0	0	0	No	
NM_hsa_4325	chrX:40249043-40249127	+	TCGGCCTTTGTACTCTGAGA	No	Yes	No	0.0569857	0	0.0732862	No	
NM_hsa_4327	chrX:46988951-46989039	+	CTGGAAAGAAGCTCTGAGCC	No	Yes	No	0	0	0	No	
NM_hsa_4328	chrX:48674797-48674881	+	GTGCTCCTCTCTGTGCTTCCAG	No	Yes	No	0	0	0	No	
NM_hsa_4329	chrX:48793356-48793445	+	TCGGGGAGCACACAGTCTAGTAAA	No	Yes	No	58.4764	80.6336	63.4097	Yes	
NM_hsa_4338	chrX:52964418-52964514	+	ATCGGGGTTCGCCGCAGGCT	No	Yes	No	0	0	0.0732862	Yes	
NM_hsa_4339	chrX:54665906-54665974	+	CCAAAAGAGTCGGAAAGGGG	No	Yes	No	0	0	0	No	
NM_hsa_4343	chrX:71476657-71476739	+	TCAGGCTCTGGCATGTTGGA	No	Yes	No	1.1967	1.32445	0.659576	Yes	
NM_hsa_4344	chrX:76539389-76539484	+	CATCTCACTGGGCAGTGCTGGA	No	Yes	No	0	0	0	No	
NM_hsa_4345	chrX:102024072-102024150	+	AGGCGTTTGGACTCAGTGGA	No	Yes	No	0	0	0	No	
NM_hsa_4346	chrX:107740993-107741070	+	AATTGTTACTCTGAAACCTGC	No	Yes	No	0	0	0.0732862	Yes	
NM_hsa_4351	chrX:122560401-122560483	+	TTTCTCAGTTTGGTCTGCAGA	No	Yes	No	0	0	0	No	
NM_hsa_4353	chrX:135138800-135138877	+	CTCAAGGAGCTCACAGTCTAGT	No	Yes	No	752.37	755.256	568.002	Yes	
NM_hsa_4355	chrX:143379240-143379305	+	ATCTGGGACCCAGATGGAAGG	No	Yes	No	0	0	0	No	
NM_hsa_4358	chrX:153626675-153626756	+	TTCGGTGTGGTGAGTAAGCG	No	Yes	No	0	0	0	No	
NM_hsa_4360	chrX:153697105-153697175	+	TGCACCTGTCCCTGGCTGCAG	No	Yes	No	0	0	0	Yes	

Additional file 5

Name	locus	strand	sequence	mirdeep2	mireap	miranalyzer	RPM_HU_KapB_cMyc	RPM_HU_KaposinB	RPM_HUVEC	IP_confidence	annotation
NM_hsa_4361	chrX:153707368-153707433	+	TCCCTGGAGAGCCTGACTGG	No	Yes	No	0	0	0.0732862	No	
NM_hsa_4362	chrX:153996835-153996965	+	ATGGGCTAAGGGAGATGATTGGG	No	Yes	Yes	0.113971	0	0	Yes	
NM_hsa_4366	chrX:6301935-6302015	-	TGAGATGACACTGTAGCTCCC	No	Yes	No	0	0	0	No	
NM_hsa_4367	chrX:8541654-8541730	-	CTGGAAGGTATTTTTTGAGT	No	Yes	No	0	0	0	No	
NM_hsa_4368	chrX:10826203-10826301	-	CATGAGATTGTGAGCTCCCTGA	No	Yes	No	1.08273	1.25823	0.366431	No	
NM_hsa_4369	chrX:11846734-11846823	-	TCTCTCAGTTCAGCAGCAGC	No	Yes	No	0	0	0	No	
NM_hsa_4370	chrX:13279750-13279817	-	TTTTTCACCTTTTGCAGGGAA	No	Yes	No	0	0	0	No	
NM_hsa_4373	chrX:29640709-29640797	-	TCTTGGGTTATCTAACTGTT	No	Yes	No	0	0	0	No	
NM_hsa_4374	chrX:38420689-38420787	-	GGCGGCGGCGGCAGGCGGCGGGCG	No	Yes	No	0.455886	0.364224	0.366431	Yes	
NM_hsa_4383	chrX:87333951-87334026	-	TCTGGACTCTGAATCTAGTAC	No	Yes	No	0.170957	0.176593	0.146572	Yes	
NM_hsa_4384	chrX:114984273-114984348	-	CAGATGGGACTCGGCGGGAC	No	Yes	No	0.0569857	0.198668	0.146572	Yes	
NM_hsa_4385	chrX:125715185-125715267	-	GGGACGGGGTCCGGGGAGCG	No	Yes	No	0	0	0	No	
NM_hsa_4393	chrX:134562082-134562157	-	GGAATACCAGGTGCTATAGGCTT	No	Yes	No	0.0596993	0.154519	0.0244288	Yes	
NM_hsa_4394	chrX:139664947-139665039	-	TCCCTGAGACACTTCATCCTG	No	Yes	No	0	0	0	Yes	
NM_hsa_4395	chrX:140008324-140008398	-	AAAAGCTGGGTTGAGAGGAGCA	No	Yes	No	0.9608	1.14841	0.694794	Yes	
NM_hsa_4398	chrX:147582246-147582344	-	GGGCCGGCGGCGGCAGCGGCGG	No	Yes	No	0.0261185	0.00965745	0.00916078	No	KapB+cMyc>KapB>H
NM_hsa_4399	chrX:149862037-149862125	-	GCCCCGGGTTCGGGCCCCCGC	No	Yes	No	0	0	0	No	
NM_hsa_4403	chrX:153030836-153030932	-	GCGGCGGTGGGGGTGGCGCC	No	Yes	No	0	0	0	No	
NM_hsa_4404	chrX:153129925-153130000	-	CTGCCACCCTCTCTCCCTGGC	No	Yes	No	0	0	0	No	
NM_hsa_4406	chrX:153665182-153665279	-	CCCCGCCCCGCCCCGCGCGCG	No	Yes	No	0.113971	0.0662225	0.0732862	Yes	
NM_hsa_4408	chrY:20111101-20111190	+	AGAGTGGCAATGGTGTTTGA	No	Yes	No	0	0	0	No	
NM_hsa_4409	chrY:8979486-8979564	-	GAGGTTGCGGTCTGTTAGTA	No	Yes	No	0	0	0	No	
NM_hsa_4410	chrY:14320395-14320483	-	GTTCAGATGGCAGGGCCCGT	No	Yes	No	0	2.18534	1.97873	No	
NM_hsa_4411	chr20:23078388-23078530	-	CTTTGCCAGATCCTTGCACGGGTC	No	No	Yes	0	0	0	No	
NM_hsa_4411	chr20:23078387-23078529	+	CTTTGCCAGATCCTTGCACGGGTC	No	No	Yes	0	0	0	No	
NM_hsa_4413	chr20:37352999-37353127	-	CGGAGGGCGGCGGCGGCGGCGGCGGCG	No	No	Yes	3.61319	3.65229	2.55481	Yes	
NM_hsa_4415	chr20:37078067-37078169	+	AAATGCTGGCGCTTTCACACACTC	No	No	Yes	0	0.0662225	0.146572	Yes	H>KapB>KapB+cMyc
NM_hsa_4418	chr20:29611893-29611987	+	GCGGGTTCTGGACAGACGTAGGCTG	No	No	Yes	0	0	0	No	
NM_hsa_4419	chr20:2520506-2520624	+	AGGAGTGGGCTAAGGAAACCTG	No	No	Yes	0	0	0	No	
NM_hsa_4421	chr20:23078404-23078530	+	CAAGGATCTGGCAAAGCTGAG	No	No	Yes	0	0	0	No	
NM_hsa_4421	chr20:23078387-23078513	-	CAAGGATCTGGCAAAGCTGAG	No	No	Yes	0	0	0	No	
NM_hsa_4422	chr20:43272688-43272826	-	AATCTGACTAGGTTTGCCTTGG	No	No	Yes	0	0	0	No	
NM_hsa_4423	chr20:60941141-60941277	+	CTAGAGCGGCCGGTTGGGAGC	No	No	Yes	0	0	0	No	
NM_hsa_4424	chr20:30194977-30195059	+	GCTCCTGCGTAGGATCTGAGGAGT	No	No	Yes	0.427393	1.26926	0.0732862	Yes	
NM_hsa_4425	chr20:2634868-2635014	+	CTGATGTCTCCATGTCTCTGAGCAAT	No	No	Yes	0.512871	0.463558	0.293145	Yes	KapB+cMyc>KapB>H
NM_hsa_4426	chr20:2636705-2636851	+	TCACGGTGATGGCTGACCAGGGCTCC	No	No	Yes	0.170957	0.132445	0.513004	Yes	
NM_hsa_4428	chr20:4795074-4795178	+	GTGCTATAGGCTGAGACTCTGAGCT	No	No	Yes	0	0	0	No	
NM_hsa_4429	chr20:34204893-34205025	+	GCTTGTGGACTGAGCAATCTGCGG	No	No	Yes	0.0569857	0	0	No	
NM_hsa_4430	chr20:37078061-37078169	+	TTCAAATGCTGGCGCTTTCACA	No	No	Yes	0	0	0	Yes	
NM_hsa_4431	chr20:37078062-37078162	+	TTGCTGAAAGCCGTTTCCCGTGTTTGGA	No	No	Yes	0.341914	0.52978	0	Yes	
NM_hsa_4432	chr20:48330494-48330588	-	CTGTGGGCGCCGAGCGAGGG	No	No	Yes	0	0	0	No	
NM_hsa_4433	chr20:23078398-23078506	+	TCAGCTTTGCCAGATCCTTGCA	No	No	Yes	0	0	0	No	
NM_hsa_4433	chr20:23078406-23078524	-	TCAGCTTTGCCAGATCCTTGCA	No	No	Yes	0	0	0	No	
NM_hsa_4435	chr21:9826466-9826570	+	GGCGCGACTGTCCCCGGGCCGGGCA	No	No	Yes	0.341914	0.728448	0.366431	Yes	
NM_hsa_4436	chr21:47705766-47705864	+	CCCTCGAGCGGGCTGGAAGGAC	No	No	Yes	0	0	0	No	
NM_hsa_4437	chr21:9827223-9827313	+	GGGCGCCGGCGGCTTTGGTGACTCTAGA	No	No	Yes	5.35666	2.58268	3.9941	Yes	
NM_hsa_4439	chr21:41935264-41935394	-	TCTGGTGGAACTCTGAGAATTTC	No	No	Yes	0	0	0	No	
NM_hsa_4440	chr21:41935273-41935393	+	ATTGATCTGGTGGAACTCTGAGA	No	No	Yes	0	0	0	No	
NM_hsa_4440	chr21:41935264-41935404	-	ATTGATCTGGTGGAACTCTGAGA	No	No	Yes	0	0	0	No	
NM_hsa_4441	chr21:9825733-9825811	+	GGGGGTCGGCCTGCGGCGCGTGCGGGG	No	No	Yes	0	0	0.219859	Yes	
NM_hsa_4442	chr21:9826730-9826870	+	CGGTGGTCGTGTCGCGTGGGGGGCGGGT	No	No	Yes	0.113971	0	0	Yes	
NM_hsa_4443	chr21:9825637-9825739	+	GGCGTCCGCGTCCCCCGGCGCGCG	No	No	Yes	0	0	0	No	
NM_hsa_4444	chr21:9826227-9826315	+	CGCCTTGCCGGTCGCTCGCCCT	No	No	Yes	0.113971	1.12578	0.879435	Yes	
NM_hsa_4445	chr21:9826410-9826542	+	CGCGCCCGCCGGCGGGGCTCGGAGCCGGG	No	No	Yes	0.512871	0.52978	1.09929	Yes	H>KapB>KapB+cMyc
NM_hsa_4446	chr21:46707943-46708053	+	CCGGAGGCCGTGGCTGTGGTTCG	No	No	Yes	0	0	0	No	
NM_hsa_4447	chr21:9826183-9826279	+	CGGACAGGCGTTCGTGCGACGTGTGG	No	No	Yes	0.0142464	0.0220742	0	Yes	
NM_hsa_4449	chr21:9826194-9826274	+	ACAGGCGTTCGTGCGACGTGTGGCGTGG	No	No	Yes	0.0142464	0	0.0732862	Yes	
NM_hsa_4450	chr21:9826690-9826778	+	CGCGGGTCTGTGGCGCGGGGCC	No	No	Yes	0.341914	0.331113	0.0732862	Yes	KapB+cMyc>KapB>H
NM_hsa_4451	chr21:36824232-36824374	+	CATGAAACTCTGAGCAAGAACTCC	No	No	Yes	0	0	0	No	
NM_hsa_4452	chr21:9826743-9826819	+	GGCGCGGGGCCCCGGTGGTCG	No	No	Yes	0	0	0	Yes	
NM_hsa_4453	chr21:9827210-9827320	+	GGCGGGCGCCGGCGGCTTTGGTGACTCT	No	No	Yes	2.05149	1.4569	1.13594	Yes	KapB+cMyc>KapB>H
NM_hsa_4454	chr21:9826739-9826871	+	CGCGCCCCGCCCCGGCCCCACCGG	No	No	Yes	0	0.0662225	0	No	
NM_hsa_4455	chr21:9826749-9826881	+	GGGGCGGGTGGTTGGGGCGTCCGG	No	No	Yes	0	0.132445	0	Yes	
NM_hsa_4456	chr21:34103267-34103389	+	ATCAGGATCAGGATCTGCAGAGTT	No	No	Yes	0	0	0	Yes	
NM_hsa_4457	chr21:36824231-36824369	+	TTGCATGAAACTCTGAGCAAGA	No	No	Yes	0	0	0	No	
NM_hsa_4458	chr21:9827328-9827406	+	GGCGGCGACGACCCATTCGAACGTCTG	No	No	Yes	1.02574	0.662225	1.39244	Yes	
NM_hsa_4459	chr21:9827260-9827348	+	GGCTGATCGCACGCCCCCCGTGGCGGC	No	No	Yes	1.82354	2.0529	3.44445	Yes	H>KapB>KapB+cMyc
NM_hsa_4461	chr21:9826538-9826630	+	GCGGTCCGCCTCTCGCTCGCCGCC	No	No	Yes	0.113971	0	0	No	
NM_hsa_4463	chr21:45622813-45622913	+	TCTCGGCCTGTGGGCACTGAGCA	No	No	Yes	0	0.0662225	0	No	
NM_hsa_4464	chr21:9826263-9826411	+	GGTCGCGGTCCCCCGTCCCGGGCGGGG	No	No	Yes	0	0.0662225	0.146572	No	H>KapB>KapB+cMyc
NM_hsa_4465	chr21:46707948-46708054	+	GAGGCCGTGGCTGTGGTTCGCGGCGG	No	No	Yes	0.227943	0.132445	0.146572	No	
NM_hsa_4466	chr21:9826061-9826141	+	CTTCCGAACCGGGTCGGCGCGTCCCCCG	No	No	Yes	1.1967	1.65556	2.34516	Yes	H>KapB>KapB+cMyc
NM_hsa_4467	chr21:9826401-9826533	+	CCGGGCCCCGGGCCCTCGACCGGG	No	No	Yes	0.0569857	0	0	No	
NM_hsa_4468	chr21:46853360-46853498	+	ACTTTGCAGGTCTCGGGTGGAT	No	No	Yes	0	0	0	No	
NM_hsa_4469	chr21:9825703-9825787	+	GGCCCGGTGGGCTTCCCGGAGGGTT	No	No	Yes	0	0	0	No	
NM_hsa_4472	chr22:44546487-44546593	+	CCTCAGTGGTCTGGGGTGCAGGCTTC	No	No	Yes	0.170957	0.463558	0.366431	Yes	
NM_hsa_4473	chr22:43011313-43011415	+	GGGATGCCTGGGAGTTGCGATCTGCCCGA	No	No	Yes	13.5911	10.706	7.86606	Yes	KapB+cMyc>KapB>H
NM_hsa_4474	chr22:50926212-50926354	+	CACTGCCTTCTCCTTCCCCGCCAG	No	No	Yes	0	0	0	No	
NM_hsa_4477	chr22:24360156-24360228	+	TTTCCAGATGGAGGGCCAGACGAA	No	No	Yes	0.227943	0.0662225	0.0732862	Yes	
NM_hsa_4478	chr22:50757084-50757212	+	CTCACCCCTGTGGCACTCTGGG	No	No	Yes	0	0	0	No	
NM_hsa_4479	chr22:21771619-21771717	+	GCGCTGGGCGGGCGGCTGTGAG	No	No	Yes	0	0	0	No	
NM_hsa_4479	chr22:21871746-21871844	-	GCGCTGGGCGGGCGGCTGTGAG	No	No	Yes	0	0	0	No	
NM_hsa_4479	chr22:20427772-20427870	-	GCGCTGGGCGGGCGGCTGTGAG	No	No	Yes	0	0	0	No	
NM_hsa_4481	chr22:50926216-50926334	+	AGGGGATGGAGAGGCCTGTGTG	No	No	Yes	0	0	0	No	
NM_hsa_4482	chr22:39097089-39097179	+	GACGCCGGGCGTGTAGGGGCGCA	No	No	Yes	0	0	0	No	
NM_hsa_4483	chr22:46973037-46973165	+	CCGGCTCCCCGGCGCCGCTGGG	No	No	Yes	2.05149	2.18534	0.366431	Yes	
NM_hsa_4484	chr22:46984181-46984311	+	TCCGTCCCTGTCTGGCTGTCCGT	No	No	Yes	0	0.0993338	0	Yes	
NM_hsa_4488	chr22:39097109-39097235	+	TTGCAAAGGCCGTGGGTGTGG	No	No	Yes	0	0	0	No	
NM_hsa_4489	chr22:46773606-46773734	-	TGAAGCTGACCCGCCTCTGCCC	No	No	Yes	0.0569857	0	0	Yes	
NM_hsa_4491	chr22:41487702-41487840	+	CTAGCCTGTAAGGAGGATTCGG	No	No	Yes	0	0	0	No	
NM_hsa_4492	chr5:138611848-138611954	+	CTCCAGCTGTAGGCAGCTGCCTAGGT	No	No	Yes	0.911772	0.397335	1.24587	No	
NM_hsa_4493	chr5:98268242-98268382	+	ACTGTTGGTCCTCTGTACCTGCA	No	No	Yes	0	0.198668	0	No	
NM_hsa_4495	chr5:34655660-34655748	+	AGAGAAGCTGTCGGGGACCTGG	No	No	Yes	0	0	0	No	
NM_hsa_4496	chr5:138614438-138614566	+	CATCCAGCGGTTGTCAGCTATCCAGGC	No	No	Yes	2.3934	2.38401	2.34516	Yes	KapB+cMyc>KapB>H
NM_hsa_4497	chr5:172447694-172447832	+	TTTCCAGCAGTAGTCAGCCATCCGGAC	No	No	Yes	0.911772	0.0662225	0.732862	Yes	
NM_hsa_4499	chr5:138611938-138612022	+	ACTACCCCAGGATGCCAGCATAGTT	No	No	Yes	5.69857	6.62225	6.88891	Yes	H>KapB>KapB+cMyc
NM_hsa_4500	chr5:180668786-180668912	-	GGACATGTCCTGCAATTCTGA	No	No	Yes	0	0	0	Yes	
NM_hsa_4501	chr5:51813670-51813770	-	GGGCGAACTGTCCCCAGTGCGAC	No	No	Yes	4.07448	3.57602	2.67495	Yes	KapB+cMyc>KapB>H
NM_hsa_4502	chr5:71146770-71146850	+	GGGAGCCCCGGGGAGAGTTCTCT	No	No	Yes	0.683829	0.52978	1.09929	Yes	
NM_hsa_4504	chr5:115257200-115257296	+	AGAACTGCCACCAGCTGTAGA	No	No	Yes	0	0	0	Yes	
NM_hsa_4505	chr5:173278543-173278643	+	GAGGGTCTGACTGTCACTTGGAG	No	No	Yes	0	0	0	Yes	
NM_hsa_4508	chr5:98109361-98109481	+	GGTGGTCGCTAGGGCTGGGCCAG	No	No	Yes	0	0	0	No	
NM_hsa_4511	chr5:102123184-102123282	+	ACAGATTTCCTGGGCTTAGTGA	No	No	Yes	0.0569857	0	0	No	
NM_hsa_4511	chr5:102123180-102123288	-	ACAGATTTCCTGGGCTTAGTGA	No	No	Yes	0.0569857	0	0	No	
NM_hsa_4513	chr5:167956344-167956468	-	AGGCGGGCGGCGGGCGGCGG	No	No	Yes	0.626843	0.507706	0.164894	No	KapB+cMyc>KapB>H
NM_hsa_4514	chr5:34537996-34538086	+	CAGGGACCCCTGACTGACAACGC	No	No	Yes	0	0.0551854	0	No	
NM_hsa_4515	chr5:34839460-34839560	+	GCTCTCAGAGCTCGACCTGCGGC	No	No	Yes	0	0	0	No	
NM_hsa_4520	chr5:141229323-141229429	+	CCCGGGAGCCGGACGGAGGAG	No	No	Yes	0	0	0	No	
NM_hsa_4523	chr5:93905141-93905261	-	GGTTGTAGTCCGTGCGAGAATAA	No	No	Yes	3.93202	7.01959	9.16078	Yes	H>KapB>KapB+cMyc
NM_hsa_4525	chr5:139166195-139166335	+	TGATTGGTTCAGGGCTGGGAGCA	No	No	Yes	0	0	0	Yes	
NM_hsa_4527	chr5:167592829-167592955	+	TCTCTCACCTGGCATAAGCAATTCAC	No	No	Yes	0	0	0	No	
NM_hsa_4528	chr7:73062127-73062227	+	GGCGGGCGGCGGGCGGCGGGGGC	No	No	Yes	0.645838	0.52978	0.164894	Yes	KapB+cMyc>KapB>H
NM_hsa_4528	chr5:167956295-167956425	-	GGCGGGCGGCGGGCGGCGGGGGC	No	No	Yes	0.645838	0.52978	0.164894	Yes	KapB+cMyc>KapB>H
NM_hsa_4529	chr5:98109363-98109473	+	GTGGGTGGTCGCTAGGGCTGGGC	No	No	Yes	0	0	0.0732862	No	
NM_hsa_4531	chr6:28757511-28757605	-	GCTTTGCATGTATGAGGTCCCGGGT	No	No	Yes	37.5536	29.2041	7.62177	Yes	KapB+cMyc>KapB>H
NM_hsa_4532	chr6:51425040-51425170	+	TGCCTAGATTTTGTCCTGTACTT	No	No	Yes	0	0	0	No	
NM_hsa_4532	chr6:51425036-51425176	-	TGCCTAGATTTTGTCCTGTACTT	No	No	Yes	0	0	0	No	
NM_hsa_4534	chr6:107780902-107781002	+	AGGTCGTCAGTGACAGGGATCAG	No	No	Yes	0	0	0	No	
NM_hsa_4535	chr6:28918850-28918954	+	GTTCGGGCGGGAGTGGTGGCTTTTA	No	No	Yes	2.27943	3.37735	3.40781	Yes	H>KapB>KapB+cMyc
NM_hsa_4537	chr6:134578178-134578322	+	TGGGAATACCAGGTGCTGTAGGCTT	No	No	Yes	603.059	474.894	380.171	Yes	KapB+cMyc>KapB>H
NM_hsa_4542	chr6:35465505-35465643	+	TTCCAGCTCGCGCCTCGGACGC	No	No	Yes	0	0	0	No	
NM_hsa_4545	chr6:13712186-13712264	+	GCAGGATCGCTGGGCTCCCGGC	No	No	Yes	0	0	0	No	
NM_hsa_4551	chr6:3069234-3069342	+	AGGTCGGGGCGCTGGACTGGTAGCGGG	No	No	Yes	0	0.0662225	0.146572	No	H>KapB>KapB+cMyc
NM_hsa_4552	chr6:31804817-31804957	+	TGGGAATGATGATTTCACAGACTAGAGT	No	No	Yes	0.113971	0.331113	0.146572	Yes	
NM_hsa_4553	chr6:39042071-39042183	+	TTTTTTTGCTGGAACATTTCTGGT	No	No	Yes	0	0	0	Yes	
NM_hsa_4554	chr6:27791829-27791941	+	CTTGCTCGTCATGTCTGGCCGCGG	No	No	Yes	0.0569857	0.198668	0.513004	No	H>KapB>KapB+cMyc
NM_hsa_4556	chr6:27095028-27095144	-	CGCTGCGGGCTGGAGAGCTGC	No	No	Yes	0	0.0993338	0	No	
NM_hsa_4557	chr6:36954298-36954420	+	CCGCGGCCTGCGTGGAACTCTGCG	No	No	Yes	0	0	0	No	
NM_hsa_4558	chr6:28697076-28697172	+	CGAGGCCCCGGGTTCGACCCCCGGCT	No	No	Yes	0	0.198668	0	Yes	
NM_hsa_4559	chr6:32164277-32164365	+	CCAGGAGACCACCGGCCTGCAG	No	No	Yes	0.113971	0	0	No	
NM_hsa_4560	chr6:30595314-30595452	+	TCTGACTTAATCTTCCCTGCAG	No	No	Yes	0.0569857	0	0	Yes	
NM_hsa_4561	chr6:107780899-107780999	+	CAAAGGTCGTCAGTGACAGGGAT	No	No	Yes	0	0	0	No	
NM_hsa_4562	chr6:53141758-53141896	-	CGTGATAACTGCTGGGCTGTGA	No	No	Yes	0	0.0662225	0	No	
NM_hsa_4563	chr6:2854245-2854361	+	ACCAGGCAAGAACTACTGTCT	No	No	Yes	3.989	3.04624	2.49173	Yes	KapB+cMyc>KapB>H
NM_hsa_4564	chr6:28663697-28663809	-	GTCAGGGGGTGTAGATCAGTGGTA	No	No	Yes	17.1717	27.6369	80.3217	Yes	H>KapB>KapB+cMyc
NM_hsa_4566	chr6:36954297-36954419	+	GCCGCGGCCTGCGTGGAACTCTGC	No	No	Yes	0	0	0	No	
NM_hsa_4567	chr6:2854232-2854370	+	GGAGACAATATTTCTTGCCTGG	No	No	Yes	0	0	0	No	
NM_hsa_4568	chr6:28795184-28795276	-	GGGGGTATAGCTCAGTGGTAGAGA	No	No	Yes	38.2279	46.8414	96.8844	Yes	H>KapB>KapB+cMyc
NM_hsa_4569	chr6:28697083-28697183	+	GGGTTCGACCCCCGGCTCCTCCA	No	No	Yes	0	0	0	Yes	
NM_hsa_4571	chr6:3069232-3069340	+	AGAGGTCGGGGCGCTGGACTGG	No	No	Yes	0	0.0662225	0	No	
NM_hsa_4573	chr6:10722977-10723093	-	CTGCGGGACCAGGGAATCTGA	No	No	Yes	0	0	0	No	
NM_hsa_4577	chr7:37824472-37824594	+	CAACAGAACCCAGAGAGTCAGCCT	No	No	Yes	0	0	0	Yes	
NM_hsa_4578	chr7:148638560-148638668	+	AGTTGGTCCGAGTGTTGTGGGTTATTG	No	No	Yes	6.51537	4.21617	3.42002	Yes	KapB+cMyc>KapB>H
NM_hsa_4579	chr7:139026479-139026587	+	CTGGTCTGTTGCCTCTGCGGAG	No	No	Yes	0	0	0	No	
NM_hsa_4583	chr7:72425176-72425296	-	GCGCGGGAACATGGGGCTGTACG	No	No	Yes	0	0	0	No	
NM_hsa_4583	chr7:75039611-75039731	+	GCGCGGGAACATGGGGCTGTACG	No	No	Yes	0	0	0	No	
NM_hsa_4586	chr7:17980164-17980312	+	TGACTTGTAGGACCCCGGGACCGGGCG	No	No	Yes	0.0569857	0.331113	0.146572	No	
NM_hsa_4587	chr7:31142092-31142210	+	AGGGGAACTCCAGGAGAGCTGC	No	No	Yes	0	0	0	Yes	
NM_hsa_4589	chr7:74438658-74438750	+	CATCTGCCTCCTCTACCCCTGCAC	No	No	Yes	0.0854786	0	0	Yes	
NM_hsa_4589	chr7:74807658-74807750	-	CATCTGCCTCCTCTACCCCTGCAC	No	No	Yes	0.0854786	0	0	Yes	
NM_hsa_4592	chr7:150783540-150783630	+	CGGGATCACGGCGCTGGGAAGCG	No	No	Yes	0	0	0	No	
NM_hsa_4594	chr7:98617315-98617421	+	ACATCAGAGCGCAGGACTGTGTCTGC	No	No	Yes	0	0	0.146572	Yes	
NM_hsa_4596	chr7:63560369-63560503	-	GCGAGGTCGGCTCCCCACCC	No	No	Yes	0	0	0	No	
NM_hsa_4599	chr7:5820747-5820885	+	GCGGCCTCGGGCCCGGGGGAGG	No	No	Yes	0.227943	0.596003	0.146572	Yes	
NM_hsa_4600	chr7:140772387-140772473	+	CCCGCGCCGGACGAGAGAGCG	No	No	Yes	0	0	0	No	
NM_hsa_4602	chr7:129414728-129414870	+	TTATGGCCCTTCGGTAATTCACTG	No	No	Yes	0.0569857	0.0662225	0.219859	Yes	H>KapB>KapB+cMyc
NM_hsa_4605	chr7:90225631-90225747	+	GCGGCGGCGCGGCGCGGGGCC	No	No	Yes	0	0	0	No	
NM_hsa_4607	chr8:128750358-128750486	+	AGAGGTTCGGGACTGTGGCGCG	No	No	Yes	0.0569857	0	0.366431	Yes	

Additional file 5

Name	locus	strand	sequence	mirdeep2	mireap	miranalyzer	RPM_HU_KapB_cMyc	RPM_HU_KaposinB	RPM_HUVEC	IP_confidence	annotation
NM_hsa_4608	chr8:21906691-21906789	+	GGGGCGGGAAGCGGGGTTGGGC	No	No	Yes	0	0	0	No	
NM_hsa_4610	chr8:65291688-65291828	-	TCAAGGTCCGCTGTGAACACGGA	No	No	Yes	0	0	0	No	
NM_hsa_4611	chr8:67026179-67026297	+	GCGGAGGACTGTAGGCGCGCGC	No	No	Yes	0	0	0	Yes	
NM_hsa_4614	chr8:11281071-11281177	+	GGGTGGCAGTGAAGAGGCTCT	No	No	Yes	0	0	0	No	
NM_hsa_4615	chr8:15403324-15403432	+	TTATGCCTGTGACTGCTGTAGA	No	No	Yes	0	0.198668	0.146572	No	
NM_hsa_4617	chr8:103425224-103425340	+	TTTCGCTCGGATTCCCCTGGGGCAGC	No	No	Yes	0.142464	0.198668	0.439717	Yes	H>KapB>KapB+cMyc
NM_hsa_4620	chr8:145584146-145584268	+	TGTGCGGGTGGAACTCAGGGCTGG	No	No	Yes	0.0569857	0.165556	0.146572	Yes	
NM_hsa_4622	chr8:109304621-109304715	-	CAGGACGGTGGCCATGGAAGTCGAA	No	No	Yes	17.3806	19.337	11.7991	Yes	
NM_hsa_4624	chr8:42752484-42752582	+	CTGGGCTGGGAACGCGGCGGGC	No	No	Yes	0	0	0	No	
NM_hsa_4628	chr8:145582150-145582286	-	CTGGTCGGCGGGCGGGCGGGC	No	No	Yes	0	0	0	No	
NM_hsa_4629	chr8:101322417-101322551	+	CCGCTGCGGGCGGCTCTGGACGCGG	No	No	Yes	0	0	0	No	
NM_hsa_4630	chr8:120868214-120868338	+	CCCACGCCGGACCGCGACTGCAGGA	No	No	Yes	0	0	0	No	
NM_hsa_4633	chr8:99405857-99405993	-	CTTATCCTCCAGTAGACTAGG	No	No	Yes	0	0	0	Yes	
NM_hsa_4634	chr8:146052957-146053065	+	GTCCTCAGGGAGGGTGAGTCGG	No	No	Yes	0	0	0	No	
NM_hsa_4635	chr8:146027629-146027725	+	GTGCAACCTTGGCCATCCTGG	No	No	Yes	0	0.0662225	0	No	
NM_hsa_4636	chr8:100305161-100305303	+	GAGAAGTTTCTCTGAACGTGTAAA	No	No	Yes	0.75981	1.10923	0.62904	Yes	
NM_hsa_4639	chr8:95961727-95961843	+	CCGGGAGCTGAGCGTGAAGGC	No	No	Yes	0	0	0	No	
NM_hsa_4642	chr8:145164591-145164681	+	GCTGCTGGAGCCGGAGGACTGCG	No	No	Yes	0	0	0	No	
NM_hsa_4644	chr9:79186665-79186763	+	CCCGCCGTGCGCCGGGACCGGGGTCCG	No	No	Yes	79.3526	57.15	14.071	Yes	KapB+cMyc>KapB>H
NM_hsa_4645	chr9:25677679-25677759	+	GAGTCGGGTTCCTGTAGAGGCTG	No	No	Yes	0.113971	0	0	No	
NM_hsa_4650	chr9:34049953-34050083	+	TTGTCACCCCATAGATCGCCAGG	No	No	Yes	4.61584	6.15869	0.439717	Yes	
NM_hsa_4652	chr9:132145419-132145525	+	TGGCCGTTGGCCTCTCAGGACTGTCA	No	No	Yes	0	0	0	No	
NM_hsa_4654	chr9:96421752-96421848	+	CTGGGTCCGGTTGTCCTGCAG	No	No	Yes	0	0	0	No	
NM_hsa_4657	chr9:137343002-137343082	+	CATGTGCTGGAATTGCTGGATGT	No	No	Yes	0	0	0	No	
NM_hsa_4661	chr9:30574375-30574507	-	CAGCTGGCGCTCTCGCAGT	No	No	Yes	0.0569857	0	0.146572	Yes	
NM_hsa_4663	chr9:88969518-88969646	+	CACTCGCTCCAGGTGTCGGGCGCCGGG	No	No	Yes	0	0	0	No	
NM_hsa_4666	chr9:139760865-139760961	+	GACGTCGGGGCCTGGCCTGGC	No	No	Yes	0	0	0.0732862	Yes	
NM_hsa_4667	chr9:79186824-79186942	+	ACCATTCGTAGACGACCTGCTTCTGGG	No	No	Yes	79.78	64.4345	57.3098	Yes	KapB+cMyc>KapB>H
NM_hsa_4669	chr9:37804198-37804314	-	TTTCTTTAGGAACTCTGGCTAACTAT	No	No	Yes	0.0427393	0.0331113	0.109929	Yes	
NM_hsa_4670	chr9:88969481-88969619	+	AGGTGTCGGGCGCCGGGTCTGG	No	No	Yes	0	0	0	No	
NM_hsa_4671	chr9:139565924-139566000	+	GCTGGACACTGACCAGGACCCCCCAG	No	No	Yes	546.094	497.728	494.755	Yes	KapB+cMyc>KapB>H
NM_hsa_4672	chr9:37804197-37804309	+	TCTTCAGGAACTCTGGCTAACTAT	No	No	Yes	0.0712322	0.0331113	0.146572	Yes	
NM_hsa_4673	chr9:138628407-138628515	-	GCGGCGGGCGGCGGGCAGCGGG	No	No	Yes	0	0.0110371	0	No	
NM_hsa_4675	chr1:38260002-38260106	-	CGGTGCGGGGCGGCGGCGGCGGCGG	No	No	Yes	1.79582	2.68129	1.7115	Yes	
NM_hsa_4676	chr1:159111368-159111508	-	AGGTTCCGGGTTCGAGTCCCGGC	No	No	Yes	0	0	0	Yes	
NM_hsa_4677	chr1:182643818-182643954	+	CCCAGGAATGTAGAGCTCTGGGTTCT	No	No	Yes	0	0	0	No	
NM_hsa_4678	chr1:9371329-9371439	+	CTGATGGGTCTCGGCGTGGCTGA	No	No	Yes	0.113971	0.0662225	0.0366431	No	KapB+cMyc>KapB>H
NM_hsa_4679	chr1:1510343-1510431	+	GCGAAACGCCGCGCTTGCCGGA	No	No	Yes	0.170957	0	0	No	
NM_hsa_4681	chr1:108962649-108962795	-	GGAGTTAGGTCTGAAGACTGTAAAGC	No	No	Yes	0	0	0	Yes	
NM_hsa_4681	chr1:108816829-108816975	+	GGAGTTAGGTCTGAAGACTGTAAAGC	No	No	Yes	0	0	0	Yes	
NM_hsa_4682	chr1:36107183-36107307	+	TCTCCTCCGGAGCAGGCTGTGCAGG	No	No	Yes	0	0	0	No	
NM_hsa_4685	chr1:154232241-154232373	+	ATAAATTGCACAACTCTCACATTC	No	No	Yes	0	0	0	Yes	
NM_hsa_4687	chr1:113424652-113424774	+	TTCAGGTCTGGGGCTGAAACCTGT	No	No	Yes	0.227943	0.26489	0.219859	No	
NM_hsa_4689	chr1:180910059-180910189	+	CACCCTCCCGGCCCTCCCCCGCC	No	No	Yes	3.5901	2.25157	0.659576	Yes	KapB+cMyc>KapB>H
NM_hsa_4690	chr1:95582796-95582908	+	TCGGCGGCCCGCCTCGGGGCCCCG	No	No	Yes	7.00924	7.41692	1.46572	Yes	
NM_hsa_4692	chr1:32633007-32633135	+	TACAGTGTCACTAGAATCTGGA	No	No	Yes	0	0	0	No	
NM_hsa_4693	chr1:45244024-45244170	+	GTGAAGATAAAGTGTGTCTGAGGAGA	No	No	Yes	0.0854786	0	0	Yes	
NM_hsa_4694	chr1:26881010-26881102	+	TCCTCCTGCCCTCCTTGCTGTAGA	No	No	Yes	0.313421	0.364224	0.0366431	Yes	
NM_hsa_4695	chr1:207226586-207226686	+	GCGACTGTAGCGCCGGTCCCGGC	No	No	Yes	0	0	0	No	
NM_hsa_4696	chr1:569763-569853	-	GTCATTAGGAGGGCTGAGAGGGC	No	No	Yes	1.42464	2.18534	0.879435	Yes	
NM_hsa_4702	chr1:32633019-32633147	+	TCTATTCCAGATTCCAGTGACA	No	No	Yes	0	0	0	No	
NM_hsa_4704	chr1:2929171-2929309	-	CCGCGGGAGCGCCGCGTGGGGC	No	No	Yes	0	0	0	Yes	
NM_hsa_4706	chr1:45244027-45244171	+	TCTCAGTGATGAAAACTTTGTCCAGTTCTG	No	No	Yes	5.12872	2.78135	5.71633	Yes	
NM_hsa_4707	chr1:28202642-28202750	-	CCTGATTTTGTAGCTGTACACT	No	No	Yes	0	0	0	No	
NM_hsa_4707	chr1:28202642-28202740	+	CCTGATTTTGTAGCTGTACACT	No	No	Yes	0	0	0	No	
NM_hsa_4708	chr1:234748680-234748806	+	AGATGCTGGGCCAAGTGTCGG	No	No	Yes	0	0	0	No	
NM_hsa_4713	chr1:149900241-149900381	+	TAGAACACTCTGGCCCCATCTGC	No	No	Yes	0.341914	0.0662225	0	Yes	KapB+cMyc>KapB>H
NM_hsa_4714	chr1:226026269-226026379	+	CTGACTGTGCTCTGTCCCCCCAG	No	No	Yes	0	0	0	Yes	
NM_hsa_4716	chr1:109643120-109643274	+	CCACTGATTATCGAGGCGATTCTGATCTGG	No	No	Yes	0.740814	0.0662225	0.146572	Yes	
NM_hsa_4717	chr1:17919127-17919269	+	TCCCAGATTGCCGCAGACCTAGTC	No	No	Yes	2.33641	1.05956	2.49173	No	
NM_hsa_4718	chr1:113162463-113162573	+	GTGTCGGATGAGGAGAAGGTAAG	No	No	Yes	0	0	0	No	
NM_hsa_4720	chr1:149822290-149822380	-	GTCGGTGTCGGTCTTGGGTCTGG	No	No	Yes	0	0	0	No	
NM_hsa_4720	chr1:149814566-149814656	+	GTCGGTGTCGGTCTTGGGTCTGG	No	No	Yes	0	0	0	No	
NM_hsa_4721	chr1:45243499-45243599	+	TTCTCGTGATGAAAACTCTGTCCAGTTC	No	No	Yes	23.82	30.9259	34.6644	Yes	H>KapB>KapB+cMyc
NM_hsa_4723	chr1:43893827-43893935	+	TGGCCCCTTCCACTTCCATCAG	No	No	Yes	0	0	0	Yes	
NM_hsa_4725	chr1:40254174-40254324	+	TCGGCGGGCGGCGGGCGGCGGGGCGGGG	No	No	Yes	2.53017	1.92266	1.51458	Yes	KapB+cMyc>KapB>H
NM_hsa_4726	chr1:145726393-145726515	+	TGGGATTGCGATGAACTCAGGTGT	No	No	Yes	0	0	0	Yes	
NM_hsa_4729	chr1:203698757-203698843	+	ATTCCAGGGTGCTGTGGCTGC	No	No	Yes	0	0	0.146572	No	
NM_hsa_4731	chr1:203259533-203259673	-	AGCTGGAGCCAAGGACTGAGCGC	No	No	Yes	0	0	0	No	
NM_hsa_4732	chr1:164528359-164528493	+	AGGCTGACGGGGCTGCTGGA	No	No	Yes	0	0	0	No	
NM_hsa_4733	chr1:158057677-158057825	+	ACTGGGGAGGCCAGGTGTCATGGATGT	No	No	Yes	0	0	0	No	
NM_hsa_4734	chr1:178995468-178995558	+	TGCGGTCGCGGCTGAAGGGACGC	No	No	Yes	0	0	0	No	
NM_hsa_4735	chr1:231400529-231400645	+	TCATGTTGCTCTGCTGTTGCC	No	No	Yes	0.683829	1.05956	0.0732862	Yes	
NM_hsa_4736	chr1:156857386-156857520	+	TCCCCAGTACCCCCACCACA	No	No	Yes	124.001	94.5657	80.0286	Yes	KapB+cMyc>KapB>H
NM_hsa_4738	chr1:232423523-232423623	+	TCCAGAGACTGGAGCTGACTTGT	No	No	Yes	0	0	0	No	
NM_hsa_4740	chr1:149900246-149900386	+	TGGATGGGATCGGAGCTCTAGAG	No	No	Yes	0.0569857	0	0	Yes	
NM_hsa_4741	chr1:78444935-78445075	+	TGATAGTGGCCAGTTGACTGCGA	No	No	Yes	0	0	0	No	
NM_hsa_4742	chr1:156186555-156186681	+	GGCCGGCCCGCCTCCGCTCCC	No	No	Yes	0	0	0	No	
NM_hsa_4747	chr1:8324223-8324343	+	TCTGTGTTTTGGACATCTCCACT	No	No	Yes	0	0	0	No	
NM_hsa_4749	chr1:144839419-144839519	-	TCAGAGTTCAAGTCTCACTGGGA	No	No	Yes	0	0.0331113	0.146572	Yes	H>KapB>KapB+cMyc
NM_hsa_4749	chr1:145379303-145379403	-	TCAGAGTTCAAGTCTCACTGGGA	No	No	Yes	0	0.0331113	0.146572	Yes	H>KapB>KapB+cMyc
NM_hsa_4750	chr1:244614485-244614625	+	GCAGGCCTCGGGCGGTAGCTCCT	No	No	Yes	0	0	0	No	
NM_hsa_4751	chr1:1342745-1342835	+	CTCCTGAACTCGCGGTGGTGGTG	No	No	Yes	0	0	0	No	
NM_hsa_4754	chr1:151431194-151431274	+	GTGCGTGTGGACGTCGGGCTGTG	No	No	Yes	0	0	0	No	
NM_hsa_4755	chr1:24668531-24668619	+	CAGCGGCCCCCTCTGTCTCCAG	No	No	Yes	0	0	0	No	
NM_hsa_4757	chr1:202858015-202858143	+	GCTCGTCGGGGGAGACTGTGGG	No	No	Yes	0	0	0	No	
NM_hsa_4758	chr1:204965420-204965550	+	TTAGCTCCCTCTTCCCATGTTCT	No	No	Yes	0	0	0	Yes	
NM_hsa_4759	chr1:154781480-154781570	+	TTACTGATCTCTGGTTTGTGGCT	No	No	Yes	0	0	0	No	
NM_hsa_4761	chr1:28202633-28202763	+	TTTGTAGCTGTACACTAAGGGAA	No	No	Yes	0	0	0	No	
NM_hsa_4762	chr1:1290479-1290617	+	GCGCGCGGGCCGGGGCGGCGGT	No	No	Yes	4.01749	2.28468	2.34516	Yes	
NM_hsa_4763	chr1:23695464-23695540	+	GAGTGCGGTCGGCAGTAGAGCCTGGC	No	No	Yes	0	0	0.219859	No	
NM_hsa_4766	chr1:32633033-32633141	+	TTCCAGATTCCAGTGACACTAT	No	No	Yes	0	0	0	No	
NM_hsa_4770	chr1:171070852-171070966	+	TTTCACCCAGATCTGCGGCCTAATC	No	No	Yes	0	0	0	No	
NM_hsa_4772	chr1:45244024-45244168	+	AGTGAAGATAAAGTGTGTCTGAGGA	No	No	Yes	0.0284929	0	0	Yes	
NM_hsa_4773	chr1:55784349-55784427	+	AGCCTCCCAGTCTGGCCTGAGT	No	No	Yes	0.113971	0	0	No	
NM_hsa_4774	chr1:156186242-156186330	+	CTCTGGCCTACCGGTGACCCGG	No	No	Yes	0	0	0.0732862	Yes	
NM_hsa_4778	chr2:174887537-174887649	-	TGCTTTAGGTAGGAGGCTGTTGAA	No	No	Yes	0	0	0	Yes	
NM_hsa_4778	chr2:174887539-174887661	+	TGCTTTAGGTAGGAGGCTGTTGAA	No	No	Yes	0	0	0	Yes	
NM_hsa_4780	chr2:217363502-217363644	+	ACCTAGGTCGCGGCGACATGGTGA	No	No	Yes	0.113971	0	0	Yes	
NM_hsa_4781	chr2:220147998-220148138	+	AACTGGGACCAGCGCCTGCAGGA	No	No	Yes	0	0	0	Yes	
NM_hsa_4782	chr2:5832505-5832623	+	CTGGGAGAGCTCGGGGTCCGGC	No	No	Yes	0	0	0	No	
NM_hsa_4783	chr2:43454421-43454545	+	CTGAGTGTCTGAGGACAAGG	No	No	Yes	0	0	0	No	
NM_hsa_4784	chr2:133012590-133012682	-	TTATTCCCATGACCCGCTGGGCAGCTTCC	No	No	Yes	40.6308	31.7206	54.5983	Yes	
NM_hsa_4785	chr2:69402650-69402766	+	AAGCCCAGAAGCTGGAAACTGCAGTG	No	No	Yes	0	0	0	Yes	
NM_hsa_4787	chr2:178178515-178178625	+	CTACCATTAGAAGGGCTGGAAGA	No	No	Yes	0	0	0	No	
NM_hsa_4788	chr2:206950678-206950778	+	TGGCAATGGACTAGGAAGCTCGG	No	No	Yes	0.113971	0	0	No	
NM_hsa_4789	chr2:239404093-239404203	+	ACTCGGACTGAGCGCTTGGAAGA	No	No	Yes	0	0.132445	0	No	
NM_hsa_4790	chr2:234184470-234184608	+	TGGGATTCTGTCAGTGTGTGTT	No	No	Yes	0	0	0	Yes	
NM_hsa_4791	chr16:33964220-33964318	+	CGCTGAGAAGACAGTCGAACTTGACTA	No	No	Yes	270.454	238.732	87.9435	Yes	KapB+cMyc>KapB>H
NM_hsa_4791	chr2:133011950-133012078	-	CGCTGAGAAGACAGTCGAACTTGACTA	No	No	Yes	270.454	238.732	87.9435	Yes	KapB+cMyc>KapB>H
NM_hsa_4792	chr2:98280700-98280838	+	GCGCGCGGCCGGAGTCTGTGGA	No	No	Yes	0	0	0	No	
NM_hsa_4794	chr2:149402333-149402435	+	AGCGGATCGGGCGGGCGAGCGGCG	No	No	Yes	0.683829	0.463558	0.183216	No	KapB+cMyc>KapB>H
NM_hsa_4795	chr2:223184096-223184244	+	CCTCGGCAGGCGGCGGCGGCGGCGGCT	No	No	Yes	0.319729	0.243672	0.110717	Yes	KapB+cMyc>KapB>H
NM_hsa_4797	chr2:66660772-66660860	+	CCACTGGTAGAGCTGCTGCGGCTGAGC	No	No	Yes	0	0.0662225	0	No	
NM_hsa_4798	chr2:21728866-21729002	+	TTGAAAAGTTCACGATTGAGC	No	No	Yes	0	0	0	Yes	
NM_hsa_4800	chr2:111892952-111893032	+	TTGTGTCCAGTTGTTGGGGGAGA	No	No	Yes	0.0569857	0	0	Yes	
NM_hsa_4801	chr2:232329472-232329558	+	GTCTCCGGTTCGCCAATGTGG	No	No	Yes	0	0	0	No	
NM_hsa_4803	chr2:230045621-230045749	-	GGGGCCCGTGCCTTGGAAAGCGTCGCT	No	No	Yes	6.83829	10.2645	24.1112	Yes	H>KapB>KapB+cMyc
NM_hsa_4805	chr2:28563432-28563552	+	ACTGAGCCCGGACTTGCTTCTGA	No	No	Yes	0	0	0	No	
NM_hsa_4806	chr2:96192951-96193091	+	ACGGGGTTTGAGGGCTGGGTGAG	No	No	Yes	0	0	0	No	
NM_hsa_4807	chr2:233562016-233562150	+	CCCGGACCGCAGGTAACCAG	No	No	Yes	0	0	0.146572	No	
NM_hsa_4809	chr2:108710009-108710145	-	GAGGTAGAGCACTAAATGGGC	No	No	Yes	0	0	0	Yes	
NM_hsa_4810	chr2:232329407-232329525	+	GTTTTTGGCGCCGGCTGTGAGG	No	No	Yes	0.0569857	0	0.806149	No	
NM_hsa_4811	chr2:225235712-225235790	+	GAAGCCAGGCTGTTAGGCTAGT	No	No	Yes	0	0	0	No	
NM_hsa_4812	chr2:114514328-114514426	+	CTCTGGTCGCCGAGCGAGGGCT	No	No	Yes	0	0.26489	0.0732862	No	
NM_hsa_4813	chr2:121010899-121010999	+	TTGGGCAGGGTCAGGGCTGGGAA	No	No	Yes	0.0569857	0.0662225	0.0732862	No	H>KapB>KapB+cMyc
NM_hsa_4814	chr2:133038221-133038355	-	CTGGTGAGCTCTGGCTGGCC	No	No	Yes	1.13971	0.79467	1.31915	Yes	
NM_hsa_4817	chr2:130635315-130635453	+	GCGCGCGTGTCGGGGGGCGGGC	No	No	Yes	1.13971	0.397335	0.0732862	Yes	KapB+cMyc>KapB>H
NM_hsa_4818	chr2:36596650-36596768	-	AAGTTCTGTAGAAATGTTTGCA	No	No	Yes	0	0	0	No	
NM_hsa_4820	chr2:234197353-234197475	+	CCCAGCTGCCTCTCCTCCATCGCA	No	No	Yes	0.0569857	0	0	Yes	
NM_hsa_4822	chr2:160595422-160595548	+	TCCAGACGGGGGCCAAGGTAG	No	No	Yes	0	0	0	Yes	
NM_hsa_4824	chr2:9144152-9144282	+	CGGCTGGCCAGCGTCGTGGGGCG	No	No	Yes	0	0	0	No	
NM_hsa_4825	chr2:157257280-157257398	+	TCTCCAAATCAGCCGACGTTTG	No	No	Yes	0.7978	1.52312	1.02601	No	
NM_hsa_4826	chr2:218905686-218905786	-	CCTTTCAGATGGTTCTCTAGAGA	No	No	Yes	0	0	0	No	
NM_hsa_4829	chr2:103141865-103141983	+	TTATGGTGACCAGGTACTGACC	No	No	Yes	0	0	0	No	
NM_hsa_4831	chr2:86363051-86363145	+	GACTGTTGCCTCAATCTGAGCCTGC	No	No	Yes	0	0	0	Yes	
NM_hsa_4833	chr2:242741102-242741230	+	CTGCGTGTGCCTTCCCTCCCAG	No	No	Yes	0	0	0	No	
NM_hsa_4834	chr2:161350435-161350547	+	CTCCCAGGCTCGCGGTTGGACGGG	No	No	Yes	0	0	0	No	
NM_hsa_4836	chr2:157190922-157191028	+	TTTCCCCCAGATGTTGCGGCTGCCGC	No	No	Yes	0.455886	0.0662225	0.0732862	Yes	
NM_hsa_4837	chr2:218905695-218905783	-	GTCCTTTCAGATGGTTCTCTAG	No	No	Yes	0	0	0	No	
NM_hsa_4838	chr2:133011965-133012089	-	TTTAGTGAGGCCCTCGGATCAGCCC	No	No	Yes	10.1435	7.3507	5.64304	Yes	KapB+cMyc>KapB>H
NM_hsa_4839	chr2:217363522-217363630	+	TAGGTCGCGGCGACATGGTGAG	No	No	Yes	0	0	0	No	
NM_hsa_4840	chr2:232379269-232379367	+	CGCGGTGTCCCAGGTCTTCGCG	No	No	Yes	0	0	0	No	
NM_hsa_4844	chrX:153996830-153996974	+	TTCATTTGCCTCCCAGCCTACAAAA	No	No	Yes	0.0569857	0.0993338	0.0366431	Yes	
NM_hsa_4844	chr2:69747135-69747279	-	TTCATTTGCCTCCCAGCCTACAAAA	No	No	Yes	0.0569857	0.0993338	0.0366431	Yes	
NM_hsa_4846	chr3:105289156-105289272	+	CAAATTGACATTTGGGAACTG	No	No	Yes	0	0	0	Yes	
NM_hsa_4849	chr3:49726983-49727085	+	GAGTGAGGGGCGGCAGGGCTCTGA	No	No	Yes	0	0.0662225	0	No	
NM_hsa_4851	chr3:44552207-44552353	+	GGCGGCGGGCGGCGGGCGGCGGGGCT	No	No	Yes	2.63464	2.28688	1.88712	Yes	KapB+cMyc>KapB>H
NM_hsa_4852	chr3:128735283-128735417	+	AGGCAGGTGGAGCTCATGGGCACGC	No	No	Yes	0.455886	0.0331113	0.0732862	No	
NM_hsa_4853	chr3:129116031-129116147	-	TGGTGATTGGAAGACACTCTGCGACA	No	No	Yes	0.0854786	0.0993338	0.146572	Yes	H>KapB>KapB+cMyc
NM_hsa_4854	chr3:178866109-178866207	+	GCCGCTGAGGTGTCGGGCTGCTGCTGC	No	No	Yes	0.0569857	0	0.146572	Yes	
NM_hsa_4855	chr3:160118950-160119060	-	TTGAATTTTTGTTTGTGACCTGT	No	No	Yes	0	0.0496669	0.0732862	No	H>KapB>KapB+cMyc
NM_hsa_4859	chr3:48468648-48468756	+	CACTGAGGAGGTCGGCCGCTGA	No	No	Yes	0	0	0	No	
NM_hsa_4860	chr3:51978936-51979048	+	CTCAGGGTGGCAGGGCTGTGGGGC	No	No	Yes	0	0.132445	0	Yes	
NM_hsa_4861	chr3:38367116-38367220	+	CTGCATCTCAGGTCTGCACCTCTGG	No	No	Yes	0	0	0	No	
NM_hsa_4862	chr3:193921967-193922067	+	CTCCGGCGCGGGCGTGTCTCTGC	No	No	Yes	0	0	0	No	
NM_hsa_4863	chr3:194406536-194406672	+	AAGGATGTGAGGCAGGCGAGC	No	No	Yes	0.113971	0.596003	0.293145	No	
NM_hsa_4864	chr3:48357840-48357962	+	TCTAGCCTCCATGAATTCTGATGA	No	No	Yes	0.940265	0.827781	0.732862	Yes	KapB+cMyc>KapB>H

Additional file 5

Name	locus	strand	sequence	mirdeep2	mireap	miranalyzer	RPM_HU_KapB_cMyc	RPM_HU_KaposinB	RPM_HUVEC	IP_confidence	annotation
NM_hsa_4865	chr3:194622959-194623097	-	AAGGTGTTAGACTGTAAGCATT	No	No	Yes	0	0	0	No	
NM_hsa_4865	chr3:194622983-194623111	+	AAGGTGTTAGACTGTAAGCATT	No	No	Yes	0	0	0	No	
NM_hsa_4868	chr3:120068354-120068460	+	CTGAGCCGGACCGGGTTTCGG	No	No	Yes	0	0	0	No	
NM_hsa_4870	chr3:156445497-156445627	-	ACTGACTGTCTCTGAAGAAATCA	No	No	Yes	0	0	0	No	
NM_hsa_4870	chr3:156445498-156445618	+	ACTGACTGTCTCTGAAGAAATCA	No	No	Yes	0	0	0	No	
NM_hsa_4874	chr3:175325965-175326085	-	CAGATTGTGGACTATGGGCTAAT	No	No	Yes	0	0	0	No	
NM_hsa_4874	chr3:175325984-175326084	+	CAGATTGTGGACTATGGGCTAAT	No	No	Yes	0	0	0	No	
NM_hsa_4878	chr3:129116035-129116139	-	GTGATTGGAAGACACTCTGCGACAG	No	No	Yes	0.0854786	0.0331113	0.0732862	Yes	
NM_hsa_4879	chr3:184043407-184043545	+	TTCCTCTGATGACTTCCTGTTAGTGCC	No	No	Yes	3.70407	3.44357	4.17732	Yes	
NM_hsa_4880	chr3:184454951-184455093	+	TTCCAGGTCAAAGGCTGCAGGAGT	No	No	Yes	0	0	0	No	
NM_hsa_4881	chr3:195270226-195270346	+	CTCTGAGAACCTGCGGGAGAGCG	No	No	Yes	0	0	0	No	
NM_hsa_4882	chr3:39452510-39452650	+	TGTGCACATTGTTAGAGCTTGGAGTTGA	No	No	Yes	4.84379	6.15869	3.00474	Yes	
NM_hsa_4884	chr3:182787676-182787778	+	AGTGGCTTAGATATGAACTCTGGA	No	No	Yes	0.113971	0.0662225	0	Yes	KapB+cMyc>KapB>H
NM_hsa_4887	chr3:48647162-48647298	+	TGGCTGACTGGCTCTGACCGCGCTGC	No	No	Yes	0	0	0	No	
NM_hsa_4888	chr3:195459934-195460054	+	CTGGCGAGCTCATGGGCCAGATG	No	No	Yes	0	0	0	No	
NM_hsa_4890	chr3:160118951-160119079	+	TGTGAATTTTTGTTTGTGACCTGTGGA	No	No	Yes	0.113971	0.380779	0.109929	Yes	
NM_hsa_4895	chr3:43220981-43221091	+	TTTCCCAAGTGTCTTGGACCAGG	No	No	Yes	0	0	0	No	
NM_hsa_4896	chr3:164059128-164059230	+	GTTAATATCTCGCTGGGGCCTCCA	No	No	Yes	427.051	325.715	306.117	Yes	KapB+cMyc>KapB>H
NM_hsa_4897	chr3:137844467-137844559	+	CCTAGAACTCTCTTCTCTTGCAGT	No	No	Yes	0	0	0	No	
NM_hsa_4898	chr4:53578762-53578870	+	GTCCCCACGAGCTTTGCCCGCC	No	No	Yes	0	0	0	No	
NM_hsa_4899	chr4:25235502-25235654	-	AGAAGGGTCGTGGCGGCGGCGGCGGCGGC	No	No	Yes	2.74637	1.80384	2.03518	Yes	
NM_hsa_4900	chr4:25661099-25661209	-	TGTCATGAACAATAAACAAGAGT	No	No	Yes	0	0	0	No	
NM_hsa_4902	chr4:125634189-125634297	+	AGGGACTCGCCGGCTGTTGGTG	No	No	Yes	0.0569857	0	0	No	
NM_hsa_4903	chr4:129576484-129576628	+	TCTTCTTTATTCCAGATCCAGGATA	No	No	Yes	0	0	0	No	
NM_hsa_4904	chr4:122618259-122618395	+	CCCGCGGCGCGGACTGTCCCG	No	No	Yes	0.227943	0	0.0732862	No	
NM_hsa_4905	chr4:119200313-119200443	+	ATCTCCATGTATCTTTGGGACCTGTCAG	No	No	Yes	6.32542	4.96669	5.27661	Yes	
NM_hsa_4911	chr4:7087084-7087210	+	AACTGGGCATGTTGGAACTAAGCTGA	No	No	Yes	0.322919	0	0.097715	Yes	
NM_hsa_4913	chr4:1006300-1006418	-	TCGGGCGGCGGCGGCCGGCGGG	No	No	Yes	1.11122	0.695336	0.366431	Yes	KapB+cMyc>KapB>H
NM_hsa_4916	chr4:4249955-4250041	+	CTGGCGTTTGGATTGGAGACTGTGTT	No	No	Yes	0	0	0.219859	No	
NM_hsa_4918	chr4:25661121-25661193	+	TGTCATGAACAATAAACAAGAGTT	No	No	Yes	0	0	0	No	
NM_hsa_4919	chr4:129471268-129471410	+	TCCTACGTGACCAGAAGTTTCAGA	No	No	Yes	0	0	0	Yes	
NM_hsa_4920	chr4:3230266-3230358	+	CACCCACCAGGAGCCTGGCACTGT	No	No	Yes	0.284929	0	0.0732862	Yes	
NM_hsa_4922	chr4:141073423-141073523	+	TTCCCCGCTTCCCCCCTAGGGGT	No	No	Yes	0	0.0662225	0	Yes	
NM_hsa_4923	chr4:86594782-86594912	+	ATGCTTAGATCTCCCCACTGGTA	No	No	Yes	0	0	0	No	
NM_hsa_4924	chr4:5209394-5209480	+	AAGAAAGTGTCTGAGACTGCAGCTGA	No	No	Yes	0	0	0	No	
NM_hsa_4925	chr4:53578771-53578869	+	GCGGCCAGAACGGAGGGGTAGAGCAGC	No	No	Yes	0	0	0	No	
NM_hsa_4930	chr4:170947699-170947831	+	CACCGGAGGCTGTAGTTGC	No	No	Yes	0	0	0	No	
NM_hsa_4932	chr4:68140-68280	+	CCCTCAAAAGACAGGAATGTGGA	No	No	Yes	0	0	0	Yes	
NM_hsa_4933	chr4:20253489-20253597	+	GCGCCCGGACGGCGGAGCTTGG	No	No	Yes	0	0	0	No	
NM_hsa_4934	chr10:107803635-107803779	+	TAGAGAACCATGGTCATAGACTAGT	No	No	Yes	0	0	0	No	
NM_hsa_4935	chr10:103870877-103870983	-	TCTCTCCTTCACTTCCTCCAG	No	No	Yes	0	0	0	Yes	
NM_hsa_4936	chr10:73550472-73550592	+	ACTTTCCTGTCCTCCAACCAGAC	No	No	Yes	0	0	0	Yes	
NM_hsa_4937	chr10:107803655-107803775	-	AGAAGCACTAGTCTATGACCATG	No	No	Yes	0	0	0	No	
NM_hsa_4938	chr10:38383735-38383845	+	CAGGCGCGGACTCCACACCCAGC	No	No	Yes	0.19945	0.26489	0.293145	Yes	H>KapB>KapB+cMyc
NM_hsa_4939	chr10:123734372-123734508	+	CGGTACTGATGGCGGATCTGG	No	No	Yes	0	0	0	No	
NM_hsa_4941	chr10:107803635-107803763	-	AGAACCATGGTCATAGACTAGT	No	No	Yes	0	0	0	No	
NM_hsa_4944	chr10:60447670-60447798	+	AACCTCCCCCGAGTGTTACAGC	No	No	Yes	0.284929	0.26489	1.02601	Yes	
NM_hsa_4945	chr10:100977961-100978071	-	TTACCTGACATTCTGAGTTTCCT	No	No	Yes	0	0	0	Yes	
NM_hsa_4946	chr10:94624475-94624605	+	TCAGGTCTTCAGACTGTGGGGCT	No	No	Yes	6.72432	6.82092	8.86764	Yes	H>KapB>KapB+cMyc
NM_hsa_4947	chr10:123790151-123790271	+	TCTGGGTCATGTCTGGCTGAGGT	No	No	Yes	0	0	0	No	
NM_hsa_4948	chr10:100977973-100978063	+	TTACCTGACACTTTGAGTTTCCT	No	No	Yes	0	0	0	Yes	
NM_hsa_4949	chr10:23003584-23003700	+	CTGCGCCTTCCCGCCCCGGCT	No	No	Yes	0	0	0.0732862	Yes	
NM_hsa_4951	chr10:5003115-5003245	+	TTAAGTGGCCTGAATTTGTAGAT	No	No	Yes	0	0	0	No	
NM_hsa_4954	chr10:3827699-3827797	+	CTGTGAGGTGCGCGGCCGAGCC	No	No	Yes	0.0569857	0	0	No	
NM_hsa_4957	chr10:82905991-82906127	+	CGAGATCTCCTGTGAACTCAG	No	No	Yes	0	0	0	No	
NM_hsa_4959	chr10:81965366-81965460	+	GCTGCTCCAGGAAGTCTCGGGCTCC	No	No	Yes	0	0	0	No	
NM_hsa_4960	chr10:79829488-79829586	+	CTGAAGGCTCTGAGCCGGGAGA	No	No	Yes	0	0	0	Yes	
NM_hsa_4961	chr10:43966644-43966784	+	CGTGTCCAGAATGGCCAGCCAGA	No	No	Yes	0	0	0	No	
NM_hsa_4962	chr10:43966664-43966772	+	TCCAGAATGGCCAGCCAGATCC	No	No	Yes	0	0	0	No	
NM_hsa_4963	chr10:24497805-24497907	-	CCGTCCGTCCGTCCGTCCGTCCTC	No	No	Yes	14.1799	8.15641	3.49331	Yes	KapB+cMyc>KapB>H
NM_hsa_4964	chr10:85899289-85899427	+	CGAAGGTGACCGGGGACCGAGG	No	No	Yes	0	0	0.293145	No	
NM_hsa_4965	chr10:1095176-1095268	+	TCAGACGTCTCGAGGCTCGCGTTC	No	No	Yes	0.3989	0	0.0732862	Yes	
NM_hsa_4967	chr10:129535410-129535542	+	TCGCCAGTGAGTTTCGGTGCGGCA	No	No	Yes	0	0	0	No	
NM_hsa_4968	chr10:120514847-120514949	+	GTGGACTGGCCAGAGTGATTGGAG	No	No	Yes	0.113971	0	0.219859	No	
NM_hsa_4970	chr10:35625374-35625492	+	TCCCTGGCCGCGCCGCACCGCG	No	No	Yes	0	0	0	Yes	
NM_hsa_4974	chr11:118663749-118663891	+	AATCAAGTGATGGGTCGCGGCTGA	No	No	Yes	0	0	0	No	
NM_hsa_4975	chr11:130442110-130442238	+	CTTGCACTGGACTGGGAATGAG	No	No	Yes	0	0	0	No	
NM_hsa_4976	chr11:123480409-123480509	+	TTGGAGGGGAAACTTGGATTGGG	No	No	Yes	0	0	0	Yes	
NM_hsa_4977	chr11:118798938-118799066	+	GGCGTTCGTCGGTGGAAGTGCC	No	No	Yes	0	0	0	No	
NM_hsa_4980	chr11:130442097-130442239	-	TTTTGCACTGGACTGGGAATGAGT	No	No	Yes	0	0	0.0732862	No	
NM_hsa_4981	chr11:118798933-118799069	+	TGGCGTTCGTCGGTGGAAGTG	No	No	Yes	0	0	0	No	
NM_hsa_4982	chr11:133012694-133012834	+	CTGGGCAAAGAACTCCAAGTGCC	No	No	Yes	0	0	0	No	
NM_hsa_4983	chr11:101982001-101982105	-	TTCCACTTCGCCTCGGGACC	No	No	Yes	0	0	0	No	
NM_hsa_4986	chr11:2950463-2950583	+	GGAAGAGGCTGTCGCTGCGCTTC	No	No	Yes	2.27943	2.45023	0.806149	No	
NM_hsa_4989	chr11:131565769-131565905	+	GCCGCTCAAAGGACAGGGCTG	No	No	Yes	0	0	0	No	
NM_hsa_4991	chr11:67166298-67166412	-	CTGCTCCCATCCTCGCCCAG	No	No	Yes	0	0	0	No	
NM_hsa_4992	chr11:8705815-8705929	+	TCAGTCACCAGGTTAGAGACATGCT	No	No	Yes	1.76656	3.50979	1.24587	Yes	
NM_hsa_4993	chr11:75946899-75947005	-	AGATGGCTCGTTGGTCTAGGGGTATG	No	No	Yes	1.9945	2.53853	4.11624	Yes	H>KapB>KapB+cMyc
NM_hsa_4995	chr11:85195143-85195261	+	GGAGGATTCAACCCGGTGGCGG	No	No	Yes	9.28867	7.28448	4.17732	Yes	KapB+cMyc>KapB>H
NM_hsa_4996	chr11:116708330-116708468	-	CAGCCCAGACCCTGGCTGCAGA	No	No	Yes	0	0	0	No	
NM_hsa_5000	chr11:65326464-65326594	+	CGGGGCTGGCTCTGGGCCTCAGA	No	No	Yes	0	0	0	No	
NM_hsa_5001	chr11:64785990-64786112	+	CCTGTCACCCGCTCCTTGCCCAGA	No	No	Yes	0	0.198668	0	Yes	
NM_hsa_5004	chr11:8705816-8705926	+	TGCTGACCTTATTCACTGGCTGT	No	No	Yes	0	0	0	No	
NM_hsa_5005	chr11:66031423-66031551	+	CTGGCTCCCTCTCCCATCCCAG	No	No	Yes	0	0	0	No	
NM_hsa_5006	chr11:2422423-2422561	+	CGGAGTGCCCCGCTGACCAGGC	No	No	Yes	0	0	0	No	
NM_hsa_5009	chr11:117872271-117872397	+	AAACAGGGCTGAGGACTCAGG	No	No	Yes	0	0	0	No	
NM_hsa_5012	chr11:100841570-100841698	+	TTCGCTGGGAATTCAGCCTCTG	No	No	Yes	0	0	0	Yes	
NM_hsa_5015	chr11:10830680-10830806	+	GCCCTTTTGAGTCTGCGTTGG	No	No	Yes	0	0	0	No	
NM_hsa_5016	chr11:118491757-118491865	+	ACAGGGGGCTGGGAATGAGACC	No	No	Yes	0.512871	0.132445	0	No	KapB+cMyc>KapB>H
NM_hsa_5018	chr11:5957526-5957652	-	CATGCTGCCGTGGCTACTAGT	No	No	Yes	0	0	0	No	
NM_hsa_5022	chr12:122905534-122905666	-	TATTCTGACTTCAGGTGCTTAGTT	No	No	Yes	0	0.132445	0	Yes	
NM_hsa_5025	chr12:97856504-97856648	+	ATTCTGCACGGACTGCTGGGCAGCA	No	No	Yes	0	0	0	Yes	
NM_hsa_5027	chr12:6965378-6965498	+	CTCTGACCCTCTGCTTCCCCCAG	No	No	Yes	0	0.132445	0.0732862	Yes	
NM_hsa_5030	chr12:56873826-56873958	+	TTCCTGTCCTTTTTCCAGAGGGTT	No	No	Yes	0	0.0662225	0	Yes	
NM_hsa_5031	chr12:7073183-7073305	+	TTCTCTGTCGGCCGGCCCTGGGTC	No	No	Yes	0	0	0	No	
NM_hsa_5032	chr12:98993538-98993680	+	ATGTTAGTCTAACGTTCTGATAGA	No	No	Yes	0	0	0	No	
NM_hsa_5034	chr12:53610576-53610672	+	GCGCGCGCGCGCGCGTGTGGT	No	No	Yes	0.0189952	0	0	Yes	
NM_hsa_5038	chr12:124798647-124798755	+	CTGAGGGCAGGGAATCGGGCTG	No	No	Yes	0	0	0	No	
NM_hsa_5043	chr12:76049398-76049506	+	TTTGCCTTGGTCTTCAGAGATC	No	No	Yes	0	0	0	No	
NM_hsa_5044	chr12:53454377-53454483	+	ATCTGCCCTTCCACCCTACAG	No	No	Yes	0	0	0	Yes	
NM_hsa_5047	chr12:52541321-52541449	+	ACACACTGGAGCTCAGGACACGGCGCC	No	No	Yes	0	0.0662225	0	No	
NM_hsa_5048	chr12:51663543-51663649	+	CTTCTGGCTCGCCTCGGACGC	No	No	Yes	0	0	0.0732862	Yes	
NM_hsa_5049	chr13:55161731-55161871	+	GTTCATAGATTTCGTGTGTGTGT	No	No	Yes	0	0	0	Yes	
NM_hsa_5050	chr13:112276311-112276431	+	TCCACAGGGTCCAGATCCGAGTG	No	No	Yes	0	0	0	No	
NM_hsa_5051	chr13:96705699-96705829	+	CGGACACTGGGCCACGTCGGAGC	No	No	Yes	0	0	0	No	
NM_hsa_5052	chr13:78556249-78556389	+	CAGATTGGTTGAACTGGTTTGCA	No	No	Yes	0	0	0	No	
NM_hsa_5059	chr13:26828218-26828354	+	CTCTCTTTGAGGAGGTACCGG	No	No	Yes	0	0	0	No	
NM_hsa_5060	chr13:55195275-55195385	+	TAGAGGGACCAGAACATACTGTG	No	No	Yes	0	0	0	Yes	
NM_hsa_5061	chr13:110889340-110889470	-	TCTGGGGCCCTGGGCAGACCTCC	No	No	Yes	2.96326	1.58934	2.78488	Yes	
NM_hsa_5065	chrY:10033318-10033406	+	CCTCCCTTCCCCAGGCGTCCCT	No	No	Yes	0	0	0	Yes	
NM_hsa_5069	chrX:48937619-48937715	+	CCCGGGTCTTCGGAGCATCGC	No	No	Yes	0	0	0	No	
NM_hsa_5076	chrX:15510139-15510265	-	TCTGTCAGCCTCATTGGTAGC	No	No	Yes	0.0569857	0.0662225	0.0732862	Yes	H>KapB>KapB+cMyc
NM_hsa_5077	chrX:77041410-77041508	+	CAGACCGCTGGGGCCCATGAGA	No	No	Yes	0	0	0	No	
NM_hsa_5079	chrX:154003303-154003437	+	AAGTGGTGAGTTCTTCTGTCCAGCG	No	No	Yes	0.626843	0.52978	0.293145	Yes	KapB+cMyc>KapB>H
NM_hsa_5080	chrX:153719107-153719233	+	CTGCCTGGACCAATCGCTTGG	No	No	Yes	0	0	0	No	
NM_hsa_5081	chrX:118921300-118921396	-	TCATGACAGATTGACATGGACAATTC	No	No	Yes	1.31067	1.72179	2.05201	Yes	H>KapB>KapB+cMyc
NM_hsa_5082	chrX:17432188-17432296	+	TTTCCAGGAGGACTGGCCCAGA	No	No	Yes	0	0	0	No	
NM_hsa_5083	chrX:36124352-36124472	+	AGATTGCTCTTTGACTAATTAGG	No	No	Yes	0	0	0	No	
NM_hsa_5084	chrX:15510142-15510264	-	TGCTACCAGTGAGGCTGACAGATG	No	No	Yes	0.0854786	0.0331113	0.0732862	Yes	
NM_hsa_5086	chrX:152600915-152601003	+	GGATGTACTGAACTGGGTCAGA	No	No	Yes	0	0	0	No	
NM_hsa_5088	chrX:14485432-14485518	+	AATGCAGCCTGAGTAGTACTA	No	No	Yes	0	0	0	No	
NM_hsa_5089	chrX:153192928-153193056	+	CAGGACCTGGACTTCCAGCAGC	No	No	Yes	0.3989	0.52978	0.293145	Yes	
NM_hsa_5092	chrX:21392409-21392515	+	CTCGTGGCGCGCGCGCCCCGC	No	No	Yes	0	0	0	No	
NM_hsa_5093	chr15:83424755-83424831	+	AAAGGTAGATAGAACAGGTCTTGTTT	No	No	Yes	72.5143	75.5599	71.8205	Yes	
NM_hsa_5095	chr15:81134308-81134416	+	GACAACTATGGATGAGCTCTCA	No	No	Yes	0	0	0	Yes	
NM_hsa_5096	chr15:45493243-45493385	+	CTGCTCGTCGTGGCGTCCCGGTAG	No	No	Yes	0.113971	0.132445	0.439717	No	H>KapB>KapB+cMyc
NM_hsa_5100	chr15:80216315-80216407	+	ATTCCGCGAGCCGGGCTGAGTTGG	No	No	Yes	0	0	0.0732862	No	
NM_hsa_5104	chr15:80772108-80772214	+	CCAGGGAGACTCTGAGCTAAG	No	No	Yes	0	0	0	No	
NM_hsa_5107	chr15:85259724-85259864	+	CTCTGGTCGAGCAAGTCTCCTGC	No	No	Yes	0	0	0	No	
NM_hsa_5111	chr15:41860977-41861115	+	CCCAGCTGGAGGCCTTCTCCCC	No	No	Yes	0	0.0662225	0	No	
NM_hsa_5112	chr15:64752769-64752849	+	TTGTCCCGGATCGCGGCGGCGGC	No	No	Yes	0	0	0	No	
NM_hsa_5113	chr15:83424733-83424847	+	CAAGACCTACTTATCTACCAACAGC	No	No	Yes	1.1967	0.331113	0.806149	Yes	
NM_hsa_5116	chr14:82806852-82806972	-	CTTCTATTGAACACGGACTGTAT	No	No	Yes	0	0	0	No	
NM_hsa_5119	chr14:101347306-101347434	+	TTGTCCTGCTTGTCCTGCGAGG	No	No	Yes	0.455886	0.0662225	0.439717	No	
NM_hsa_5120	chr14:100437709-100437827	+	AGGCGCGGACGGTGGGGCCCCG	No	No	Yes	0	0	0	No	
NM_hsa_5122	chr14:103804255-103804385	+	CCGTTATATAGACTTAAACAGTG	No	No	Yes	0	0	0	Yes	
NM_hsa_5125	chr14:24541908-24542026	+	CTCCCCTGCTCAGAACTCTGGT	No	No	Yes	0	0	0	No	
NM_hsa_5134	chr14:20811312-20811426	-	GGGGCCCGGCGGATGCCTCC	No	No	Yes	0	0	0	No	
NM_hsa_5135	chr14:101012826-101012966	+	TGGCGCGTGGAGTCGAACTCGGT	No	No	Yes	0	0	0	Yes	
NM_hsa_5139	chr14:102684350-102684482	+	TCCTGGATGTTGTGGCTGTGTAGA	No	No	Yes	0	0	0	No	
NM_hsa_5142	chr14:94802175-94802267	+	ATGTGTAGAGCCAGAAAGTGGACC	No	No	Yes	0	0	0	No	
NM_hsa_5144	chr14:82806854-82806974	-	TGCTTCTATTGAACACGGACTGT	No	No	Yes	0	0	0	No	
NM_hsa_5145	chr14:93581797-93581879	+	CCTGAGTCTGAGTCGGAGCTGGGG	No	No	Yes	0	0	0	No	
NM_hsa_5149	chr14:23342431-23342549	+	TGTCCTACCTTCCTCTCTCCAG	No	No	Yes	0	0	0	Yes	
NM_hsa_5152	chr14:94802167-94802295	-	ATGTGTAGAGCCAGAAAGTGGA	No	No	Yes	0	0	0	No	
NM_hsa_5156	chr17:75158275-75158397	-	TACCATCTGTGGGATTATGACTGA	No	No	Yes	25.3919	16.5887	22.7859	Yes	
NM_hsa_5157	chr17:8125763-8125885	+	ACAGAGTGCCTTCAGGACTGCAGA	No	No	Yes	0	0	0	No	
NM_hsa_5159	chr17:60766791-60766913	+	GAACCCTGACCCCTTCCCTGTAGA	No	No	Yes	0	0.198668	0	No	
NM_hsa_5160	chr17:79825010-79825154	+	GCTGTGAAGGGCTGGTGCTCTAGGC	No	No	Yes	0	0	0	Yes	
NM_hsa_5161	chr17:27075456-27075534	+	TTGCAGGGTAGGTGACAGGTAG	No	No	Yes	0	0	0	No	
NM_hsa_5164	chr17:27075452-27075532	+	CTCCTGCCTCTCTACTTCTGTGG	No	No	Yes	0	0	0	No	
NM_hsa_5168	chr17:59438861-59439003	+	TAATATATCTGTGGACACATCTGA	No	No	Yes	0	0	0	No	
NM_hsa_5169	chr17:47269793-47269911	+	GAGACTCGCGGCCGCCTTAAGA	No	No	Yes	0.170957	0.132445	0.0732862	Yes	KapB+cMyc>KapB>H
NM_hsa_5171	chr17:1547592-1547702	+	CTGGCTTTCTCCTGATCTCAGGC	No	No	Yes	0.256436	0.430446	0.109929	No	
NM_hsa_5175	chr17:4621097-4621207	+	TCCTGCCCCTACTCTGATCCCAG	No	No	Yes	1.02574	0.463558	0.293145	Yes	KapB+cMyc>KapB>H
NM_hsa_5178	chr17:17991694-17991822	+	CTGGGGCCTGGGTCTACTCTGT	No	No	Yes	0.0569857	0	0.219859	Yes	
NM_hsa_5179	chr17:44302652-44302772	-	AGGTCCGGGGCTGGGGGAGCTGC	No	No	Yes	0	0	0	No	
NM_hsa_5181	chr17:45026434-45026574	+	AACTGGGGACCAGGCGTGGCTGG	No	No	Yes	0	0.0662225	0	No	

Additional file 5

Name	locus	strand	sequence	mirdeep2	mireap	miranalyzer	RPM_HU_KapB_cMyc	RPM_HU_KaposinB	RPM_HUVEC	IP_confidence	annotation
NM_hsa_5182	chr17:20841686-20841816	+	ATAATAGTCTCCAACTCTGATTG	No	No	Yes	0	0.0331113	0	Yes	
NM_hsa_5184	chr17:26926128-26926222	+	ACCTCAGACACCGGCGGTAGGGCTG	No	No	Yes	0.170957	0	0	No	
NM_hsa_5185	chr17:63119528-63119636	+	GTCGAGCGGCGGAGCCTGTGGT	No	No	Yes	0	0	0	No	
NM_hsa_5186	chr17:19045346-19045454	-	CTGAGCAAACTCACAAGGCTGG	No	No	Yes	0	0	0	Yes	
NM_hsa_5186	chr17:18935718-18935826	+	CTGAGCAAACTCACAAGGCTGG	No	No	Yes	0	0	0	Yes	
NM_hsa_5189	chr17:46114780-46114920	+	CTCTCCGGTCGGCGCTGGGACGA	No	No	Yes	0	0.0662225	0.0732862	Yes	H>KapB>KapB+cMyc
NM_hsa_5190	chr17:16343309-16343461	+	CTGCTCTGATGAAATCACTAATAGGAAGT	No	No	Yes	7.09472	9.33737	9.52721	Yes	H>KapB>KapB+cMyc
NM_hsa_5192	chr17:31149412-31149526	+	TCGTCTTCCTCCCTCCCGGC	No	No	Yes	0	0	0	No	
NM_hsa_5194	chr17:6347731-6347871	+	GTGAGTGCGGGCTGGGCACAAGT	No	No	Yes	0.0284929	0.0662225	0	Yes	
NM_hsa_5196	chr17:37043120-37043204	+	TAAAAACAGGCTCTCGCTCTGTAGC	No	No	Yes	0.569857	0.662225	0.366431	Yes	
NM_hsa_5198	chr17:81037582-81037722	-	CCGGCTCCGCCGCCCGGCCCCGC	No	No	Yes	2.47888	1.22512	0.842792	No	KapB+cMyc>KapB>H
NM_hsa_5199	chr17:36888028-36888140	-	GAAATACCAGGTGCTGTAGGCTTT	No	No	Yes	202.915	116.183	104.291	Yes	KapB+cMyc>KapB>H
NM_hsa_5201	chr17:8534364-8534480	-	GCGCCGGCCGCCGCGGGGCCC	No	No	Yes	0.170957	0.0662225	0	Yes	KapB+cMyc>KapB>H
NM_hsa_5202	chr17:17739712-17739838	+	TGTCCCGGCCCGGAGCCGCCA	No	No	Yes	0.740814	0.596003	0.659576	Yes	
NM_hsa_5203	chr17:78389280-78389384	+	AGGGACTGTGGCCTCGGCGGTATGT	No	No	Yes	0	0	0	Yes	
NM_hsa_5204	chr17:4458709-4458849	+	GCCGTAGCTCTGAGCGGAAGTCC	No	No	Yes	0	0	0	No	
NM_hsa_5205	chr17:27507523-27507611	+	CTCCGTCCGCTGTGGACTCCGA	No	No	Yes	0	0	0	No	
NM_hsa_5206	chr17:29421336-29421474	+	CACCGGGTCTAGCATTGGGATT	No	No	Yes	0	0	0	Yes	
NM_hsa_5208	chr17:39676661-39676799	+	TGTCGTTGGGCATCAGAACATG	No	No	Yes	0	0	0	No	
NM_hsa_5209	chr17:18886816-18886958	+	ATGCATCCTTGACCAGGTCTGAGC	No	No	Yes	0	0	0	No	
NM_hsa_5210	chr17:65822043-65822163	-	GGGGGGGGCCGGCGGCGGCGGCG	No	No	Yes	0.159877	0.13686	0.0299611	Yes	KapB+cMyc>KapB>H
NM_hsa_5212	chr17:62223671-62223791	+	GACGCGCTGTCTTTGAGCCCCCGCCGAG	No	No	Yes	4.10297	3.11246	4.03074	Yes	
NM_hsa_5214	chr17:18066959-18067079	+	AGTGGGGCCAGAAGGACAGAGGT	No	No	Yes	0	0	0	No	
NM_hsa_5216	chr17:7748348-7748460	+	GCTCCGGACTGGAAGCCTGGCAGC	No	No	Yes	0.170957	0	0.0732862	No	
NM_hsa_5217	chr17:65628053-65628151	+	ATTCTTGCGCATTCTGAGGCTG	No	No	Yes	0	0	0.0732862	Yes	
NM_hsa_5220	chr16:11891318-11891426	+	CTCGCCTGGGCCGCGGCCTGAG	No	No	Yes	0	0.198668	0.146572	No	
NM_hsa_5221	chr16:87887439-87887577	+	CTGCGGAGACACTGAGCACTGC	No	No	Yes	0.0569857	0	0.146572	No	
NM_hsa_5222	chr16:87799640-87799742	+	CGAGGTTCGGTCTTGGAGCTCCGT	No	No	Yes	0	0.198668	0.0732862	No	
NM_hsa_5223	chr16:33963171-33963263	+	TTGGGAGCGGGCGGGCAGTCCGCC	No	No	Yes	0.455886	0.397335	0.366431	Yes	KapB+cMyc>KapB>H
NM_hsa_5224	chr16:12594519-12594659	+	GAGCTTGTCGGATGTGCAGATTT	No	No	Yes	0	0	0	Yes	
NM_hsa_5227	chr16:78414378-78414468	+	ACTCTTTAAGGATAGGGCTGAAT	No	No	Yes	0	0	0	Yes	
NM_hsa_5228	chr16:33966238-33966382	+	GTCCGCCATCGCGCGCCCGCCTCCG	No	No	Yes	0	0	0	No	
NM_hsa_5229	chr16:50903350-50903434	-	ATACCGGGTGCTGTAGGCTTAAAAA	No	No	Yes	420.624	273.136	147.951	Yes	KapB+cMyc>KapB>H
NM_hsa_5231	chr16:33966232-33966378	+	GTGTCGTCCGCCATCGCGCGCCCGCC	No	No	Yes	0.113971	0.397335	0.366431	No	
NM_hsa_5232	chr16:33963137-33963285	+	GCCACTGCCCGTCCCCGCCCCTTGCCT	No	No	Yes	3.76106	2.91379	2.93145	Yes	
NM_hsa_5233	chr16:2128374-2128464	+	CATCCACCTCCTCACCTCAGGCC	No	No	Yes	0	0	0	Yes	
NM_hsa_5235	chr16:67969865-67969955	+	GCGCAGGATGCGAGTGACCGTGG	No	No	Yes	0	0	0	No	
NM_hsa_5236	chr16:89988964-89989058	+	TCTTGCCTGGCTTGTGCGTCCTTGT	No	No	Yes	0.142464	0.0662225	0.0732862	Yes	
NM_hsa_5237	chr16:33963143-33963271	+	GCGAGCCACTGCCCGTCCCCGC	No	No	Yes	0	0.662225	0.293145	Yes	
NM_hsa_5239	chr16:33966039-33966153	+	GTCCCGGTCGCCGCGGTTCGCCGCC	No	No	Yes	2.07998	2.48334	1.24587	Yes	
NM_hsa_5241	chr16:33964195-33964329	+	CTCTGATCAGCCCCGCCGGG	No	No	Yes	0.284929	0.662225	0.293145	No	
NM_hsa_5243	chr16:33963388-33963496	+	TAGAGGTGAAATTCTTGGACCGGCGCA	No	No	Yes	14.0755	7.74803	8.72106	Yes	
NM_hsa_5244	chr16:33963146-33963264	+	GCTCCTAGTTGGATCTTGGGAGCGGGC	No	No	Yes	23.82	12.5161	6.52248	Yes	KapB+cMyc>KapB>H
NM_hsa_5245	chr16:30957964-30958080	+	ACCTCTCATCTGGCTGCCCAG	No	No	Yes	0	0	0	No	
NM_hsa_5246	chr16:85507455-85507583	+	AGCTGGTCCAATGCCTGGGAGA	No	No	Yes	0	0	0.0732862	Yes	
NM_hsa_5247	chr16:33966038-33966146	+	GAGCCCGTCCCGGTCGCCGCGGTTCGC	No	No	Yes	0.769307	1.15889	1.68558	Yes	H>KapB>KapB+cMyc
NM_hsa_5248	chr16:27561484-27561610	+	CCCCTCCGGGAGGTCGGGCTGTTATT	No	No	Yes	0.0569857	0	0	No	
NM_hsa_5249	chr16:33965485-33965597	+	CAGATTGATCATCGACACTTCGAACGCAA	No	No	Yes	17.4946	15.2974	24.1112	Yes	
NM_hsa_5251	chr16:22206996-22207126	-	GGTTCGAATCCCACCGCTGCCAC	No	No	Yes	0.0569857	0.132445	0.146572	Yes	H>KapB>KapB+cMyc
NM_hsa_5252	chr16:50903359-50903435	-	AGGGAATACCGGGTGCTGTAGGCTTA	No	No	Yes	2644.2	2172.24	1560.78	Yes	KapB+cMyc>KapB>H
NM_hsa_5253	chr19:35652414-35652504	+	TCATGTCTGAACCAATGAGAGCC	No	No	Yes	0.712322	0.0993338	0.146572	Yes	
NM_hsa_5254	chr19:3201433-3201551	+	TTGCCTCTCCCGTCCCTGTAGT	No	No	Yes	6.04049	4.63558	2.41845	Yes	KapB+cMyc>KapB>H
NM_hsa_5259	chr19:1253321-1253461	+	CTGGGAGGCGGTCGGTTCTGAGT	No	No	Yes	0	0	0	Yes	
NM_hsa_5264	chr19:24185902-24185988	-	CCAGAGTCGGGTTGCTTGGGAATGCA	No	No	Yes	1.42464	0.26489	0.659576	Yes	
NM_hsa_5265	chr19:17581269-17581365	+	GAGCGGGTCGTGGGGCGGGGC	No	No	Yes	0.683829	0.198668	0.219859	Yes	
NM_hsa_5268	chr19:53700354-53700438	+	GCGGATCGCGGGGAGTGAAGGCTGC	No	No	Yes	0	0	0	No	
NM_hsa_5271	chr19:14192493-14192583	+	CGAGGCCGGGCTGCACTGCGAGA	No	No	Yes	2.64984	2.08601	3.9941	Yes	
NM_hsa_5281	chr19:50357813-50357949	+	CGCCCCTCCTGCCCCCACAGA	No	No	Yes	0	0	0.0732862	Yes	
NM_hsa_5287	chr19:12606239-12606349	+	GCCTCGAACGTCGCTAGGTGGCT	No	No	Yes	0	0	0	No	
NM_hsa_5288	chr19:39926776-39926896	+	TCCTCTGTAGTCGACTACCGCAG	No	No	Yes	0.455886	0.331113	0.513004	No	
NM_hsa_5290	chr19:48981443-48981561	+	TCTGTCCACCTTGCTTCTTCAG	No	No	Yes	0	0	0	Yes	
NM_hsa_5295	chr19:39214988-39215088	+	TCCCACACCGCGTCTCCTCTGCC	No	No	Yes	0.113971	0	0.0366431	Yes	
NM_hsa_5298	chr19:35500708-35500816	+	CTCACCTCTCAAACTTCCTCAG	No	No	Yes	0	0	0	Yes	
NM_hsa_5300	chr19:42855296-42855382	+	CTCTGCCCAACTGACCCCCAG	No	No	Yes	0	0	0	No	
NM_hsa_5306	chr19:12897900-12898040	+	TAGCTGAACTCTAGCCTGAGCAA	No	No	Yes	0	0.0331113	0.0244288	Yes	
NM_hsa_5308	chr19:49993917-49994009	+	CACATGCACTACCATCTGAGGCCA	No	No	Yes	5.5846	6.42358	5.20332	Yes	
NM_hsa_5310	chr19:38943362-38943498	+	ACAGCTGCGAGGTCCCTGTAG	No	No	Yes	0	0	0	No	
NM_hsa_5315	chr19:54940961-54941091	+	TGCTGTGGGAGTGTGGAATCGGG	No	No	Yes	0	0	0	No	
NM_hsa_5318	chr18:34833624-34833752	+	CCCCCGTCCCCGCGCCCCGGCC	No	No	Yes	0	0	0	No	
NM_hsa_5319	chr18:35147120-35147260	+	GCCGCCGGACCGAGACTCGCGCG	No	No	Yes	0	0	0	No	
NM_hsa_5320	chr18:30350188-30350288	+	AGCAGCTTGTCGTCGGGGGAGGA	No	No	Yes	0	0	0	No	
NM_hsa_5322	chr18:33161789-33161871	-	CTAGGCGCGCGGCGGCGGCGGCGG	No	No	Yes	3.26645	1.9943	1.41465	Yes	KapB+cMyc>KapB>H
NM_hsa_5323	chr18:53146428-53146544	-	TTCATTGGACTGCTGATGGCCTGTCA	No	No	Yes	1.16821	1.09267	2.80931	No	
NM_hsa_5324	chr18:21750452-21750546	-	GGGAATACCAGGTGCTGTCGGCTTT	No	No	Yes	0.563073	0.739485	0.183216	Yes	
NM_hsa_5325	chr18:77748604-77748688	+	CGAACGTGTAGTTGGCCGGGTGGAG	No	No	Yes	0	0	0	No	
NM_hsa_5328	chr18:35424892-35424990	+	CTTGCCTCTTGGAGTTGTAGGA	No	No	Yes	0	0	0	No	
