# Supplementary material for: c-Myc and viral cofactor Kaposin B co-operate to elicit angiogenesis through modulating miRNome traits of endothelial cells
Source: BMC Syst Biol. 2016 Jan 11;10(Suppl 1):1. doi: 10.1186/s12918-015-0242-3 (PMC4895700; doi:10.1186/s12918-015-0242-3)
Supplement: Additional file 6: — List of Kaposin B and c-Myc down-regulated novel miRNAs which exist in Ago1/2-IP-seq data. (PDF 79 kb) [file 12918_2015_242_MOESM6_ESM.pdf]

Additional file 6

Name	locus	strand	sequence	HU_KapB_cMyc	HU_KaposinB	HUVEC	IP_confidence	annotation
NM_hsa_894	chr12:53856935-53857015	+	TGGGCTGTGGTGCCGTGACCTTT	0	0.0662225	0.439717	Yes	KaposinB+c-Myc<KaposinB
NM_hsa_3498	chr22:46508621-46508711	-	CTATACAACCTACTACCTCAC	0	0.0220742	0.097715	Yes	KaposinB+c-Myc<KaposinB
NM_hsa_4297	chr9:96938234-96938324	-	CTATACAACCTACTACCTCA	0	0.0220742	0.097715	Yes	KaposinB+c-Myc<KaposinB
NM_hsa_4749	chr1:144839419-144839519	-	TCAGAGTTCAAGTCTCACTGGGA	0	0.0331113	0.146572	Yes	KaposinB+c-Myc<KaposinB
NM_hsa_2482	chr12:123756348-123756444	+	GCGGGGCGGCCGGGGCCGGG	0	0.0331113	0.0732862	Yes	KaposinB+c-Myc<KaposinB
NM_hsa_4415	chr20:37078067-37078169	+	AAATGCTGGCGCTTTCACACACTC	0	0.0662225	0.146572	Yes	KaposinB+c-Myc<KaposinB
NM_hsa_1443	chr3:160118981-160119039	+	TGTGAATTTTTGTTTGTGACCTG	0	0.0496669	0.109929	Yes	KaposinB+c-Myc<KaposinB
NM_hsa_1	chrM:15955-16024	-	GTTAAAGACTTTTTCTCTGAT	0.113971	0.26489	1.24587	Yes	KaposinB+c-Myc<KaposinB
NM_hsa_2	chrM:15955-16024	-	CAGAGAATAGTTTAAATTAGA	0.512871	0.927115	1.46572	Yes	KaposinB+c-Myc<KaposinB
NM_hsa_2714	chr16:3208908-3208995	+	GTCCTGGCTCGTTGGTCTAGGGG	0.455886	0.816744	2.87038	Yes	KaposinB+c-Myc<KaposinB
NM_hsa_4564	chr6:28663697-28663809	-	GTCAGGGGGTGTAGATCAGTGGTA	17.1717	27.6369	80.3217	Yes	KaposinB+c-Myc<KaposinB
NM_hsa_3645	chr2:117475329-117475394	-	TCGAATCCTGTTCGTGACGCC	1.05424	1.65556	2.78488	Yes	KaposinB+c-Myc<KaposinB
NM_hsa_4803	chr2:230045621-230045749	-	GGGGCCCGTGCCTTGGAAAGCGTCGCT	6.83829	10.2645	24.1112	Yes	KaposinB+c-Myc<KaposinB
NM_hsa_4018	chr6:28626018-28626100	-	GAGGTGGGGATGTAGCTCAGTGGT	140.517	196.239	593.179	Yes	KaposinB+c-Myc<KaposinB
NM_hsa_4617	chr8:103425224-103425340	+	TTTCGCTCGGATTCCCCTGGGGCAGC	0.142464	0.198668	0.439717	Yes	KaposinB+c-Myc<KaposinB
NM_hsa_3964	chr6:26328352-26328450	+	GTCAGAGGGCCAGTGGCGCAATGGA	17.8365	23.1117	60.3879	Yes	KaposinB+c-Myc<KaposinB
NM_hsa_4993	chr11:75946899-75947005	-	AGATGGCTCGTTGGTCTAGGGGTATG	1.9945	2.53853	4.11624	Yes	KaposinB+c-Myc<KaposinB
NM_hsa_2715	chr16:3214929-3215020	+	GCCTGGCTAGCTCAGTCGGC	10.4854	13.1783	42.506	Yes	KaposinB+c-Myc<KaposinB
NM_hsa_4568	chr6:28795184-28795276	-	GGGGGTATAGCTCAGTGGTAGAGA	38.2279	46.8414	96.8844	Yes	KaposinB+c-Myc<KaposinB
NM_hsa_3880	chr5:122403755-122403839	+	GCGCGCACGCGCGCGTGTGTGGTG	0.0189952	0.0220742	0.0732862	Yes	KaposinB+c-Myc<KaposinB
NM_hsa_4039	chr7:780250-780368	+	CCGGCCACGCGCCTCTGTCTTG	0.0569857	0.0662225	0.366431	Yes	KaposinB+c-Myc<KaposinB
NM_hsa_4602	chr7:129414728-129414870	+	TTATGGCCCTTCGGTAATTCACTG	0.0569857	0.0662225	0.219859	Yes	KaposinB+c-Myc<KaposinB
NM_hsa_830	chr3:48357870-48357935	+	TCTAGCCTCCATGAATTCTGAT	0.370407	0.430446	0.659576	Yes	KaposinB+c-Myc<KaposinB
NM_hsa_4459	chr21:9827260-9827348	+	GGCTGATCGCACGCCCCCCGTGGCGGC	1.82354	2.0529	3.44445	Yes	KaposinB+c-Myc<KaposinB
NM_hsa_4003	chr6:127440552-127440625	+	CACCCTTGCCTCTCGGCGCCC	3.36216	3.64224	5.64304	Yes	KaposinB+c-Myc<KaposinB
NM_hsa_2539	chr13:42030048-42030148	-	GTTCGATTCCCGGTCAGGGAACAA	13.1067	13.9729	32.6124	Yes	KaposinB+c-Myc<KaposinB
NM_hsa_3199	chr1:112162347-112162425	+	TCTGGAGGAGGCGCCGCCGC	7.23719	7.48314	28.0686	Yes	KaposinB+c-Myc<KaposinB
NM_hsa_4445	chr21:9826410-9826542	+	CGCGCCCGCCGGCGGGGCTCGGAGCCGGG	0.512871	0.52978	1.09929	Yes	KaposinB+c-Myc<KaposinB
NM_hsa_3824	chr4:147607357-147607441	+	TTGTCTTTTTCTCTGACCAC	0.3989	0.397335	0.732862	Yes	
NM_hsa_2936	chr18:24128979-24129074	+	GGGGGGTCGGGGCGGCGGCT	1.36766	1.35756	2.74823	Yes	
NM_hsa_1622	chr12:97983686-97983744	+	AAATGTAATTGTGGTTTTTGCC	0.0262739	0.026016	0.133137	Yes	
NM_hsa_3728	chr3:176232880-176232951	+	TTTCCCAGCCAACGCACCAA	50.6603	48.7729	84.0227	Yes	
NM_hsa_4944	chr10:60447670-60447798	+	AACCTCCCCCGAGTGTTACAGC	0.284929	0.26489	1.02601	Yes	
NM_hsa_5271	chr19:14192493-14192583	+	CGAGGCCGGGCTGCACTGCGAGA	2.64984	2.08601	3.9941	Yes	
NM_hsa_334	chr9:37804224-37804280	-	TCTTTAGGAACTCTGGCTAACT	0.0427393	0.0331113	0.109929	Yes	
NM_hsa_4669	chr9:37804198-37804314	-	TTTCTTTAGGAACTCTGGCTAACTAT	0.0427393	0.0331113	0.109929	Yes	
NM_hsa_4426	chr20:2636705-2636851	+	TCACGGTGATGGCTGACCAGGGCTCC	0.170957	0.132445	0.513004	Yes	
NM_hsa_4238	chr9:86535927-86536016	+	TGCGCTCTGAAAGGCCGGGTCC	0.170957	0.132445	0.293145	Yes	
NM_hsa_4502	chr5:71146770-71146850	+	GGGAGCCCCGGGGAGAGTTCTCT	0.683829	0.52978	1.09929	Yes	
NM_hsa_2260	chr10:101295022-101295110	-	CGGCGGCGGCGGGGGCGCGTG	0.00712322	0.00413891	0.082447	Yes	
NM_hsa_3556	chr2:101619083-101619167	+	TCTGAGCCTCCTTGTCGCCTGC	0.626843	0.331113	1.09929	Yes	
NM_hsa_4235	chr9:43128014-43128100	+	AAAAGGAGAACTTTGAAGGA	2.50737	1.32445	3.95746	Yes	
NM_hsa_3191	chr1:96079530-96079609	+	GCGCGCGCGCGTGTGTGGTGTG	0.113971	0.0551854	0.329788	Yes	
NM_hsa_4672	chr9:37804197-37804309	+	TCTTCAGGAACTCTGGCTAACTAT	0.0712322	0.0331113	0.146572	Yes	
NM_hsa_758	chr9:37804215-37804292	+	TCTTCAGGAACTCTGGCTAACT	0.0712322	0.0331113	0.146572	Yes	
NM_hsa_3906	chr5:166405925-166406021	+	GTGCGCGCGCGCGCGTGTGTTGTG	0.132967	0.0551854	0.329788	Yes	
NM_hsa_632	chr17:73402150-73402240	-	TCCGGTCCGTACAAACTCTGCA	0.227943	0.0662225	0.366431	Yes	
NM_hsa_1285	chr6:33169450-33169518	+	TGGGAGAGAGAAGGGCTGGTTCT	0.284929	0.0662225	0.58629	Yes	
NM_hsa_1130	chr1:193126362-193126490	+	CCTGTTCGTAAATGTTAGTGGA	0	0	0.0732862	Yes	
NM_hsa_1237	chr4:142629145-142629206	+	CAGCAAGGCCATGCAGCTCTGC	0	0	0.0732862	Yes	
NM_hsa_1336	chr17:46114826-46114881	+	TCTCCGGTCGGCGCTGGGACGA	0	0	0.0732862	Yes	
NM_hsa_1617	chr7:63811988-63812051	+	AATTAGGCCCAACAGTTCCCGT	0	0	0.0732862	Yes	
NM_hsa_1731	chr10:114468976-114469036	+	AACACCTCAGAAAAGGGACAGA	0	0	0.146572	Yes	
NM_hsa_1776	chr7:74140308-74140373	+	CTTCCCTTCCCCTGGGCCTCAGC	0	0	0.293145	Yes	
NM_hsa_1921	chr17:37043135-37043194	+	AAAACAGGCTCTCGCTCTGTAG	0	0	0.146572	Yes	
NM_hsa_2243	chr10:50377361-50377454	-	AGGGTTGGGTGGAGGATGTCT	0	0	0.109929	Yes	
NM_hsa_2530	chr13:97079472-97079558	+	TTATGTGACCTCGGATGAAT	0	0	0.146572	Yes	
NM_hsa_263	chrX:134459119-134459179	-	TGCATGCCAGCCACTGTACTGA	0	0	0.0732862	Yes	
NM_hsa_2994	chr19:10939643-10939722	+	TCCCCAACCCCTGCCCGCAGA	0	0	0.0732862	Yes	
NM_hsa_3225	chr1:156186286-156186380	+	CTGGCCTACCGGTGACCCGG	0	0	0.0732862	Yes	
NM_hsa_3316	chr1:117210401-117210485	-	GGCGGGGCCGGGGGCGGGGCCG	0	0	0.0732862	Yes	
NM_hsa_3325	chr1:153278768-153278852	-	GAGAGGGAAAGAGAGAGTGGG	0	0	0.0732862	Yes	
NM_hsa_3729	chr3:178866226-178866356	+	CGCCCGCTCTCCTCTCCCTCGGC	0	0	0.0732862	Yes	
NM_hsa_3736	chr3:186504502-186504594	+	CAGCACTAGATTGTAAAGACTGG	0	0	0.293145	Yes	
NM_hsa_3840	chr4:84437523-84437597	-	ATCCCATTGCCAGGATTTAT	0	0	0.0732862	Yes	
NM_hsa_3860	chr5:14871905-14871990	+	ACGAGGCGCCGCCGCCGCCG	0	0	0.0732862	Yes	
NM_hsa_3912	chr5:180229852-180229924	+	GGCGGGGCGGGGCGGACTGA	0	0	0.0732862	Yes	
NM_hsa_3959	chr6:21686023-21686094	+	TCACTTCCTGTTCTGTGGCCTGT	0	0	0.439717	Yes	
NM_hsa_3978	chr6:32147587-32147664	+	TTTCTCTCTCCACTTCCTCAGA	0	0	0.0732862	Yes	
NM_hsa_4147	chr8:27325578-27325662	+	TGTGGCAGTAGGTTGTGTTA	0	0	0.0732862	Yes	
NM_hsa_4338	chrX:52964418-52964514	+	ATCGGGGTTCGCCGCAGGCT	0	0	0.0732862	Yes	
NM_hsa_4346	chrX:107740993-107741070	+	AATTGTTACTCTGAAACCTGC	0	0	0.0732862	Yes	
NM_hsa_4441	chr21:9825733-9825811	+	GGGGGTCGGCCTGCGGCGCGTGCGGGG	0	0	0.219859	Yes	
NM_hsa_4594	chr7:98617315-98617421	+	ACATCAGAGCGCAGGACTGTGTCTGC	0	0	0.146572	Yes	
NM_hsa_4666	chr9:139760865-139760961	+	GACGTCGGGGCCTGGCCTGGC	0	0	0.0732862	Yes	
NM_hsa_4774	chr1:156186242-156186330	+	CTCTGGCCTACCGGTGACCCGG	0	0	0.0732862	Yes	
NM_hsa_4949	chr10:23003584-23003700	+	CTGCGCCTTCCCGCCCCGGCT	0	0	0.0732862	Yes	
NM_hsa_5048	chr12:51663543-51663649	+	CTTCTGGCTCGCCTCGGACGC	0	0	0.0732862	Yes	
NM_hsa_5217	chr17:65628053-65628151	+	ATTCTTGCGCATTCTGAGGCTG	0	0	0.0732862	Yes	
NM_hsa_5246	chr16:85507455-85507583	+	AGCTGGTCCAATGCCTGGGAGA	0	0	0.0732862	Yes	
NM_hsa_5281	chr19:50357813-50357949	+	CGCCCCTCCTGCCCCCACAGA	0	0	0.0732862	Yes	
NM_hsa_574	chr17:1633610-1633673	+	TCAGGGCCTGCTCCCACCCCGCA	0	0	0.0732862	Yes	
NM_hsa_612	chr10:89307592-89307647	+	ATAGAGCCATCGATCAATATGT	0	0	0.0732862	Yes	
NM_hsa_803	chr19:4215924-4215976	+	CACGGGGGCTGAGAGCAGAACC	0	0	0.0732862	Yes	
NM_hsa_4607	chr8:128750358-128750486	+	AGAGGTTCGGGACTGTGGCGCG	0.0569857	0	0.366431	Yes	
NM_hsa_4449	chr21:9826194-9826274	+	ACAGGCGTTCGTGCGACGTGTGGCGTGG	0.0142464	0	0.0732862	Yes	
NM_hsa_5178	chr17:17991694-17991822	+	CTGGGGCCTGGGTCTACTCTGT	0.0569857	0	0.219859	Yes	
NM_hsa_3487	chr22:24407605-24407699	-	GGCGGCGCGCGCGCGCGTGCGC	0.0569857	0	0.146572	Yes	
NM_hsa_3948	chr5:179316784-179316863	-	CTACAGCCTCCATCTCCCGGACT	0.0569857	0	0.146572	Yes	
NM_hsa_4661	chr9:30574375-30574507	-	CAGCTGGCGCTCTCGCAGT	0.0569857	0	0.146572	Yes	
NM_hsa_4854	chr3:178866109-178866207	+	GCCGCTGAGGTGTCGGGCTGCTGCTGC	0.0569857	0	0.146572	Yes	
NM_hsa_774	chr6:21686164-21686292	+	TAGGAGCATGAACCTGGTGAGC	0.0569857	0	0.146572	Yes	
NM_hsa_918	chr19:16198771-16198838	+	TGGGCCTTTCTGTCTCTGCAGG	0.227943	0	0.366431	Yes	
