# Supplementary material for: c-Myc and viral cofactor Kaposin B co-operate to elicit angiogenesis through modulating miRNome traits of endothelial cells
Source: BMC Syst Biol. 2016 Jan 11;10(Suppl 1):1. doi: 10.1186/s12918-015-0242-3 (PMC4895700; doi:10.1186/s12918-015-0242-3)
Supplement: Additional file 7: — List of Kaposin B and c-Myc up-regulated novel miRNAs which exist in Ago1/2-IP-seq data. (PDF 83 kb) [file 12918_2015_242_MOESM7_ESM.pdf]

Additional file 7

Name	locus	strand	sequence	HU_KapB_cMyc	HU_KaposinB	HUVEC	IP_confidence	annotation
NM_hsa_3797	chr4:13629077-13629147	+	CGGGGCCCGGCGGCGGCGGCGGT	0.0633175	0.0412051	0	Yes	KaposinB+c-Myc>KaposinB
NM_hsa_2842	chr17:36875913-36876031	+	AAGGGCCAGAGGAGCCTGGAGT	0.0854786	0.0331113	0	Yes	KaposinB+c-Myc>KaposinB
NM_hsa_3150	chr1:26881016-26881096	+	CCTCCTGCCCTCCTTGCTGTAG	0.0854786	0.0331113	0	Yes	KaposinB+c-Myc>KaposinB
NM_hsa_4884	chr3:182787676-182787778	+	AGTGGCTTAGATATGAACTCTGGA	0.113971	0.0662225	0	Yes	KaposinB+c-Myc>KaposinB
NM_hsa_1352	chr8:121511219-121511337	+	TTATGTCACTCCTTTGCCCAGA	0.170957	0.0662225	0	Yes	KaposinB+c-Myc>KaposinB
NM_hsa_1374	chr19:35500733-35500793	+	TCACCTCTCAAACTTCCTCAGA	0.170957	0.0662225	0	Yes	KaposinB+c-Myc>KaposinB
NM_hsa_5201	chr17:8534364-8534480	-	GCGCCGGCCGCCGCGGGGCCC	0.170957	0.0662225	0	Yes	KaposinB+c-Myc>KaposinB
NM_hsa_868	chr19:35652419-35652495	+	TCATGTCTGAACCAATGAGAGC	0.256436	0.0331113	0	Yes	KaposinB+c-Myc>KaposinB
NM_hsa_2856	chr17:42432096-42432164	+	GGTCGAAGGGCGGCGGCGGC	0.303924	0.110371	0	Yes	KaposinB+c-Myc>KaposinB
NM_hsa_1090	chr21:43741396-43741479	+	TCTCCCTTCCTGCCCTGGCCT	0.313421	0.0331113	0	Yes	KaposinB+c-Myc>KaposinB
NM_hsa_2182	chr10:29333102-29333178	+	TGGAGTGTGACAATGGTGTTAT	0.313421	0.0331113	0	Yes	KaposinB+c-Myc>KaposinB
NM_hsa_4713	chr1:149900241-149900381	+	TAGAACACTCTGGCCCCATCTGC	0.341914	0.0662225	0	Yes	KaposinB+c-Myc>KaposinB
NM_hsa_3678	chr3:38012172-38012248	+	CTACTCTTTGCACCCCTCTGC	0.7978	0.397335	0	Yes	KaposinB+c-Myc>KaposinB
NM_hsa_1953	chrY:19692289-19692341	-	GTCTGAGGAGCCAATGGGGT	0.854786	0.79467	0	Yes	KaposinB+c-Myc>KaposinB
NM_hsa_3970	chr6:28732097-28732196	+	GCCAAAATTGCTCAGTTGGGAGA	4.87228	3.67535	0.329788	Yes	KaposinB+c-Myc>KaposinB
NM_hsa_3949	chr6:393374-393467	+	AGCCGGCGGGGGCGCGCCGG	232.559	137.345	15.2435	Yes	KaposinB+c-Myc>KaposinB
NM_hsa_4113	chr7:26331600-26331683	-	GGCGCGCGGCGGCAGCGGCGG	0.186787	0.163717	0.0183216	Yes	KaposinB+c-Myc>KaposinB
NM_hsa_2631	chr14:74892786-74892879	-	GGGGCGGGGCGGCGGCGGCGGCG	0.498625	0.463558	0.0732862	Yes	KaposinB+c-Myc>KaposinB
NM_hsa_4817	chr2:130635315-130635453	+	GCGCGCGTGTCGGGGGGCGGGC	1.13971	0.397335	0.0732862	Yes	KaposinB+c-Myc>KaposinB
NM_hsa_3773	chr3:138624104-138624175	-	TGAGGTAGGAGGTTGCAGTGA	1.49113	0.960226	0.207644	Yes	KaposinB+c-Myc>KaposinB
NM_hsa_2506	chr12:127650709-127650800	-	TCGGTTGGCCTCGGATGGCCTC	3.81804	3.70846	0.806149	Yes	KaposinB+c-Myc>KaposinB
NM_hsa_5210	chr17:65822043-65822163	-	GGGGGGGGCCGGCGGCGGCGGCG	0.159877	0.13686	0.0299611	Yes	KaposinB+c-Myc>KaposinB
NM_hsa_3154	chr1:31381667-31381758	+	AGGAGGGCCCGGGGCGGGG	0.455886	0.331113	0.0732862	Yes	KaposinB+c-Myc>KaposinB
NM_hsa_4450	chr21:9826690-9826778	+	CGCGGGTCTGTGGCGCGGGGCC	0.341914	0.331113	0.0732862	Yes	KaposinB+c-Myc>KaposinB
NM_hsa_3332	chr1:167153912-167153981	-	TGAGGTAGTAGGTTGGGAGT	10.2448	7.33598	1.62858	Yes	KaposinB+c-Myc>KaposinB
NM_hsa_128	chr6:158914875-158914957	-	TGAGGTAGTAGGTGGTGTGC	10.3954	7.70178	1.78214	Yes	KaposinB+c-Myc>KaposinB
NM_hsa_3536	chr2:73246462-73246545	+	GCCGAAGTAGCTCAGTTGGGAG	48.9222	48.5742	11.396	Yes	KaposinB+c-Myc>KaposinB
NM_hsa_4644	chr9:79186665-79186763	+	CCCGCCGTGCGCCGGGACCGGGGTCCG	79.3526	57.15	14.071	Yes	KaposinB+c-Myc>KaposinB
NM_hsa_4531	chr6:28757511-28757605	-	GCTTTGCATGTATGAGGTCCCGGGT	37.5536	29.2041	7.62177	Yes	KaposinB+c-Myc>KaposinB
NM_hsa_4273	chr9:134153375-134153457	+	TCCGCAGGCGGCGGCGGCGGCGGC	0.205757	0.137716	0.0374307	Yes	KaposinB+c-Myc>KaposinB
NM_hsa_2828	chr17:18965290-18965366	+	CCCCGAGGAAGAGAGGTAGCG	0.569857	0.52978	0.146572	Yes	KaposinB+c-Myc>KaposinB
NM_hsa_1312	chr15:24920989-24921066	-	GCAGGGGCCGGCGGCGGCA	0.626843	0.26489	0.0732862	Yes	KaposinB+c-Myc>KaposinB
NM_hsa_3491	chr22:39711178-39711250	-	ACTGCCTTCCTTCTGAGAACA	0.341914	0.26489	0.0732862	Yes	KaposinB+c-Myc>KaposinB
NM_hsa_969	chr17:37043284-37043351	+	CAGGGTTTTGCAGACCGGGTCT	0.284929	0.26489	0.0732862	Yes	KaposinB+c-Myc>KaposinB
NM_hsa_4689	chr1:180910059-180910189	+	CACCCTCCCGGCCCTCCCCCGCC	3.5901	2.25157	0.659576	Yes	KaposinB+c-Myc>KaposinB
NM_hsa_4528	chr7:73062127-73062227	+	GGCGGGCGGCGGGCGGCGGGGGC	0.645838	0.52978	0.164894	Yes	KaposinB+c-Myc>KaposinB
NM_hsa_2185	chr10:35929773-35929871	+	GCCGGGCGGCGGCGGCGGCAGGC	0.374742	0.268832	0.0875969	Yes	KaposinB+c-Myc>KaposinB
NM_hsa_3619	chr2:240322061-240322158	+	ACCCCCGCGCCCCCGCCCCGGC	10.2859	5.76136	1.90544	Yes	KaposinB+c-Myc>KaposinB
NM_hsa_2164	chr3:186505088-186505150	+	AAAGCAGGATTCAGACTACAAT	1.76656	1.25823	0.439717	Yes	KaposinB+c-Myc>KaposinB
NM_hsa_3965	chr6:26328352-26328450	+	CAGGTTCGACTCCTGGCTGGCTCG	13.9615	9.90026	3.51774	Yes	KaposinB+c-Myc>KaposinB
NM_hsa_4791	chr16:33964220-33964318	+	CGCTGAGAAGACAGTCGAACTTGACTA	270.454	238.732	87.9435	Yes	KaposinB+c-Myc>KaposinB
NM_hsa_1054	chr16:57770361-57770435	+	TAGCTCTGTCCTTGGGAATCGC	0.740814	0.397335	0.146572	Yes	KaposinB+c-Myc>KaposinB
NM_hsa_884	chr1:150207085-150207139	+	TGAGGCCGAGAAGGCAACCGCGA	0.455886	0.397335	0.146572	Yes	KaposinB+c-Myc>KaposinB
NM_hsa_3296	chr1:35893406-35893490	-	TTGTGGGTTATTGTTAAGCTGAT	14.4744	10.3307	3.81089	Yes	KaposinB+c-Myc>KaposinB
NM_hsa_198	chr17:65822071-65822137	-	GGGGGGCCGGCGGCGGCGGCGG	0.168644	0.0927115	0.0355985	Yes	KaposinB+c-Myc>KaposinB
NM_hsa_3308	chr1:60809985-60810083	-	CACTAGTTTGTAGGTCCTTAA	33.4858	29.5816	11.9017	Yes	KaposinB+c-Myc>KaposinB
NM_hsa_1274	chr16:51272972-51273049	-	CACTAGATTGTGAGCTGCTTGAG	33.6282	29.6699	11.975	Yes	KaposinB+c-Myc>KaposinB
NM_hsa_4963	chr10:24497805-24497907	-	CCGTCCGTCCGTCCGTCCGTCCTC	14.1799	8.15641	3.49331	Yes	KaposinB+c-Myc>KaposinB
NM_hsa_1727	chr10:94624510-94624568	+	TCAGGTCTTCAGACTGTGGGG	0.3989	0.331113	0.146572	Yes	KaposinB+c-Myc>KaposinB
NM_hsa_2470	chr12:112354552-112354633	+	AGATGTAGTAGTTTGTGCTTT	178.804	162.278	72.678	Yes	KaposinB+c-Myc>KaposinB
NM_hsa_4795	chr2:223184096-223184244	+	CCTCGGCAGGCGGCGGCGGCGGCGGCT	0.319729	0.243672	0.110717	Yes	KaposinB+c-Myc>KaposinB
NM_hsa_2951	chr18:68879684-68879769	+	GGCGGCGGGGGGCGGGGCGGGGC	1.2005	0.708581	0.323681	Yes	KaposinB+c-Myc>KaposinB
NM_hsa_2719	chr16:9057259-9057333	+	CGCGGCGGGCGGCGGCGGCGGC	1.36006	0.805707	0.382957	Yes	KaposinB+c-Myc>KaposinB
NM_hsa_3403	chr20:54606257-54606348	-	TGAGGTAGTAGTTTGTGTCTT	325.458	293.394	147.771	Yes	KaposinB+c-Myc>KaposinB
NM_hsa_2154	chr1:70859275-70859325	+	TGAGGGAGTAGTTTGTGCAA	313.671	284.254	145.109	Yes	KaposinB+c-Myc>KaposinB
NM_hsa_5244	chr16:33963146-33963264	+	GCTCCTAGTTGGATCTTGGGAGCGGGC	23.82	12.5161	6.52248	Yes	KaposinB+c-Myc>KaposinB
NM_hsa_5254	chr19:3201433-3201551	+	TTGCCTCTCCCGTCCCTGTAGT	6.04049	4.63558	2.41845	Yes	KaposinB+c-Myc>KaposinB
NM_hsa_4913	chr4:1006300-1006418	-	TCGGGCGGCGGCGGCCGGCGGG	1.11122	0.695336	0.366431	Yes	KaposinB+c-Myc>KaposinB
NM_hsa_2798	chr16:88519967-88520040	-	GGGCGGCGGCGGGCGGCGGGGGC	0.968758	0.695336	0.366431	Yes	KaposinB+c-Myc>KaposinB
NM_hsa_3832	chr4:1006319-1006406	-	CGGGCGGCGGCGGCCGGCGGGA	0.940265	0.695336	0.366431	Yes	KaposinB+c-Myc>KaposinB
NM_hsa_5229	chr16:50903350-50903434	-	ATACCGGGTGCTGTAGGCTTAAAAA	420.624	273.136	147.951	Yes	KaposinB+c-Myc>KaposinB
NM_hsa_2566	chr14:65937572-65937650	+	CTGCACAGAGGGCGGCGGGCTGT	0.626843	0.26489	0.146572	Yes	KaposinB+c-Myc>KaposinB
NM_hsa_2910	chr17:38278611-38278692	-	GCCTCGCCGCCGCCTCTGCTGC	0.341914	0.26489	0.146572	Yes	KaposinB+c-Myc>KaposinB
NM_hsa_2399	chr11:97593310-97593396	-	TGGTAGTAGATTGTTTAGTG	0.256436	0.110371	0.0610719	Yes	KaposinB+c-Myc>KaposinB
NM_hsa_2707	chr16:2222119-2222202	+	TCAGCACGCCCTCCTCTCCCAGA	0.284929	0.132445	0.0732862	Yes	KaposinB+c-Myc>KaposinB
NM_hsa_2772	chr16:89297532-89297619	+	AAGGTCATCAGATTAGCGGGA	0.227943	0.132445	0.0732862	Yes	KaposinB+c-Myc>KaposinB
NM_hsa_2822	chr17:17598203-17598283	+	AGGCGGCGCGCATCTCTGGGC	0.227943	0.132445	0.0732862	Yes	KaposinB+c-Myc>KaposinB
NM_hsa_2991	chr19:9653379-9653476	+	TTACAGTGGAGGGCAAGTCTG	0.170957	0.132445	0.0732862	Yes	KaposinB+c-Myc>KaposinB
NM_hsa_5169	chr17:47269793-47269911	+	GAGACTCGCGGCCGCCTTAAGA	0.170957	0.132445	0.0732862	Yes	KaposinB+c-Myc>KaposinB
NM_hsa_5079	chrX:154003303-154003437	+	AAGTGGTGAGTTCTTCTGTCCAGCG	0.626843	0.52978	0.293145	Yes	KaposinB+c-Myc>KaposinB
NM_hsa_1520	chr1:18073682-18073757	+	TTAGGGTTTGCCTGGGACTGA	102.717	70.6594	40.3441	Yes	KaposinB+c-Myc>KaposinB
NM_hsa_4995	chr11:85195143-85195261	+	GGAGGATTCAACCCGGTGGCGG	9.28867	7.28448	4.17732	Yes	KaposinB+c-Myc>KaposinB
NM_hsa_3989	chr6:43762884-43762978	+	CCAGGCTCGGCTCCTGGCTG	1.22519	1.05956	0.622933	Yes	KaposinB+c-Myc>KaposinB
NM_hsa_2375	chr11:21022414-21022489	-	ACCGGGTGCTGTAGGCTGTC	77.4151	50.6657	31.8001	Yes	KaposinB+c-Myc>KaposinB
NM_hsa_3494	chr22:42519490-42519567	-	AAGGAGAGAGAACAGGCTGAGG	0.284929	0.231779	0.146572	Yes	KaposinB+c-Myc>KaposinB
NM_hsa_5175	chr17:4621097-4621207	+	TCCTGCCCCTACTCTGATCCCAG	1.02574	0.463558	0.293145	Yes	KaposinB+c-Myc>KaposinB
NM_hsa_4425	chr20:2634868-2635014	+	CTGATGTCTCCATGTCTCTGAGCAAT	0.512871	0.463558	0.293145	Yes	KaposinB+c-Myc>KaposinB
NM_hsa_4447	chr21:9826183-9826279	+	CGGACAGGCGTTCGTGCGACGTGTGG	0.0142464	0.0220742	0	Yes	
NM_hsa_190	chr17:79919290-79919367	+	GCGCCGCCGCCCCCCCCCGCG	0.0284929	0.0331113	0	Yes	
NM_hsa_3374	chr20:30194978-30195067	+	CTCCTGCGTAGGATCTGAGGA	0.0284929	0.0772596	0	Yes	
NM_hsa_35	chr19:7533656-7533712	+	TATCTGCTGTTGTCCCCTCAGG	0.0284929	0.0662225	0	Yes	
NM_hsa_5194	chr17:6347731-6347871	+	GTGAGTGCGGGCTGGGCACAAGT	0.0284929	0.0662225	0	Yes	
NM_hsa_1308	chr1:178712679-178712756	+	TTAGTTCTGTGGCAACATCGA	0.0569857	0.0662225	0	Yes	
NM_hsa_134	chr14:21460202-21460252	+	TCTGAGCCCTGTTCTCCCTAGG	0.0569857	0.132445	0	Yes	
NM_hsa_146	chr1:171560222-171560285	+	TAGGAGCTATCAGAACTTAGTGA	0.0569857	0.397335	0	Yes	
NM_hsa_1502	chr17:33760236-33760295	+	CACACTTCCCCAGCGTGGCCTGC	0.0569857	0.463558	0	Yes	
NM_hsa_269	chr10:72082639-72082769	-	CCCATCTGATGGGGAATGGCCTG	0.0569857	0.198668	0	Yes	
NM_hsa_3094	chr19:59061035-59061102	+	TCTGGGTGTTGGGCTGTCTGGA	0.0569857	0.132445	0	Yes	
NM_hsa_3634	chr2:75340449-75340537	-	GTGAGCCTTGAAGCCTTGAG	0.0569857	0.0662225	0	Yes	
NM_hsa_4312	chrM:8153-8229	-	TAGGACGATGGGCATGAAACTGTG	0.0569857	0.198668	0	Yes	
NM_hsa_520	chr19:5978291-5978403	+	CATCTCCCGCCTCCTTTTCCCGCC	0.0569857	0.331113	0	Yes	
NM_hsa_554	chr10:90957414-90957473	+	TCTGCAGGCTGGGATCTGGGAT	0.0569857	0.198668	0	Yes	
NM_hsa_4186	chr8:6333106-6333174	-	CTGGGGATAGAGCATTGCCCT	0.113971	0.132445	0	Yes	
NM_hsa_1324	chr19:13063444-13063504	+	CTTCCCCACCCTCTCCTGCAGC	0.170957	0.198668	0	Yes	
NM_hsa_970	chr17:37043284-37043351	+	GCCTGGCCTGTTTTAACCCTGTG	0.170957	0.26489	0	Yes	
NM_hsa_3185	chr1:67529202-67529298	+	TCAGACCTTTGAAGAGAGAG	0.227943	0.26489	0	Yes	
NM_hsa_4431	chr20:37078062-37078162	+	TTGCTGAAAGCCGTTTCCCGTGTTTGGA	0.341914	0.52978	0	Yes	
NM_hsa_4424	chr20:30194977-30195059	+	GCTCCTGCGTAGGATCTGAGGAGT	0.427393	1.26926	0.0732862	Yes	
NM_hsa_4735	chr1:231400529-231400645	+	TCATGTTGCTCTGCTGTTGCC	0.683829	1.05956	0.0732862	Yes	
NM_hsa_4650	chr9:34049953-34050083	+	TTGTCACCCCATAGATCGCCAGG	4.61584	6.15869	0.439717	Yes	
NM_hsa_4694	chr1:26881010-26881102	+	TCCTCCTGCCCTCCTTGCTGTAGA	0.313421	0.364224	0.0366431	Yes	
NM_hsa_4127	chr7:105924959-105925045	-	GGCGCGCGGCGGCGGCGGCGCG	0.265933	0.362384	0.0427503	Yes	
NM_hsa_899	chr15:75495340-75495407	+	TCCACTGGAGACTTTGGGCTGCCCC	0.455886	0.463558	0.0732862	Yes	
NM_hsa_4393	chrX:134562082-134562157	-	GGAATACCAGGTGCTATAGGCTT	0.0596993	0.154519	0.0244288	Yes	
NM_hsa_4483	chr22:46973037-46973165	+	CCGGCTCCCCGGCGCCGCTGGG	2.05149	2.18534	0.366431	Yes	
NM_hsa_3141	chr1:10571690-10571772	+	TGTGGACCCAGGGACCTCTGA	0.284929	0.860893	0.146572	Yes	
NM_hsa_3216	chr1:153508714-153508844	+	TCACCCCTGCTTCCTCTGCCAGA	0.170957	0.397335	0.0732862	Yes	
NM_hsa_3367	chr20:17662487-17662571	+	GCGGCGGGCGGCGGGCGGGCCG	0.0854786	0.18763	0.0366431	Yes	
NM_hsa_4690	chr1:95582796-95582908	+	TCGGCGGCCCGCCTCGGGGCCCCG	7.00924	7.41692	1.46572	Yes	
NM_hsa_4093	chr7:139876420-139876499	+	CGGGCGGCCGGCGGCGGCGG	0.0427393	0.087193	0.0183216	Yes	
NM_hsa_3765	chr3:104974866-104974953	-	GCTGTGATGGCCGAGTGGTTAAGG	0.113971	0.331113	0.0732862	Yes	
NM_hsa_4599	chr7:5820747-5820885	+	GCGGCCTCGGGCCCGGGGGAGG	0.227943	0.596003	0.146572	Yes	
NM_hsa_5324	chr18:21750452-21750546	-	GGGAATACCAGGTGCTGTCGGCTTT	0.563073	0.739485	0.183216	Yes	
NM_hsa_3544	chr2:87141955-87142032	+	TGGCCGGGCGGCGGCGGCGGCGGC	0.745855	0.873784	0.262873	Yes	
NM_hsa_2169	chr20:36065455-36065501	-	AGAGTTTGATCCTGGCTCAG	0.455886	0.463558	0.146572	Yes	
NM_hsa_4166	chr8:103425233-103425322	+	TTTCGCTCGGATTCCCCTGGGGC	0.142464	0.198668	0.0732862	Yes	
NM_hsa_4844	chrX:153996830-153996974	+	TTCATTTGCCTCCCAGCCTACAAAA	0.0569857	0.0993338	0.0366431	Yes	
NM_hsa_591	chr3:133785734-133785795	+	TCTGCTGAGAGTTTCTGACTGA	1.82354	2.0529	0.806149	Yes	
NM_hsa_4696	chr1:569763-569853	-	GTCATTAGGAGGGCTGAGAGGGC	1.42464	2.18534	0.879435	Yes	
NM_hsa_216	chr3:138553852-138553972	+	TCTACCGTTGCCAGCCCGGCTGC	0.455886	0.52978	0.219859	Yes	
NM_hsa_3969	chr6:28687504-28687569	+	GCATGCACGAGGCCCCGGGT	2.67833	2.78135	1.17258	Yes	
NM_hsa_4882	chr3:39452510-39452650	+	TGTGCACATTGTTAGAGCTTGGAGTTGA	4.84379	6.15869	3.00474	Yes	
NM_hsa_2152	chr13:113006387-113006454	+	AAGCTGCCAGTTGAACCACTGT	2.70682	2.88068	1.42908	Yes	
NM_hsa_4343	chrX:71476657-71476739	+	TCAGGCTCTGGCATGTTGGA	1.1967	1.32445	0.659576	Yes	
NM_hsa_5239	chr16:33966039-33966153	+	GTCCCGGTCGCCGCGGTTCGCCGCC	2.07998	2.48334	1.24587	Yes	
NM_hsa_2420	chr12:12715716-12715803	+	TTCCCTTCCAGAGTCCGGCGA	0.113971	0.132445	0.0732862	Yes	
NM_hsa_5196	chr17:37043120-37043204	+	TAAAAACAGGCTCTCGCTCTGTAGC	0.569857	0.662225	0.366431	Yes	
